# Assessing morphology and function of the semicircular duct system: introducing new *in-situ* visualization and software toolbox

**DOI:** 10.1038/srep32772

**Published:** 2016-09-08

**Authors:** R. David, A. Stoessel, A. Berthoz, F. Spoor, D. Bennequin

**Affiliations:** 1 Department of Human Evolution, Max Planck Institute for Evolutionary Anthropology, Deutscher Platz 6, 04103 Leipzig, Germany; 2 Centre de Recherches sur la Paléobiodiversité et les Paléoenvironnements (CR2P, UMR 7207), Sorbonne Universités-MNHN, CNRS, UPMC-Paris6, Muséum national d’Histoire naturelle, CP38, 57 rue Cuvier, F-75005, Paris, France; 3 Collège de France, 11 place Marcelin Berthelot, 75231 Paris, France; 4 Department of Cell and Developmental Biology, University College London, London WC1E 6BT, UK; 5 Université Paris Diderot-Paris 7, UFR de Mathématiques, Equipe Géométrie et Dynamique, Bâtiment Sophie Germain, 8 place Aurélie Nemours, 75013 Paris Cedex 13, France

## Abstract

The semicircular duct system is part of the sensory organ of balance and essential for navigation and spatial awareness in vertebrates. Its function in detecting head rotations has been modelled with increasing sophistication, but the biomechanics of actual semicircular duct systems has rarely been analyzed, foremost because the fragile membranous structures in the inner ear are hard to visualize undistorted and in full. Here we present a new, easy-to-apply and non-invasive method for three-dimensional *in-situ* visualization and quantification of the semicircular duct system, using X-ray micro tomography and tissue staining with phosphotungstic acid. Moreover, we introduce Ariadne, a software toolbox which provides comprehensive and improved morphological and functional analysis of any visualized duct system. We demonstrate the potential of these methods by presenting results for the duct system of humans, the squirrel monkey and the rhesus macaque, making comparisons with past results from neurophysiological, oculometric and biomechanical studies. Ariadne is freely available at http://www.earbank.org.

The semicircular duct system of vertebrates is part of the sensory organ of balance, and one of the smallest and most delicate structures of the head. Located inside the bony labyrinth of the inner ear, it monitors rotational head motion through a cascade of biomechanical events. This involves fluid displacement and cupula deflection, which leads to the modulation of its nerve output to the brain after further signal processing at the junction between hair cells and afferent fibers^1^. The semicircular duct system thus forms a key part of one of the most basic senses shared by all vertebrates, the detection of body motion and orientation, essential for navigation^2^,^3^, motor coordination^4^,^5^,^6^ and spatial awareness^7^. In particular, it helps stabilizing gaze and head motion, through the vestibulo-ocular and vestibulo-colic reflexes^1^,^8^,^9^,^10^,^11^,^12^, provides a stable reference frame for the perception and control of movement^4^,^5^, and also participates in the maintenance of posture through vestibulo-spinal reflexes^13^,^14^. Investigating how the morphology of the semicircular duct system affects function thus not only contributes to a better understanding of sensory physiology in general, but also has a bearing on a wide range of important practical applications that relate to humans, from balance disorders in clinical medicine to performance of pilots and astronauts.

The biomechanics of the semicircular duct system has been modelled with increasing sophistication^15^,^16^, but these models are rarely applied to actual organisms, with the notable exception of toadfish^17^ and humans^18^. Strikingly, primate species used in vestibular research have not yet been analyzed comprehensively, even though this would offer a unique opportunity to correlate modelled biomechanical behavior of the duct system with experimentally obtained neurophysiological data. An important underlying reason preventing analyses of actual species is that the comprehensive models require detailed morphological information, such as endolymph volumes contained in various parts of the duct system. Most morphological studies of the vestibular apparatus have thus far focused on the relatively easy to visualize bony semicircular canals rather than the delicate membranous ducts they enclose^19^,^20^,^21^,^22^,^23^,^24^,^25^,^26^,^27^,^28^,^29^,^30^. Since the degree to which the canals reflect the size and shape of the ducts inside is highly variable such studies cannot provide the essential information for functional analyses. Instead, undistorted and detailed three-dimensional (3D) visualization of the membranous semicircular duct system is required, something that has proved to be hard to achieve. Methods used most recently include dissection^31^, histological serial sectioning^18^ and orthogonal plane fluorescence optical sectioning microscopy^32^. Each of these techniques has particular limitations, by either being destructive with the associated risk of distortion and loss of 3D context, showing insufficient spatial resolution, exposing only a single duct of each specimen, or being only applicable to species with easily accessible inner ears.

Here we present a new, comprehensive and integrated methodological approach to the study of the semicircular duct system which covers both visualization and analysis. An easy-to-apply and non-invasive visualization method based on X-ray micro tomography (micro-CT) for the first time offers high-resolution 3D imagery and quantification of the complete membranous labyrinth *in-situ* inside the petrosal bone. Furthermore, to process the digitized semicircular duct system we introduce Ariadne, a user-friendly software toolbox which provides detailed morphological descriptions and analyses that are the most realistic yet in representing the biomechanical response of the actual specimen. We test these methods by exploring the biomechanics of the semicircular duct system of a human, a squirrel monkey and a rhesus macaque as examples, and compare the results with evidence from neurophysiology, oculometry and previous biometric studies. Binaries, source code and user manuals of Ariadne are freely available at http://www.earbank.org.

## Results

### *In-situ* visualization of the membranous labyrinth

Over the last two decades micro-CT scanning has become the method of choice for non-invasive exploration of animal morphology, as it is widely available and high spatial resolution can be achieved^33^. However, visualizing soft-tissues tends to be problematic because of the lack of contrast resolution. This issue can be addressed by using specific stains to enhance contrast, an approach shown to be effective in a wide range of applications^33^,^34^, including visualization of aspects of the inner ear^35^.

**Figure 1:**
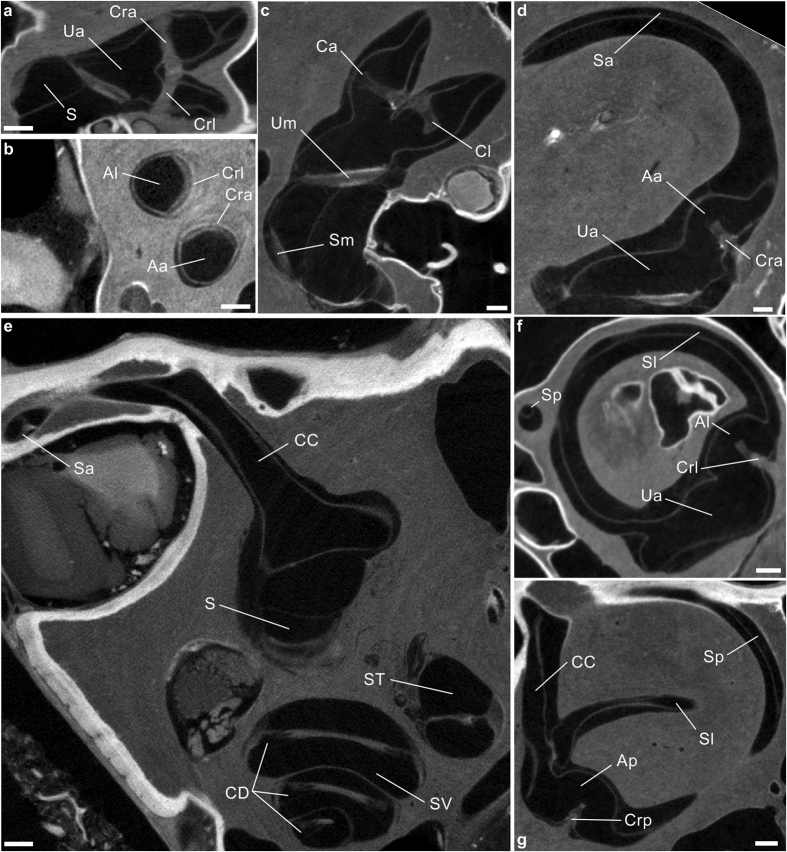
Raw visualizations of stained membranous labyrinths through micro-CT scanning. (**a–g**) Soft-tissues structures contained inside the bony labyrinth of the human (**c,d**), rhesus macaque (**b,e,g**) and squirrel monkey (**a,f**) specimens. Aa, Ap, Al, anterior, posterior and lateral ampullae, respectively; Ca, Cl, anterior and lateral cupulae (artificially shrunk); CC, common crus; CD, cochlear duct; Cra, Crp, Crl, anterior, posterior and lateral cristae ampullares; S, sacculus; Sa, Sp, Sl, slender parts of the anterior, posterior and lateral semicircular ducts; Sm, macula sacculi; ST, scala tympani; SV, scala vestibuli; Ua, anterior utriculus; Um, macula utriculi. Scale bar is 0.5 mm.

**Figure 2:**
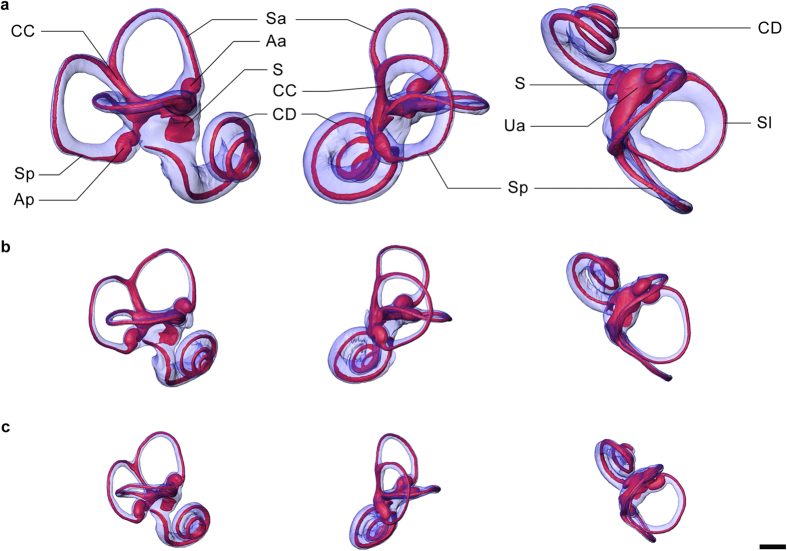
Three-dimensional visualizations of the membranous labyrinth. (**a–c**) From left to right, lateral, posterior and superior views of the bony (blue) and membranous (red) labyrinths of the human (**a**), rhesus macaque (**b**) and squirrel monkey (**c**) specimens. Abbreviations as in Fig. 1. Scale bar is 2.0 mm.

We found that all parts of the semicircular duct system, including the ducts, utricle, ampullae, and cupulae, can be successfully visualized with micro-CT after staining with phosphotungstic acid (PTA)^33^ (Fig. 1) Tissue fixation should happen within a few days after death, preferably using Bouin solution, and a specimen should not have been frozen as this invariably destroys the delicate membranous labyrinth. Visualization with micro-CT requires a voxel size of less than 20 μm, and a scanning protocol which provides the best available image quality, low noise levels in particular. The most productive approach for 3D visualization and digital quantification of the membranous labyrinth, including the semicircular duct system, is by surface rendering rather than volume rendering^36^, based on manual selection of the relevant structures in the CT images (Fig. 2, Supplementary Data 1, 2 and 3). Detailed descriptions of all procedures, from harvesting the petrosal bones to the digitization of the membranous labyrinth, are given in the Methods part.

Thus far we have been able to follow the protocols described here to visualize the semicircular duct systems of 71 different species, including mammals, birds and reptiles, which range in size from a shrew to an elephant. Based on this experience we found that our approach should be applicable to the full range of sizes and morphologies shown by the tetrapod inner ear.

The main limitations of the new visualization method are the requirement to use specimens that have not been frozen, and are either fresh or previously fixed with the appropriate protocol, the potentially lengthy process of staining for up to several weeks in the case of larger specimens, and the need to access a micro-CT scanner which produces high-quality images. Several of these limitations are shared with methods used thus far^18^,^31^,^32^,^35^, and our new approach benefits from an overall simpler, safer and non-destructive production of full 3D imagery of the membranous labyrinth, at high spatial resolution and without the risk of mechanical distortion and loss of 3D context.

Assessing the morphometric accuracy of the semicircular duct systems we visualized is hampered by the absence of reliable benchmark data to compare with. Previous measurements are based on visualization methods that share the potential impact of tissue shrinkage with our approach, but have additional sources of error that follow from invasive procedures (e.g. mechanical distortion in the case of histology or micro-dissection). Hence, reconstructions of the duct system with our method are likely as accurate as currently available, as long as the micro-CT scanner produces accurate, well-calibrated images, a voxel size is selected which is small enough to adequately represent the membranous ducts, and the endolymph segmentation is done with care. It is reassuring to find that measurements taken from the lateral semicircular duct reconstruction of our human specimen match those based on careful micro-dissection^37^ (Supplementary Fig. 1), and previously confirmed by histology^18^.

The precision of reconstructing the semicircular duct system was assessed by analyzing inter- and intra-observer variation of segmented duct lumina (see Methods for details). Intra-observer variation of their cross-sectional area ranges from 1.6% to 5.3% (average: 2.7%, standard-deviation: 1.3%), whereas inter-observer variation ranges from 0.2% to 4.9% (average: 3.1%, standard-deviation: 2.0%). Uniformly applied to the full surface of the reconstructed semicircular duct system, these variations would correspond to a dilation/contraction of 0.09 to 0.24 voxels for the intra-observer variation, and of 0.01 to 0.24 voxels for the inter-observer variation. The implication for calculated biomechanical parameters will be discussed below.

### Overview of Ariadne

**Figure 3:**
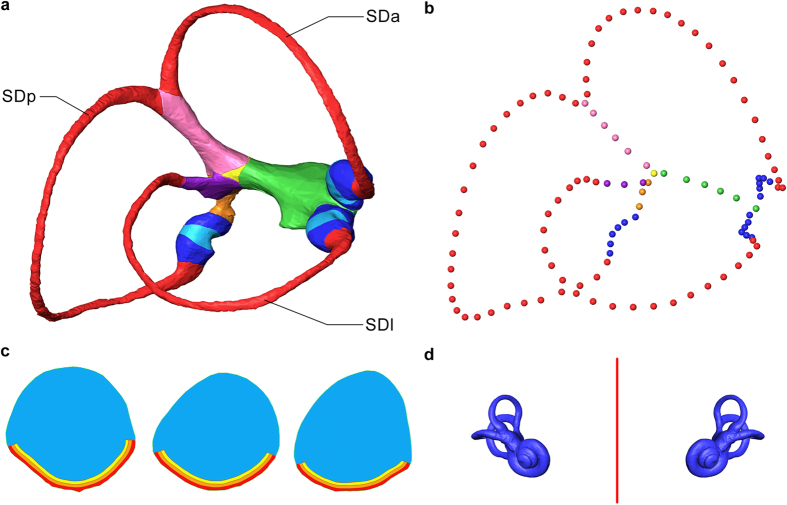
Input data required by Ariadne. (**a**) The right semicircular duct system of the human specimen, with colors marking the parts required as separate STL surface files. SDa, SDp and SDl indicate the anterior, posterior and lateral semicircular ducts, respectively. (**b**) The landmark sets registering the pathway of the central streamlines of the ducts. (**a,b**) Slender ducts shown in red, ampullae in blue, the cupulae in light blue, the common crus in pink, the simple crus in purple, the anterior utricle in green, the posterior utricle in orange and the common utricle in yellow. (**c**) Normal view of the FEA meshes built from the mid-section of, left to right, the anterior, posterior and lateral cupulae. The red, orange and yellow areas along the base are associated with the upward reach of three alternative models of stereocilia/kinocilia length; the blue area represents the rest of the cupula. (**d**) Mid-sagittal plane (red), required for the vestibular reference frame, shown with the bilateral bony labyrinths (blue).

**Figure 4:**
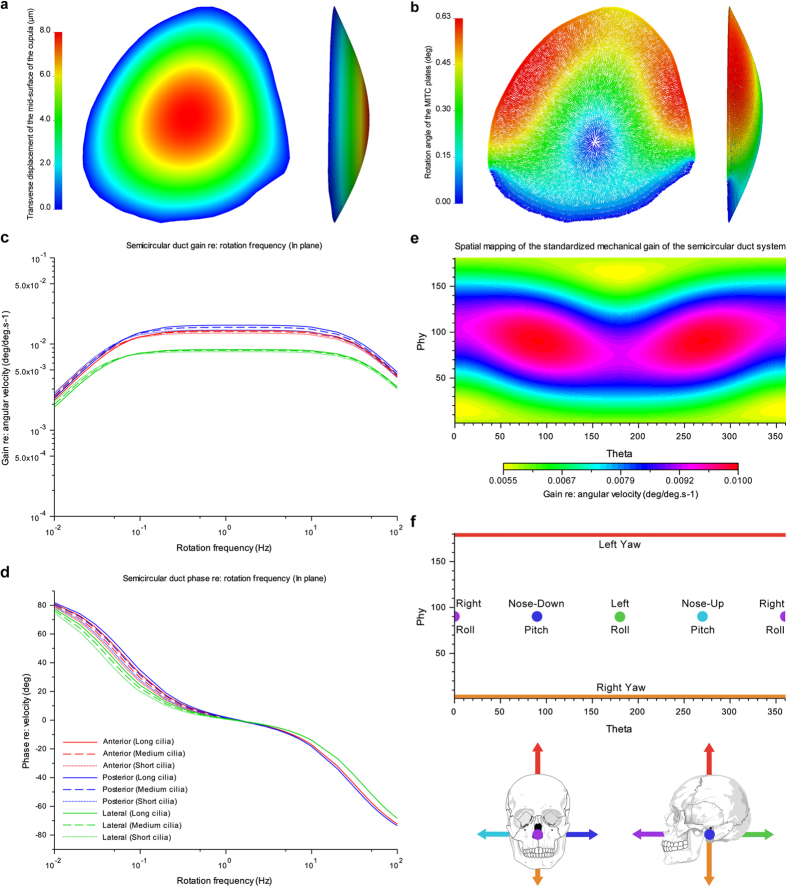
Examples of Ariadne output. (**a,b**) Normal and lateral views of the FEA mesh of the lateral cupula of the human specimen, showing the patterns of transverse displacement (**a**) and rotation (**b**) of its constituting MITC plate elements after the application of a maintained homogeneous pressure of 0.05 Pa at its surface. Mesh deformation has been exaggerated 25 times. (**c,d**) Bode plots illustrating how mechanical gain (**c**) and phase (**d**) of the semicircular duct system of the human specimen are related to head motion frequency. The effect of three alternative stereocilia/kinocilia lengths, short, medium, long, is shown for each duct. (**e**) Mercator projection mapping the predicted standard mechanical sensitivity of the duct system relative to the axis and direction of head rotation. (**f**) The relationship between the coordinates of the Mercator projection maps and the axes of head rotation, with arrows marking the thumb direction following the right hand rule.

The Ariadne software toolbox analyses both the morphology and function of the semicircular duct system. For the latter it implements and expands upon seminal biomechanical models of the three interconnected semicircular ducts^16^ (Methods section and Supplementary Notes 1, 2). The input, obtained from the CT images, consists of a digital description of the endolymphatic fill of the duct system, landmark data characterizing its central streamlines, evidence-based models of the cupulae, and the plane of symmetry of both ears (Fig. 3). The output consists of an accurate description of the functionally important morphological parameters, such as the lengths, cross-sectional areas, enclosed areas, and maximal response planes of the ducts, as well as a complete biomechanical analysis, including Bode analysis and spatial mapping of the mechanical sensitivity (Fig. 4). The preparation of input data is straightforward (Supplementary Note 3), and the results are equally relevant for fundamental vestibular research^1^ or practical applications assessing species-specific sensory specialization, for example in relation to diverse locomotor behaviors^20^,^22^,^26^,^28^,^38^,^39^.

Ariadne consists of 19 executable modules written in C that perform all the computational work required to analyze inputted data. Some of these executables require the free software packages GMSH^40^ (http://geuz.org/gmsh/) and Elmer (https://www.csc.fi/web/elmer/) to be installed. Each executable can be launched independently, or in combination with others using batch files. The user can thus choose either to press one button to get all the morphological and biomechanical results as output, or to launch each executable individually to scrutinize the output in sequential steps. Visualization of biomechanical data can be done optionally through the use of two Scilab (http://www.scilab.org/) scripts provided in the Ariadne package. Ariadne comes with a manual that details the function of all executables, batch files and algorithms that are implemented (Supplementary Note 2).

The biomechanical analyses in Ariadne combine the best available models of the semicircular duct system^15^,^16^ with an improved, more realistic treatment of the cupulae. As viscoelastic structures that deflect under fluid motion they are fully modelled using anatomical cues shown in the CT images (Supplementary Note 3), and their biomechanical properties are directly quantified through finite element analysis under the Mindlin–Reissner theory of plates^41^ (Fig. 4a,b, Supplementary Note 1, 2).

The way the cupulae are treated in Ariadne inherently leads to differences with previous models with respect to values computed for important biomechanical parameters. For example, the mechanical sensitivity, or mechanical gain, reflects the magnitude of the biomechanical response following a given head rotation, and in the past this has been expressed either in terms of mean cupula displacement^15^ or endolymph displacement^16^,^42^. However, if one aims to link mechanical sensitivity with neurophysiological output, it is better expressed in terms of shear strain between the adjacent stereocilia that cover the surface of the *crista ampullaris*^16^. Indeed, while neural discharge response differs from hair bundle deflection because of neural processing^16^, stereocilia deflection is the last aspect of the mechano-transduction that can be studied using morphological evidence. Hence, based on finite element analysis Ariadne calculates and uses the amount of cupula deflection that occurs in the areas where stereocilia and/or kinocilia can be found (Fig. 3c).

Ariadne offers the option to explore the impact of two potential sources of error on the computed morphological and biomechanical parameters. Shrinkage of structures is a phenomenon that has to be considered in any study based on preserved soft-tissues. In the case of the membranous labyrinth this is particularly true when fixatives other than Bouin solution are used. In Ariadne, estimated levels of shrinkage on the output can be taken into account (Supplementary Note 2). Similarly, it is possible to explore how the accuracy and precision of the morphological and biomechanical parameters is affected by the spatial resolution of the images used to prepare a digital model of the semicircular duct system, whether based on micro-CT or scanned histological sections (Supplementary Note 2).

The output of biomechanical analyses can be visualized in the form of Bode plots resulting from pitch, roll, yaw and in-plane canal activation (Fig. 4c,d & Supplementary Note 2), and various maps that precisely characterize the behavior of the system’s mechanical sensitivity within the space of the vestibular frame of reference, which is based on the functional plane of the synergistic pair of lateral semicircular ducts (or canals) and the mid-sagittal plane of symmetry between the bilateral duct or canal systems^43^,^44^ (Fig. 4e,f & Supplementary Note 2). Using this reference frame Ariadne shows the semicircular duct system and any associated structures along the pitch, roll and yaw axes of rotation as a routine application (Fig. 2 & Supplementary Fig. 2). Moreover, the maximal response axes that are part of the biomechanical output can be used to obtain in-plane views for each semicircular duct (Supplementary Fig. 3). These standard view allow for visual comparisons between semicircular duct systems in a replicable and functionally meaningful way.

Biomechanical analyses in Ariadne are mostly based on the morphology of the semicircular duct system that is being analyzed, but three physiological parameters are involved of which little is known across vertebrates. Of the three, endolymph density is similar to that of water^16^ and may remain relatively constant across species, but endolymph viscosity^45^,^46^ and the cupula shear modulus^41^ are expected to vary. The magnitude of this variability is currently unknown but could lead to significant deviations from the biomechanical output that has been computed using default values. Hence, the latter can be changed by the user to explore their impact on the results (Supplementary Note 2).

At this stage Ariadne does not implement some theoretical aspects of semicircular ducts biomechanics that have been considered more recently, but have yet to be evaluated in the literature more widely. These include the effect of head motion frequency on the profiles of endolymph flow and cupula deformation^47^,^48^, and the potential effect of cupula porosity onto its shear modulus^49^. Consequently, semicircular duct biomechanics computed by Ariadne can safely be trusted in the lower and medium parts of the head motion frequency bandwidth, but taking these theoretical developments into account could lead to deviations in the higher part.

### Proof of concept: primate semicircular duct biomechanics

In order to assess the applicability of our methodology, we used Ariadne to analyze the semicircular duct system of a squirrel monkey (*Saimiri sciureus*), a rhesus macaque (*Macaca mulatta*) and a human (*Homo sapiens*). We chose the two non-human primate species because their vestibular neurophysiology is well-studied^50^,^51^, making it possible to compare the biomechanical data produced by Ariadne with experimental neurophysiological information. The semicircular duct system of humans is obviously of great interest generally, making it the prime example to demonstrate both the *in-situ* visualization method and the capabilities of Ariadne. Computed values of the main biomechanical parameters are provided for each semicircular duct of the three test specimens (Tables 1, 2 and 3). To test the effect of segmentation errors when digitizing the semicircular duct system we report the variation of the parameters associated with dilating or contracting its reconstructed surface by the maximum inter-observer variation reported above. Full biomechanical data can be found in the Supplementary Data 4.

The biomechanical long time constant of a semicircular duct reflects the time taken by the cupula to recover its neutral position after a head rotation event. Using Ariadne, we obtained values of 5.7 ± 0.0 s, 6.7 ± 1.2 s and 3.2 ± 0.6 s for the average long time constant of the three ducts in the squirrel monkey, rhesus macaque and human, respectively (±range here and below based on inter-observer variation). Using low adaptation fibers, it is possible to measure the biomechanical long time constant through neurophysiological experiments. Values thus obtained are 5.73 ± 0.23 s for the squirrel monkey^50^ and 7 s for the rhesus macaque^51^. That Ariadne calculated a very similar value for the squirrel monkey cannot be used as independent validation because the neurophysiological results for this species were used in the software to calibrate the default shear modulus of the cupulae (γ = 1.44 Pa). However, the good match between the values for the rhesus macaque does independently corroborate the result calculated by Ariadne.

The value of the long time constant of human semicircular ducts remains uncertain as it cannot be obtained through neurophysiological experiments. Biomechanical studies estimated its average value to be around 10 s^18^, but recent experimental analysis based on the slow phase of the angular vestibulo-ocular reflex, measured from the response to steps of rotation about a yaw axis, provided a value of 4.2 ± 0.6 s^52^. The latter value is closer to the 4.0 ± 0.8 s calculated by Ariadne for the lateral semicircular duct, suggesting consistency between the semicircular duct biomechanics it implements and experimental, oculometry-based physiology.

The short time constant reflects the response speed of the semicircular duct system. Using Ariadne, we obtained values of 1.7 ± 0.1 ms, 2.2 ± 0.1 ms and 4.8 ± 0.2 ms for the average short time constant of the semicircular duct system of the squirrel monkey, rhesus macaque and human, respectively. It remains impossible to measure the short time constant through neurophysiological experiments, and these values can only be compared to those calculated using previous biomechanical models. Values of 4 ms^15^ and 6 ms^16^ were previously computed for the lateral semicircular duct of humans, and 2.1 ms^16^ was found for the lateral semicircular duct of the squirrel monkey. The results provided by Ariadne do not depart drastically from these values and give the first estimation of the short time constant of the rhesus macaque.

Lastly, Ariadne calculated the mechanical sensitivity of the semicircular ducts in the velocity bandwidth (“velocity gain”) as listed in Table 1. These values based on angular cilia deflection cannot be compared directly with previously reported biomechanical sensitivities based on either endolymphatic volume displacement^16^,^18^ or average linear cupula displacement^15^. The aim of our new definition is to get the best possible biomechanical representation of the neurophysiological (afferent nerve) sensitivity, which has been measured for the squirrel monkey^53^ and the rhesus macaque^51^. These results are reported in spikes.s^−1^/deg.s^−1^ and our mechanical sensitivity in mdeg/deg.s^−1^ so that their relationship can only be compared in future research, through correlation analyses of a larger, more diverse species sample. No experimental data exist for humans, but the sensitivity of regular and high-gain irregular fibers of the lateral duct has been estimated by extrapolation, using the linear relationship with the duct’s radius of curvature, as empirically found for a sample of eight mammal species^43^. This finding demonstrates that aspects of neurophysiological sensitivity can be inferred from morphological information. As Ariadne considers the full semicircular duct system, and not just the radius of curvature of a single duct, it can be expected that the mechanical sensitivity calculated here will provide a more realistic and accurate representation. Indeed, some insight into the difference between the two methods can be gained by comparing the results obtained for the three primate species assessed here (Table 3). The mechanical sensitivity shows more distinct interspecific differences than the radii of curvature, and thus any estimates of the neurophysiological sensitivity based on this single parameter. In particular, the human duct system stands out by having a lateral duct that is notably less mechanically sensitive than the anterior and posterior duct, whereas all three ducts are more similar in the two monkeys. This contrast is less pronounced for the radii of curvature, suggesting that incomplete information is obtained when only one of several factors affecting the mechanical sensitivity is considered^43^.

## Discussion

The methods introduced here offer the opportunity to investigate the morphology and function of the semicircular duct system more comprehensively than before, on a large scale and mostly based on actual rather than modelled inner ear structures. The new *in-situ* visualization method makes it possible to obtain substantial sample sizes, both in number of individuals and diversity of tetrapod species. Assuming compliance with relevant legislation, typical sources are human tissue donor programs and veterinary pathology departments for both zoo animals and domesticated species. Moreover, wild animals can be sampled as long as the inner ear is preserved after death within a time limit that depends on environmental temperature.

Ariadne offers the toolbox to analyze large and diverse samples routinely, and without the necessary need for expert knowledge of vestibular biomechanics. Validation of the biomechanical analyses currently implemented is perforce constrained by the limited independent evidence that is available, testament of the great difficulty of accessing the functioning inner ear. However, where comparisons can be made the results obtained for the three primate species examined here are consistent with neurophysiological and oculometric studies. In particular, the match between experimentally measured and independently computed long time constants gives credit to the capability of Ariadne to compute such parameters, endorsing the accuracy of the visualization approach on which the software directly depends. The observed intra- and inter-observer variation of reconstructing the semicircular duct system results in small fluctuations of the biomechanical parameters calculated by Ariadne. These are small compared with differences between the three species examined here, but the full impact can only be explored when larger samples will give better insight into ranges of intra- and inter-specific variation.

The methods described here can be applied in a wide range of studies, dealing with the sensory biology of the organ of balance. Past attempts to associate the semicircular duct system with modes of locomotion have been hindered by visualization that was mostly restricted to the bony labyrinth and by using limited aspects of its morphology^20^,^22^,^26^,^28^,^30^,^38^,^39^,^54^. Now, the mechanical sensitivity, the time constants and the maximal response planes can be assessed for a large number of species with greatly varying locomotor behaviors, including flying, swimming, climbing and bipedal gaits. By examining multiple specimens per species the intraspecific variation of these biomechanical parameters can be quantified. Within a clinical setting, standards can be developed for normal variation of the human semicircular duct system, and the membranous labyrinth in general. Based on post-mortem analyses, attempts can be made to link specific vestibular disorders both morphologically and functionally with unusual duct systems, leading to a better understanding of the biological basis of pathology.

The development of Ariadne is intended as an ongoing project in which researchers in the field of vestibular physiology can expand and modify the current modules and develop new ones as our understanding of inner ear function improves over time.

## Methods

### Specimen acquisition and preparation

Petrosal bones should be sampled as soon as possible after death, up to 72 hours if a specimen is kept in a cool environment (~4 °C). Freezing should be avoided as it destroyed the membranous labyrinth of all the specimens we tested. Petrosals are most easily accessed endocranially, after removal of all or part of the brain, and extracted using an oscillating saw with a straight thin blade. If the two petrosal bones cannot be sampled jointly, a surface scan of the cranial base can be made to document the spatial position(s) prior to extraction. Other methods can be used later to estimate the spatial configuration of the semicircular ducts system (see Supplementary Note 3 – Section I.vi.). Fixation can be done using 4% formaldehyde, but Bouin solution is preferred mainly because it is a very good preservative for soft-tissues, but also a mordant for staining procedures. Fixation with 10% formaldehyde should be avoided as it shrunk the membranous labyrinth of all the specimens we tested. Fixation duration depends on the size of the specimens. We usually leave the samples in Bouin solution for 7 days, but fixation duration as long as 60 days does not seem to affect labyrinth morphology notably. After fixation, the specimens should be rinsed overnight with running water to clean residuals of Bouin solution, followed by dehydration in successive baths of 30% and 50% ethanol for 2 hours each, with final storage in 70% ethanol.

The rhesus macaque (CEB130105) and the squirrel monkey (CEB130037) samples presented in this paper were obtained from the Institute of Veterinary Pathology at Leipzig University. The human sample (CEB130017) was obtained from the Institute of Anatomy at Leipzig University. All three are held in the Comparative Ear Bank (CEB) that is currently housed at the Max Planck Institute for Evolutionary Anthropology in Leipzig, Germany.

### Staining procedure

To visualize the membranous labyrinth with high-resolution computed tomography (micro-CT), the contrast of the soft-tissues needs to be enhanced by staining the samples using phosphotungstic acid (PTA)^33^. In order to prepare 500 ml of 2.5% PTA solution, 50 ml of distilled water was gently added to a beaker with 12.5 g of PTA crystals (Sigma-Aldrich P4006–100G). After gently stirring to allow the crystals to dissolve, the solution was added to 350 ml absolute ethanol and 100 ml water. It was stored in a cool place after stirring the solution for 10 minutes.

To stain the membranous labyrinth the specimens were kept in the 2.5% PTA at room temperature. Our observations from CT images of partially-stained specimens indicate that the staining of the membranous labyrinth begins through diffusion along nerve fibers and later propagates along the vestibular epithelium, without signs of overstaining the specimen. Consequently, the required stain volume and duration of the process both seem to be better reflected by nerve fibers length and size of the membranous labyrinth than by petrosal bone density or thickness. Empirically, we recommend using eight times more solution of 2.5% PTA than the volume of the petrosal bone (160 ml of PTA 2.5% for a 20 ml petrosal bone) to ensure that complete staining of the membranous labyrinth is reached in about 15 days.

Experiments using 2.5% solution of phosphomolybdic acid for staining resulted in the formation of crystals inside and outside the membranous labyrinth when used for Bouin preserved specimens, and this alternative is therefore not recommended.

### Scanning procedure

To visualize the membranous labyrinth, a stained sample should be put in a small plastic tube filled with 70% ethanol, and scanned using a conventional micro-CT scanner. To prevent the sample from moving during the scan process, pressure can be exerted by foam pieces placed on its sides and/or above, but not below as this was found to cause the specimen to move in some cases. Since visualization of labyrinth soft-tissues is primarily limited by the thickness of its thin membranes, a voxel size of between 5 and 15 μm should be used. Our best visualizations of the membranous labyrinth were obtained using 130 kV, 61 μA, a brass filter of 0.25 mm, a rotation step of 0.15 degrees per frame, averaging of 8 frames per step, no random motion, no binning, a 360° acquisition and a “step and shoot” type of motion. This protocol results in scanning time that often exceeds 10 hours, but reducing this time, for example by scanning while continuously rotating the specimen, results in inferior visualization (Supplementary Fig. 4).

For this study, the rhesus macaque, squirrel monkey and human specimens were scanned with a Skyscan 1173 micro-CT scanner, using the parameters recommended above. The resulting images have a spatial resolution of 7.88 μm, 12.9 μm and 13.57 μm, respectively.

### Preparation of input data

To use Ariadne, input data have to be prepared following specific protocols that are summarized below, and are described in Supplementary Note 3 in the form of tutorials. We currently use Geomagic Studio 12 (Raindrop Geomagic Inc, Morrisville, NC, USA) and Avizo 7.1 (Visualization Science Group, Burlington, MA, USA) to implement these protocols. However, other software can be used for data preparation as long as general guidelines and naming conventions described in the tutorials are followed.

Data preparation involves six main steps. First and foremost, the semicircular duct system has to be segmented, excluding neighboring structures like the saccule and the cochlear duct (Supplementary Note 3 – Section I.i). In the stack of CT images the areas that are filled with endolymph have to be delineated, being careful to not include surrounding membranes into the selection. Additionally, the cupulae (if visible), including the stereocilia/kinocilia layers, need to be added to the selection when filling the endolymphatic volume around the *cristae ampullares*. We do the segmentation manually in Avizo 7.1, using the lasso tool in conjunction with a Cintiq 22HD screen-tablet (Wacom, Kazo, Saitama, Japan). After completing the segmentation a three-dimensional (3D) surface mesh of the semicircular duct system has to be computed.

The second step of data preparation consists of dividing the 3D mesh of the duct system into 11 parts that will be used to create 12 surface and 11 volume files that are saved in STL format (Supplementary Note 3 – Section I.ii). These parts correspond to the main anatomical regions of the semicircular duct system. As indicated by their names, the volume STL files are used to compute the volume of endolymph contained inside each part of the semicircular duct system (Supplementary Note 2 - Section I.iii.1). Similarly, the surface STL files are used to compute the surface area of the inner walls of each part (Supplementary Note 2 - Section I.iii.2).

The third step consists of registering the coordinates of the central streamlines passing through each semicircular duct part (Supplementary Note 3 – Section I.iii). Central streamlines reflect the path of endolymph motion inside semicircular ducts under the assumption of laminar flow, and are thus essential to the computation of their 3D biomechanics. Central streamline coordinates are used to compute the maximal area enclosed by the semicircular ducts, the length of each of their parts, their maximal response axes and their corresponding ipsilateral and synergistic angular relationships (Supplementary Note 2 - Sections I.iii.4 & I.iii.8). Central streamlines are defined by placing landmarks on the calculated central line inside 3D meshes of the semicircular ducts system.

The fourth step consists of building a 3D model for the cupula of each semicircular duct from which three volume and 12 surface STL files can be extracted (Supplementary Note 3 – Section I.iv). A complete cupula without shrinkage and visualized *in-situ* (Supplementary Fig. 5) corresponds to an extrusion of the stereocilia/kinocilia layer covering the corresponding *crista ampullaris* towards the roof of the ampulla. Extruding the shape of the *crista ampullaris* toward the roof of the ampulla thus provides a cupula model, with a thickness which can later be adapted in Ariadne by choosing different stereocilia/kinocilia lengths (Supplementary Note 3 – Fig. 22). These 3D models are used to compute the mean cross-sectional area and the mean thickness of each of the three cupulae (Supplementary Note 2 - Section I.iii.6).

The fifth step consists of extracting the central cross-section of each cupula, perpendicular to the streamline. Each cross-section is divided into four parts and saved in STL format (12 files in total). The four parts are three thin areas along the base associated with the upward reach of three alternative models of stereocilia or kinocilia length, plus one representing the rest and largest part of the cupula (Supplementary Note 3 - Section I.v). Values of 30, 60 and 90 μm are considered for upward reach of the cilia because they efficiently bracket the known variation of stereocilia/kinocilia length on the *crista ampullaris* of vertebrates^16^. For each cupula a two-dimensional (2D) finite element mesh is built using the four area files (Supplementary Note 2 - Section I.ii), so that deflection patterns can be analyzed through finite element analysis (Supplementary Note 2 - Section I.iv). This approach results in a more accurate computation of cupula stiffness. It also increases the relevance of computed mechanical duct sensitivity because cilia deflection is closer to afferent nerve output than metrics used traditionally^16^.

The sixth and last step consists of recording the mid-sagittal plane of the vestibular frame of reference (Supplementary Note 3 – Section I.vi). The coordinates of this plane are used for building the complete vestibular frame of reference (Supplementary Note 2 - Section I.iii.8). This step can be skipped if all previous steps were applied for both membranous labyrinths of the same specimen.

### Precision of the segmentation

In order to assess how manual segmentation of the endolymphatic cavities could affect the precision of reconstructing the semicircular duct system, and thus affect biomechanical parameters, three of us (RD, FS and AS) segmented the cross-sectional endolymphatic fill of two semicircular ducts of the squirrel monkey specimen in ImageJ (NIH, Bethesda, Maryland, USA). The observers segmented on different computers with individual monitor settings, without prior discussion of how to identify the membrane/endolymph interface position. To do so, the same slice, the same tool (polygon tool) and the same zoom level (600%) were used. Each cross-section was segmented 10 times per observer, alternating between the two ducts. The cross-sectional areas of all 60 segmented endolymphatic fills were measured in number of voxels and analyzed to study inter- and intra-observer variation in segmentation. For the study of intra-observer variation, the average and standard deviation was calculated for each of the six repeated segmentations (two ducts, by three observers), from which a percentage error was obtained. For the study of inter-observer variation, the six average values computed in the intra-observer variation study were used (two ducts, by three observers), per duct they were paired using each possible combination of observers (RD-FS, RD-AS, FS-AS) and the average between the values of each pair was calculated. The difference between the average value of each observer found in a pair and the average value of that pair was calculated for each of the 6 combinations (two ducts, by three pairs), from which a percentage error was obtained.

To assess how the inter-observer variation affects biomechanical parameters, the reconstructed semicircular duct systems needed to be dilated and contracted by a given number of voxels reflecting this range of variation, everywhere and perpendicularly to the surface. To translate the variation in cross-sectional area into voxel units all around the perimeter of the area, the corresponding variation in the length of the long and short axes was calculated, based on the measured ratio between the long and short axes and considering the cross-sectional shape as an ellipse. This was done for each of the 60 area measurements, and the average of the long and short axes was calculated for each of the six repeated segmentations (two ducts, by three observers). Per duct these averages were paired using each possible combination of observers (RD-FS, RD-AS, FS-AS) and the average of each pair was calculated. Finally, the difference between the average value of each observer found in a pair and the average value of that pair was calculated for each of the six combinations (two ducts, by three pairs), thus giving the voxel errors.

### Overview of Ariadne

At present Ariadne consists of 19 executable files, 8 batch files and 2 Scilab scripts. It runs on Windows NT platforms and needs the freeware software packages Gmsh (http://geuz.org/gmsh/) and Elmer (https://www.csc.fi/web/elmer/) to be installed on the computer. After preparing the primary input files, the use of Ariadne only requires some basic information, such as specimen name and spatial resolution, and subsequently the results of the morphological and biomechanical analyses are provided in specific folders. Descriptions of algorithms implemented in Ariadne and in-depth explanation of using the software is provided in Supplementary Note 2.

After loading the input files Ariadne performs five main tasks: (1) the preparation of the cupula meshes for finite element analysis (Supplementary Note 2 - Section I.ii), (2) the complete description of the morphology of the semicircular duct system, including the cupulae (e.g. lengths, cross-sectional areas, enclosed areas, and maximal response planes of the ducts) (Supplementary Note 2 - Section I.iii), (3) the finite element analysis of cupula deflection (Supplementary Note 2 - Section I.iv), (4) the complete biomechanical analysis of the semicircular duct system, including Bode analysis and spatial mapping of semicircular duct system mechanical sensitivity (Supplementary Note 2 - Section I.v) and (5) the transfer of surfaces, landmarks and maximal response axes from the scan frame of reference to the vestibular frame of reference (Supplementary Note 2 - Section I.vi).

### Ariadne: implementation of the biomechanical model

The biomechanics of the semicircular duct system can be modeled through the second order torsion pendulum equation^16^:


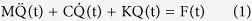


where 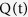, 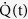 and 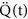 correspond to 3 × 1 vertical vectors describing the volume displacement, velocity and acceleration of the endolymph filling the three semicircular ducts, at a time t, and where M, C and K respectively correspond to the mass, damping and stiffness 3 × 3 matrices of the semicircular duct system.

In this equation, 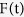 is a 3 × 1 vertical vector representing inertial forces that are applied to the three semicircular ducts through head motion, which are expressed as the dot products:


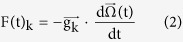


where 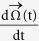 is the vector of head angular acceleration, at a time t; where 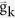 corresponds to a vector representing, by its direction, the axis of rotation that will optimally displace the endolymph contained inside the semicircular duct_k_, and by its magnitude, the ease with which the endolymph will be displaced; and where 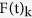 corresponds to the component of the vector 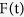 that is related to the semicircular duct_k_.

The torsion pendulum matrices M and C, as well as the vectors 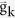 depend on morphological attributes of the semicircular ducts, as well as physiological parameters of the endolymph. The vectors 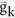 can be expressed as:


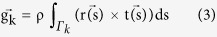


where s denotes the position along the central streamline 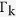 of the semicircular duct_k_, where 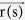 corresponds to a vector running from a fixed origin to position s, where 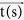 corresponds to a unit vector that is tangent to the central streamline at position s, and where ρ correspond to the density of the endolymph. The vectors 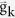 are oriented in such a manner that endolymph flows initially have equal signs in the common parts of the semicircular duct system.

The matrix M can be expressed as:



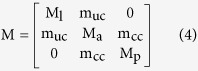



where


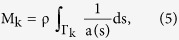



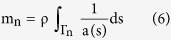


and where s denotes the position along the central streamline 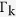 of the anterior_k=a_, posterior_k=p_ and lateral_k=l_ semicircular duct, as well as the position along the central streamline 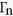 shared between the anterior and lateral semicircular ducts in the anterior utricle_n=uc_, and between the anterior and posterior semicircular ducts in the common crus_n=cc_, and where a(s) corresponds to the cross-sectional area of the semicircular duct part at position s.

The matrix C can be expressed as:


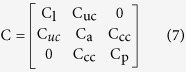


where


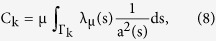



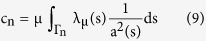


where 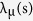 corresponds to the wall shape drag factor of the semicircular duct at position s, and where μ corresponds to the viscosity of the endolymph.

In Ariadne, parameters mentioned above are computed through discrete approximations of these equations, based on the use of 11 discrete landmark sets which represent the central streamline of each part, but also, for M and C, on the volume and surface of each part, from which the corresponding mean cross-sectional area and mean wall shape drag factor are derived. The stiffness matrix K, on the other hand, depends on morphological and physiological attributes of the cupulae and can be expressed as:


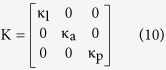


where


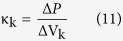


where ΔP corresponds to the differential of all pressures applied upon the cupula of the semicircular duct_k_, and where ΔV_k_ represents the volume of cupula that get displaced in reaction to these pressure applications. In Ariadne, this stiffness parameter is computed through the finite element analysis of 2D cupula models, composed of MITC plate elements to prevent shear and volumetric locking, after application of a homogeneous pressure of 0.05 Pa on one side of the cupula and under the Mindlin–Reissner theory of plates (see Supplementary Note 1). Additionally, the finite element method allows Ariadne to precisely assess cupula deflection occurring in areas where stereocilia and/or kinocilia can be found and thus, to link it to the volume displacement of the cupula through the following equation:


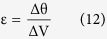


where ε corresponds to the transfer factor for volume displacement to cilia deflection, and where Δθ corresponds to the average cupula deflection occurring in areas where stereocilia and/or kinocilia can be found.

All torsion pendulum parameters are then used to recover the time constants of the semicircular duct system, as well as the mechanical sensitivities s_k_ of the three semicircular ducts. To find the time constants, the torsion pendulum equation is rewritten as the first order system:


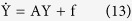


where


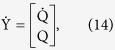



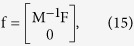



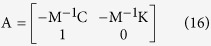


and where the reciprocal opposites of the real part of the eigenvalues of the 6 × 6 matrix A correspond to the six time constants of the semicircular duct system. In Ariadne, the eigenvalues are found through the use of the GNU Scientific Library^55^.

Finally, to find the mechanical sensitivities s_k_, Ariadne implements the following equations:


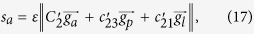



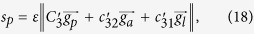



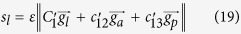


where


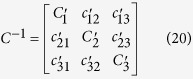


While the biomechanical model of semicircular duct function, as implemented in Ariadne, is primarily based on the above equations, more details on the implementation itself can be found in the Supplementary Note 2.

### Availability of materials and data

Datasets supporting the conclusions of this article are included within the article and its additional files. Additional datasets supporting the conclusions of this article are available a the Comparative Ear Bank website, http://www.earbank.org.

## Additional Information

**How to cite this article**: David, R. *et al.* Assessing morphology and function of the semicircular duct system: introducing new *in-situ* visualization and software toolbox. *Sci. Rep.* **6**, 32772; doi: 10.1038/srep32772 (2016).

## Supplementary Material

Supplementary Information

## Figures and Tables

**Table 1:** Torsion pendulum parameters.

	ASD	PSD	LSD	cc	uc
**M** (mg.mm^−4^)					
*Homo sapiens*	155.7 ± 5.3	176.6 ± 5.9	181.1 ± 6.9	6.9 ± 0.1	2.6 ± 0.0
*Macaca mulatta*	237.5 ± 6.9	278.2 ± 8.3	260.4 ± 7.9	13.5 ± 0.2	3.0 ± 0.0
*Saimiri sciureus*	296.2 ± 16.0	257.6 ± 13.1	297.5 ± 16.5	23.0 ± 0.7	3.6 ± 0.1
**C** (g.s^−1^.mm^−4^)					
*Homo sapiens*	31.2 ± 2.2	32.9 ± 2.2	45.1 ± 3.5	0.3 ± 0.0	0.1 ± 0.0
*Macaca mulatta*	104.1 ± 6.2	125.1 ± 7.6	118.0 ± 7.2	1.5 ± 0.0	0.1 ± 0.0
*Saimiri sciureus*	180.7 ± 20.1	136.4 ± 14.2	187.9 ± 21.1	4.7 ± 0.3	0.3 ± 0.0
**K** (g.s^−2^.mm^−4^)					
*Homo sapiens*	11.1 ± 1.8	12.6 ± 2.1	11.5 ± 1.9		
*Macaca mulatta*	15.5 ± 2.4	16.8 ± 2.6	21.9 ± 3.5		
*Saimiri sciureus*	23.1 ± 2.5	36.0 ± 3.9	33.7 ± 3.7		
**g** (mg.mm^−1^)					
*Homo sapiens*	92.7	101.3	82.1		
*Macaca mulatta*	48.6	49.9	46.3		
*Saimiri sciureus*	32.1	25.6	27.4		
**ε** (mdeg.nL^−1^)					
*Homo sapiens*	268.4 ± 4.9	293.0 ± 5.6	264.6 ± 4.7		
*Macaca mulatta*	447.7 ± 5.8	469.9 ± 6.1	541.7 ± 7.4		
*Saimiri sciureus*	722.2 ± 16.8	862.6 ± 20.5	908.4 ± 22.1		

Given are the coefficient values of the torsion pendulum matrices M, C, K, and vector g, as well as the values of the transfer factor ε for volume displacement to cilia deflection, as calculated by Ariadne for the anterior (ASD), posterior (PSD) and lateral (LSD) semicircular ducts, as well as for the shared common crus (cc) and anterior utricle (uc). Reported errors reflect how dilating or contracting the full surface of the reconstructed semicircular duct systems by the maximum inter-observer error would affect the results. The values of g values are not affected by homogeneous dilation and contraction of the reconstructions.

**Table 2:** Time constants.

	ASD	PSD	LSD
**τ**_**1**_ **(s)**			
*Homo sapiens*	2.9 ± 0.5	2.7 ± 0.5	4.0 ± 0.8
*Macaca mulatta*	6.9 ± 1.3	7.7 ± 1.4	5.5 ± 1.0
*Saimiri sciureus*	7.8 ± 0.0	3.8 ± 0.0	5.6 ± 0.0
**τ**_**2**_ **(ms)**			
*Homo sapiens*	5.0 ± 0.2	5.5 ± 0.2	4.0 ± 0.2
*Macaca mulatta*	2.2 ± 0.1	2.4 ± 0.1	2.2 ± 0.1
*Saimiri sciureus*	1.6 ± 0.1	1.9 ± 0.1	1.6 ± 0.1

Given are the values of the long (τ_1_) and short (τ_2_) time constants, as calculated by Ariadne for the anterior (ASD), posterior (PSD) and lateral (LSD) semicircular ducts. Reported errors as in Table 1. Low error levels for the long time constants of *Saimiri sciureus* are a by-product of using the average neurophysiological long time constant of this species to calibrate the shear modulus (γ) of the cupula. The latter was found to equal 1.45 ± 0.20 Pa depending on dilation or contraction of the reconstructed semicircular duct system of *Saimiri sciureus*. Reported errors for the long time constants of *Homo sapiens* and *Macaca mulatta* reflect the worst combinations of dilation/contraction of the duct system and the error range of γ.

**Table 3:** Mechanical sensitivity and radius of curvature.

	ASD s	PSD s	LSD s	ASD R	PSD R	LSD R
**absolute**						
*Saimiri sciureus*	2.3 ± 0.3	2.9 ± 0.4	2.3 ± 0.3	2.4	2.1	2.2
*Macaca mulatta*	3.7 ± 0.2	3.3 ± 0.2	3.7 ± 0.2	2.9	2.9	2.8
*Homo sapiens*	14.0 ± 0.7	15.7 ± 0.7	8.4 ± 0.5	4.0	4.1	3.7
**relative**						
*Saimiri sciureus*	30 ± 0	39 ± 0	31 ± 0	36	31	33
*Macaca mulatta*	34 ± 0	31 ± 0	35 ± 0	34	34	33
*Homo sapiens*	37 ± 0	41 ± 0	22 ± 0	34	35	31

Given are the absolute values of the mechanical sensitivity (s) in mdeg/deg.s^−1^ and the radius of curvature (R) in mm, calculated by Ariadne for the anterior (ASD), posterior (PSD) and lateral (LSD) semicircular ducts, as well as the relative contribution of each duct as a percentage of the total of the three (100%). Reported errors as in Table 1. The values of R are not affected by homogeneous dilation and contraction of the reconstructions.

## References

[b1] Highstein S. M., Fay R. R. & Popper A. N. The Vestibular System. (Springer-Verlag, New York, 2004).

[b2] Fitzpatrick R. C., Butler J. E. & Day B. L. Resolving head rotation for human bipedalism. Curr. Biol. 16, 1509–1514 (2006). 16890526 10.1016/j.cub.2006.05.063

[b3] Valerio S. & Taube J. S. Head Direction Cell Activity Is Absent in Mice without the Horizontal Semicircular Canals. J. Neurosci. 36, 741–754 (2016). 26791205 10.1523/JNEUROSCI.3790-14.2016 PMC4719012

[b4] Owen B. & Lee D. Establishing a frame of reference for action In Motor development in children: aspect of coordination and control (eds Wade M. & Whiting H.) 341–360 (Martinus Nijhoff, Dordrecht, 1986).

[b5] Berthoz A. Reference frames for the perception and control of movement In Brain and space (ed. Paillard J.) 81–111 (Oxford University Press, USA, 1991).

[b6] Mergner T., Huber W. & Becker W. Vestibular-neck interaction and transformation of sensory coordinates. J. Vestib. Res. 7, 347–367 (1997). 9218246

[b7] Angelaki D. E. & Cullen K. E. Vestibular system: The many facets of a multimodal sense. Annu. Rev. Neurosci. 31, 125–150 (2008). 18338968 10.1146/annurev.neuro.31.060407.125555

[b8] Bronstein A. M. Evidence for a vestibular input contributing to dynamic head stabilization in man. Acta Otolaryngol. (Stockh.) 105, 1–6 (1988). 3257597 10.3109/00016488809119438

[b9] Pozzo T., Berthoz A. & Lefort L. Head stabilization during various locomotor tasks in humans. 1. Normal subjects. Exp. Brain Res. 82, 97–106 (1990). 2257917 10.1007/BF00230842

[b10] Berthoz A. & Pozzo T. Intermittent head stabilization during postural and locomotory tasks in humans In Posture and Gait: Development, adaptation and modulation (eds Amblard B., Berthoz A. & Clarac F.) 189–198 (Excerpta medica, Amsterdam, 1988).

[b11] Dunbar D. C., Badam G. L., Hallgrímsson B. & Vieilledent S. Stabilization and mobility of the head and trunk in wild monkeys during terrestrial and flat-surface walks and gallops. J. Exp. Biol. 207, 1027–1042 (2004). 14766961 10.1242/jeb.00863

[b12] Dunbar D. C., Macpherson J. M., Simmons R. W. & Zarcades A. Stabilization and mobility of the head, neck and trunk in horses during overground locomotion: comparisons with humans and other primates. J. Exp. Biol. 211, 3889–3907 (2008). 19043061 10.1242/jeb.020578 PMC2768006

[b13] Allum J. H., Bloem B. R., Carpenter M. G., Hulliger M. & Hadders-Algra M. Proprioceptive control of posture: a review of new concepts. Gait Posture 8, 214–242 (1998). 10200410 10.1016/s0966-6362(98)00027-7

[b14] Creath R., Kiemel T., Horak F. & Jeka J. J. The role of vestibular and somatosensory systems in intersegmental control of upright stance. J. Vest. Res. 18, 39–49 (2008). PMC2938746 18776597

[b15] Oman C. M., Marcus E. N. & Curthoys I. S. The Influence of Semicircular Canal Morphology on Endolymph Flow Dynamics: An Anatomically Descriptive Mathematical Model. Acta Otolaryngol. (Stockh.) 103, 1–13 (1987). 3494374 10.3109/00016488709134691

[b16] Rabbitt R. D., Damiano E. R. & Grant J. W. Biomechanics of the Semicircular Canals and Otolith Organs in *The Vestibular System* (eds Highstein S. M., Fay R. R. & Popper A. N.) 153–201 (Springer-Verlag, New York, 2004).

[b17] Rabbitt R. D. Directional coding of three-dimensional movements by the vestibular semicircular canals. Biol. Cybern. 80, 417–431 (1999). 10420568 10.1007/s004220050536

[b18] Ifediba M. A., Rajguru S. M., Hullar T. E. & Rabbitt R. D. The Role of 3-Canal Biomechanics in Angular Motion Transduction by the Human Vestibular Labyrinth. Ann. Biomed. Eng. 35, 1247–1263 (2007). 17377842 10.1007/s10439-007-9277-y PMC3005417

[b19] Blanks R. H. I., Curthoys I. S., Bennett M. L. & Markham C. H. Planar relationships of the semicircular canals in rhesus and squirrel monkeys. Brain Res. 340, 315–324 (1985). 3896405 10.1016/0006-8993(85)90928-x

[b20] Spoor F., Wood B. & Zonneveld F. Implications of early hominid labyrinthine morphology for evolution of human bipedal locomotion. Nature 369, 645–648 (1994). 8208290 10.1038/369645a0

[b21] Spoor F. & Zonneveld F. Comparative review of the human bony labyrinth. Yearb. Phys. Anthropol. 41, 211–251 (1998). 10.1002/(sici)1096-8644(1998)107:27+<211::aid-ajpa8>3.3.co;2-m 9881527

[b22] Spoor F., Bajpai S., Hussain S. T., Kumar K. & Thewissen J. G. M. Vestibular evidence for the evolution of aquatic behaviour in early cetaceans. Nature 417, 163–166 (2002). 12000957 10.1038/417163a

[b23] Della Santina C. C., Potyagaylo V., Migliaccio A. A., Minor L. B. & Carey J. P. Orientation of human semicircular canals measured by three-dimensional multiplanar CT reconstruction. J. Assoc. Res. Otolaryngol. 6, 191–206 (2005). 16088383 10.1007/s10162-005-0003-x PMC2504595

[b24] Hullar T. E. & Williams C. D. Geometry of the semicircular canals of the chinchilla (Chinchilla laniger). Hear. Res. 213, 17–24 (2006). 16439079 10.1016/j.heares.2005.11.009 PMC1448857

[b25] Calabrese D. R. & Hullar T. E. Planar relationships of the semicircular canals in two strains of mice. J. Assoc. Res. Otolaryngol. 7, 151–159 (2006). 16718609 10.1007/s10162-006-0031-1 PMC2504575

[b26] Spoor F. *et al.* The primate semicircular canal system and locomotion. Proc. Natl. Acad. Sci. 104, 10808–10812 (2007). 17576932 10.1073/pnas.0704250104 PMC1892787

[b27] Bradshaw A. *et al.* A Mathematical Model of Human Semicircular Canal Geometry: A New Basis for Interpreting Vestibular Physiology. J. Assoc. Res. Otolaryngol. 11, 145–159 (2010). 19949828 10.1007/s10162-009-0195-6 PMC2862918

[b28] Malinzak M. D., Kay R. F. & Hullar T. E. Locomotor head movements and semicircular canal morphology in primates. Proc. Natl. Acad. Sci. 109, 17914–17919 (2012). 23045679 10.1073/pnas.1206139109 PMC3497779

[b29] Ekdale E. G. Comparative Anatomy of the Bony Labyrinth (Inner Ear) of Placental Mammals. Plos One 8, e66624, http://dx.doi.org/10.1371/journal.pone.0066624 (2012). 23805251 10.1371/journal.pone.0066624 PMC3689836

[b30] Pfaff C., Martin T. & Ruf I. Bony labyrinth morphometry indicates locomotor adaptations in the squirrel-related clade (Rodentia, Mammalia). Proc. R. Soc. B 282, 20150744 (2015). 10.1098/rspb.2015.0744 PMC4590456 26019162

[b31] Ghanem T. A., Rabbitt R. D. & Tresco P. A. Three-dimensional reconstruction of the membranous vestibular labyrinth in the toadfish, Opsanus tau. Hear. Res. 124, 27–43 (1998). 9822900 10.1016/s0378-5955(98)00108-7

[b32] Hofman R., Segenhout J. M. & Wit H. P. Three-dimensional reconstruction of the guinea pig inner ear, comparison of OPFOS and light microscopy, applications of 3D reconstruction. J. Microsc. 233, 251–257 (2009). 19220691 10.1111/j.1365-2818.2009.03115.x

[b33] Metscher B. D. MicroCT for comparative morphology: simple staining methods allow high-contrast 3D imaging of diverse non-mineralized animal tissues. BMC Physiol. 9, 11 (2009). 19545439 10.1186/1472-6793-9-11 PMC2717911

[b34] Metscher B. D. X-ray microtomographic imaging of intact vertebrate embryos. Cold Spring Harb. Protoc. 2011, 1462–1471 (2011). 22135670 10.1101/pdb.prot067033

[b35] Uzun H., Curthoys I. S. & Jones A. S. Attachment of the utricular and saccular maculae to the temporal bone. Hear. Res. 233, 77–85 (2007). 17919861 10.1016/j.heares.2007.07.008

[b36] Uzun H., Curthoys I. S. & Jones A. S. A new approach to visualizing the membranous structures of the inner ear – high resolution X-ray micro-tomography. Acta Otolaryngol. (Stockh.) 127, 568–573 (2007). 17503224 10.1080/00016480600951509

[b37] Curthoys I. S. & Oman C. M. Dimensions of the horizontal semicircular duct, ampulla and utricle in the human. Acta Otolaryngol. (Stockh.) 103, 254–261 (1987). 10.3109/00016488709107791 21449649

[b38] Lindenlaub T., Burda H. & Nevo E. Convergent evolution of the vestibular organ in the subterranean mole-rats, Cryptomys and Spalax, as compared with the aboveground rat, Rattus. J. Morphol. 224, 303–311 (1995). 7595956 10.1002/jmor.1052240305

[b39] McVean A. Are the semicircular canals of the European mole, Talpa europaea, adapted to a subterranean habitat? Comp. Biochem. Physiol. A. Mol. Integr. Physiol. 123, 173–178 (1999). 10425737 10.1016/s1095-6433(99)00047-1

[b40] Geuzaine C. & Remacle J.-F. Gmsh: A 3-D finite element mesh generator with built-in pre- and post-processing facilities. Int. J. Numer. Methods Eng. 79, 1309–1331 (2009).

[b41] Selva P., Oman C. M. & Stone H. A. Mechanical properties and motion of the cupula of the human semicircular canal. J. Vestib. Res. 19, 95–110 (2009). 20448336 10.3233/VES-2009-0359

[b42] Muller M. Size Limitations in Semicircular Duct Systems. J. Theor. Biol. 198, 405–437 (1999). 10366494 10.1006/jtbi.1999.0922

[b43] Yang A. & Hullar T. E. Relationship of Semicircular Canal Size to Vestibular-Nerve Afferent Sensitivity in Mammals. J. Neurophysiol. 98, 3197–3205 (2007). 17913986 10.1152/jn.00798.2007

[b44] David R. *et al.* Motion from the past. A new method to infer vestibular capacities of extinct species. C R Palevol 9, 397–410 (2010).

[b45] Oman C. M. The influence of Duct and Utricular Morphology on Semicircular Canal Response In The Vestibular System: Function and Morphology (ed. Gualtierotti T.) 251–274 (Springer, New York, 1981).

[b46] Kate J. H. Ten & Kuiper J. W. The Viscosity of the Pike’s Endolymph. J. Exp. Biol. 53, 495–500 (1970).

[b47] Rabbitt R. D. & Damiano E. R. A hydroelastic model of macromechanics in the endolymphatic vestibular canal. J. Fluid Mech. 238, 337–369 (1992).

[b48] Damiano E. R. & Rabbitt R. D. A singular perturbation model of fluid dynamics in the vestibular semicircular canal and ampulla. J. Fluid Mech. 307, 333–372 (1996).

[b49] Damiano E. R. A poroelastic continuum model of the cupula partition and the response dynamics of the vestibular semicircular canal. J. Biomech. Eng. 121, 449–461 (1999). 10529911 10.1115/1.2835073

[b50] Fernandez C. & Goldberg J. M. Physiology of peripheral neurons innervating semicircular canals of the squirrel monkey. II. Response to sinusoidal stimulation and dynamics of peripheral vestibular system. J. Neurophysiol. 34, 661–675 (1971). 5000363 10.1152/jn.1971.34.4.661

[b51] Haque A., Angelaki D. E. & Dickman J. D. Spatial tuning and dynamics of vestibular semicircular canal afferents in rhesus monkeys. Exp. Brain Res. 155, 81–90 (2003). 15064888 10.1007/s00221-003-1693-0

[b52] Dai M., Klein A., Cohen B. & Raphan T. Model-based study of the human cupular time constant. J. Vestib. Res. Equilib. Orientat. 9, 293–301 (1999). 10472042

[b53] Goldberg J. M., Fernandez C. & Smith C. E. Responses of vestibular-nerve afferents in the squirrel monkey to externally applied galvanic currents. Brain Res. 252, 156–160 (1982). 6293651 10.1016/0006-8993(82)90990-8

[b54] Jones G. M. & Spells K. E. A Theoretical and Comparative Study of the Functional Dependence of the Semicircular Canal upon Its Physical Dimensions. Proc. R. Soc. Lond. B Biol. Sci. 157, 403–419 (1963). 14041997 10.1098/rspb.1963.0019

[b55] Galassi M. *et al.* GNU Scientific Library Reference Manual. (Network Theory Ltd., 2009).

