# Supplementary material for: Assessing morphology and function of the semicircular duct system: introducing new in-situ visualization and software toolbox
Source: Sci Rep. 2016 Sep 8;6:32772. doi: 10.1038/srep32772 (PMC5015051; doi:10.1038/srep32772)
Supplement: Supplementary Information [file srep32772-s1.pdf]

Supplementary Information for: Assessing morphology and function of the semicircular duct system: introducing new in-situ visualization and software toolbox

R. David [a, b,*], A. Stoessel [a], A. Berthoz [c], F. Spoor [a, d,*], D. Bennequin [e]

a Department of Human Evolution, Max Planck Institute for Evolutionary Anthropology, 04103 Leipzig, Germany

b Centre de Recherches sur la Paléobiodiversité et les Paléoenvironnements (CR2P, UMR 7207), Sorbonne Universités-MNHN, CNRS, UPMC-Paris6, Muséum national d'Histoire naturelle, 57 rue Cuvier, CP38, F-75005, Paris, France

c Collège de France, 11 place Marcelin Berthelot, 75231 Paris, France

d Department of Cell and Developmental Biology, University College London, London WC1E 6BT, UK

e Université Paris Diderot-Paris 7, UFR de Mathématiques, Equipe Géométrie et Dynamique, Bâtiment Sophie Germain, 5 rue Thomas Mann, 75205 Paris Cedex 13, France

Supplementary Figure 1: Morphometrics of the human lateral duct. (p.4)

Supplementary Figure 2: The vestibular frame of reference. (p.5)

Supplementary Figure 3: In-plane views of the semicircular ducts. (p.6)

Supplementary Figure 4: Impact of CT scanning protocol on image quality. (p.7)

Supplementary Figure 5: Three-dimensional shape of an unshrunk cupula. (p.8)

Supplementary Note 1: From fluids and Solids to Pendulums. Theoretical note presenting a mathematically exact description of the biomechanics of the semicircular ducts, which is used to evaluate the effect of approximations implemented in Ariadne. (p.9-42)

Supplementary Note 2: Ariadne Manual – Description of the modules. Manual explaining how to use the Ariadne Toolbox and detailed descriptions of the algorithms implemented in the software. (p.43-164)

Supplementary Note 3: Ariadne Manual - Data preparation. Manual explaining how to prepare input data for analysis using the Ariadne Toolbox. (p.165-228)

Supplementary Data 1: Three-dimensional models of the human inner ear. The bony and membranous labyrinths of the human used in this study. Colors as in Figure 2. Units are in meters.

Supplementary Data 2: Three-dimensional models of the rhesus macaque inner ear. The bony and membranous labyrinths of the rhesus macaque used in this study. Colors as in Figure 2. Units are in meters.

Supplementary Data 3: Three-dimensional models of the squirrel monkey inner ear. The bony and membranous labyrinths of the squirrel monkey used in this study. Colors as in Figure 2. Units are in meters.

Supplementary Data 4: Raw outputs of Ariadne. Morphometrical, biomechanical and sensitivity results produced by Ariadne for the specimens analyzed in this study. (p. 229-264)

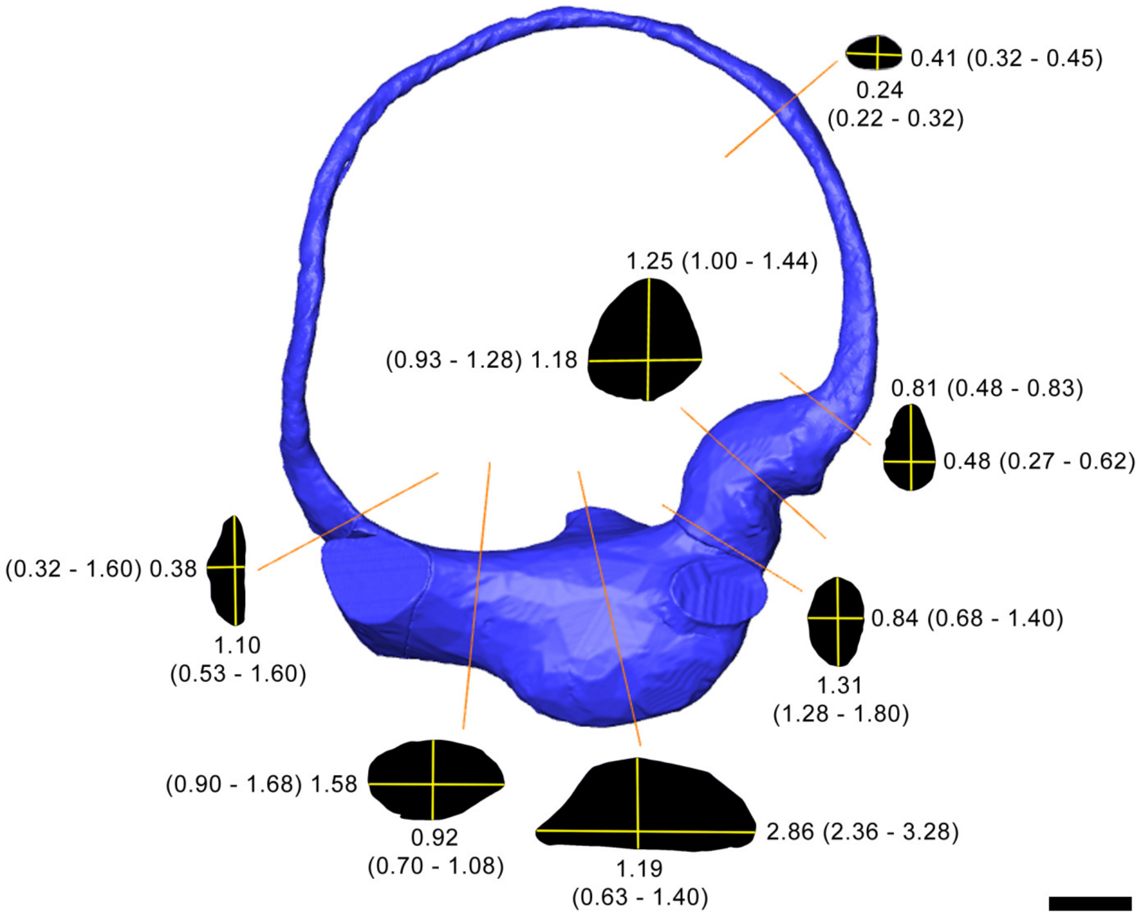

Supplementary Figure 1: Morphometrics of the human lateral duct. The left semicircular duct of the human specimen, with long and short axes measured on cross-sections in 7 locations, according to the protocol of reference 37. Ranges of equivalent measurements obtained in the later study are given in parenthesis. Measurements are provided in millimeters. Scale bar is 1mm.

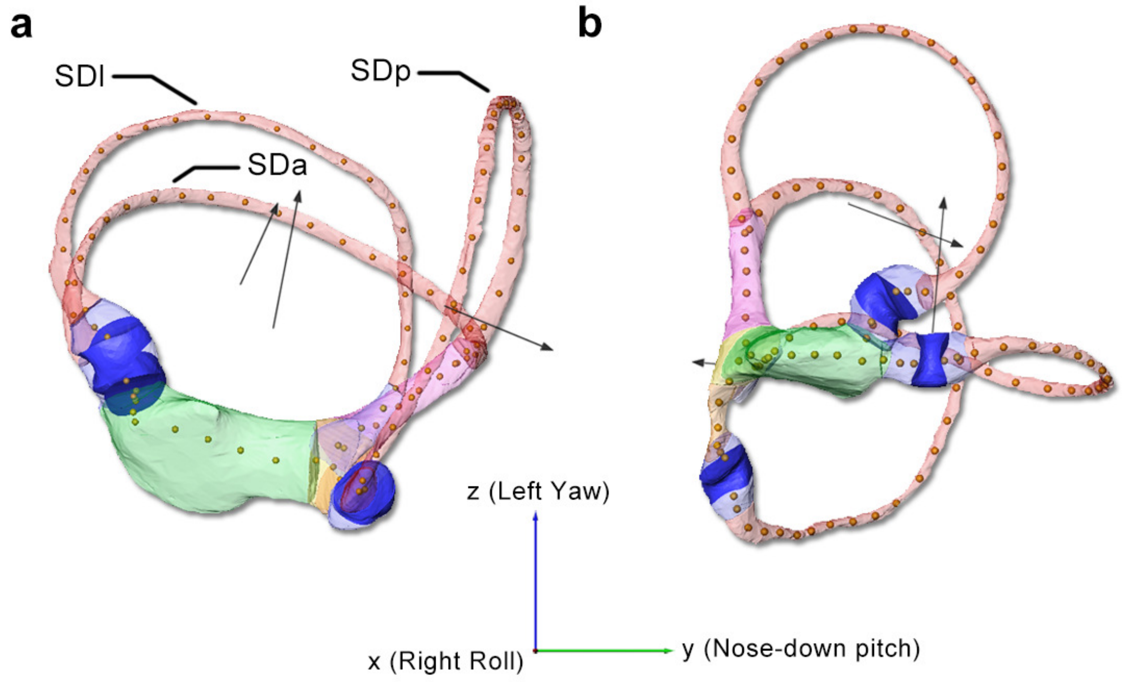

Supplementary Figure 2: The vestibular frame of reference. (a-b) Left semicircular duct system of a human, with the color-coded components rendered transparent to show the landmarks inside. Slender ducts shown in red, ampullae in blue, cupulae in dark blue, the common crus in pink, the simple crus in purple, the anterior utricle in green, the posterior utricle in orange and the common utricle in yellow. Arrows indicate the maximal response axes of each duct, In (a) the system is shown in the reference frame (x,y,z) determined by the random way it was CT scanned. In (b) Ariadne has jointly realigned the duct system, landmarks and response axes with the vestibular frame of reference defined by the vestibular right roll (x), nose-down pitch (y) and left yaw (z) rotations.

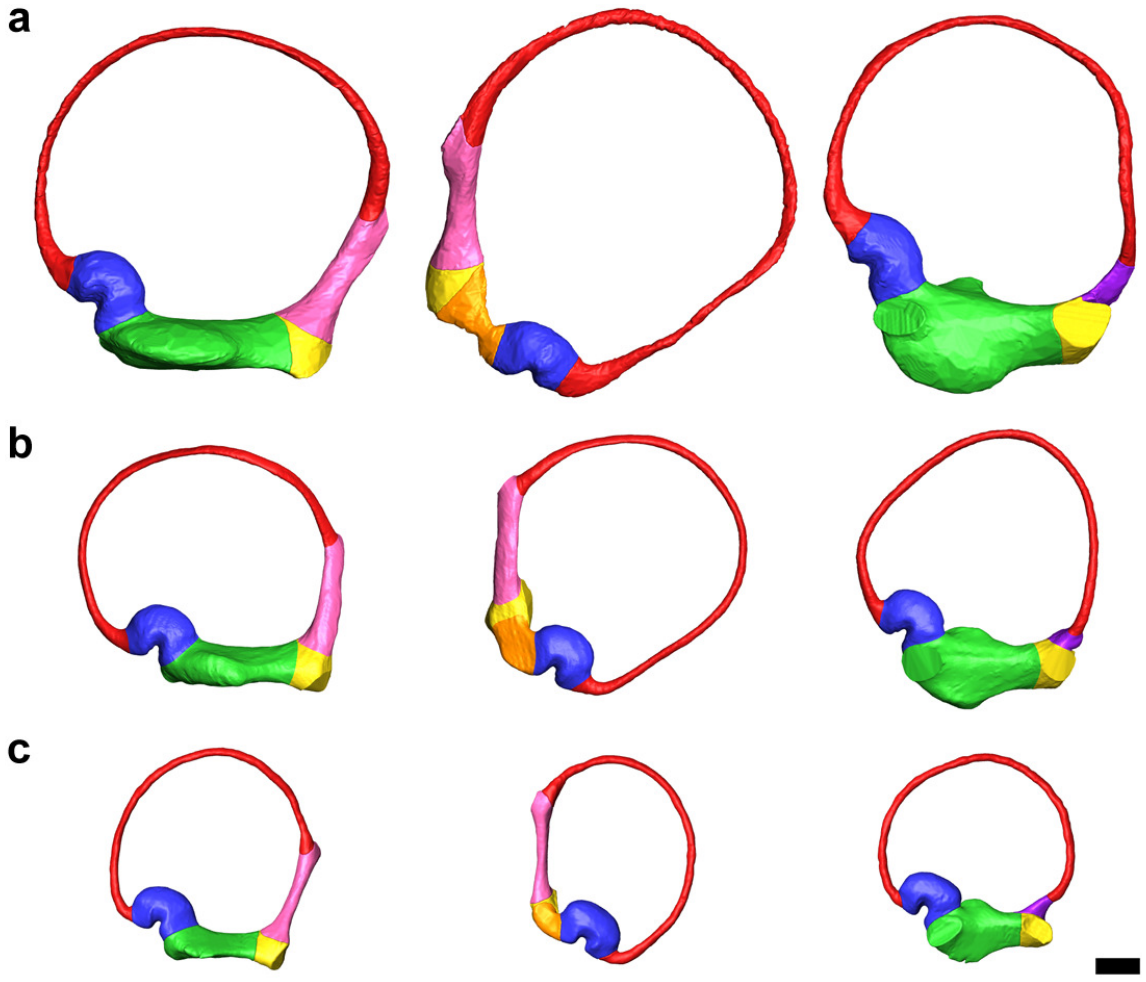

Supplementary Figure 3: In-plane views of the semicircular ducts. (a-c) From left to right, in-plane views of the anterior, posterior and lateral semicircular ducts of the human (a), rhesus macaque (b) and squirrel monkey (c), defined by aligning the maximum response axis of each duct with the viewing direction. Colors as in Supplementary Figure 2. Scale bar is 1.0 mm.

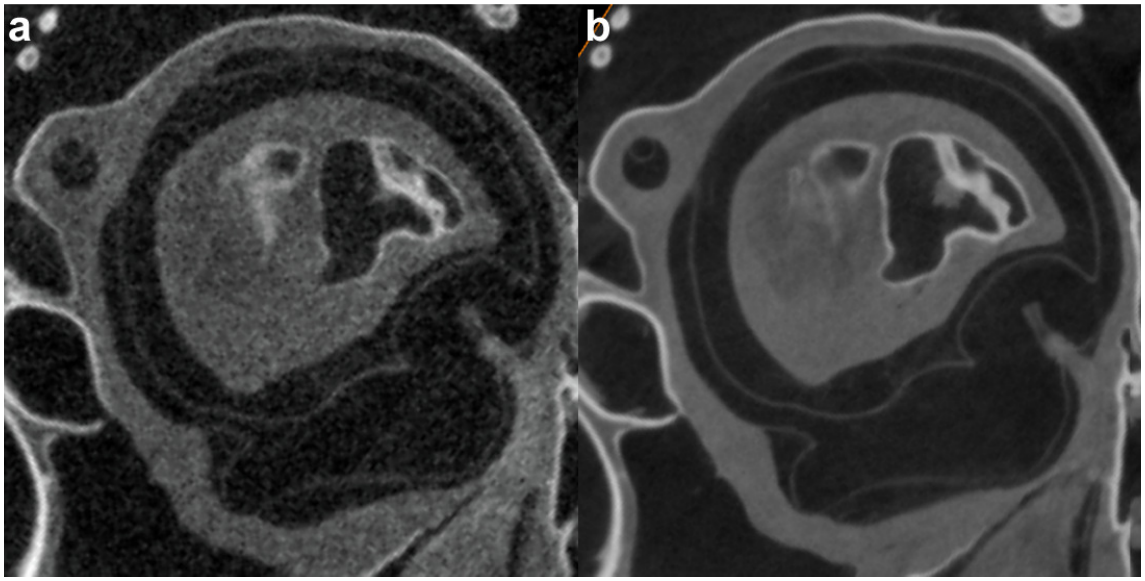

Supplementary Figure 4: Impact of CT scanning protocol on image quality. (a-b) Matching CT images at the level of the lateral semicircular canal of a squirrel monkey, made with (a) a BIR Actis 225/300 micro CT scanner with continuous rotation during data acquisition and no frame averaging, and (b) a Skyscan 1173 micro CT scanner with stepped rotation (”step and shoot”) and averaging eight frames per step. All other parameters are equal, including the scan resolution and duration of soft-tissue staining.

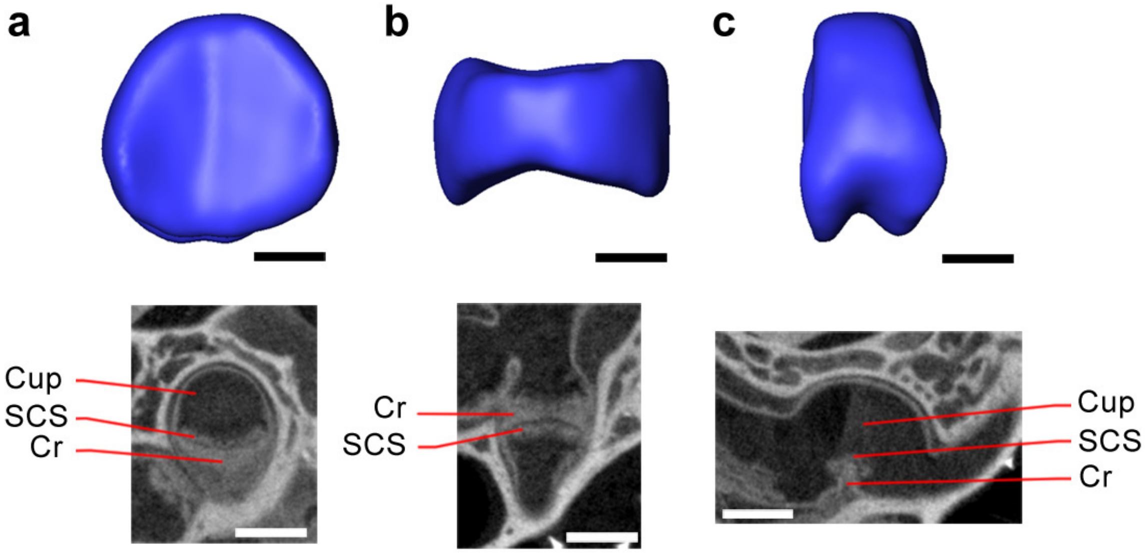

Supplementary Figure 5: Three-dimensional shape of an unshrunk cupula. (a-c) Three-dimensional model of the anterior cupula of *Galago senegalensis* (CEB130084) as seen from (a) the duct entrance to the ampulla, (b) the roof of the ampulla and (c) the side of the ampulla, along with corresponding *in-situ* cross-sections showing the placement of the cupula inside the ampulla. Cup, cupula; SCS, subcupular space; Cr, *crista ampullaris*. Scale bars are 0.2 mm (Black) and 0.5 mm (White).

From fluids and solids to pendulums

1 Second order transfers

A long tradition models the work of semi-circular canals by a second order transfer equation :

$$m_k\ddot{Q}_k(t) + c_k\dot{Q}_k(t) + \kappa_k Q_k(t) = F_k(t), \tag{1}$$

where $k = 1, ..., 6$ indices the canal, m_k, c_k, κ_k are parameters interpreted as *inertia momentum* or *mass parameter*, *viscosity momentum* or *damping parameter* and *effective stiffness* respectively, cf. Victor J. Wilson et Geoffrey Melvill Jones [1].
The letter Q_k denotes the volume of displaced fluid in the semicircular duct near the cupula.
The right member F_k is the scalar product

$$F_k(t) = -m_k\overrightarrow{X_k} \cdot \frac{d\overrightarrow{\Omega}(t)}{dt} \tag{2}$$

of the angular acceleration vector of the head with a fixed vector that measures together the optimal axis of rotation of the given canal, by its direction, and the sensitivity of this canal, by its norm.
Here we have followed the notation of Ariadne ; m is the mass parameter, c is the damping and κ is the stiffness, all depending on the morphological and physical characteristics of the labyrinth and the cupula.
This accords to the notations M, C, K of Damiano et Rabbitt (1992, 1996), [2], [3], Rabbitt, Biological Cybernetics (1999) [4]. Other frequent notations are I for m, B for c and K for κ.
Oman et al., Rabbitt and Damiano (cf. [5], [6]), have suggested formulae for these quantities based on the geometry of the labyrinth. They are integrals

over a central streamline Γ_k, as described in Ariadne :

$$m_k = \int a^{-1}(s)\rho(s)ds \tag{3}$$

$$c_k = \lambda_\mu \int a^{-2}(s)\mu(s)ds \tag{4}$$

$$\kappa_k = \lambda_\gamma \int a^{-2}(s)\gamma(s)ds; \tag{5}$$

where s denotes the arc-length along Γ_k, $\rho(s)$ is the fluid density around s, $\mu(s)$ the viscosity around s, $\gamma(s)$ a stiffness parameter, *a priori* different in the fluid and in the cupula membrane, λ_μ is a constant depending on units and types of dynamics and morphology (for instance $\lambda_\mu = 8\pi$ reflects the stationary Poiseuille flow in a duct with circular section), λ_γ is another constant, and $a(s)$ is the transversal area in the labyrinth, varying in the slender part of the duct, the crus communis, the utriculus or the ampulla part.
Note that the above formula for κ_k in [6] represents the contribution of the duct, and another term has to be added, taking into account the cupula, through the pressure difference.
The vector $m_k\overrightarrow{X_k}$ is given by the maximal enclosed area vector (Oman) :

$$m_k\overrightarrow{X}_k = \rho \int_{\Gamma_k} (\overrightarrow{r(s)} \times \overrightarrow{t(s)})ds \tag{6}$$

These formulas are accepted by Ariadne, with minor modifications, except for the stiffness contribution $\kappa_{c,k}$ of the cupula, which is computed apart, by solving numerically the partial differential equation for the cupula displacement under given applied pressure P :

$$\kappa_k = \frac{P}{a_{c,k}u_{c,k}}; \tag{7}$$

where $a_{c,k}$ is the area of the surface of the cupula, and $u_{c,k}$ the mean displacement of the middle cross section of the cupula. Then λ_γ and γ are estimated from κ_k, which is the reverse of what Rabbitt and Damiano do.

The main goal of this chapter is to suggest another justification for the equations of Oman et al., and Rabbitt and Damiano, in the general case of arbitrarily twisted labyrinths. Our analysis will also make more apparent the reason of our choice for computing the stiffness.
In addition, we will suggest more precise formulas for the transfers coefficients, specially for m_k.

All that relies on a beautiful theorem, conjectured by Lord Kelvin and proved in 1951 by Duff and Spencer using Hodge-deRham theory of harmonic tensors, cf. [7].

Before exposing this general argument, we give a short independent exposition of the computation of long and short time constants, and Bode analysis.

Dividing all the coefficients by the mass parameter and suppressing the index k, we get

$$\ddot{\theta}(t) + B\dot{\theta}(t) + C\theta(t) = f(t); \tag{8}$$

where $B = c/m$, $C = \kappa/m$, $f(t) = F(t)/m$ and $Q(t) = \theta(t)$.
We can suppose, because it is the case in all experiments with semi-circular canals, that B^2 is much larger than $4C$, which guaranties that the fundamental solutions of (8) when $f = 0$ are the linear combinations of two exponential functions :

$$\theta(t) = \alpha_1 e^{-t/T_1} + \alpha_2 e^{-t/T_2}; \tag{9}$$

where the exact expressions of the constants $1/T_1$ and $1/T_2$ are :

$$\frac{1}{T_1} = \frac{1}{2}(B - \sqrt{B^2 - 4C}), \quad \frac{1}{T_2} = \frac{1}{2}(B + \sqrt{B^2 - 4C}). \tag{10}$$

Remark that, due to the negligibility of $1/B$ in front of B/C, the following approximations are generally retained in the present context

$$T_2 \approx \frac{1}{B}, \quad T_1 \approx \frac{B}{C}. \tag{11}$$

Thus T_2 is negligible in front of T_1.

The Bode analysis concerns the case of pure waves inputs :

$$f(t) = f_0 \cos(\omega t + \varphi) \tag{12}$$

In this case there is a unique solution which is also a pure wave with the same frequency ω. It is given by the following exact formula

$$\theta(t) = f_0 T_1 T_2 Re[e^{i(\omega t+\varphi)}(1 + i\omega T_2)^{-1}(1 + i\omega T_1)^{-1}] \tag{13}$$

This can be written explicitly in the standard form :

$$\theta(t) = f_0 G(\omega) \cos(\omega t + \varphi - \omega\Phi(\omega)) \tag{14}$$

The quantity $G(\omega)$ is called the *gain*, and the quantity $\Phi(\omega)$ is called the *phase delay.*

Exact formulas for G and Φ are as follows :

$$G(\omega) = T_1T_2 \frac{\sqrt{(1-\omega^2T_1T_2)^2 + \omega^2(T_1+T_2)^2}}{(1+\omega^2T_1^2)(1+\omega^2T_2^2)}, \tag{15}$$

or, equivalently,

$$G(\omega) = T_1T_2 \frac{\sqrt{1+\omega^2T_1^2+\omega^2T_2^2+\omega^4T_1^2T_2^2}}{(1+\omega^2T_1^2)(1+\omega^2T_2^2)} \tag{16}$$

And

$$\Phi(\omega) = \frac{1}{\omega}\arctan\frac{\omega(T_1+T_2)}{1-\omega^2T_1T_2} \tag{17}$$

or more precisely

$$\cos(\omega\Phi(\omega)) = \frac{1-\omega^2T_1T_2}{\sqrt{1+\omega^2T_1^2+\omega^2T_2^2+\omega^4T_1^2T_2^2}} \tag{18}$$

$$\sin(\omega\Phi(\omega)) = \frac{\omega(T_1+T_2)}{\sqrt{1+\omega^2T_1^2+\omega^2T_2^2+\omega^4T_1^2T_2^2}} \tag{19}$$

It can be easily seen on these formulas that, when ωT_1 is small, these quantities do not vary a lot, the gain is about T_1T_2 which is strictly equal to $1/C$, the phase delay is nearly 0. Also, in an interval of frequency ω between $1/T_1 \approx C/B$ and $1/T_2 \approx B$, when ωT_2 is quite small but ωT_1 is quite large, the gain varies as T_1/ω and the phase delay is around $-\pi/2$, which is interpreted as a regression to the capture to velocity if f is seen as an acceleration. So, it is legitimate to conclude from the model that, when the reproduced frequency varies from C/B to B, the *velocity sensitivity* is estimated by $T_2 \approx 1/B$.
Finally, when both ωT_1 and ωT_2 are large. By developing in the inverses of these terms it is easy to obtain the following approximation :

$$G(t) = \frac{f_0}{\omega^2}\cos(\omega t + \varphi). \tag{20}$$

Remark : in the section below on the models of coupling between the canals pendulum equations, we discuss the extension of the above formulas for G and Φ to the case of a system of second order equations.

2 Navier-Stokes equations

Inside the labyrinth, we assume that the density ρ of the endolymph is constant, which means that the fluid inside is incompressible.
We denote by $\overrightarrow{v}$ the velocity vector field with respect to a fixed inertial Galilean frame, not linked to the head, and we denote by $\overrightarrow{u}$ the velocity vector field with respect to a frame rigidly attached to the head and centred in the gravity centre of the head.
The motion of a point which is fixed in the head, results from a time dependent rotation $R(t)$ and a time dependent translation in space $\overrightarrow{r}(t)$. The instantaneous velocity of the head is given by

$$\overrightarrow{\Omega} = R^{-1}\frac{dR}{dt}. \tag{21}$$

If $\overrightarrow{r}(t)$ is the position of the gravity centre of the head in the fixed frame, and if x denotes a fixed point in the head, the relation between $\overrightarrow{v}(x)$ and $\overrightarrow{u}(x)$ is given by the equation

$$\overrightarrow{u}(x) = R^{-1}.\overrightarrow{v}(x) - \overrightarrow{\Omega} \times \overrightarrow{x} - R^{-1}.\frac{d\overrightarrow{r}}{dt}, \tag{22}$$

In order to write the Navier-Stokes equation of the fluid, we introduce the so called material derivative :

$$\frac{D\varphi}{\partial t} = \frac{\partial\varphi}{\partial t} + \overrightarrow{v}.\overrightarrow{\nabla}\varphi. \tag{23}$$

If $\overrightarrow{G}$ is an external force applied to the fluid, like gravitation, the *balance equation*, which expresses the conservation of momentum, tells

$$\rho\frac{D\overrightarrow{v}}{\partial t} = -\overrightarrow{\nabla}p + \mu\Delta(\overrightarrow{v}) + \overrightarrow{G} \tag{24}$$

This is the Navier-Stokes equation. (Cf. Chorin-Marsden, 2000, [8]).

To determinate ρ, $\overrightarrow{v}$ and p, this equation must be completed by boundary conditions, and equations expressing conservation of mass and energy.

When ρ is assumed constant, the mass conservation is simply expressed by the annihilation of the divergence of the velocity field :

$$div(\overrightarrow{v}) = 0. \tag{25}$$

We consider the membranous labyrinth on one side of the head as a three dimensional manifold M with smooth boundary $\Sigma = \partial M$.

The distance on M comes from its embedding in the Euclidian space $\mathbb{R}^3$.

The energy conservation is expressed by the following integral formula

$$\frac{d}{dt}\int_M \frac{1}{2}\rho\|\overrightarrow{v}\|^2 dV$$
$$= \int_M (\overrightarrow{v}.\overrightarrow{G} - \mu\|\overrightarrow{\nabla}\overrightarrow{v}\|^2)dV - \int_{\partial M}(p\overrightarrow{v} - \mu\overrightarrow{v}.\overrightarrow{\nabla}\overrightarrow{v}).\overrightarrow{dS} \quad (26)$$

In the moving frame attached to the head, the Navier-Stokes takes the following form

$$\rho\frac{\partial\overrightarrow{u}}{\partial t} + \rho\overrightarrow{u}.\nabla\overrightarrow{u}$$
$$= -\overrightarrow{\nabla}p + \mu\Delta(\overrightarrow{u}) + \overrightarrow{G} - \rho\overrightarrow{A} - \rho\frac{d\overrightarrow{\Omega}}{dt}\times\overrightarrow{x} - \rho\overrightarrow{\Omega}\times(\overrightarrow{\Omega}\times\overrightarrow{x}) \quad (27)$$

The proof uses that $div(\overrightarrow{u}) = 0$ and the fact that every operator of the form $\overrightarrow{w}.\overrightarrow{\nabla}$ gives zero when it is applied to a field $\overrightarrow{\omega}\times\overrightarrow{x}$ where $\overrightarrow{\omega}$ doesn't depend on x.
In the formula (27), we recognize on the side of the external force $\overrightarrow{G}$, the inertial force of rotation $\rho\frac{d\overrightarrow{\Omega}}{dt}\times\overrightarrow{x}$, the centrifugal force $\rho\overrightarrow{\Omega}\times(\overrightarrow{\Omega}\times\overrightarrow{x})$ and the inertial force of translation $\rho\overrightarrow{A}$ issued from the linear acceleration $\overrightarrow{A} = d^2\overrightarrow{r}/dt^2$. The resultant of all these forces fields will be written $\overrightarrow{F}$.
A priori we also should be careful with the possible forces of Coriolis, $\overrightarrow{F_c} = 2\overrightarrow{\Omega}\times(d\overrightarrow{x}/dt)$, due to eventual displacements of frontiers inside the domain. In particular we have to discuss if these forces are negligible in our case or not.

As the size of $\overrightarrow{u}(x)$ is very small in natural motions, we will neglect in a first approximation the non-linearity in the velocity, which is the convection term $\rho\overrightarrow{u}.\nabla\overrightarrow{u}$.

As boundary condition, it is convenient to impose that $\overrightarrow{u}(x)$ is zero when x belongs to ∂M.
However, inside each ampulla, the cupula imposes another kind of boundary condition, which tells that the velocity $\overrightarrow{u}(x)$ along the walls coincides with the velocity of the wall.

$$\overrightarrow{u}(x,t) = \frac{\partial\overrightarrow{w}(x,t)}{\partial t}; \quad (28)$$

where $\overrightarrow{w}(x,t)$ denotes the displacement of the wall at the point x and the time t.

The cupula obeys its own dynamical equations, which belong to elasticity theory. As we cannot discard the thickness of the membrane, we found convenient to use the Reissner-Mindlin equation, taking into account the shear deformation, cf. Ciarlet [9]. Then we have a coupling between the Navier-Stokes equation for the fluid and the Reissner-Mindlin equation for the solid.

In fact, the exact elasticity model which is chosen has important influence only on the quantitative result, but it is the final relation between the gradient of pressure and the displacement of the membrane which is theoretically meaningful. And here, we assume that, under physiological conditions, this displacement is proportional to the difference of pressure between both cupula walls. In other terms, we assume that

$$\triangle p = \kappa_{c,k,h} a_{c,k} u_{c,k,h} \tag{29}$$

where $\kappa_{c,k,h}$ corresponds to the stiffness parameter of the cupula of the semicircular duct number k, $a_{c,k}$ corresponds to the Euclidian area of the wall, $u_{c,k,h}$ corresponds to the mean transversal displacement of the middle crosssection of the cupula of the semicircular duct number k, and $\triangle p$ corresponds to an homogeneous difference pressure, applied onto the surface of each cupula. The constant $\kappa_{c,k,h}$ is estimated by simulation when $\triangle p$ equals $0.05 Pa$.

To estimate the displacement of the cupula under constant difference of pressure, ElmerPreparation uses the equilibrium equations of the Reissner-Mindlin model. Hence we indicate how is working this model.
These equations are well adapted to the a finite element method (cf. [9]), and they permit to compute the displacement at every place of the cupula, in particular at the level of stereocilia and kinocilia over the crista ampullaris, which is used by Ariadne.

3 Reissner-Mindlin equations

Coordinates on $\mathbb{R}^3$ are noted (x, y, z) or (x_1, x_2, x_3) ; a smooth closed bounded domain Σ_0 is given in the horizontal plane $\mathbb{R}^2$; its boundary is a smooth curve Γ_0. The standard membrane M_0 is the cylinder on Σ_0 of width H, that is described by (x, y) varying in Σ_0 and z varying between $-H/2$ and $H/2$. Then the deformed membrane M is associated to a vector field F on M_0, in such a manner that every point $X = (x, y, z)$ of M_0 is displaced to

the point $X + F(X)$.
We will write the coordinates of F by

$$F(x, y, z) = (u(x, y, z), v(x, y, z), w(x, y, z)). \tag{30}$$

The hypothesis of Reissner and Mindlin states that F has the following particular form :

$$u(x, y, z) = u_1(x, y) - z\varphi_1(x, y); \tag{31}$$
$$v(x, y, z) = u_2(x, y) - z\varphi_2(x, y); \tag{32}$$
$$w(x, y, z) = w(x, y). \tag{33}$$

Which means that the middle surface Σ_0 is displaced as follows :

$$(x, y) \mapsto (X = x + u_1(x, y), Y = y + u_2(x, y), Z = w(x, y)); \tag{34}$$

and that the points in M_0 over this surface at the altitude z, that is (x, y, z), have the translated coordinates $Z = z + w(x, y)$, and are submitted to a shear displacement proportional to z, which is given by $-z(\varphi_1(x, y), \varphi_2(x, y))$.

A more particular case is described by the hypothesis of Kirchhoff, where it is imposed in addition that $\varphi_1(x, y) = \partial_1 w(x, y)$ and $\varphi_2(x, y) = \partial_2 w(x, y)$. This annihilates the transversal shear strain, contrarily to the hypothesis of Reissner and Mindlin.

The *strain tensor* is the deformation tensor of the metric ; for small deformation, this is the symmetric tensor given by the following linear formulas

$$u_{ij} = \frac{1}{2}(\partial_i u_j + \partial_j u_i) - \frac{z}{2}(\partial_i \varphi_i + \partial_j \varphi_i), \tag{35}$$
$$u_{j3} = \frac{\kappa}{2}(\partial_j w - \varphi_j), \tag{36}$$
$$u_{33} = 0. \tag{37}$$

where i and j vary in $1, 2$.

Here κ is a constant, named the *shear correction factor*.
Frequently the value of κ is taken as $5/6 \approx 0.83$. M.V.V. Murthy [10], gave a theoretical derivation, for the present case of a cylinder M_0 over a surface Σ_0, whatever be this surface, a disc or a rectangle, or our cupula. We are confident in Elmer for taking the best value.
Note that in general, when the width varies, this factor takes smaller values, cf. Gruttmann and Wagner [11].

According to Timoshenko [12], the contant κ corresponds to the ratio of the average shear strain of a section to the shear strain at the centroid. This interpretation seems to work very well at the equilibrium, but not for high oscillations, cf. [11].

The *stress tensor* $(\sigma_{ij})_{i,j=1,2,3}$ is related to the strain tensor by a structural system of equations, which is assumed linear in the theory of Reissner-Mindlin, and more generally in linear elasticity.
The matrix is named the *Hook matrix*.

If we work under the hypothesis that the plate is homogeneous and isotropic, the relation is simply given from the *Young modulus* E, the *Poisson ratio* ν, and the *shear modulus* $G = E/(2(1+\nu))$, by the following formulas :

$$\sigma_{11} = au_{11} + bu_{22}, \tag{38}$$
$$\sigma_{22} = bu_{11} + au_{22}, \tag{39}$$
$$\sigma_{12} = \sigma_{21} = (a-b)u_{12}, \tag{40}$$
$$\sigma_{13} = \sigma_{31} = 2Gu_{13}, \tag{41}$$
$$\sigma_{23} = \sigma_{32} = 2Gu_{23}, \tag{42}$$
$$\sigma_{33} = 0. \tag{43}$$

where $a = E/(1-\nu^2)$ and $b = \nu a$.

Let us introduce the three *separate strain tensors*, by

$$v_{ij} = \frac{1}{2}(\partial_i u_j + \partial_j u_i), \tag{44}$$
$$\varphi_{ij} = \frac{1}{2}(\partial_i \varphi_j + \partial_j \varphi_i), \tag{45}$$
$$w_i = \frac{1}{2}(\partial_i w - \varphi_i). \tag{46}$$

Then define the separate *effective stress tensors*, where i, j vary in $1, 2$, by the following equations :

$$N_{11} = aH(v_{11} + \nu v_{22}), \tag{47}$$
$$N_{22} = aH(\nu v_{11} + v_{22}), \tag{48}$$
$$N_{12} = N_{21} = aH(1-\nu)v_{12}; \tag{49}$$

$$M_{11} = -a\frac{H^3}{12}(\varphi_{11} + \nu\varphi_{22}), \tag{50}$$

$$M_{22} = -a\frac{H^3}{12}(\nu\varphi_{11} + \varphi_{22}), \tag{51}$$

$$M_{12} = M_{21} = -a\frac{H^3}{12}(1 - \nu)\varphi_{12}; \tag{52}$$

$$Q_i = \kappa G H w_i; \quad i = 1, 2, 3. \tag{53}$$

Remind that H denotes the width of the plate.
The quantity $12aH^3$ is denoted by D and named the *bending rigidity* or *bending stiffness.*
The quantity aH is named the *extensional rigidity* or the *extensional stiffness.*

Then the equilibrium equations of the membrane are

$$\sum_{i=1}^{2} \partial_i(N_{ij}) = 0, \tag{54}$$

$$\sum_{j=1}^{2} \partial_j(M_{ij}) - Q_i = 0, \tag{55}$$

$$\sum_{i=1}^{2} \partial_i(Q_i) = -P; \tag{56}$$

where P denotes the pressure applied to the horizontal boundary.

They are named the *Reissner-Mindlin equations.*

They constitute an elliptic system of linear partial differential equations of degree two in the five unknown functions, u_1, u_2, w, φ_1 and φ_2.

It is easy to bypass the hypothesis of homogeneity and isotropy, if it is assumed that the Hook matrix is made by two blocks, a first one $C(1,2;z)$ for the $2D$ deformation, and a second one $C(3;z)$ for the 3-vector depending on the index 3, which is assumed to be diagonal.
The tensors v_{ij}, φ_{ij} and w_i are defined by exactly the same formulas as before. But the tensors N_{ij}, M_{ij} and Q_i are defined by applying linear operators to v_{ij}, φ_{ij} and w_i respectively, which are obtained by integrating in z from $-H/2$ to $H/2$, matrices deduced from the Hook matrix; precisely N_{ij} is obtained by applying the integral of $C(1,2;z)$, M_{ij} by applying the integral of

$-z^2 C(1,2;z)$, and Q_i by applying the integral of $C(3;z)$ multiplied by $\kappa/2$. And finally the equations for N_{ij}, M_{ij} and Q_i have exactly the same form as before.
This could be useful for further investigations, if we want to take care of the fact that the cupula is composite.

The boundary condition can be a *clamped membrane*, which means that the vertical boundary $V_0 = \Gamma_0 \times [-H/2, H/2]$, is totally fixed. Equivalently, in restriction to Γ_0, the five functions are supposed identically zero, or having given values. This is the traditional Dirichlet condition.

Another possible boundary condition is a *supported membrane*, which means that the vertical boundary V_0 is sent into itself, and that the normal derivatives are zero on it ; this condition involves five equations on the functions u_1, u_2, w, φ_1, φ_2 and their derivatives v_{ij}, φ_{ij} and w_i. This is the traditional Neumann condition.

We can also assume that the boundary is partly clamped, partly supported. This is a not less traditional Dirichlet and Neumann mixed condition.

4 Kelvin harmonic streams

Our starting point is a difficult result in Analysis of Duff and Spencer (1951) (cf. [7]), which was conjectured by Lord Kelvin, Sir William Thomson, a long time ago, and which was partially proved in 1941 by Tucker. In our case, it can be stated as follows :

Theorem 1 : for each oriented semi-circular canal C_k there exists a unique smooth vector field $\overrightarrow{N_k}(x)$ in M, which is tangent to the boundary Σ, which satisfies the two equations

$$\overrightarrow{rot}(\overrightarrow{N}_k) = \overrightarrow{0}, \quad div(\overrightarrow{N}_k) = 0, \tag{57}$$

and which has circulation 2π along the canal C_k, but circulation 0 along the two other canals, i.e. for every simples curves Γ'_k which deforms to the stream-lines Γ_k

$$\oint_{\Gamma'_k} \overrightarrow{N_k}(x(s)).d\overrightarrow{s} = 2\pi, \quad \forall l \neq k, \oint_{\Gamma'_l} \overrightarrow{N_k}(x(s)).d\overrightarrow{s} = 0. \tag{58}$$

The equations (57) tell that $\overrightarrow{N_k}$ is a closed and co-closed degree one current, also said harmonic current of degree one. They can be rewritten as

$$\overrightarrow{\nabla} \times \overrightarrow{N}_k = \overrightarrow{0}, \quad \overrightarrow{\nabla}.\overrightarrow{N}_k = 0, \tag{59}$$

The complete reference is [7]

Advanced remark : the general result of Duff and Spencer concerns compact manifolds with boundaries in every dimension and proves the existence of harmonic currents (closed and co-closed, to be defined by generalizations of rotational and divergence in every dimensions) with given integrals on different kinds of geometric sub-complexes, named their periods. The equivalence relation between these sub-complexes which guaranties that periods of closed currents are invariants, is called *homology.* Thus the mentioned result states that all linear functions on sums of sub-complexes can be realized by harmonic currents, in a unique way.
For a membraneous labyrinth, the interesting sub-complexes are curves and surfaces. Oriented closed curves describe the different paths inside the labyrinths ; an homology between two oriented curves is made by a succession of continuous deformations and local surgeries that follow crossing, i.e. an oriented arc AB intersecting an oriented arc CD in a point E gives rise to the two oriented arcs AED and CEB. The homology classes give a precise meaning of the different plans of route contained in the labyrinth. What we call individual semi-circular canals belongs to such classes. Homology in the biological sense allows us to realize them by a set of almost clear pieces of the labyrinth.
The three cupula surfaces in a labyrinth M give a basis of the relative homology $H_2(M, \Sigma; \mathbb{Z})$ and the three virtual semi-circular canals correspond to the dual basis in $H^1(M; \mathbb{Z})$ in the sense of Poincaré-Lefschetz.

A general reference for the arguments underlying our discussion is the book of Georges De Rham, *Differentiable Manifolds. Forms, currents, harmonic forms*, Springer Grundlehren (1984), cf. [13].

Because $\overrightarrow{N}_k$ is closed, i.e. $\overrightarrow{rot}(\overrightarrow{N}_k) = \overrightarrow{0}$, and has integers periods when divided by 2π, the orthogonal planes of $\overrightarrow{N}_k(x)$ generate a family of surfaces $\mathcal{F}_k$. In fact, there exists a function φ_k from M to the standard circle $S^1 = \mathbb{R}/2\pi\mathbb{Z}$, which is an harmonic function, and such that each level of φ_k (i.e. the sets of x in M going to the same angle θ) is a union of components of $\mathcal{F}_k$, which we name $\mathcal{F}_k(\theta)$. Along $\Sigma = \partial M$ the surface $\mathcal{F}_k(\theta)$ is orthogonal to Σ. And everywhere, the following equations holds true :

$$\overrightarrow{N}_k(x) = \overrightarrow{\nabla}\varphi_k(x). \tag{60}$$

The function φ_k can be seen as the most important thing. It is the unique solution of the following optimization problem : to find an angular valued

function φ_k which minimizes the Dirichlet integral

$$I(\varphi) = \int_M \|\overrightarrow{\nabla}\varphi(x)\|^2 dV(x); \tag{61}$$

among the angular functions which satisfy : (i) the Von Neumann boundary condition on ∂M, i.e. the normal derivative $\partial_\nu \varphi_k$ is identically zero ; (ii) the periods conditions : along Γ'_k the function varies from 0 to 2π, but along $\Gamma'_l; l \neq k$, the function has zero total variation.

Remark : in standard cases, when the succession of pieces constituting the canal are almost cylindrical, $\mathcal{F}_k$ is close to the evident family of discs transverse to the stream-line which is used in Ariadne. What is amazing is that for every form of the labyrinth, such a family of surfaces exists for each canal. But in general, these surfaces could have singularities somewhere, making them more complicated than discs. They could even have internal components.

The surfaces $\mathcal{F}_k$ came naturally equipped with a unit of signed area, written $*\alpha_k$, which is in general different from the area induced by M in $\mathbb{R}^3$. If α_k is the dual form of $\overrightarrow{N_k}$, which means that for any vector $\overrightarrow{Z}$ we have $\alpha_k(\overrightarrow{Z}) = \overrightarrow{N_k}.\overrightarrow{Z}$, the form $*\alpha_k$ is the unique form such that, for any 1-form β on M, we have

$$\beta \wedge *\alpha_k = (\beta|\alpha_k)dV. \tag{62}$$

Cf. De Rham [13]. In this case, the density of $*\alpha_k$ with respect to the Euclidian area $d\sigma$ is equal to the norm $n_k(x) = \|\overrightarrow{N_k(x)}\|$ of the vector $\overrightarrow{N_k(x)}$.
The form $*\alpha_k$ is an invariant of the flow of $\overrightarrow{N_k}$; which means that if we consider any sub-piece $U(\theta)$ on a given level $\mathcal{F}_k(\theta)$, then the trajectories of the vector field $\overrightarrow{N_k}$ through the points of $U(\theta)$ make a tube, which intersect any other level $\mathcal{F}_k(\theta')$ in a set $U(\theta')$ over which the integral of $*\alpha_k$ equals its integral over $U(\theta)$.
This generates an important *invariant quantity* of the canal :

Definition : $C_{0,k}$ is the algebraic integral of $*\alpha_k$ on any level set $\mathcal{F}_k(\theta)$. It is independent of the angle θ.
Equivalently, using the Euclidian surface, we have, for any value of θ,

$$C_{0,k} = \int_{\mathcal{F}_k(\theta)} n_k(x) d\sigma(x) \tag{63}$$

Lemma 1 : $2\pi C_{0,k}$ is equal to the minimal value of the integral $I(\varphi)$ of (61),

i.e.
$$C_{0,k} = \frac{1}{2\pi}\int_M n_k^2 dV \tag{64}$$
Proof : by using the definition of φ_k and the invariance of $*\alpha_k$, we obtain
$$\int_M *\alpha_k d\varphi_k = \int_0^{2\pi} d\theta \int_{\mathcal{F}_k(\theta)} *\alpha_k = 2\pi C_{0,k} \tag{65}$$
On the other side, at every point x of M, from the definition of $*\alpha_k$, we have
$$*\alpha_k(x)d\varphi_k(x) = n_k(x)^2 dV(x); \tag{66}$$
which proves the lemma.

5 Projections on the main streams

We make the hypothesis that the cupula is placed in such a manner that at equilibrium, its walls are approximately two level surfaces $\mathcal{F}_k(\theta')$, $\mathcal{F}_k(\theta")$, and we choose the determination of the angle θ such that on one side it is approximately 2π and on the other side it is approximately 0.
Without this hypothesis, there is no chance that the pendulum equations we are now looking for, do exist.
Note the existence of an arbitrary choice for the orientations of the canals. A natural choice is to orient each canal in the sense that corresponds to excitation of afferent neurons, i.e. depolarization of the hair cells over the corresponding crista ; this consists to give to φ_k the values 0 and 2π on the side where a pressure exerted on the cupula has an inhibitory or an excitatory effect respectively. This choice is retained in Ariadne. For the right labyrinth, with the Ampère rule, this gives vectors X_k that points up (resp. in front) for the lateral (resp. the two vertical) canals ; for the left labyrinth, the opposite holds.

To get three scalar differential equations from the Navier-Stokes-Reissner-Mindlin system, we project the Navier-Stokes equations to the direction of the three vector fields $\overrightarrow{N_k}; k = 1, 2, 3$ associated to one labyrinth, and integrate over the volume of the whole labyrinth.
Starting with the equation, valid in the domain M', which is the complementary set of the three cupulae membranes,
$$\overrightarrow{\nabla} p + \rho\frac{\partial \overrightarrow{u}}{\partial t} = \mu\Delta\overrightarrow{u} + \overrightarrow{F}, \tag{67}$$

we obtain

$$\int_{M'} \overrightarrow{\nabla} p.\overrightarrow{N_k} dV + \rho \int_{M'} \frac{\partial \overrightarrow{u}}{\partial t}.\overrightarrow{N_k} dV = \mu \int_{M'} \Delta \overrightarrow{u}.\overrightarrow{N_k} dV + \int_{M'} \overrightarrow{F}.\overrightarrow{N_k} dV. \quad (68)$$

where dV denotes the element of Euclidian volume.
The special properties of $\overrightarrow{N_k}$ allows the use of Stokes formulas for computing the terms involving $\overrightarrow{u}$ in this equation.

Lemma 2 :

$$\rho \int_{M'} \frac{\partial \overrightarrow{u}}{\partial t}.\overrightarrow{N_k} dV = 2\pi\rho \int_{C_k} \frac{\partial \overrightarrow{u}}{\partial t}.\overrightarrow{d\sigma} \quad (69)$$

where C_k is the cupula surface number k, and where $\overrightarrow{d\sigma}$ denotes the unit normal vector times the element of Euclidian area.

Proof : using the fact that $\overrightarrow{u}$ is divergence free, and the relation between $\overrightarrow{N_k}$ and φ_k, the integrand is equal to $\rho div(\varphi_k \partial \overrightarrow{u}/\partial t)$. By applying the Green-Stokes formula, we obtain the integral of $\rho\varphi_k \partial \overrightarrow{u}/\partial t.\overrightarrow{d\sigma}$ over the sum of the boundaries of M'. This sum is made by a part $\partial' M$ of the inner membrane ∂M, where $\overrightarrow{u}$ annihilates, by the cupula surfaces on both sides of C_j, for $j \neq k$, but here φ_k induces the same functions on both sides, thus the two integrals compensate, and last but not least the cupula surfaces on both sides of C_k, where φ_k differs by the constant 2π ; which gives the formula of the lemma.

Lemma 3 :

$$\mu \int_{M'} \Delta \overrightarrow{u}.\overrightarrow{N_k} dV = \mu \int_{C_k} \Delta_k \overrightarrow{u}(t) \overrightarrow{d\sigma}; \quad (70)$$

where Δ_k denotes the two dimensional Laplace operator on the cupula surface C_k.

Proof : first by using that $\overrightarrow{N_k}$ is divergence free, the Green-Stokes formula gives

$$\mu \int_{M'} \Delta \overrightarrow{u}.\overrightarrow{N_k} dV = \mu(\int_{\partial' M} + \sum_j \int_{C'_j} - \int_{C"_j}) \overrightarrow{rot}(\overrightarrow{u}).\overrightarrow{N_k} d\sigma. \quad (71)$$

On the part $\partial' M$, as $\overrightarrow{u}$ is zero, the rotational of $\overrightarrow{u}$ is the vector orthogonal to the derivative $\partial_\nu \overrightarrow{u}$ along the normal to Σ. By using the function φ_k and applying Stokes formula in dimension two, we obtain the circulation of $\overrightarrow{rot}(\overrightarrow{u})$ along the cupula attachment number k, multiplied by 2π. This gives

the second member in the formula of the lemma once we apply again the Stokes formula, but this time applied to the cupula wall considered as a surface with boundary. The terms involving the three boundary parts $C'_j - C"_j$, whatever being the index, k or not k, give 0, because along each cupula the vector field $\overrightarrow{d\sigma}$ reverses its direction and $\overrightarrow{N_k}$ conserves its direction.

For the pressure term, we have a mean of variations along the trajectories of $\overrightarrow{N_k}$:

Lemma 4 :

$$\int_{M'} \overrightarrow{\nabla} p.\overrightarrow{N_k} dV = \triangle p C_{0,k}; \tag{72}$$

where $\triangle p$ is the difference between the pressure on the side $\theta = 2\pi - \epsilon$ of the cupula and its side $\theta = \epsilon$.

Proof : expressed in terms of measures and forms, we have in every point x,

$$\overrightarrow{\nabla} p.\overrightarrow{N_k} dV = dp(x) n_k(x) d\sigma(x) = dp(x) \wedge *\alpha_k(x). \tag{73}$$

Integrating on M', this gives the lemma.

But we know from elasticity the relation

$$\triangle p = \kappa_k Q_k(t); \tag{74}$$

where $Q_k(t)$ is the displaced volume of fluid in the ampulla k.

Remind that along the cupula walls, we have the continuity equation relating velocities of the fluid and the solid : $\overrightarrow{u}(t) = \frac{\partial \overrightarrow{w}}{\partial t}$.

Thus the lemma 2 can directly be expressed with $Q_k(t)$:

$$\rho \int_{M'} \frac{\partial \overrightarrow{u}}{\partial t}.\overrightarrow{N_k} dV = \rho \frac{d^2 Q_k(t)}{dt^2} \tag{75}$$

From the lemma 3, we shall expect the existence of a new constant $C_{1,k}$ such that

$$\mu \int_{M'} \Delta \overrightarrow{u}.\overrightarrow{N_k} dV = \mu C_{1,k} \frac{dQ_k(t)}{dt}. \tag{76}$$

The argument is as follows : first, the formula (70) tells us that the integral of

$\Delta\overrightarrow{u}.\overrightarrow{N_k}$ over the whole labyrinth is equal to the flux of the surface Laplacian $\Delta_k\overrightarrow{u}$ in the ampulla, which in first approximation must be a linear function of the derivative field $\partial\overrightarrow{w}(x,t)/\partial t$ along the cupula ; second, the principal mode of this derivative field is $dQ_k(t)/dt$, the integral over the cupula surface.

However, we will show just below, that with a better accuracy, $dQ_k(t)/dt$ should be here more accurately replaced by a linear combination of the three velocities of displaced volumes in the three semi-circular canals.

Let us begin with the particular case, where we can assume that the section of the canal is a disc and that the velocity $\overrightarrow{u}$ in this canal has an approximate Poiseuille profile :

In polar coordinate of a cylindrical part where $a(s)$ varies slowly, we have

$$u(s,r,\theta) = u_0(s)\frac{R^2 - r^2}{R^2}. \tag{77}$$

One integration over the measure $rdrd\theta$ implies

$$\frac{dQ(t)}{dt} = \frac{1}{2}u_0(s)a(s). \tag{78}$$

But the Poiseuille formula allows us to evaluate $\Delta(\overrightarrow{u})$ in the duct :

$$\Delta u(s,r,\theta) = -4\frac{u_0(s)}{R^2} = -4\pi\frac{u_0(s)}{a(s)}. \tag{79}$$

Then,

$$\Delta\overrightarrow{u} = -4\pi\frac{u_0(s)}{a(s)}\overrightarrow{t} = -\frac{8\pi}{a(s)^2}\frac{dQ_k(t)}{dt}\overrightarrow{t}; \tag{80}$$

which gives, by integrating over a stream-line of length L_k,

$$\mu\int_{M'}\Delta\overrightarrow{u}.\overrightarrow{N_k}dV = -8\pi\mu\frac{dQ(t)}{dt}\int_0^{L_k} ds\frac{1}{a(s)^2}\int_{F_k(\theta)} n_k(x)d\sigma(x) \tag{81}$$

Thus we get the expected proportionality (85), with the constant

$$C_{1,k} = -8\pi C_{0,k}\int_0^{L_k} ds\frac{1}{a(s)^2}. \tag{82}$$

Remark that we do not have used the lemma 3 to establish this formula ; thus using lemma 3 we obtain a relation between $\Delta\overrightarrow{u}$ in the duct and $\Delta\overrightarrow{u}$

along the cupula.

With more general morphologies for the canal considered in Ariadne, like elliptic sections, the constant 8π has to be replaced by a larger constant named λ_μ, cf. [3]. We define

$$c_k = -\mu\lambda_\mu \int_0^{L_k} ds \frac{1}{a(s)^2}; \tag{83}$$

in such a manner that $C_{1,k}/C_{0,k} = c_k/\mu$.

In reality, each canal has common part with another canal, and in this common part the sections are wider than in the duct. The anterior and lateral canals posses a common piece in the anterior utriculus; the anterior and the posterior canals posses a common piece alon the common crux. The preceding argument invites us to suppose that there exist constants λ_{jk} and signs $\epsilon_{jk} = \pm 1$, depending on the chosen orientations, such that, in these common parts,

$$\Delta\overrightarrow{u} = -\frac{\lambda_{jk}}{a(s)^2}(\frac{dQ_k(t)}{dt} + \epsilon_{jk}\frac{dQ_j(t)}{dt})\overrightarrow{t}. \tag{84}$$

Thus, in this part of the volume :

$$\mu\int_{M_{jk}} \Delta\overrightarrow{u}.\overrightarrow{N_k}dV = \mu C^j_{1,k}(\frac{dQ_k(t)}{dt} + \epsilon_{jk}\frac{dQ_j(t)}{dt}); \tag{85}$$

where

$$C^j_{1,k} = -\lambda_{jk}C_{0,k}\int_{L_{0kj}}^{L_{1kj}} ds\frac{1}{a(s)^2}. \tag{86}$$

Then we define

$$c^j_k = -\mu\lambda_{jk}\int_{L_{0kj}}^{L_{1kj}} ds\frac{1}{a(s)^2}; \tag{87}$$

in such a manner that $C^j_{1,k}/C_{0,k} = c^j_k/\mu$.
Thus, when we integrate $\Delta\overrightarrow{u}.\overrightarrow{N_k}$ over the whole canal number k, we obtain a linear combination of the three times derivatives $dQ_j(t)/dt$.

At this stage, we already have succeeded to prove that the joint system of Navier-Stokes and Reissner-Mindlin, projected on the three Kelvin streams, gives a system of three coupled pendulum equations, for $k = 1, 2, 3$:

$$2\pi\rho\frac{d^2Q_k(t)}{dt^2} + C_{0,k}\sum_j c^j_k\epsilon_{jk}\frac{dQ_j(t)}{dt} + C_{0,k}\kappa_k Q_k(t) = \int_{M'}\overrightarrow{F}.\overrightarrow{N_k}dV. \tag{88}$$

We now have to give an estimate for the value of $C_{0,k}$, and to precise what gives the integral of forces at the right side.

6 Approximate formulas

For instance, suppose that the norm function $n_k(x)$ depends only on θ, i.e. it is given by a function $\overline{n_k}(\theta)$, constant along the surfaces $\mathcal{F}_k(\theta)$; then we have, for any θ,

$$C_{0,k} = \overline{n_k}(\theta) a_k(\theta); \tag{89}$$

where a_k denotes the total Euclidian area.

In addition, assume that a trajectory of $\overrightarrow{N_k}$ of length L_k goes from one side of $\mathcal{F}_k(\theta)$ to the other side, being always transverse to the foliation $\mathcal{F}_k$; then the following formula holds true :

Lemma 5 :

$$C_{0,k}^{-1} = \frac{1}{2\pi} \int_0^{L_k} \frac{ds}{a_k(s)} \tag{90}$$

Proof : we use the lemma 1, parameterizing the family $\mathcal{F}_k$ by s, which gives

$$C_{0,k} = \frac{1}{2\pi} \int_0^{L_k} ds \int_{\mathcal{F}_k(s)} n_k(x)^2 d\sigma(x). \tag{91}$$

Then, replacing $n_k(x)$ by $C_{0,k}/a_k(s)$, we obtain

$$C_{0,k} = \frac{1}{2\pi} \int_0^{L_k} ds \frac{C_{0,k}^2}{a_k(s)^2} \int_{\mathcal{F}_k(s)} d\sigma(x) \tag{92}$$

$$= \frac{1}{2\pi} \int_0^{L_k} ds \frac{C_{0,k}^2}{a_k(s)^2} a_k(s); \tag{93}$$

thus, dividing by $C_{0,k}^2$, we get the assertion of the lemma.

As we will now explain, this formula has some validity even if n_k varies on the leaves $\mathcal{F}_k(\theta)$.

Let us look at the simplest case for M, when there exists only one canal; M is a solid torus of large radius 1, and small diameter R. In cylindro-polar coordinates r, θ, z the manifold M is defined by $z^2 + (r-1)^2 \leq R^2$, and the boundary Σ is parameterized by $(1 + R\cos\omega, \theta, R\sin\omega)$, where θ and ω vary between 0 and 2π. In this case, the harmonic function φ_k is θ. The norm of

its gradient is $n_k = 1/r$, then the dual form $*\alpha_k$ is equal to $r^{-1}drdz$, and the Euclidian $d\sigma$ on the surfaces $\mathcal{F}_k(\theta)$ is $drdz$. Thus $C_{0,k}$ is given by the following integral :

$$C_{0,k} = \int_{-R}^{R} dz \int_{1-\sqrt{R^2-z^2}}^{1+\sqrt{R^2-z^2}} \frac{dr}{r} = \int_{-\frac{\pi}{2}}^{\frac{\pi}{2}} R\cos\omega \ln\frac{1+R\cos\omega}{1-R\cos\omega} d\omega \tag{94}$$

We get an asymptotic development in R^2 with coefficients computed from the even Wallis integrals :

$$C_{0,k} = 2\pi \sum_{n=1}^{\infty} \frac{2n!}{2^{2n}(n!)^2} \frac{R^{2n}}{2n-1} \sim \pi R^2 + \frac{\pi}{4}R^4 + \frac{\pi}{8}R^6 + ... \tag{95}$$

Then the approximate formula (90) holds true at the order 2 in R, and we know how to correct it for higher orders.
Note that θ was chosen here to coincide with the curvilinear abscissa s of the streamline. However, if we apply a dilatation factor η to M, the function φ_k continues to be θ, the constant $C_{0,k}$ is changed in $\eta C_{0,k}$, and at first order in $(\eta R)^2$, we continue to have (90), because

$$\frac{1}{\eta C_{0,k}} = \frac{1}{\eta(\pi R^2 + \frac{\pi}{4}R^4 + \frac{\pi}{8}R^6 + ...)} \approx \frac{1}{2\pi}\int_0^{2\pi\eta} \frac{ds}{\pi\eta^2 R^2}. \tag{96}$$

Note that the torsion of the streamline could introduce terms in R^3 in the above development.

A true semi-circular canal is a mixture of a fine tube and a larger room ; in the fine part the above development holds true, depending on curvature, but in the large part the constant approximation is relevant because no significant curvature seems to appear for the utricular cavity or the common crus. Consequently, computing $C_{0,k}$ from the lemma 1, and considering the two pieces independently, we can justify the application of (90), but we also suggest departures as given by (95).

Remark : we cannot exclude that the constants $C_{0,k}$ are better approximated by solving a system of equations like the following one :

$$\forall k; \quad \sum_j A_k^j C_{0,k} = \frac{1}{2\pi}\int_0^{L_k} \frac{ds}{a_k(s)}, \tag{97}$$

which takes into account the common parts of the canals.

Remark that in general it is difficult to find analytic formulas for $C_{0,k}$, but the fact that it is the minimum of the Dirichlet integral over circular functions satisfying Neumann condition and having constrained periods on central streamlines, allows to obtain upper bounds for $C_{0,k}$, i.e. lower bounds for m_k. It suffices to compute $I(\varphi)$ for concrete functions φ.

To summarize, our method indicates that the mass parameter in front of the second order term of the pendulum equation must be

$$m_k = \frac{2\pi\rho}{C_{0,k}}. \tag{98}$$

And our discussion shows that m_k is close to the value suggested by Damiano and Rabbitt.
It also shows how to ameliorate theoretically this value, even for the simpler torus.

7 The sensitivity vectors

The elegance of Kelvin currents is also visible on the manner they eliminate the influence of all external forces except rotation inertia, which comes from the angular acceleration vector.

Lemma 6 : the integral of the scalar product of $\overrightarrow{N_k}$ with $\overrightarrow{G}$, $\overrightarrow{A}$ and $\overrightarrow{\Omega} \times (\overrightarrow{\Omega} \times \overrightarrow{x(s)})$ gives zero.

Proof : it is evident that $\overrightarrow{G}$ and $\overrightarrow{A}$ are gradients of linear potentials, but the third force, which is the centrifugal force, is also a gradient, of a quadratic potential; this follows from the fact that

$$\overrightarrow{x} \mapsto \overrightarrow{\Omega} \times (\overrightarrow{\Omega} \times \overrightarrow{x}) = \|\overrightarrow{\Omega}\|^2(pr_{\overrightarrow{\Omega}} - Id)(\overrightarrow{x}) \tag{99}$$

where $pr_{\overrightarrow{\Omega}}$ denotes the orthogonal projection orthogonal on the axis $\overrightarrow{\Omega}$, is a linear symmetric operator.
But for every global gradient force $\overrightarrow{\nabla}U(x)$, the Stokes formula implies that the integral of $\overrightarrow{\nabla}U(x).\overrightarrow{N_k}dV$ over M is equal to the integral of $U(x) * \alpha_k(x)$ over Σ, which is zero because $*\alpha_k$ is identically zero on Σ.

The movement of the cupula membranes generates *Coriolis forces*, which are localized in the ampullae, where boundaries are moving :

$$\overrightarrow{F}_c(x) = 2\overrightarrow{\Omega} \times \frac{\partial w}{\partial t} = 2\overrightarrow{\Omega} \times \overrightarrow{u}. \tag{100}$$

Here, we must distinguish the case of the cupula C_k and the case of the two other cupulae.
In the first case, we have already supposed that $\vec{u}$ is almost proportional to $\overrightarrow{N}_k$ along both walls of C_k, which gives $\overrightarrow{F}_c.\overrightarrow{N}_k(x) = 0$.
Note that for more turbulent fluid motions, this identity could become false.
On a cupula $C_j; j \neq k$, the mean flux of $\overrightarrow{N}_k$ is zero, which doesn't guaranty the complete annihilation of the integral of $\overrightarrow{F}_c.\overrightarrow{N}_k(x)$ but guaranties that it is small, as soon as $\overrightarrow{u}$ has no large local variation.
In fact the order of Coriolis forces themselves seem to be rather small, even without the above precautions.

We come now to the angular acceleration. Here, we have the triple product identity :

$$\rho \int_M (\frac{d\overrightarrow{\Omega}}{dt} \times \overrightarrow{x}).\overrightarrow{N_k}(x)dV = \rho \int_M (\overrightarrow{x} \times \overrightarrow{N_k}(x)).\frac{d\overrightarrow{\Omega}}{dt}dV \tag{101}$$

This gives the exact theoretical formula of the sensitivity vector of the canal number k :

$$\overrightarrow{X}_k = -\rho \int_M (\overrightarrow{x} \times \overrightarrow{N_k}(x))dV. \tag{102}$$

Let us introduce the *vector of maximal enclosed area* associated to any closed curve Γ in $\mathbb{R}^3$:

$$\overrightarrow{A}(\Gamma) = \oint_\Gamma \overrightarrow{x}(t) \times \frac{d\overrightarrow{x}(t)}{dt}dt \tag{103}$$

Any change of variable in the integral, for instance parameterizations by arc-length $s(t)$, gives the same value for $\overrightarrow{A}(\Gamma)$.
As the circulation of $\overrightarrow{N}_k$ on any closed curve Γ'_k deforming the streamline is equal to 2π, the vector $-\overrightarrow{X}_k/2\pi\rho$ is the normalized mean for $*\alpha_k$ of the vectors of maximal area for the set of all the trajectories $\Gamma'_k(x)$ of $\overrightarrow{N}_k$; this is expressed by the following formula, for any fixed θ :

$$\overrightarrow{X}_k = -2\pi\rho C_{0,k}^{-1} \int_{\mathcal{F}_k(\theta)} \overrightarrow{A}(\Gamma'_k(x)) * \alpha_k(x). \tag{104}$$

And if $n_k(x)$ depends only of θ, we also have

$$\overrightarrow{X}_k = -2\pi\rho a(\theta)^{-1} \int_{\mathcal{F}_k(\theta)} \overrightarrow{A}(\Gamma'_k(x))d\sigma(x). \tag{105}$$

The natural hypothesis which expresses the choice of Oman, Damiano and Rabbitt is that the central streamline corresponds to the mean of the trajectories of $\overrightarrow{N}_k$, in the sense that the vector $\overrightarrow{A}(\Gamma_k)$ is equal to $\overrightarrow{X}_k/2\pi\rho$, i.e.

$$\overrightarrow{X}_k = -2\pi\rho \int_{\Gamma_k} (\overrightarrow{x}(s) \times \overrightarrow{t}(s))ds. \tag{106}$$

With this equality we get the expected formula to get (6) ; this follows from (88) after multiplying by $C_{0,k}^{-1}$.
In fact, by the way we construct Γ_k in Ariadne, it is reasonable to expect that for any differential form $\overrightarrow{V}(x).\overrightarrow{dx}$ defined in the manifold M, the mean of the integral of this form over the trajectories of $\overrightarrow{N_k}$ for the transverse measure $d\sigma$ is equal to its integral on Γ_k. Thus, at least if $n_k(x)$ depends only of θ, the mean of the integral of $\overrightarrow{V}(x).\overrightarrow{dx}$ over the trajectories of $\overrightarrow{N_k}$ for $*\alpha_k$ is close to its integral on Γ_k.

Remark that a stronger assumption could be valid, which implies this vector equality : it would be that the central streamline Γ_k is a solution of the following system of non-linear ordinary differential equations, of the Euler type :

$$\forall\theta, \quad \overrightarrow{x}(\theta) \times \frac{d\overrightarrow{x}(\theta)}{d\theta} = C_{0,k}^{-1} \int_{\mathcal{F}_k(\theta)} n_k(x)\overrightarrow{x} \times \overrightarrow{d\sigma}(x). \tag{107}$$

The right member is given by the Kelvin current. To justify this assertion, the formal difficulty is to prove the existence of a closed solution, and to show it stays close to the stream-line computed in Ariadne. However this approach would have the merit to be canonical.
Note that replacing scalar product of fields with $\overrightarrow{N}_k$ by circulation on a closed curve gives also zero on gradients of scalar potential, then lemma 6 persists to be valid if we replace $\overrightarrow{N}_k$ by Γ_k.

At the end we obtain the system of pendulum equations (where m_k is given by (98), c_k^j by (87) and κ_k by (72)) :

$$m_k \frac{d^2Q_k(t)}{dt^2} + \sum_j c_k^j \epsilon_{jk} \frac{dQ_j(t)}{dt} + \kappa_k Q_k(t) = -m_k \overrightarrow{X}_k.\frac{d\overrightarrow{\Omega}(t)}{dt}; \tag{108}$$

where ϵ_{jk} is a sign ± 1 telling if the two involved canals are oriented in the same manner in their common part or no.

The approximate formulas for m_k and c_k^j and the way to evaluate κ_k are compatible with Ariadne.

8 Questions of coupling

It was suggested by many authors, Muller and Verhagen, Rabbitt, Ifediba et al. (cf. [14], [4], [6], [15]), that a coupling between the canals equations must occur, in particular via the common crus for anterior and posterior canals and via the utricular room for anterior and lateral canals.
We saw in the preceding analysis, that in our approach, a coupling intervenes naturally through a damping matrix $c_k^j; j, k = 1, 2, 3$.

Consequently we decided to include in the algorithm of Ariadne, the possibility of a general linear coupling between the pendulum equations, according to the models that were introduced by the above mentioned authors.

To describe this coupling we follow the presentation of [4], [6] and [15] with minor modifications. The quantities used in this approach are all accessible on Ariadne.
In this section, we orient the canals in such a manner that they have the same orientations in their common parts ; this is possible because the pair lateral and posterior has no common part, then it is sufficient to reorient the lateral canal to achieve the goal, this is done in the corresponding intermediate computational algorithms of Ariadne. This choice was made by Ifediba et al. [15].

Let us denote respectively by $1, 2, 3$ the lateral (i.e. horizontal), anterior and posterior canals. The interactions $(1, 2)$ and $(2, 3)$ correspond respectively to the anterior utriculus and to the common crux. As Rabbitt, Ifediba et al. *loc cit.* we neglect a possible very small interaction $(1, 3)$, but the following discussion would not be changed by this interaction, except the necessary intervention of a sign minus for the interaction $(1, 3)$.
Also we are working in the hypothesis of an impermeable canal, thus we equal the flux near the cupula number k with the flux Q_k.
Then we define the vertical vector Q with the three components Q_1, Q_2, Q_3. The 3 masses are replaced by a 3×3 symmetric matrix of mass M, whose diagonal elements are the $M_k; k = 1, 2, 3$ we have defined before as m_k, and whose off diagonal elements correspond to the common segments of

the canals :

$$M = \begin{pmatrix} M_1 & m_{12} & 0 \\ m_{12} & M_2 & m_{23} \\ 0 & m_{23} & M_3 \end{pmatrix} ; \tag{109}$$

where $M_1 = m_{11} + m_{12}$, $M_2 = m_{22} + m_{12} + m_{23}$, $M_3 = m_{33} + m_{23}$, if m_{jj} denotes the mass of the slender duct number j alone.
Note that the matrix M has a strict dominant principal diagonal (each element along the diagonal has an absolute value which is larger than the sum of the absolute values of the other elements in the corresponding line), thus M is *invertible.*
In the same manner the viscosity constants are replaced by a 3×3 matrix of mass C, whose diagonal elements are the $C_k; k = 1, 2, 3$ and off diagonal elements correspond to the common segments of the canals :

$$C = \begin{pmatrix} C_1 & c_{12} & 0 \\ c_{12} & C_2 & c_{23} \\ 0 & c_{23} & C_3 \end{pmatrix} ; \tag{110}$$

where $C_1 = c_{11} + c_{12}$, $C_2 = c_{22} + c_{12} + c_{23}$, $C_3 = c_{33} + c_{23}$, with c_{jj} denoting the viscosity of the slender duct number j.
Note that again this matrix is invertible by diagonal domination, which is important for the computation of velocity sensitivity, as exposed below.
The stiffness parameters are maintained in a diagonal matrix K

$$K = \begin{pmatrix} \kappa_1 & 0 & 0 \\ 0 & \kappa_2 & 0 \\ 0 & 0 & \kappa_3 \end{pmatrix} . \tag{111}$$

It is again invertible.
And finally, the inertial forces are represented now by the vertical vector F of components F_1, F_2, F_3, that are obtained by taking the scalar products of the vectors $-M_k X_k$ with the angular acceleration vector of the head, with respect to the head.

The coupled equations can be written in matrix form as follows :

$$M\ddot{Q} + C\dot{Q} + KQ = F, \tag{112}$$

or

$$\ddot{Q} + M^{-1}C\dot{Q} + M^{-1}KQ = M^{-1}F. \tag{113}$$

As noticed in Rabbitt 1999, 2004, to compute the solutions of this second order system, it is simpler to rewrite it as a first order system, by introducing the vectors of six components

$$Y = Q^* = \begin{pmatrix} \dot{Q} \\ Q \end{pmatrix} \tag{114}$$

and

$$f = \begin{pmatrix} M^{-1}F \\ 0 \end{pmatrix} \tag{115}$$

Then the equation becomes

$$\frac{dY}{dt} = AY + f \tag{116}$$

with

$$A = \begin{pmatrix} -M^{-1}C & -M^{-1}K \\ 1 & 0 \end{pmatrix} \tag{117}$$

The eigenvalues of the matrix A are the six frequencies which replace the three pairs of basic frequencies of the canals.
They are given by the characteristic equation :

$$det(\lambda Id_6 - A) = det(\lambda^2 Id_3 + \lambda M^{-1}C + M^{-1}K) = 0. \tag{118}$$

Under the hypothesis that the diagonal coefficients of C^2 are much larger than the diagonal coefficients of MK, which is the case in every labyrinth we know, these eigenvalues are real numbers.
Moreover, when the off diagonal coefficients of M and C are small with respect to the diagonal elements, these eigenvalues are close to the usual individual high and low frequency constants.

The Bode analysis is easily extended to the matrix case as follows :
when a $6D$ forces vector $f(t) = f_0 \cos(\omega t + \varphi)$ of given frequency ω is applied a unique solution Y exists which has the same frequency. It is given by

$$Y_f(t) = Re(e^{i\omega t + i\varphi}(i\omega - A)^{-1})f_0. \tag{119}$$

Proof : the solution that corresponds to $f(t) = f_0 \exp(i\omega t + i\varphi)$ and has initial value Y_0 for $t = 0$ is

$$\begin{aligned} Y(t) &= e^{At}Y_0 + \int_0^t e^{A(t-u)} f_0 e^{i\omega u + i\varphi} du \\ &= e^{At}(Y_0 + \int_0^t e^{u(i\omega - A)} f_0 e^{i\varphi} du) \\ &= e^{At}(Y_0 + (i\omega - A)^{-1}(e^{t(i\omega - A)} - Id)) f_0 e^{i\varphi}) \\ &= e^{At}(Y_0 - (i\omega - A)^{-1} f_0 e^{i\varphi}) + (i\omega - A)^{-1} f_0 e^{i\omega t + i\varphi}. \end{aligned}$$

Consequently the unique solution of frequency ω corresponds to $Y_0 = (i\omega - A)^{-1} f_0 e^{i\varphi}$, and is given by $Y(t) = (i\omega - A)^{-1} f(t)$. To get the announced

result we just have to take the real part.

Let us compute the matrix $(i\omega - A)^{-1}$, which corresponds to the gain and phase delay for the coupled system, up to multiplication by the matrix with two diagonal blocks equal to M.
We have to find 3×3 matrices A', B', C', D' such that

$$\begin{pmatrix} i\omega + M^{-1}C & M^{-1}K \\ -1 & i\omega \end{pmatrix} \begin{pmatrix} A' & B' \\ C' & D' \end{pmatrix} = \begin{pmatrix} 1 & 0 \\ 0 & 1 \end{pmatrix} \tag{120}$$

By identification, we find

$$A' = i\omega C', \quad B' = i\omega D' - Id, \tag{121}$$

$$C' = (M^{-1}K + i\omega(i\omega Id + M^{-1}C))^{-1}, \tag{122}$$

$$D' = C' i\omega(i\omega Id + M^{-1}C). \tag{123}$$

When we come back to the $3D$ vector Q, this gives

$$Q_f(t) = Re(C'M^{-1}F(t)) = Re((K + i\omega(i\omega M + C))^{-1}F(t)); \tag{124}$$

where $F(t) = F_0 \exp(i\omega t + i\varphi)$.

$\omega = 2\pi f$.

As in the scalar case, we find that for ω very small, the gain is well approximated by K^{-1} and the phase delay is zero ; thus the *sensitivity to acceleration* is well defined by the formula

$$\widetilde{AG_k} = K_k^{-1}(M_k X_k.Z). \tag{125}$$

If the matrix K has off-diagonal coefficients, the formula for AG_k should involve the other vectors X_j.
The maximal response arises for Z proportional to X_k, thus a measure of the *acceleration gain* is $K_k^{-1}M_k\|X_k\| = K_k^{-1}\|G_k\|$.
However, it is in some sense better to speak of an *acceleration gain vector* :

$$\overrightarrow{AG_k} = K_k^{-1}M_k X_k = K_k^{-1}G_k. \tag{126}$$

For intermediary ω, at least when ωC is much larger than K, the gain varies approximatively as $C^{-1}\omega^{-1}$, the inverse of the viscosity matrix, in place of the diagonal matrix of the inverses of separate viscosities, and the phase delay is $-\pi/2$, which is the characteristic of a regression to angular velocity.

Then *velocity sensitivity* associated to a given ampulla k is deformed according to C^{-1}, more precisely, if

$$C^{-1} = \begin{pmatrix} C'_1 & c'_{12} & c'_{13} \\ c'_{21} & C'_2 & c'_{23} \\ c'_{31} & c'_{32} & C'_3 \end{pmatrix}, \tag{127}$$

and if an oscillatory angular rotation of frequency ω in intermediary range is applied about a certain axis Z, then the response of the ampulla number k, will be proportional to ω^{-1} and to the coefficient

$$\widetilde{VG_k} = C'_k(M_k X_k.Z) + c'_{kj}(M_j X_j.Z) + c'_{kl}(M_l X_l.Z) \tag{128}$$

where k, j, l is a permutation of $1, 2, 3$.
Here, the predicted maximal response corresponds to the *velocity gain vector* :

$$\overrightarrow{VG_k} = C'_k M_k X_k + c'_{kj} M_j X_j + c'_{kl} M_l X_l. \tag{129}$$

We could name its norm the *velocity gain* of the canal number k.

Finally for high frequency ω the gain is $\omega^{-2} M^{-1}$ with a phase delay of π. Correspondingly, the *vector of sensitivity* associated to a given ampulla k is deformed according to M^{-1}, more precisely, if

$$M^{-1} = \begin{pmatrix} M'_1 & m'_{12} & m'_{13} \\ m'_{21} & M'_2 & m'_{23} \\ m'_{31} & m'_{32} & M'_3 \end{pmatrix}, \tag{130}$$

the vectors $X_k; k = 1, 2, 3$ have to be replaced by the vectors

$$\widetilde{X_k} = M'_k M_k X_k + m'_{kj} M_j X_j + m'_{kl} M_l X_l \tag{131}$$

where k, j, l is a permutation of $1, 2, 3$.
This expression gives the modification of the sensitivity vector due to the coupling, in this linear model.

From these computations, the above model of coupling has the following effect on the three functional characteristics of the canals we are reporting, time constants, vector sensitivity and velocity sensitivity :

The *intrinsic frequencies* of the coupled system are the 6 eigenvalues of the matrix A. In what follows we assume that A is diagonalizable, which is true for a perturbation of the decoupled canals, but could be false in general.

Therefore, to estimate the intrinsic frequencies at the ampulla number k, we have to express the two canonical vectors e_k, $\dot{e}_k$ of $\mathbb{R}^6$ that are associated to the index k, in the basis of the six eigenvectors of A, then in general all the frequencies play a role for each ampulla. However, for canals possessing fine slender ducts, as mammals or birds, the eigenvectors will be close to the combinations of e_k and $\dot{e}_k$ that correspond to the large and small time constants. Consequently, we can attribute to each ampulla a perturbed pair of time constants, the other eigenvalues being taken into account with small coefficients.
Only for labyrinths where the small radius of section is of the same order as the utriculus or the crux communis, the six eigenvalues can contribute significatively to the dynamic of each cupula.

To determine the more precise structure of gains and phases, we must compute the inverse $L(\omega)^{-1}$ of the complex matrix $L(\omega) = K + i\omega C - \omega^2 M$,

$$L^{-1}(\omega) = \begin{pmatrix} L'_1 & L'_{12} & L'_{13} \\ L'_{21} & L'_2 & L'_{23} \\ L'_{31} & L'_{32} & L'_3 \end{pmatrix}, \tag{132}$$

then we define the *true velocity gain vector* by

$$\overrightarrow{TVG_k} = \omega(L'_k M_k X_k + L'_{kj} M_j X_j + L'_{kl} M_l X_l). \tag{133}$$

Without multiplying by ω, we would have obtained the acceleration gain, and multiplying by the square factor ω^2, we would have obtained the rotation gain.
It is expected that the gain in function of frequency has the shape of a trapezoid, equals to zero for small and high frequencies, then the *functional frequency band for angular velocity* would be defined by the interval of frequencies where this vector is nearly constant.

Note that $\overrightarrow{TVG_k}$ is a complex vector, i.e. its coordinates are complex numbers $a_k^j + ib_k^j; j = 1, 2, 3$, where the numbers a_k^j and b_k^j are real, and depend of ω.
The norm of this vector is the square root of the sum of the three squares of moduli :

$$\|\overrightarrow{TVG_k}\| = \sqrt{\sum_j (a_k^j)^2 + (b_k^j)^2}; \tag{134}$$

it can be interpreted as the maximal velocity gain for the given frequency.
The interpretation of the phase is more subtle : in fact, for each given ω, the response is maximal for a given set of differences of frequency between

the three components of the angular velocity vector Ω given in input, corresponding to the negative of the differences between the three arguments $\arctan b_k^j/a_k^j$. Furthermore, the *mean* of these three arguments can be interpreted as the generalisation of the *phase shift* ; which is expected to be 0 for small frequencies, $-\pi/2$ for intermediate and $-\pi$ for high frequencies :

$$\triangle\Phi_k = \frac{1}{3}(\sum_j \arctan b_k^j/a_k^j). \tag{135}$$

Remark a better notion for the mean could be given by taking into account the sizes of the various components, as for instance

$$\widetilde{\triangle\Phi_k} = \frac{1}{\|\overrightarrow{TVG_k}\|^2}(\sum_j (a_k^j)^2 + (b_k^j)^2) \arctan b_k^j/a_k^j). \tag{136}$$

Technical remark : the argument of a (non-zero) complex number $z = a + ib$ is an oriented angle ψ, i.e. a real number modulo 2π, but the usual formula for $\arctan b/a$ gives *a priori* a real number in the interval $[-\pi/2, \pi/2]$, which is only a representant modulo π. To obtain the number ψ, we have to use both $\arccos a/|z|$ and $\arcsin b/|z|$ (where $|z| = \sqrt{a^2 + b^2}$) which give respectively numbers in $[0, \pi]$ and in $[-\pi/2, \pi/2]$. But this is not the main technical problem for Bode diagrams, the main problem in this case is to obtain a continuous function $\widetilde{\psi}(\omega)$ with values in $]-\infty, +\infty[= \mathbb{R}$. This map is a lift of the angle $\psi(\omega)$, taken modulo 2π. Practically, for very small frequencies ω, the choice for ψ is zero, and increasing ω gives a unique continuous lift $\widetilde{\psi}(\omega)$, that goes to $-\pi/2$ for the functional band for angular velocity, then goes to $-\pi$ for the very large frequencies. Probably the worst way is to use $\arctan b/a$ because it is strongly discontinuous over $\pm\pi/2$, corresponding to $b/a = \pm\infty$, and the best way is to use $-\arccos a/|z|$.

What is the gain and phase shift of a given canal k, in a given direction of rotation Z, not necessarily optimal ?
We normalize the vector Z to be real and of norm 1 ; then we apply the matrix L' to the vector of components $M_j X_j.Z; j = 1, 2, 3$. This gives a complex number depending on ω ; then its norm is the gain, its argument is the phase shift.

Note the natural generalization : we start with a complex vector Z of norm 1, thus having different phases for each components in pitch, roll and yaw ; the apply the preceding method ; we get a gain and a phase shit which depends

on these differences of phases. A biological interpretation, specially for the optimal set of differences of phases, could be that pitch, roll and yaw are optimally not registered in synchrony, but with optimal asynchrony.

Technical remark : the inversion of the matrix L can be made by using the usual Cramer formulas, where the multiplications of real numbers are replaced by the multiplications of complex numbers.
Another manner to perform the computation consists to replace any complex number $z = \alpha + i\beta$ in the matrix L by the 2×2 matrix given by

$$z = \alpha I + \beta X = \begin{pmatrix} \alpha & -\beta \\ \beta & \alpha \end{pmatrix} \tag{137}$$

This gives a 6×6 matrix L'_c in place of L', which has the same form, of a 3×3 table of 2×2 blocs of the preceding type. Then the formula (133) gives a vector of 2×2 matrix.
Note that the modulus $|z| = \sqrt{\alpha^2 + \beta^2}$ is given by the square root of the determinant of the matrix in (137), and the argument $\theta = \arctan \beta/\alpha$ is also given by $\theta = \arccos \alpha/|z|$ or $\theta = \arcsin \beta/|z|$.

9 Conclusion

In what precedes we gave theoretical arguments for the existence of three coupled second order transfer equations governing the displaced fluid volume in the three ampullae, starting solely from the Navier-Stokes equations for the endolymph and the Reissner-Mindlin equations for the cupula membrane, in the case of the most general geometry for the labyrinth.

The parameters for damping c_k^j, and stiffness κ_k agree with the computations of Ariadne. The way the stiffness parameter κ_k is computed in Ariadne, which departs from the tradition, accords fully with the presented theoretical analysis.

The theoretical formulas (98) for the mass parameter m_k and (102) for the sensitivity vector $\overrightarrow{X_k}$ slightly differ from the formulas used by Ariadne ; but we established in this note that the approximations used by Ariadne are valid. In the case of m_k, this is because the departure in the fine part is of order two in the area of the section, and the departure in the larger segments is negligible in virtue of their straightness. In the case of $\overrightarrow{X_k}$, we have *a posteriori* confirmations, for instance coming from the results on time constants, but we also exposed reasons to expect that the streamline is a

good approximation of the mean of trajectories of the Kelvin harmonic current $\overrightarrow{N_k}$ associated to the canal, thus justifying the approximate formula in Ariadne.

Exact computations of m_k and $\overrightarrow{X_k}$ could in theory be achieved through a finite elements analysis applied to the whole triangulation of the labyrinth.

It was suggested by several groups, Muller and Verhagen, Rabbitt, Ifediba et al. (cf. [14], [4], [6], [15]), that a coupling between the canals equations must occur, in particular via the common crus for anterior and posterior canals and via the utricular room for anterior and lateral canals. Correlatively, the dynamical model of canals functioning should be ameliorated by taking in account this coupling. Thus we included in Ariadne a general coupling of the equations, following the linear model of Rabbitt et al. [6]. We have applied this method to our data for human, rhesus macaques and squirrel monkeys labyrinths. We found that coupling introduces no significant difference with respect to the absence of coupling for these species.
Remark that the canonical Kelvin harmonic streams $\overrightarrow{N_k}$ implicitly incorporate a coupling by the common parts, and we deduce the second order equations of the canals, by approximating the Navier-Stokes projected on these currents $\overrightarrow{N_k}$. *A priori* this method does not support a coupling at the level of masses $m_k^j; j, k = 1, 2, 3$, in front of the second order derivatives in time, but it supports a coupling by a matrix $(c_k^j)_{j,k=1,2,3}$ of damping coefficients, in front of the first order derivatives in time. However we also included in Ariadne the computation with the $m_k^j; j, k = 1, 2, 3$.

Note that another kind of coupling could occur due to the Coriolis forces, because with these forces, the motion of all the cupulae would be present on the second member $F_k(t)$. However, following the arguments we gave, this can happen only for complex fluid motions and for large velocities, and in this highly non-linear case, it would be also difficult to justify the pendulum equations. In particular the consideration of $Q_k(t)$ would be not sufficient to describe the cupula deflection, an infinite number of parameters would *a priori* come into the play.

In fact, the exact coupling between the three equations representing the dynamics of the semi-circular canals, should also be analysed through a finite elements analysis of the Navier-Stokes and Reissner-Mindlin coupled equations. However, while algorithms required to perform such analyses are not particularly difficult to implement, running FEA analyses on fluid-structure interactions, under the assumption of incompressible fluid and using triangulated meshes obtained from natural objects, which generate tetrahedron that are highly subject to both shear locking and volume locking, is known

to give very unstable results and to require very good computational power to run at an acceptable speed. In particular, it seems hopeless at this moment to propose a stable automatic algorithm in Ariadne that would perform such analyses while insuring the user to get trustable results at the same time.

Ariadne, in its current form, thus appears to be the best compromise we can found between accuracy of results, speed of analysis and easiness of use. We hope that as it is, it combines elegantly practical experiments, technological discoveries, computational tools and theoretical justifications, without adding a too heavy material without strong necessity. This doesn't tell that Ariadne is a fixed process, to the contrary we also hope it will evolve to be more and more accurate and easy to use.

To finish, we insist on the remarkable reduction of dimensions realized by the labyrinth, to offer three scalar functions, representing a three dimensional rotation at the second order, starting from the very complicated movement of one fluid and three elastic membranes. This is comparable to the manner the three dimensions of colors are resulting from a complex spatial distribution of light frequencies. The way this reduction appears for the labyrinth is by using its *homology* in the mathematical sense, which is of dimension three, linking form with function.

Références

[1] V. J. Wilson, *Mammalian vestibular physiology.* Springer Science & Business Media, 2013.

[2] R. Rabbitt and E. Damiano, "A hydroelastic model of macromechanics in the endolymphatic vestibular canal," *Journal of Fluid Mechanics*, vol. 238, pp. 337–369, 1992.

[3] E. Damiano and R. Rabbitt, "A singular perturbation model of fluid dynamics in the vestibular semicircular canal and ampulla," *Journal of Fluid Mechanics*, vol. 307, no. 1, pp. 333–372, 1996.

[4] R. D. Rabbitt, "Directional coding of three-dimensional movements by the vestibular semicircular canals," *Biological cybernetics*, vol. 80, no. 6, pp. 417–431, 1999.

[5] C. M. Oman, E. N. Marcus, and I. S. Curthoys, "The influence of semicircular canal morphology on endolymph flow dynamics : An anatomically descriptive mathematical model," *Acta oto-laryngologica*, vol. 103, no. 1-2, pp. 1–13, 1987.

[6] R. D. Rabbitt, E. R. Damiano, and J. W. Grant, "Biomechanics of the semicircular canals and otolith organs," in *The vestibular system*, pp. 153–201, Springer, 2004.

[7] G. F. Duff and D. C. Spencer, "Harmonic tensors on riemannian manifolds with boundary," *Annals of Mathematics*, pp. 128–156, 1952.

[8] A. J. Chorin and J. E. Marsden, *A mathematical introduction to fluid mechanics*, vol. 4 of *Texts in Applied Mathematics*. Springer-Verlag, New York, third ed., 1993.

[9] P. G. Ciarlet, *Mathematical elasticity. Vol. II*, vol. 27 of *Studies in Mathematics and its Applications*. North-Holland Publishing Co., Amsterdam, 1997. Theory of plates.

[10] M. Murthy, "An Improved Transverse Shear Deformation Theory for Laminated Anisotropic Plates," *NASA Technical Paper*, 1981.

[11] F. Gruttmann and W. Wagner, "Shear correction factors in Timoshenko's beam theory for arbitrary shaped cross-sections," *Computational Mechanics*, vol. 27, pp. 199–207, 2001.

[12] S. Timoshenko, *Strength of Materials*. D. Van Nostrand Company, Inc New-York, 2nd edition ed., 1940.

[13] G. De Rham, *Differentiable Manifolds : Forms, Currents, Harmonic Forms*. Berlin Heidelberg New York Tokyo : Springer-Verlag, 1984.

[14] M. Muller and J. Verhagen, "A mathematical approach enabling the calculation of the total endolymph flow in the semicircular ducts," *Journal of theoretical biology*, vol. 134, no. 4, pp. 503–529, 1988.

[15] M. A. Ifediba, S. M. Rajguru, T. E. Hullar, and R. D. Rabbitt, "The role of 3-canal biomechanics in angular motion transduction by the human vestibular labyrinth," *Annals of biomedical engineering*, vol. 35, no. 7, pp. 1247–1263, 2007.

Ariadne Manual

Modules description

Version 1.1

Romain David, Alain Berthoz, Alexander Stoessel, Fred Spoor and Daniel Bennequin

Max Planck Institute for Evolutionary Anthropology, Leipzig, Germany
Laboratoire de Physiologie de la Perception et de l'Action, Paris, France
Museum National d'Histoire Naturelle, Paris, France
University College London, United Kingdom

July 5th, 2016

First published as Supplementary Information of David et al (submitted)[1]

About this document

The Ariadne Manual is part of the documentation of Ariadne Toolbox, and assumes a basic understanding of semicircular duct morphology and function. It presents step-by-step tutorials to help the user preparing raw input data, explains how to use each module of Ariadne Toolbox, and provides detailed information about algorithms and equations implemented in these modules, including structure and format of related files. Information presented in this manual corresponds to Ariadne Toolbox 1.0 for Windows NT platforms. Latest documentations and executables of Ariadne are available at:

http://www.earbank.org

Copyright information

Ariadne Toolbox is distributed free of charge if used for non-commercial purposes, and is provided without any warranty. To be fully functional, Ariadne needs the software packages Elmer, Gmsh and Scilab to be installed on the same computer. These can be downloaded without costs from their respective websites:

Elmer: https://www.csc.fi/web/elmer

Gmsh: http://geuz.org/gmsh/

Scilab: http://www.scilab.org/fr

Information about the software can be found in their respective manuals. Ariadne Toolbox is known to work with Gmsh 2.8.5, Elmer 8.0 and Scilab 5.4.0. It may also work with more recent versions but this has not been tested. Information and specifications given in this document are believed to be true and accurate at the time of writing, but reports of any errors and typos as well as general comments are welcome, and can be submitted at http://www.earbank.org.

Table of Contents

I. Overview of Ariadne Toolbox 7

i. Using Ariadne_SCDS 13

ii. MeshPreparation 21

1. FEAPreparation 21

a. Description 21
b. Algorithm 21
c. Input files 23
d. Output files 23

2. GMSHPreparation 24

a. Description 24
b. Instructions 24
c. Output files 25

3. GMSH 25

a. Description 25
b. Procedure 25
c. Input files 27
d. Output files 27

4. ElmerGrid 28

a. Description 28
b. Procedure 28
c. Input files 28
d. Output files 29

iii. MorphometryAuto 30

1. VolumeCalc 30

a. Description 30
b. Algorithm 30
c. Input Files 31
d. Output files 32

2. SurfaceCalc 32

a. Description 32

b. Algorithm 32
c. Input files 33
d. Output files 33
3. LineSetCreator 33
a. Description 33
b. Template 33
c. Input Files 35
d. Output files 35
4. MaximalResponseAxes 36
a. Description 36
b. Algorithm 36
c. Input files 39
d. Output files 39
5. DuctForm 40
a. Description 40
b. Algorithm 40
c. Input files 41
d. Output files 42
6. CupulaMorpho 42
a. Description 42
b. Algorithm 42
c. Input files 45
d. Output files 45
7. ShrinkCor 46
a. Description 46
b. Input parameters 46
c. Algorithm 48
d. Input files 54
e. Output files 54
8. SCDSReferenceFrame 55
a. Description 55
b. Algorithm 55
c. Input files 60

d. Output files 60
9. SCDSMorphometry 61
a. Description 61
b. Algorithm 61
c. Input files 62
d. Output files 62
iv. FEAAuto.......... 63
1. ElmerPreparation 63
a. Description 63
b. Input parameters 63
c. Instructions 64
d. Input files 66
e. Output files 67
2. ElmerSolver 67
a. Description 67
b. Procedure 68
c. Input files 70
d. Output files 71
v. BiomechanicsAuto 72
1. BiomechaParam 72
a. Description 72
b. Input parameters 73
c. Algorithm 74
d. Input Files 89
e. Output Files 89
2. BodePlotsGenerator 91
a. Description 91
b. Algorithm 91
c. Input Files 95
d. Output Files 95
3. SensitivityMapping 96
a. Description 96

b. Algorithm 96
c. Input files 104
d. Output files 104
vi. VestibularRefAuto 108
1. DataVestibularReference 108
a. Description 108
b. Algorithm 108
c. Input files 111
d. Output files 112
2. STLVestibularReference 114
a. Description 114
b. Algorithm 114
c. Input files 115
d. Output files 115
vii. Miscellaneous 116
1. DataToTable 116
a. Description 116
b. Algorithm 116
c. Abbreviations 116
d. Input files 120
e. Output files 120
References 121

I. Overview of Ariadne Toolbox

Ariadne Toolbox is a software package that currently consists of 19 executable files, 8 batch files and 2 Scilab scripts. Its main purpose is to analyze the morphometrics and biomechanics of the semicircular duct system. Ariadne Toolbox requires primary input files prepared as described in section I of the Ariadne Manual – Data preparation.

The modules of Ariadne Toolbox and their hierarchical structure are shown in Figure 1. To use Ariadne Toolbox, one can either (1) directly run the batch files **Ariadne_SCDS** or **Ariadne_SCDS_Lite**, (2) sequentially run the batch files **MeshPreparation**, **MorphometryAuto**, **FEAAuto**, **BiomechanicsAuto** and potentially **VestibularRefAuto**, or (3) sequentially run the executable files **FEAPreparation**, **GMSHPreparation**, **GMSH**, **ElmerGrid**, **VolumeCalc**, **SurfaceCalc**, **LineSetCreator**, **MaximalResponseAxes**, **DuctForm**, **CupulaMorpho**, **ShrinkCor**, **SCDSReferenceFrame**, **SCDSMorphometry**, **ElmerPreparation**, **ElmerSolver**, **BiomechaParam**, **BodePlotsGenerator**, **SensitivityMapping** and potentially **DataVestibularReference** and **STLVestibularReference**. We strongly recommend following options 1 or 2 as these are more user-friendly than option 3.

A step-by-step tutorial explaining how to use **Ariadne_SCDS** and **Ariadne_SCDS_Lite** can be found in section I.i. Detailed information concerning algorithms and procedures implemented by each software module of Ariadne is provided in subsequent sections. The general purpose of each of these modules can be summarized as follows:

1. **FEAPreparation** (section I.ii.1.) converts STL files of the middle cross-section of each cupula (section I.v. of the Ariadne Manual – Data preparation), from a three-dimensional space to a two dimensional space.

2. **GMSHPreparation** (section I.ii.2.) writes '.geo' instruction files for module **GMSH**.

3. **GMSH** (section I.ii.3.) is used in to build a FEA mesh of the middle cross-section of a cupula, and to identify and register the labels of boundaries that are important for subsequent modules.

4. **ElmerGrid** (section I.ii.4.) is used to convert FEA meshes, produced by **GMSH**, into **Elmer** mesh format, and to clean resulting meshes for finite element analysis.

5. **VolumeCalc** (section I.iii.1.) computes the volume of endolymph that is contained inside each relevant anatomical part of the semicircular duct system.

6. **SurfaceCalc** (section I.iii.2.) computes the surface area of the inner walls of each anatomical part of the semicircular ducts.

7. **LineSetCreator** (section I.iii.3.) structures the coordinates of the landmarks that define the central streamline of each relevant part of the semicircular duct system (section I.iii. of the Ariadne Manual – Data preparation) for **MaximalResponseAxes** to compute various morphological metrics (section I.iii.4.).

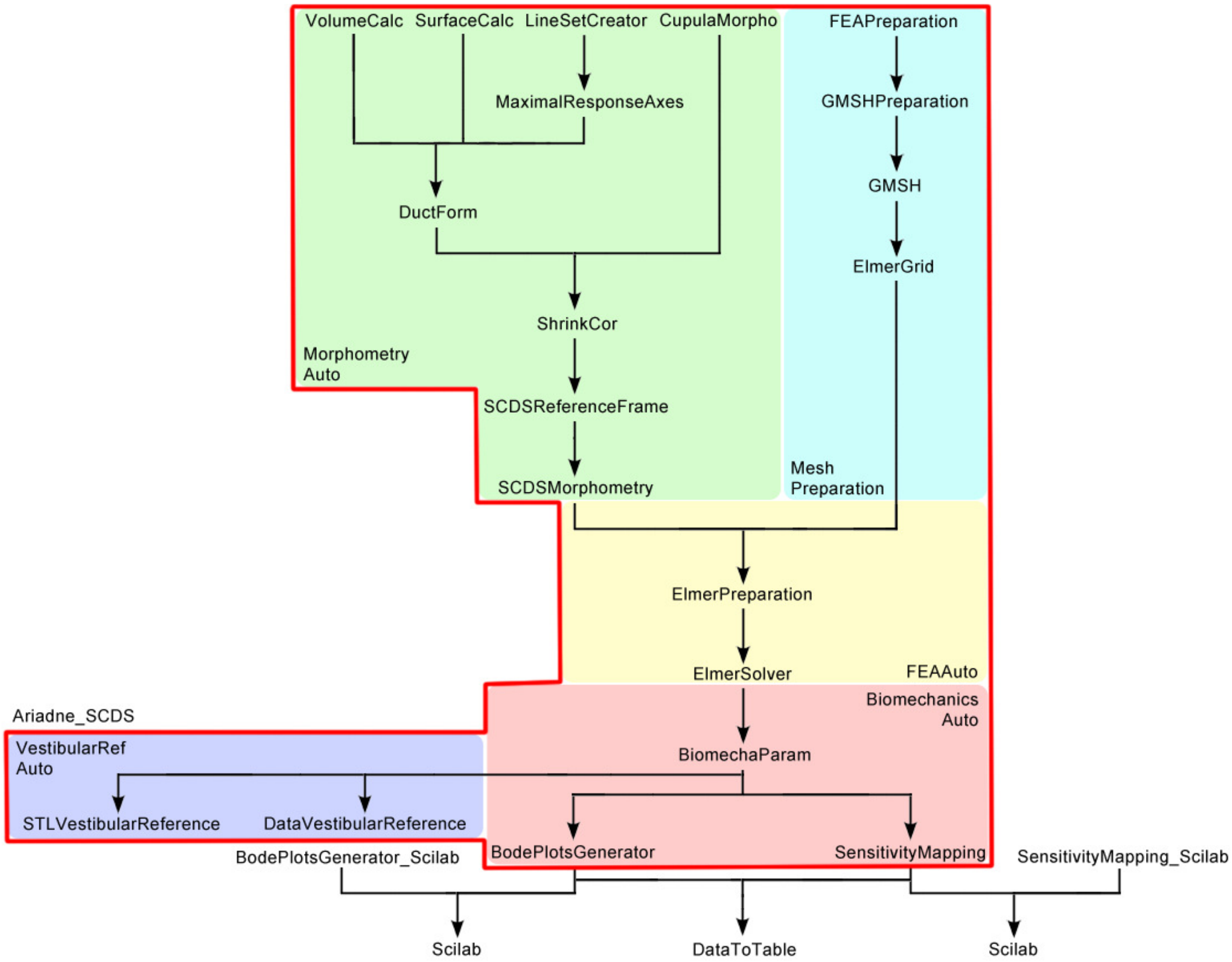

Figure 1: Hierarchical structure and dependencies of the batch and executable files of Ariadne Toolbox. The colors highlight which modules pertain to each batch process.

8. **MaximalResponseAxes** (section I.iii.4.) computes, based on a model of uncoupled ducts:

- the length of the central streamlines running inside each part of the semicircular duct system
- the maximal area enclosed by the central streamline of each semicircular duct
- the mean radius of curvature (R) of each semicircular duct
- the vector components of the maximal response plane of each semicircular duct, in the scan frame of reference
- the center of rotation of each semicircular duct, in the scan frame of reference
- the ipsilateral angular relationships of the maximal response planes of the semicircular ducts
- the variance of the ipsilateral angular relationships of the maximal response planes of the semicircular ducts from orthogonality (90var[2])
- the average deviation of the ipsilateral angular relationships of the maximal response planes of the semicircular ducts from orthogonality (90dev[3]).

MaximalResponseAxes also output 'lineset' files that can be opened in Avizo, and which graphically represent the excitation direction of the maximal response axis of each semicircular duct, following the right hand rule.

9. **DuctForm** (section I.iii.5.) computes:

- the mean cross-sectional area of the endolymphatic volume contained inside each part of the semicircular duct system
- the mean cross-sectional radius (r) of the endolymphatic volume contained inside the slender part of each semicircular duct
- ratios between the mean cross-sectional radii of the endolymphatic volumes contained inside each part of a semicircular duct and that of its respective slender part
- the mean wall shape drag factors of the cross-sections of the endolymphatic volume contained inside each part of the semicircular duct system.

10. **CupulaMorpho** (section I.iii.6.) computes:

- the mean cross-sectional areas of the cupulae
- the central cross-sectional areas of the cupulae
- the mean thickness of the cupulae, testing three different assumptions on stereocilia/kinocilia length
- the thickness of the cupulae according to Rabbitt's empirical law[4].

11. **ShrinkCor** (section I.iii.7.):

- computes shrinkage correction factors derived from empirical modelling of relative shrinkage inside the toadfish inner ear[5]
- computes resolution correction factors to adjust for potential errors caused by manual segmentation
- applies both factors to volumes, surfaces, lengths, areas, vector components and angles that were previously calculated by **VolumeCalc** (section I.iii.1.), **SurfaceCalc** (section I.iii.2.), **MaximalResponseAxes** (section I.iii.4.) and **DuctForm** (section I.iii.5.) and **CupulaMorpho** (section I.iii.6.).

12. **SCDSReferenceFrame** (section I.iii.8.) computes, based on a model of uncoupled ducts, and each time with three levels of correction for potential shrinkage and segmentation errors:

- the vector components of the maximal response plane of each semicircular duct, in the vestibular frame of reference[6]
- the center of rotation of each semicircular duct, in the vestibular frame of reference
- the synergistic angular relationships of the maximal response planes of the semicircular ducts
- the angular relationships of the maximal response planes of the semicircular ducts, with the three vestibular reference planes of the head
- the variance of the synergistic angular relationships of the maximal response planes of the semicircular ducts from planarity (180var)

- the average deviation of the synergistic angular relationships of the maximal response planes of the semicircular ducts from planarity (180dev)

SCDSReferenceFrame also output 'matrix' files that can be used by **DataVestibularReference** (section I.vi.1.) and **STLVestibularReference** (section I.vi.2.) to mirror data objects (linesets, landmarks, STLs) across the mid-sagittal plane of the head.

13. **SCDSMorphometry** (section I.iii.9.) compiles morphological information provided by the modules **VolumeCalc** (section I.iii.1.), **SurfaceCalc** (section I.iii.2.), **MaximalResponse Axes** (section I.iii.4.), **DuctForm** (section I.iii.5.), **CupulaMorpho** (section I.iii.6.), **ShrinkCor** (section I.iii.7.) and **SCDSReferenceFrame** (section I.iii.8.) into few summary files, which are much easier to read and to handle than files initially outputted by these modules.

14. **ElmerPreparation** (section I.iv.1.) writes '.sif' instruction files for the module **ElmerSolver** (section I.iv.2.).

15. **ElmerSolver** (section I.iv.2.) is used to:

- map the transverse displacement at the level of the middle cross section of each cupula, which follows from the application of a maintained homogeneous pressure on their surface
- analyze the deflection of areas of the cupula where stereocilia/kinocilia with various lengths can be found (section I.v. of the Ariadne Manual – Data preparation).

16. **BiomechaParam** (section I.v.1.) computes the viscosity of both the endolymph and the cupulae from body temperature, following a modified version of the empirical law of Ten Kate and Kuiper[7].

It also computes, based on a model of coupled ducts, each time with three levels of correction for potential shrinkage and segmentation errors, and with three different assumptions on stereocilia/kinocilia length, when applicable:

- the vector components of the maximal response plane of each semicircular duct, in the scan frame of reference (uncoupled values are also outputted),
- the center of rotation of each semicircular duct, in the scan frame of reference
- the vector components of the prime direction of each semicircular duct, in the vestibular frame of reference
- the ipsilateral angular relationships of the maximal response planes of the semicircular ducts
- the synergistic angular relationships of the maximal response planes of the semicircular ducts
- the angular relationships of the maximal response planes of the semicircular ducts, with the three vestibular reference planes of the head
- the variance of the ipsilateral angular relationships of the maximal response planes of the semicircular ducts from orthogonality (90var[2])
- the average deviation of the ipsilateral angular relationships of the maximal response planes of the semicircular ducts from orthogonality (90dev[3])

- the variance of the synergistic angular relationships of the maximal response planes of the semicircular ducts from planarity (180var)
- the average deviation of the synergistic angular relationships of the maximal response planes of the semicircular ducts from planarity (180dev).
- the displacement profile factor of each cupula
- the deflection of the stereocilia/kinocilia areas of each cupula
- the mass parameter of each part of the semicircular duct system
- the damping parameter of each part of the semicircular duct system
- the stiffness parameter of each cupula
- the inertial forcing parameter of each semicircular duct torus
- the long time constant of each semicircular duct (uncoupled values are also outputted)
- the short time constant of each semicircular duct (uncoupled values are also outputted)
- the lower corner frequency of each semicircular duct (uncoupled values are also outputted)
- the upper corner frequency of each semicircular duct (uncoupled values are also outputted)
- the natural frequency of each semicircular duct (only uncoupled values are outputted)
- the fractional bandwidth of the middle frequency range of each semicircular duct below and above their natural frequency (only uncoupled values are outputted)
- the in-plane mechanical sensitivity of each semicircular duct, relative to angular rotation (uncoupled values are also outputted)
- the in-plane mechanical velocity gain of each semicircular duct, relative to angular velocity (uncoupled values are also outputted)
- the in-plane mechanical acceleration gain of each semicircular duct, relative to angular acceleration).

The latter three parameters are expressed in units of cupula volumic displacement, cupula average linear displacement and cilia average deflection.

BiomechaParam also output 'matrix' files that can be used by **DataVestibularReference** (section I.vi.1.) and **STLVestibularReference** (section I.vi.2.) to place objects in the vestibular frame of reference, as well as 'lineset' files that can be opened in Avizo, and which graphically represent the excitation direction of the maximal response axis of each semicircular duct, following the right hand rule.

17. **BodePlotsGenerator** (section I.v.2.) generates data, based on a model of coupled ducts, that can be visualized in the form of bode plots using **SciLab** 5.4.0 (see tutorial in section I.i. and accompanying Fig.4). These bode plots describe the continuous variation of both mechanical phase and gain of each semicircular duct across the whole frequency bandwidth of head motion, relative to angular velocity. **BodePlotsGenerator** produce data for in-plane, for pitch, for roll and for yaw rotations, each time with three different assumptions on stereocilia/kinocilia length, with three levels of correction for potential shrinkage and segmentation errors, and expressed in units of cupula volumic displacement, cupula average linear displacement and cilia average deflection.

18. **SensitivityMapping** (section I.v.3.) generates data, based on a model of coupled ducts, which can be visualized in the form of Mercator projections using **SciLab** 5.4.0 (see tutorial in section I.i. and accompanying Fig.5). These maps describe, each time with three levels of correction for potential shrinkage and segmentation errors, three different assumptions on stereocilia/kinocilia length, two assumption on gain summation, and with up to three different sensitivity metrics, the spatial distribution of:

- the average mechanical gain
- the standard deviation of the mechanical gain
- the maximal mechanical gain
- the saturating mechanical gain
- the standardized mechanical gain
- the 'Hullar sensitivity[2]' of the semicircular duct system

covering the full directional range of head motion.

SensitivityMapping also outputs files summarizing these maps by providing statistical information including the global mean, the global standard deviation, the maximum and the minimum of the corresponding sensitivity values across the whole spatial distribution, as well as information about the specific sensitivity values that are retrieved for pitch, roll and yaw rotations.

19. **DataVestibularReference** (section I.vi.1.) uses transformation matrices outputted by **SCDSReferenceFrame** (section I.iii.8.) and **BiomechaParam** (section I.v.1.) in order to:

- reflect landmarks, linesets and STL files describing a semicircular duct system, across the mid-sagittal plane of the head (Fig.6c)
- transfer the complete set of landmarks, linesets and STL files from the scan frame of reference to the vestibular frame of reference[6], based on a model of coupled ducts.

20. **STLVestibularReference** (section I.vi.2.) transfers any STL file registered in the scan frame of reference to the vestibular frame of reference[6], based on a model of coupled ducts.

i. Using Ariadne_SCDS

Ariadne_SCDS is a batch file that automatically executes **MeshPreparation** (section I.ii.), **MorphometryAuto** (section I.iii.), **FEAAuto** (section I.iv.), **BiomechanicsAuto** (section I.v.), and **VestibularRefAuto** (section I.vi.), in that order. Please note that the lite version, called **Ariadne_SCDS_Lite**, automatically executes **MorphometryAuto**, **FEAAuto** and **BiomechanicsAuto**, in that order, with omission of both **MeshPreparation** and **VestibularRefAuto**.

Ariadne_SCDS is coded in such a way that it will seek most information needed by the Ariadne modules by reading a **TaxaInfo.txt** file that has to be placed at the root of the **Data** folder, and by looking into the name of files and folders contained in that same folder. It is then essential, to use this module, to strictly follow the naming convention of input files as described in Ariadne Manual – Data preparation and to structure the **Data** folder accordingly.

It should be noted that **Ariadne_SCDS** is not limited to analyze only one specimen at a time but can read in every specimen found in **TaxaInfo.txt**. This makes analysis of multiple specimens possible as long as naming conventions are rigorously followed.

Here we describe how to use **Ariadne_SCDS**, assuming the user carefully prepared all input data according to section I. of the Ariadne Manual – Data preparation. The procedure to use **Ariadne_SCDS_ Lite** is similar but steps 3.d. to 3.j. have to be skipped:

1. If step 1 has already been done go directly to step 2. Otherwise:
    a. Download Gmsh 2.8.5 at http://geuz.org/gmsh/ and install it.
    b. Place the Gmsh executable in the folder **/AriadneToolbox/Gmsh/** and call it **gmsh.exe**.
    c. Download Elmer 8.1 at https://www.csc.fi/web/elmer and install it.
    d. Click the [Start] button of Windows.
    e. In the Search textbox, type 'cmd'. The Windows Command Prompt should open.
    f. In the Command Prompt, type 'ElmerSolver'.
    g. Make sure ElmerSolver launched (do not give attention to the ELMERSOLVER_STARTINFO problem).
    h. In the Command Prompt, type 'ElmerGrid'.
    i. Make sure ElmerGrid launched. If neither ElmerSolver or/nor ElmerGrid launched reinstall Elmer 8.1 with administration rights.
    j. Download Scilab 5.4.0 at http://www.scilab.org/fr and install it.
2.
    a. Open the folder **/AriadneToolbox/Data/**.
    b. Double-click the file **TaxaInfo.txt**.
    c. Go to the end of the line "#Taxa_Name Units Temperature EC Resolution" and press the [Enter] key.
    d. On the new line, type in the same **SPECIESNAME** that was used for all files and folders related to the specimen of interest, as described in section I. of the Ariadne Manual – Data preparation.
    e. Press the [Spacebar] key.

f. Type in ‘**1**’ if input files are in micrometers, ‘**2**’ if they are in millimeters and ‘**3**’ if they are in centimeters.
g. Press the [Spacebar] key.
h. Type in the body temperature of the specimen in °C.
i. Press the [Spacebar] key.
j. Type in ‘**0**’ if the specimen lacks *eminentia cruciatae* and ‘**1**’ if they are present on the specimen (see section I.v. of the Ariadne Manual – Data preparation).
k. Press the [Spacebar] key.
l. Type in the scan resolution of the specimen in µm.
m. Press the [Enter] key.
n. Redo steps d-m for every specimens that can be found in the folder **/AriadneToolbox/Data**/.
o. Add the ‘**#**’ symbol just before the **SPECIESNAME** of specimens that should not be analyzed.

3.

a. Open the folder **/AriadneToolbox/Batch/**.
b. Double-click the file **Ariadne_SCDS.bat**.
c. When prompted “Please enter the segmentation error factor in voxel units:” enter the factor you want to use to take account of potential manual segmentation errors. We obtained an empirical value of 0.24 for this parameter by performing an inter-observer study[1].
d. When prompted “Please enter the level of the shrinkage correction. Between 0 (no correction) and 1 (full correction)” enter the factor you want to use to take account of potential shrinkage effect. Choosing “0” means that the data won’t be corrected for shrinkage; whereas choosing “1” means that the data will likely be overcorrected for shrinkage.
e. To the question “You assume that the cupula is…”, type in ‘**1**’ if the cupula is considered to be clamped at the level of both the *crista ampullaris* and the walls of the ampulla, type in ‘**2**’ if the cupula is considered to be clamped at the level of the *crista ampullaris* only and supported at the level of the walls of the ampulla, and type in ‘**3**’ if the cupula is considered to be clamped at the level of the *crista ampullaris* only and loose at the level of the walls of the ampulla.
f. Press the [Enter] key.
g. When asked “Press any key to continue…”, press the [Enter] key.
h. When the GMSH window pop-up, the sentence “The label of the crista floor boundary is…” should be written in the console. Look for the corresponding boundary on the mesh appearing in the GMSH window and type the correct label number in the console, then press the [Enter] key (Fig.2).
i. Now the sentence “The label of the ampulla walls boundary is…” should appears in the console. Look for the corresponding boundary on the mesh appearing in the GMSH window and type the correct label number in the console, then press the [Enter] key (Fig.2).
j. Redo steps f-g for all other cupulae popping-up in GMSH windows.

k. In the ElmerGrid log, check that neither the boundaries nor the bodies changed indexes, and then press the [Enter] key. Indexes that changed are not the same before and after the arrows (example 1 -> 2).
l. Redo step i for all other ElmerGrid logs.
m. Press the [Enter] key when computation is done.

Note: It can sometimes happen that error messages pop up during the **GMSH** part and/or the **ElmerSolver** part. This is linked to the inability of current algorithms implemented into **GMSH** to triangulate meshes from STL files containing very poor triangles (high aspect ratios, intersections). If that happens, redo the preparation of the FEA model of the problematic cupula(e) by making sure that all steps presented in section I.v. of the Ariadne Manual – Data preparation, particularly the steps 7-13, were rigorously followed. Increasing the number of triangles in step 19 of section I.v. of the Ariadne Manual – Data preparation can also potentially help.

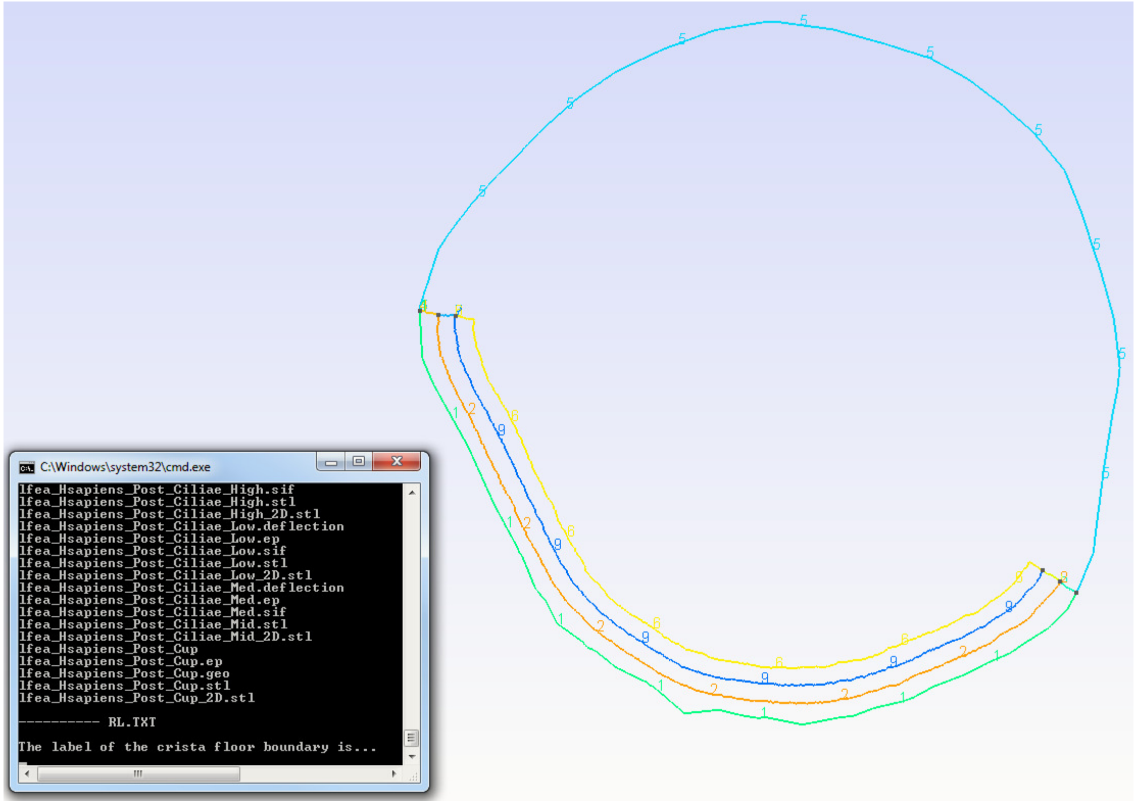

Figure 2: Illustration of the interactive procedure included in the process of the MeshPreparation batch file. On the bottom-left, the console prompts the user about the label of "the crista floor boundary". On the right, in GMSH, the boundaries are shown with different colors and labels. Here, the crista floor boundary is colored in light green and is labelled 1. The user then has to type '1' into the console to answer the question. The other boundary that is important during this interactive procedure, the ampulla walls boundary, is here colored in light blue and labelled 5.

Application of this protocol will lead to the creation of numerous output files located in several folders of **/AriadneToolbox/Data/SPECIESNAME/**:

21. At the root of **/AriadneToolbox/Data/SPECIESNAME/**, three '.morph' files (section I.iii.9.), six '.biomecha' files (section I.v.1.) and six '.sensitivity' files (section I.v.3.) can be found, eventually with an additional '.errorlog' file if problems occurred during the computation. All these files can be opened with the traditional windows' notepad but we recommend using Notepad++ instead.

22. The folder **/AriadneToolbox/Data/SPECIESNAME/VestRef/** contains all the files that were initially placed in the folder **/AriadneToolbox/Data/SPECIESNAME/ScanRef/** except they now are transposed to the vestibular frame of reference, along with mirrored version relative to the mid-sagittal plane of the head (section I.vi.). STL files can be found in the sub folders **/Other/**, **/Surfaces/** and **/Volumes/** and can be opened using any mesh visualization software (including Avizo 7.1). Landmark files can be found in the sub folder **/Landmarks/**, whereas Lineset files (section I.iii.4.) can be found in the folder **/MaxAxes/**. These files are formatted to be opened with Avizo 7.1.

23. The folder **/AriadneToolbox/Data/SPECIESNAME/FEA/** contains a lot of new files, most of which being only interesting for the computation process itself. However '.ep' files, which correspond to deformation patterns at the level of the middle cross-section of the cupula following a maintained homogeneous pressure of 0.05Pa at its surface, can be visualized (Fig.3). To open an '.ep' file:

1. To visualize how each element of the cupula is transversally displaced after stimulation:
    a. In the folder **/Elmer7/bin/**, double-click **ElmerPost.exe**.
    b. In the GUI ribbon, click [File>Open…].
    c. Click [Browse…], move to the folder **/AriadneToolbox/Data/ SPECIESNAME/FEA/**, select the '.ep' file to visualize, click [Open], then [OK].
    d. In Elmer-Post textbox type: 'math tmp = size(Deflection.1)' then press the [Enter] key.
    e. In Elmer-Post textbox type: 'math n = tmp(1)' then press the [Enter] key.
    f. In Elmer-Post textbox type: 'math on = nodes' then press the [Enter] key.
    g. In Elmer-Post textbox type: 'math nodes(0,0:n-1) = (on(0,0:n-1)+((-Deflection.2) *Deflection.1))' then press the [Enter] key.
    h. In Elmer-Post textbox type: 'math nodes(1,0:n-1) = (on(1,0:n-1)+((-Deflection.3) *Deflection.1))' then press the [Enter] key.
    i. In Elmer-Post textbox type: 'math nodes(2,0:n-1) = 10*(on(2,0:n-1)+Deflection.1)' then press the [Enter] key. This way the deflection will be exaggerated 10 times.
    j. Click [Color Mesh].
    k. In [Mesh Style], select [Surface]
    l. In [Color Variable], select [Deflection.1], click [OK], then [Apply].
    m. Click [Color Scale].
    n. In [Color Variable], select [Deflection.1], click [OK], then [Apply].

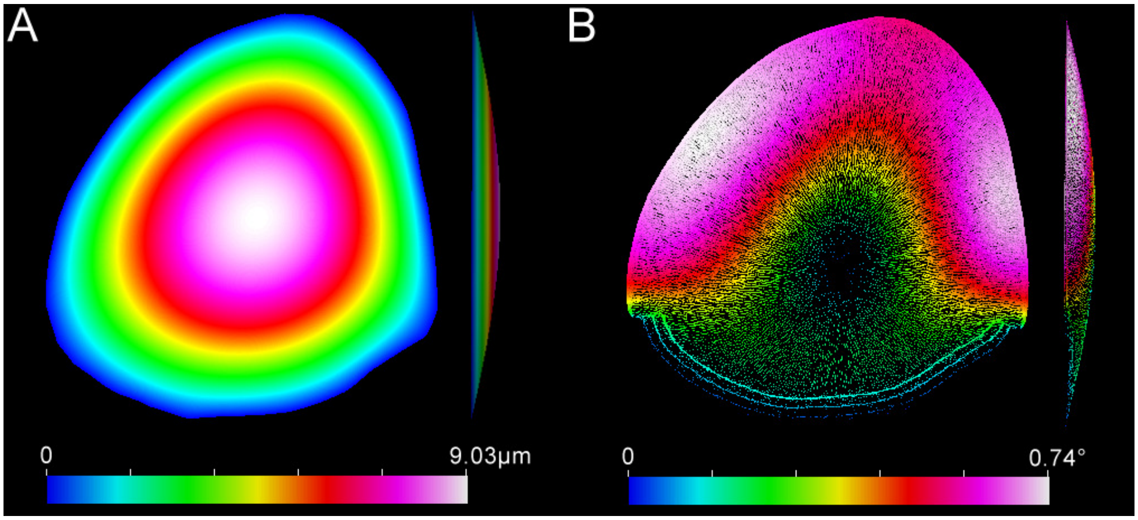

Figure 3: Frontal and lateral views of transverse displacement (A) and rotation (B) patterns of the plate elements constituting the middle cross-section of the cupula that follow the application of a maintained homogeneous pressure of 0.05Pa at its surface. Visual deformation has been exaggerated ten times.

2. To visualize how each element of the cupula is rotated after stimulation:
    a. Click [Color Mesh] to make the mesh disappear.
    b. In Elmer-Post textbox type: 'math Rotation = 0', then press the [Enter] key.
    c. In Elmer-Post textbox type: 'math Rotation(0,0:n-1) = Deflection.2', then press the [Enter] key.
    d. In Elmer-Post textbox type: 'math Rotation(1,0:n-1) = Deflection.3', then press the [Enter] key.
    e. In Elmer-Post textbox type: 'math Rotation(2,0:n-1) = 0', then press the [Enter] key.
    f. In Elmer-Post textbox type: 'math AbsRotation = sqrt(vdot(Rotation,Rotation))', then press the [Enter] key.
    g. Click [Vectors].
    h. In [Color Variable], select [AbsRotation] and then click [OK].
    i. In [Length Variable], select [AbsRotation], click [OK].
    j. In [Arrow Variable], select [Rotation], click [OK] and then [Apply].
    k. Click [Color Scale].
    l. In [Color Variable], select [AbsRotation], click [OK] and then [Apply].

24. The folder **/AriadneToolbox/Data/SPECIESNAME/BodePlots/** contains 9 '.bode' files that can be visualized using Scilab 5.4.0. (Fig.4). To do so:

1.
    a. In the folder **/AriadneToolbox/Scilab_Scripts/**, open **BodePlotsVisualizer_Scilab.txt** using Notepad ++ (right-click, Edit with Notepad++).
    b. In the [Species =] field, write the **SPECIESNAME** between quotation marks.
    c. In the [Measure =] field, select the metrics to be used to express semicircular duct gain. 'Cilia' corresponds to the average deflection of areas of the cupulae where stereocilia/kinocilia can be found. 'Cupula' corresponds to the average transverse

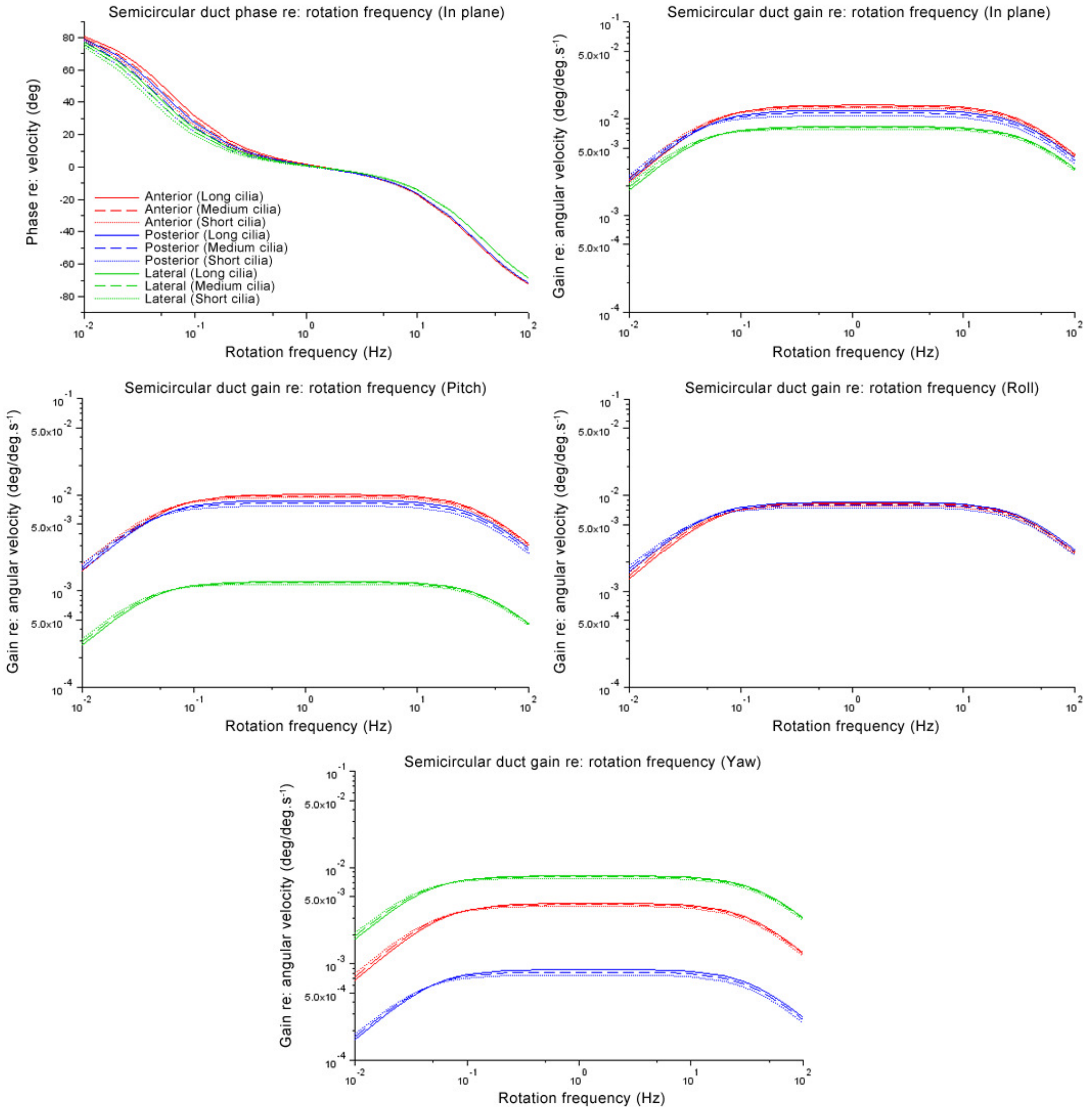

Figure 4: Overview of bode plots computed using "BodePlotsVisualizer_Scilab.txt" script and illustrating how biomechanical phase and gain of the human semicircular duct system are related to head motion frequency. Results depicted here consider the average deflection of areas of the cupulae where stereocilia/kinocilia can be found as the metrics for semicircular duct gain.

displacement across the cupula. 'Volume' corresponds to the volume displacement of the cupula.

d. In the [Data =] field, select the level of correction to be used. 'Cor' corresponds to shrinkage correction only. 'MinusCor' corresponds to shrinkage correction plus a contraction of the segmented labyrinth. 'PlusCor' corresponds to shrinkage correction plus a dilation of the segmented labyrinth.
e. Click anywhere on the script, press the [Ctrl+A] keys, then the [Ctrl+C] keys.

2.

a. Launch Scilab 5.4.0.
b. In the GUI ribbon, click [File>Change current directory…].

c. Select **/AriadneToolbox/Data/** then click [Open].
d. Click anywhere on the Scilab console, press the [Ctrl+V] keys, then the [Enter] key

25. The folder **/AriadneToolbox/Data/SPECIESNAME/SensitivityMap/** contains 594 directional sensitivity maps that can be visualized using Scilab 5.4.0. (Fig.5). To do so:

1.
   a. In the folder **/AriadneToolbox/Scilab_Scripts/**, open **SensitivityMapping_Scilab.txt** using Notepad ++ (right-click, Edit with Notepad++).
   b. In the [Species =] field, write the **SPECIESNAME** in parenthesis.
   c. In the [Cilia =] field, choose the stereocilia/kinocilia length to be considered. 'Short' corresponds to a length of 30µm, 'Medium' corresponds to a length of 60µm and 'Long' corresponds to a length of 90µm.
   d. In the [Stimulus =] field, choose between 'Velocity' and 'Acceleration'.
   e. In the [Measure =] field, select the metrics to be used to express semicircular duct gain. 'Cilia' corresponds to the average deflection of areas of the cupulae where stereocilia/kinocilia can be found. 'Cupula' corresponds to the average transverse displacement across the cupula. 'Volume' corresponds to the volume displacement of the cupula.
   f. In the [Canals =] field, select '_Synergistic' if synergistic semicircular duct pairs are to be considered as functional units in spatial sensitivity calculations. Leave the field blank if each semicircular duct is to be considered independently in spatial sensitivity calculations.
   g. In the [Data =] field, select the level of correction to be used. 'Cor' corresponds to shrinkage correction only. 'MinusCor' corresponds to shrinkage correction plus a contraction of the segmented labyrinth. 'PlusCor' corresponds to shrinkage correction plus a dilation of the segmented labyrinth.
   h. In the [MaxGain =] field, select 'SatGain' if heterogeneously weighted inhibitory and activatory rotations are to be considered, according to their effect on saturation of nervous activity. Select 'MaxGain' if inhibitory and activatory rotations are to be homogeneously weighted instead.
   i. In the [Hullar =] field, select 'HullarResponse' if the result of Hullar's Bubble script is to be visualized. Leave the field blank if this sub-process is not needed.
   j. Click anywhere on the script, press the [Ctrl+A] keys, then the [Ctrl+C] keys.
2.
   a. Launch Scilab 5.4.0.
   b. In the GUI ribbon, click [File>Change current directory…].
   c. Select **/AriadneToolbox/Data/** then click [Open].
   d. Click anywhere on the Scilab console, press the [Ctrl+V] keys, then the [Enter] key.

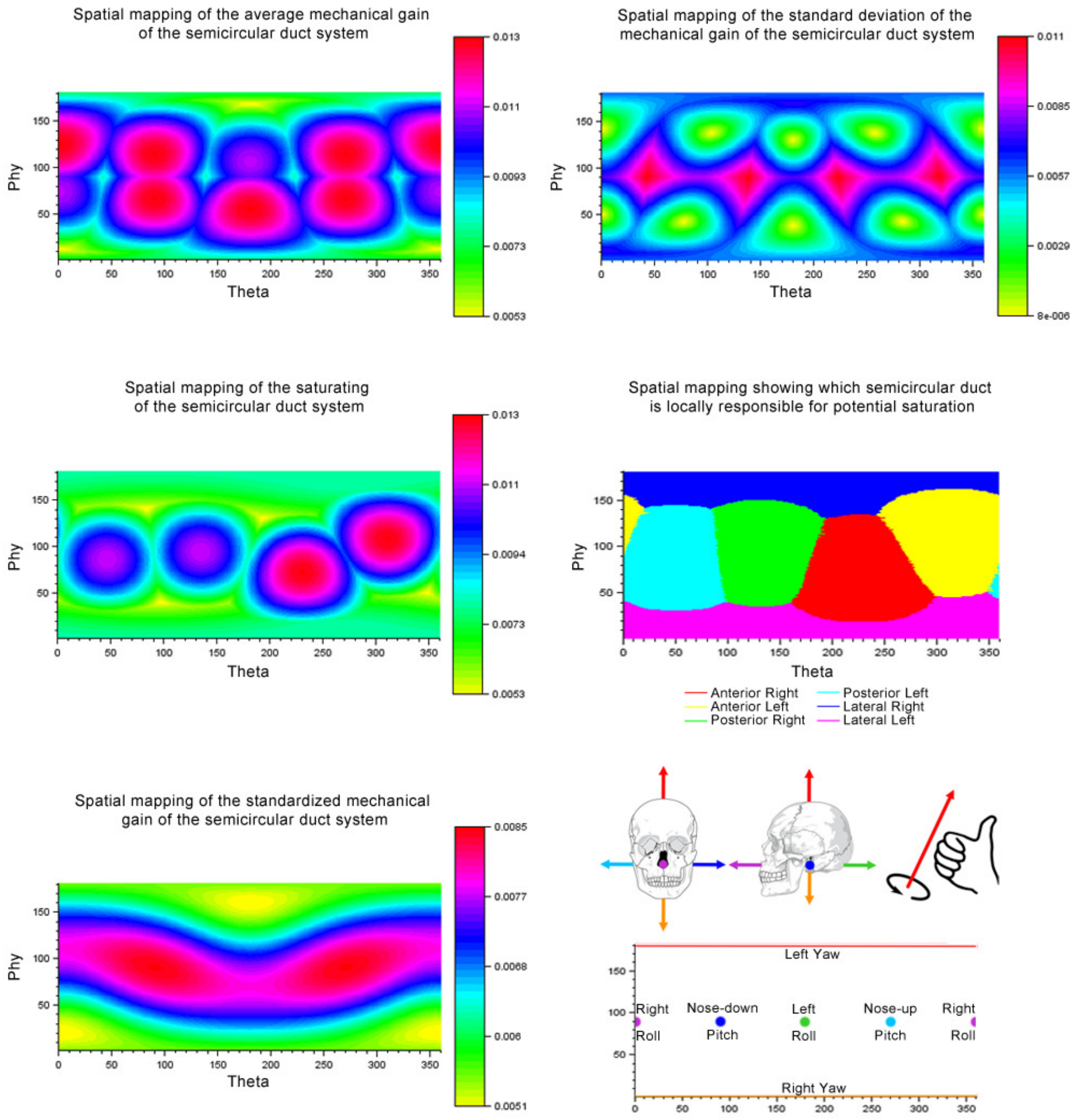

Figure 5: Overview of spatial sensitivity maps computed using "SensitivityMapping_Scilab.txt" script and illustrating how various metrics of the sensitivity of the semicircular duct system vary relative to axis and direction of rotations. Results depicted here consider the average deflection of areas of the cupulae where stereocilia/kinocilia can be found as the metrics for semicircular duct sensitivity. The bottom right panel shows were to look on the spatial sensitivity maps for the sensitivity of the semicircular duct system to a particular head rotation. Note that Phy has been reversed compared to the computation process (see section I.vi.3.) such that left yaw rotation corresponds to Phy = 180 in visualizations and to Phy = 0 during computation.

ii. MeshPreparation

MeshPreparation is a batch file that automatically executes **FEAPreparation** (section I.ii.1.), **GMSHPreparation** (section I.ii.2.), **GMSH** (section I.ii.3.) and **ElmerGrid** (section I.ii.4.), in that order. To use it, double-click it, in the folder **/AriadneToolbox/Batch/**, and to follow steps 3 e-k from section I.i.

MeshPreparation is coded in such a way that it will seek most information needed by its modules by reading a **TaxaInfo.txt** file that has to be placed at the root of the **Data** folder (see section I.i., step (2), and by looking into the name of files and folders contained in that same folder. It is then essential, to use this module, to strictly follow the naming convention for input files as described in Ariadne Manual – Data preparation, for files pertaining to the **FEAPreparation**, **GMSHPreparation**, **GMSH** and **ElmerGrid** modules, and to structure the **Data** folder accordingly.

It should be noted that **MeshPreparation** is not limited to analyze only one specimen at a time but can read in every specimen found in **TaxaInfo.txt**. This makes analysis of multiple specimens possible as long as naming conventions are rigorously followed.

1. FEAPreparation

a. Description

FEAPreparation is an executable, written in C, which converts STL files of the middle cross-section of each cupula (section I.v. of the Ariadne Manual – Data preparation), from a three-dimensional space to a two dimensional space. Transferred files can be opened in **GMSH** (section I.ii.3.) to build FEA meshes that are compatible with the two-dimensional **SmitcSolver** of **ElmerSolver** (section I.v.2.).

FEAPreparation can either be used as stand-alone software by double-clicking on **FEAPreparation.exe**, in the folder **/AriadneToolbox/**, and following instructions appearing in the console, or as part of the batch processes **Ariadne_SCDS.bat** or **MeshPreparation.bat** (sections I.i & I.ii.).

b. Algorithm

In order to transfer STL files of the split middle cross-section of each cupula from an orthonormal basis of three dimensions $B=\left\{\vec{x},\vec{y},\vec{z}\right\}$ to an orthonormal basis of two dimensions $B'=\left\{\vec{X},\vec{Y}\right\}$, **FEAPreparation** first reads-in the three-dimensional coordinates $\left\{i_n, j_n, k_n\right\}$ of each vertex of the first triangle of the bulk part of a given cupula. Then, it applies the formula:

$$\vec{Z}=\frac{\left(\vec{p}_{b,1,2}-\vec{p}_{b,1,1}\right)\times\left(\vec{p}_{b,1,3}-\vec{p}_{b,1,1}\right)}{\left\|\left(\vec{p}_{b,1,2}-\vec{p}_{b,1,1}\right)\times\left(\vec{p}_{b,1,3}-\vec{p}_{b,1,1}\right)\right\|}=\begin{pmatrix}Z_x\\Z_y\\Z_z\end{pmatrix}$$

where $\vec{Z}$ corresponds to the unit vector that is perpendicular to the plane of the middle cross-section of the given cupula; and where $\vec{p}_{b,1,1}$, $\vec{p}_{b,1,2}$ and $\vec{p}_{b,1,3}$ respectively correspond to vectors running from the origin of B to the first, second and third vertices of the first triangle of the bulk part of the middle cross-section of the given cupula.

After that, it applies the formulae:

$$\vec{Y} = \frac{\vec{Z} \times \vec{U}}{\left\| \vec{Z} \times \vec{U} \right\|} = \begin{pmatrix} Y_x \\ Y_y \\ Y_z \end{pmatrix}$$

and

$$\vec{X} = \frac{\vec{Y} \times \vec{Z}}{\left\| \vec{Y} \times \vec{Z} \right\|} = \begin{pmatrix} X_x \\ X_y \\ X_z \end{pmatrix}$$

where

$$\vec{U} = \begin{pmatrix} \frac{1}{\sqrt{3}} \\ \frac{1}{\sqrt{3}} \\ \frac{1}{\sqrt{3}} \end{pmatrix}$$

and where $\vec{X}$ and $\vec{Y}$ form an orthonormal basis of two dimensions $B' = \{\vec{X}, \vec{Y}\}$, which is coplanar with the middle cross-section of the given cupula, and to which STL files will be transferred.

To do so, **FEAPreparation** applies the following formula to each vertex p_n found in STL files of the middle cross-section of the given cupula:

$$p_n^{'} = P_{B'}^{B} p_n$$

where

$$P_{B'}^{B} = \frac{1}{\det \begin{bmatrix} X_x & Y_x & Z_x \\ X_y & Y_y & Z_y \\ X_z & Y_z & Z_z \end{bmatrix}} \begin{bmatrix} X_x & X_y & X_z \\ Y_x & Y_y & Y_z \end{bmatrix}$$

corresponds to the passage matrix from B to B'; and where

$$p_n = \begin{pmatrix} i_n \\ j_n \\ k_n \end{pmatrix}$$

and

$$p_n^{'} = \begin{pmatrix} i_n^{'} \\ j_n^{'} \end{pmatrix}$$

respectively correspond to each vertex of the STL files of the split middle cross-section of the given cupula, as registered in the orthonormal bases B and B'.

Finally, **FEAPreparation** creates new STLs of the split middle cross-section of the given cupula by registering the newly computed coordinates $\left\{i_n^{'}, \mathrm{j}_n^{'}, 0\right\}$ of all transferred vertices in new files.

c. Input files

As input, **FEAPreparation** needs the files:

rfea_SPECIESNAME_Ant_Cup.stl

rfea_SPECIESNAME_Ant_Cilia_Short.stl

rfea_SPECIESNAME_Ant_Cilia_Medium.stl

rfea_SPECIESNAME_Ant_Cilia_Long.stl

rfea_SPECIESNAME_Post_Cup.stl

rfea_SPECIESNAME_Post_Cilia_Short.stl

rfea_SPECIESNAME_Post_Cilia_Medium.stl

rfea_SPECIESNAME_Post_Cilia_Long.stl

rfea_SPECIESNAME_Lat_Cup.stl

rfea_SPECIESNAME_Lat_Cilia_Short.stl

rfea_SPECIESNAME_Lat_Cilia_Medium.stl

rfea_SPECIESNAME_Lat_Cilia_Long.stl

with '**lfea_**' instead of '**rfea_**' for a left labyrinth, to be placed in the folder **/AriadneToolbox/Data/SPECIESNAME/FEA/** (see section I.v. of the Ariadne Manual – Data preparation).

d. Output files

FEAPreparation outputs the files:

rfea_SPECIESNAME_Ant_Cup_2D.stl

rfea_SPECIESNAME_Ant_Cilia_Short_2D.stl

rfea_SPECIESNAME_Ant_Cilia_Medium_2D.stl

rfea_SPECIESNAME_Ant_Cilia_Long_2D.stl

rfea_SPECIESNAME_Post_Cup_2D.stl

rfea_SPECIESNAME_Post_Cilia_Short_2D.stl

rfea_SPECIESNAME_Post_Cilia_Medium_2D.stl

rfea_SPECIESNAME_Post_Cilia_Long_2D.stl

rfea_SPECIESNAME_Lat_Cup_2D.stl

rfea_SPECIESNAME_Lat_Cilia_Short_2D.stl

rfea_SPECIESNAME_Lat_Cilia_Medium_2D.stl

rfea_SPECIESNAME_Lat_Cilia_Long_2D.stl

in the folder **/AriadneToolbox/Data/SPECIESNAME/FEA/**.

2D.stl files correspond to the STL that compose the split middle cross-section of each cupula, but now registered in the new two-dimensional coordinate system.

2. GMSHPreparation

a. Description

GMSHPreparation is an executable, written in C, which writes '.geo' instruction files for module **GMSH**. These files order **GMSH** to build FEA meshes of the middle cross-section of studied cupulae, and to highlight the labels of the boundaries of these FEA meshes, on the visualisation screen of **GMSH** (section I.ii.3.).

GMSHPreparation can either be used as stand-alone software by double-clicking on **GMSHPreparation.exe**, in the folder **/AriadneToolbox/**, and following instructions appearing in the console, or as part of the batch processes **Ariadne_SCDS.bat** or **MeshPreparation.bat** (sections I.i & I.ii.).

b. Instructions

GMSHPreparation writes following instructions in corresponding .geo file:

Mesh.Algorithm = 6; // 2D mesh algorithm (1=MeshAdapt, 2=Automatic, 5=Delaunay, 6=Frontal, 7=bamg, 8=delquad)
Mesh.SaveAll = 0; // Ignore Physical definitions and save all elements
Mesh.LabelSampling = 250; // Frequency of labels display
Mesh.LabelType = 1; // Type of label displayed (0=element number, 1=elementary entity number, 2=physical entity number, 3=partition number, 4=coordinates)
Mesh.Lines = 1; // Display lines
Mesh.LineNumbers = 1; // Display lines numbers
Mesh.LineWidth = 2; // Widths of displayed lines
Mesh.Prisms = 0; // Display Prisms
Mesh.Pyramids = 0; // Display Pyramids
Mesh.Quadrangles = 0; // Display Quadrangles
Mesh.SurfaceEdges = 0; // Display Surface Edges
Mesh.Tetrahedra = 0; // Display Tetrahedra
Mesh.Triangles = 0; // Display Triangles
Mesh.VolumeEdges = 0; // Display Volume Edges

Merge "**xfea_SPECIESNAME_Duct**_Cilia_Short_2D.stl";
Merge "**xfea_SPECIESNAME_Duct**_Cup_2D.stl";
Merge "**xfea_SPECIESNAME_Duct**_Cilia_Long_2D.stl";
Merge "**xfea_SPECIESNAME_Duct**_Cilia_Medium_2D.stl";

CreateTopology;

Mesh 2;

Save "**xfea_SPECIESNAME_Duct**_Cup.msh";

where **xfea** is replaced by **rfea** for a right labyrinth and **lfea** for a left labyrinth, where **SPECIESNAME** is replaced by the name used for the studied specimen in every other sections, and where **Duct** is either replaced by **Ant**, **Post** or **Lat** depending on which cupula is studied.

These instructions allow quality FEA meshes of the cupula to be built in **GMSH**, thanks to the use of the Frontal meshing algorithm (see GMSH documentation for more information). They also allow the

boundaries of each body of the cupula to be clearly visible and labelled on the visualization screen of **GMSH**, which proves to be very useful to identify both crista and ampulla wall boundaries and to accordingly fill the .boundaries file that is needed for the finite element analysis (section I.ii.3.).

c. Output files

GMSHPreparation outputs the files:

rfea_SPECIESNAME_Ant_Cup.geo **rfea_SPECIESNAME_Lat_Cup.geo**

rfea_SPECIESNAME_Post_Cup.geo

in the folder **/AriadneToolbox/Data/SPECIESNAME/FEA/**.

.geo files contain instructions for **GMSH** (section I.ii.3.) to build FEA meshes of the cupula from STL files outputted by **FEAPreparation** (section I.ii.1.).

3. GMSH

a. Description

GMSH is a software, developed by Christophe Geuzaine and Jean-François Remacle, which has not been developed as part of Ariadne Toolbox, but which is nevertheless needed for it to be fully operational. **GMSH** can be downloaded on the website http://geuz.org/gmsh/, and its executable **gmsh.exe** needs to be placed in the folder **/AriadneToolbox/Gmsh/**. As stated in the above mentioned website, **GMSH** "is a 3D finite element grid generator with a build-in CAD engine and post-processor, whose design goal is to provide a fast, light and user-friendly meshing tool with parametric input and advanced visualization capabilities". Inside Ariadne framework, **GMSH** is used in to build a FEA mesh of the middle cross-section of a cupula, and to identify and register the labels of boundaries that are important for subsequent modules.

GMSH can either be used as stand-alone software by double-clicking on **gmsh.exe**, in the folder **/AriadneToolbox/Gmsh/**, and following the procedure described below, or as part of the batch processes **Ariadne_SCDS.bat** or **MeshPreparation.bat** (sections I.i & I.ii.).

b. Procedure

1.
   a. Open the folder **/AriadneToolbox/Data/SPECIESNAME/FEA/**.
   b. Right-click anywhere and select [New > Text Document].
   c. Name the file **rfea_SPECIESNAME_boundaries.labels**.
   d. Open **rfea_SPECIESNAME_boundaries.labels** with any notepad software.

2.
   a. Open the folder **/AriadneToolbox/Gmsh/**.
   b. Double-click **gmsh.exe**.
   c. Click [File>Open].

d. Select the file **rfea_SPECIESNAME_Ant_Cup.geo**, in the folder **/Ariadne Toolbox/Data/SPECIESNAME/FEA/** and click [Open].
e. In **rfea_SPECIESNAME_boundaries.labels**, on the first line, type in the label that correspond to the boundary of the floor of the *crista ampullaris* according to what can be seen in **GMSH** visualization window (label '1' in Fig.2).
f. Press the [Spacebar] key.
g. In **rfea_SPECIESNAME_boundaries.labels**, type in the label that correspond to the boundary of the walls of the ampulla according to what can be seen in **GMSH** visualization window (label '5' in Fig.2).
h. Press the [Spacebar] key.
i. Close **GMSH**.

3.
    a. Double-click **gmsh.exe**.
    b. Click [File>Open].
    c. Select the file **rfea_SPECIESNAME_Post_Cup.geo**, in the folder **/Ariadne Toolbox/Data/SPECIESNAME/FEA/** and click [Open].
    d. In **rfea_SPECIESNAME_boundaries.labels**, type in the label that correspond to the boundary of the floor of the *crista ampullaris* according to according to what can be seen in **GMSH** visualization window.
    e. Press the [Spacebar] key.
    f. In **rfea_SPECIESNAME_boundaries.labels**, type in the label that correspond to the boundary of the walls of the ampulla according to according to what can be seen in **GMSH** visualization window.
    g. Press the [Spacebar] key.
    h. Close **GMSH**.

4.
    a. Double-click **gmsh.exe**.
    b. Click [File>Open].
    c. Select the file **rfea_SPECIESNAME_Lat_Cup.geo**, in the folder **/Ariadne Toolbox/Data/SPECIESNAME/FEA/** and click [Open].
    d. In **rfea_SPECIESNAME_boundaries.labels**, type in the label that correspond to the boundary of the floor of the *crista ampullaris* according to according to what can be seen in **GMSH** visualization window.
    e. Press the [Spacebar] key.
    f. In **rfea_SPECIESNAME_boundaries.labels**, type in the label that correspond to the boundary of the walls of the ampulla according to according to what can be seen in **GMSH** visualization window.
    g. Close **GMSH**.
    h. Save the file **rfea_SPECIESNAME_boundaries.labels** in the folder **/Ariadne Toolbox/Data/SPECIESNAME/FEA/**.

This full procedure allows getting good quality meshes of the middle cross-section of a cupula, which are suited for finite element analysis (section I.iv.2.). It also provides **ElmerPreparation** with important files containing information about boundaries labels, which are vital for the finite element analysis to perform well (section I.iv.1.).

Note: It can sometimes happen that error messages pop up during the **GMSH** procedure. This is linked to the inability of current algorithms implemented into **GMSH** to triangulate meshes from STL files containing very poor triangles (high aspect ratios, intersections). If that happens, redo the preparation of the FEA model of the problematic cupula(e) by making sure that all steps presented in section I.v. of the Ariadne Manual – Data preparation, particularly the steps 7-13, were rigorously followed. Increasing the number of triangles in step 17 of section I.v. of the Ariadne Manual – Data preparation can also potentially help.

c. Input files

As input, **GMSH** needs the files:

rfea_SPECIESNAME_Ant_Cup_2D.stl

rfea_SPECIESNAME_Ant_Cilia_Short_2D.stl

rfea_SPECIESNAME_Ant_Cilia_Medium_2D.stl

rfea_SPECIESNAME_Ant_Cilia_Long_2D.stl

rfea_SPECIESNAME_Post_Cup_2D.stl

rfea_SPECIESNAME_Post_Cilia_Short_2D.stl

rfea_SPECIESNAME_Post_Cilia_Medium_2D.stl

rfea_SPECIESNAME_Post_Cilia_Long_2D.stl

rfea_SPECIESNAME_Lat_Cup_2D.stl

rfea_SPECIESNAME_Lat_Cilia_Short_2D.stl

rfea_SPECIESNAME_Lat_Cilia_Medium_2D.stl

rfea_SPECIESNAME_Lat_Cilia_Long_2D.stl

rfea_SPECIESNAME_Ant_Cup.geo

rfea_SPECIESNAME_Post_Cup.geo

rfea_SPECIESNAME_Lat_Cup.geo

to be placed in the folder **/AriadneToolbox/Data/SPECIESNAME/FEA/** (see sections I.ii.1 & I.ii.2.). Note that these input files will be suppressed when **GMSH** is done (except if it was used as stand-alone).

d. Output files

GMSH procedure leads to output the files:

rfea_SPECIESNAME_Ant_Cup.msh

rfea_SPECIESNAME_Post_Cup.msh

rfea_SPECIESNAME_Lat_Cup.msh

rfea_SPECIESNAME_boundaries.labels

in the folder **/AriadneToolbox/Data/SPECIESNAME/FEA/**.

.**labels** files contain information about boundaries numbering that will be used by **ElmerPreparation** (section I.iv.1.) in building instruction files for **ElmerSolver** procedure (section I.iv.2.).

.**msh** files correspond to FEA meshes of the middle cross-section of the cupulae, which will be further processed by **ElmerGrid** (section I.ii.4.) to make them compatible for analysis with **ElmerSolver** (section I.iv.2.).

4. ElmerGrid

a. Description

ElmerGrid is a software, developed by CSC – IT Center for Science (CSC), which has not been developed as part of Ariadne Toolbox, but which is nevertheless needed for it to be fully operational. **ElmerGrid** can be downloaded as part of **Elmer FEM** on the website https://www.csc.fi/web/elmer. As stated in the above mentioned website, **ElmerGrid** "is a simple mesh generator and mesh manipulation utility that can also read meshes generated by other mesh generators and manipulate them". Inside Ariadne framework, **ElmerGrid** is used to convert FEA meshes, produced by **GMSH**, into **Elmer** mesh format, and to clean resulting meshes for finite element analysis.

ElmerGrid can either be used as stand-alone software by following the procedure described below, or as part of the batch processes **Ariadne_SCDS.bat** or **MeshPreparation.bat** (sections I.i & I.ii.).

b. Procedure

1.
    a. Click the [Start] button of Windows.
    b. In the Search textbox, type 'cmd'. The Windows Command Prompt should open.
    c. In the Command Prompt, type 'cd user_computer_specific_path\AriadneToolbox\Data\SPECIESNAME\FEA'.
    d. In the Command Prompt, type 'ElmerGrid 14 2 rfea_SPECIESNAME_Ant_Cup.msh -autoclean -centralize -decimals 12'.
    e. In the Command Prompt, type 'ElmerGrid 14 2 rfea_SPECIESNAME_Post_Cup.msh -autoclean -centralize -decimals 12'.
    f. In the Command Prompt, type 'ElmerGrid 14 2 rfea_SPECIESNAME_Lat_Cup.msh -autoclean -centralize -decimals 12'.
    g. For each **ElmerGrid** log, check that neither the boundaries nor the bodies changed indexes. Indexes that changed are not the same before and after the arrows (example 1 -> 2).

This full procedure allows converting **GMSH** mesh format into **Elmer** mesh format, which is more suited for finite element analysis with **ElmerSolver** (section I.iv.2.). It also allows further cleaning, by remeshing bad triangles, and centering the meshes by moving them to the origin of the coordinate system. This last step is needed to prevent some errors linked to decimal limitations in **Elmer**.

c. Input files

As input, **ElmerGrid** needs the files:

rfea_SPECIESNAME_Ant_Cup.msh

rfea_SPECIESNAME_Lat_Cup.msh

rfea_SPECIESNAME_Post_Cup.msh

to be placed in the folder **/AriadneToolbox/Data/SPECIESNAME/FEA/** (see section I.ii.3.). Note that these input files are suppressed when **ElmerGrid** work is done (except if it was used as a stand-alone).

d. Output files

ElmerGrid outputs the folders:

rfea_SPECIESNAME_Ant_Cup

rfea_SPECIESNAME_Lat_Cup

rfea_SPECIESNAME_Post_Cup

in the folder **/AriadneToolbox/Data/SPECIESNAME/FEA/**.

Each of these folders contains the files:

mesh.boundary

mesh.header

mesh.elments

mesh.nodes

which contain information on FEA meshes of the cupulae, in a format readable by **ElmerSolver** (section I.iv.2., see Elmer documentation for more information).

iii. MorphometryAuto

MorphometryAuto is a batch file that automatically executes **VolumeCalc** (section I.iii.1.), **SurfaceCalc** (section I.iii.2.), **LineSetCreator** (section I.iii.3.), **MaximalResponseAxes** (section I.iii.4.), **DuctForm** (section I.iii.5.), **CupulaMorpho** (section I.iii.6.), **ShrinkCor** (section I.iii.7.), **SCDSReferenceFrame** (section I.iii.8.) and **SCDSMorphometry** (section I.iii.9.), in that order. To use it, double-click it, in the folder **/AriadneToolbox/Batch/**, and press any key.

MorphometryAuto is coded in such a way that it will seek most information needed by its modules by reading a **TaxaInfo.txt** file that has to be placed at the root of the **Data** folder (see section I.i., step (2), and by looking into the name of files and folders that are contained in that same folder. It is then essential, to use this module, to strictly follow the naming convention for input files as described in Ariadne Manual – Data preparation, for files pertaining to the **VolumeCalc**, **SurfaceCalc**, **LineSetCreator**, **MaximalResponseAxes**, **DuctForm**, **CupulaMorpho**, **ShrinkCor**, **SCDSReferenceFrame** and **SCDSMorphometry** modules, and to structure the **Data** folder accordingly.

It should be noted that **MorphometryAuto** is not limited to analyze only one specimen at a time but can read in every specimen found in **TaxaInfo.txt**. This makes analysis of multiple specimens possible as long as naming conventions are rigorously followed.

1. VolumeCalc

a. Description

VolumeCalc is an executable, written in C, which computes the volume of endolymph that is contained inside each relevant anatomical part of the semicircular duct system.

VolumeCalc can either be used as stand-alone software by double-clicking on **VolumeCalc.exe**, in the folder **/AriadneToolbox/**, and following instructions appearing in the console, or as part of the batch processes **Ariadne_SCDS.bat**, **Ariadne_SCDS_Lite.bat** or **MorphometryAuto.bat** (sections I.i & I.iii.).

b. Algorithm

VolumeCalc implements the divergence theorem in order to compute the volume of the 11 inputted STL files (sections I.i. & I.ii. of the Ariadne Manual – Data preparation). It also controls for effects smoothing, triangle reduction and splitting of the semicircular duct system may have induced on volume computation. To do so, it reads-in the coordinates of the vertices of each STL file and applies the following formulae:

$$V_n = \frac{1}{6}\sum_{i=1}^{N_{Vn}} \vec{p}_{Vn,i,1} \cdot \left(\vec{p}_{Vn,i,2} \times \vec{p}_{Vn,i,3} - \vec{p}_{Vn,i,2} \times \vec{p}_{Vn,i,1} - \vec{p}_{Vn,i,1} \times \vec{p}_{Vn,i,3} \right)$$

,

$$V_{tot} = \sum_{n=1}^{11} V_n$$

,

$$S_e = 100\frac{V_{SCDS} - V_{control}}{V_{control}}$$

and

$$D_e = 100\frac{V_{tot} - V_{SCDS}}{V_{SCDS}}$$

where V_n corresponds to the volume of the part $_n$ of the semicircular duct system; V_{tot} corresponds to the summation of computed volumes of endolymph contained inside each part of the semicircular duct system; $V_{control}$ corresponds to the volume of the semicircular duct system, as initially segmented (section I.i. of the Ariadne Manual – Data preparation); and V_{SCDS} corresponds to the volume of the semicircular duct system, after smoothing and triangle reduction (section I.ii. of the Ariadne Manual – Data preparation); where S_e corresponds to the effect smoothing and triangle reduction have on volume computation; and where D_e corresponds to the effect splitting the semicircular duct system has on volume computation; where $\vec{p}_{Vn,i,1}$, $\vec{p}_{Vn,i,2}$ and $\vec{p}_{Vn,i,3}$ respectively correspond to vectors running from the origin to the first, second and third vertices of the i-th triangle of the mesh of the part $_n$ of the semicircular duct system; and where N_{Vn} corresponds to the number of triangles contained in the mesh of the part $_n$ of the semicircular duct system.

At the end of the procedure, **VolumeCalc** output one file (two if both right and left labyrinths are studied) containing information on all computed volume metrics described above.

c. Input Files

As input, **VolumeCalc** needs the files:

rv_SPECIESNAME_Sa.stl

rv_SPECIESNAME_Sp.stl

rv_SPECIESNAME_Sl.stl

rv_SPECIESNAME_CC.stl

rv_SPECIESNAME_Aa.stl

rv_SPECIESNAME_Ap.stl

rv_SPECIESNAME_Al.stl

rv_SPECIESNAME_Ua.stl

rv_SPECIESNAME_Up.stl

rv_SPECIESNAME_SC.stl

rv_SPECIESNAME_Uc.stl

rv_SPECIESNAME_Control.stl

with '**lv_**' instead of '**rv_**' for a left labyrinth, to be placed in the folder **/AriadneToolbox/Data/SPECIESNAME/ScanRef/Volumes/** (see sections I.i & I.ii. of the Ariadne Manual – Data preparation).

It also needs the file:

r_SPECIESNAME_SCDS.stl

with '**_l_**' instead of '**_r_**' for a left labyrinth, to be placed in the folder **/AriadneToolbox/Data/SPECIESNAME/ScanRef/Other/** (see section I.ii. of the Ariadne Manual – Data preparation).

d. Output files

VolumeCalc outputs the file:

SPECIESNAME_R_Raw.volume

in the folder **/AriadneToolbox/Data/SPECIESNAME/**, with '**_L_**' instead of '**_R_**' for a left labyrinth.

.**volume** files contain information about the volume of endolymph contained inside the semicircular duct system, as well as inside each of its parts. They also contain information about the effects smoothing, triangle reduction and splitting of the semicircular duct system may have induced on volume computation.

2. SurfaceCalc

a. Description

SurfaceCalc is an executable, written in C, which computes the surface area of the inner walls of each anatomical part of the semicircular ducts.

SurfaceCalc can either be used as stand-alone software by double-clicking on **SurfaceCalc.exe**, in the folder **/AriadneToolbox/**, and following instructions appearing in the console, or as part of the batch processes **Ariadne_SCDS.bat**, **Ariadne_SCDS_Lite.bat** or **MorphometryAuto.bat** (sections I.i & I.iii.)

b. Algorithm

SurfaceCalc uses properties of the cross product in order to compute surface areas of the inner walls of the inputted STL files (section I.ii. of the Ariadne Manual – Data preparation). To do so, it reads-in the coordinates of the vertices of each STL file and applies the following formulae:

$$S_{n,k} = \frac{1}{2}\sum_{i=1}^{N_{Sn,k}} \left\| \left(\vec{p}_{Sn,k,i,2} - \vec{p}_{Sn,k,i,1}\right) \times \left(\vec{p}_{Sn,k,i,3} - \vec{p}_{Sn,k,i,1}\right) \right\|$$

where $S_{n,k}$ corresponds to the surface area of the inner walls of the part $_n$ of the semicircular duct $_k$; where $\vec{p}_{Sn,k,i,1}$, $\vec{p}_{Sn,k,i,2}$ and $\vec{p}_{Sn,k,i,3}$ respectively correspond to vectors running from origin to the first, second and third vertices of the i-th triangle of the mesh of the inner walls of the part $_n$ of the semicircular duct $_k$; and where $N_{Sn,k}$ corresponds to the number of triangles contained in the inner walls of the part $_n$ of the semicircular duct $_k$.

At the end of the procedure, **SurfaceCalc** output one file (two if both right and left labyrinths are studied) containing information on all computed surface area metrics described above.

c. Input files

As input, **SurfaceCalc** needs the files:

rs_SPECIESNAME_Sa.stl

rs_SPECIESNAME_Sp.stl

rs_SPECIESNAME_Sl.stl

rs_SPECIESNAME_CCa.stl

rs_SPECIESNAME_CCp.stl

rs_SPECIESNAME_Aa.stl

rs_SPECIESNAME_Ap.stl

rs_SPECIESNAME_Al.stl

rs_SPECIESNAME_UCa.stl

rs_SPECIESNAME_UCp.stl

rs_SPECIESNAME_UCl.stl

rs_SPECIESNAME_SC.stl

with '**ls_**' instead of **'rs_'** for a left labyrinth, to be placed in the folder **/AriadneToolbox/Data/SPECIESNAME/ScanRef/Surfaces/** (see section I.ii. of the Ariadne Manual – Data preparation).

d. Output files

SurfaceCalc outputs the file:

SPECIESNAME_R_Raw.surface

in the folder **/AriadneToolbox/Data/SPECIESNAME/**.

.surface files contain information about the surface area of the inner walls of the membranes that make up each part of the semicircular duct system.

3. LineSetCreator

a. Description

LineSetCreator is an executable, written in C, which structures the coordinates of the landmarks that define the central streamline of each relevant part of the semicircular duct system (section I.iii. of the Ariadne Manual – Data preparation) for **MaximalResponseAxes** to compute various morphological metrics (section I.iii.4.).

LineSetCreator can either be used as stand-alone software by double-clicking on **LineSetCreator.exe**, in the folder **/AriadneToolbox/**, and following instructions appearing in the console, or as part of the batch processes **Ariadne_SCDS.bat**, **Ariadne_SCDS_Lite.bat** or **MorphometryAuto.bat** (sections I.i & I.iii.)

b. Template

LineSetCreator job is to provide **MaximalResponseAxes** with structured information about the coordinates of each landmark making up the central streamlines of the semicircular duct system, as well as with information about the interconnections existing between the streamlines. To do so, **LineSetCreator** first reads-in all landmark files describing the central streamlines (section I.iii. of the

Ariadne Manual – Data preparation), and then rewrite their three-dimensional coordinates in a ‘lineset’ file, which follow this template:

ADT
Uc_{x1} Uc_{y1} Uc_{z1}
Ua_{x1} Ua_{y1} Ua_{z1}
⋮
Ua_{xn} Ua_{yn} Ua_{zn}
Aa_{x1} Aa_{y1} Aa_{z1}
⋮
Aa_{xn} Aa_{yn} Aa_{zn}
Sa_{x1} Sa_{y1} Sa_{z1}
⋮
Sa_{xn} Sa_{yn} Sa_{zn}
CC_{x1} CC_{y1} CC_{z1}
⋮
CC_{xn} CC_{yn} CC_{zn}
Uc_{x1} Uc_{y1} Uc_{z1}
PDT
Uc_{x1} Uc_{y1} Uc_{z1}
Up_{x1} Up_{y1} Up_{z1}
⋮
Up_{xn} Up_{yn} Up_{zn}
Ap_{x1} Ap_{y1} Ap_{z1}
⋮
Ap_{xn} Ap_{yn} Ap_{zn}
Sp_{x1} Sp_{y1} Sp_{z1}
⋮
Sp_{xn} Sp_{yn} Sp_{zn}
CC_{x1} CC_{y1} CC_{z1}
⋮
CC_{xn} CC_{yn} CC_{zn}
Uc_{x1} Uc_{y1} Uc_{z1}
LDT
Uc_{x1} Uc_{y1} Uc_{z1}
Ua_{x1} Ua_{y1} Ua_{z1}
⋮
Ua_{xn} Ua_{yn} Ua_{zn}
Al_{x1} Al_{y1} Al_{z1}
⋮
Al_{xn} Al_{yn} Al_{zn}
Sl_{x1} Sl_{y1} Sl_{z1}
⋮
Sl_{xn} Sl_{yn} Sl_{zn}
SC_{x1} SC_{y1} SC_{z1}
⋮
SC_{xn} SC_{yn} SC_{zn}
Uc_{x1} Uc_{y1} Uc_{z1}
Sa
Sa_{x1} Sa_{y1} Sa_{z1}
⋮
Sa_{xn} Sa_{yn} Sa_{zn}
Sp
Sp_{x1} Sp_{y1} Sp_{z1}
⋮
Sp_{xn} Sp_{yn} Sp_{zn}
Sl
Sl_{x1} Sl_{y1} Sl_{z1}
⋮
Sl_{xn} Sl_{yn} Sl_{zn}
CCa
Sa_{xn} Sa_{yn} Sa_{zn}
CC_{x1} CC_{y1} CC_{z1}
⋮
CC_{xn} CC_{yn} CC_{zn}
CCp
Sp_{xn} Sp_{yn} Sp_{zn}
CC_{x1} CC_{y1} CC_{z1}
⋮
CC_{xn} CC_{yn} CC_{zn}
CC
CC_{x1} CC_{y1} CC_{z1}
⋮
CC_{xn} CC_{yn} CC_{zn}
Uc_{x1} Uc_{y1} Uc_{z1}
SC
Sl_{xn} Sl_{yn} Sl_{zn}
SC_{x1} SC_{y1} SC_{z1}
⋮
SC_{xn} SC_{yn} SC_{zn}
UCa
CC_{xn} CC_{yn} CC_{zn}
Uc_{x1} Uc_{y1} Uc_{z1}
Ua_{x1} Ua_{y1} Ua_{z1}
⋮
Ua_{xn} Ua_{yn} Ua_{zn}
Aa_{x1} Aa_{y1} Aa_{z1}
UCp
CC_{xn} CC_{yn} CC_{zn}
Uc_{x1} Uc_{y1} Uc_{z1}
Up_{x1} Up_{y1} Up_{z1}
⋮
Up_{xn} Up_{yn} Up_{zn}
Ap_{x1} Ap_{y1} Ap_{z1}
UCl
SC_{xn} SC_{yn} SC_{zn}
Uc_{x1} Uc_{y1} Uc_{z1}
Ua_{x1} Ua_{y1} Ua_{z1}
⋮
Ua_{xn} Ua_{yn} Ua_{zn}
Al_{x1} Al_{y1} Al_{z1}
UC
Uc_{x1} Uc_{y1} Uc_{z1}
Ua_{x1} Ua_{y1} Ua_{z1}
⋮
Ua_{xn} Ua_{yn} Ua_{zn}
Aa
Aa_{x1} Aa_{y1} Aa_{z1}
⋮
Aa_{xn} Aa_{yn} Aa_{zn}
Sa_{x1} Sa_{y1} Sa_{z1}
Ap
Ap_{x1} Ap_{y1} Ap_{z1}
⋮
Ap_{xn} Ap_{yn} Ap_{zn}
Sp_{x1} Sp_{y1} Sp_{z1}
Al
Al_{x1} Al_{y1} Al_{z1}
⋮
Al_{xn} Al_{yn} Al_{zn}
Sl_{x1} Sl_{y1} Sl_{z1}

where landmark coordinates in dark red correspond to the central streamline of the slender part of the anterior semicircular duct; landmark coordinates in red correspond to the central streamline of the slender part of the posterior semicircular duct; landmark coordinates in orange correspond to the central streamline of the slender part of the lateral semicircular duct; landmark coordinates in light purple correspond to the central streamline of the common crus; landmark coordinates in purple correspond to the central streamline of the simple crus; landmark coordinates in blue correspond to the central streamline of the anterior utricle; landmark coordinates in dark blue

correspond to the central streamline of the posterior utricle; landmark coordinates in brown correspond to the central streamline of the common utricle; landmark coordinates in light green correspond to the central streamline of the anterior ampulla; landmark coordinates in green correspond to the central streamline of the posterior ampulla and landmark coordinates in light blue correspond to the central streamline of the lateral ampulla.

Note that the index $_1$ indicates the first landmark of the central streamline, whereas the index $_n$ indicates the last one.

c. Input Files

As input, **LineSetCreator** needs the files:

rcs_SPECIESNAME_Sa.landmarkAscii

rcs_SPECIESNAME_Sp.landmarkAscii

rcs_SPECIESNAME_Sl.landmarkAscii

rcs_SPECIESNAME_CC.landmarkAscii

rcs_SPECIESNAME_Aa.landmarkAscii

rcs_SPECIESNAME_Ap.landmarkAscii

rcs_SPECIESNAME_Al.landmarkAscii

rcs_SPECIESNAME_Ua.landmarkAscii

rcs_SPECIESNAME_Up.landmarkAscii

rcs_SPECIESNAME_SC.landmarkAscii

rcs_SPECIESNAME_Uc.landmarkAscii

with '**lcs_**' instead of '**rcs_**' for a left labyrinth, to be placed in the folder **/AriadneToolbox/Data/SPECIESNAME/ScanRef/Landmarks/** (see section I.iii. of the Ariadne Manual – Data preparation).

LineSetCreator has been coded to natively accept Avizo 6 and 7 landmark files saved in Ascii format. If the user can't access neither of these softwares, one option to obtain the lineset file consists of opening the example **.landmarkAscii** files, which can be found in the example folder **/AriadneToolbox/Data/SPECIESNAME/ScanRef/Landmarks/**, in filling the sections pertaining to the coordinates and to the total number of landmarks of the given central streamline, and then in saving the resulting file by following the correct naming conventions (see section I.iii. of the Ariadne Manual – Data preparation). This process needs to be reiterated for every central streamline of the upper part of the membranous labyrinth.

The other option consists of opening the file **Lineset_Template.lineset**, which can be found in the example folder **/AriadneToolbox/Data/SPECIESNAME/**, and to directly fill the lineset file with streamline coordinates by using the template presented in section I.iii.3.b. as guidance.

d. Output files

LineSetCreator outputs the file:

SPECIESNAME_R.lineset

in the folder **/AriadneToolbox/Data/SPECIESNAME/**.

.lineset files contain coordinates of landmarks defining the central streamline of each part making up the semicircular duct system, structured in a suitable way for **MaximalResponseAxes** to compute various morphological metrics (section I.iii.4.).

4. MaximalResponseAxes

a. Description

MaximalResponseAxes is an executable, written in C, which computes, based on a model of uncoupled ducts:

- the length of the central streamlines running inside each part of the semicircular duct system
- the maximal area enclosed by the central streamline of each semicircular duct
- the mean radius of curvature (R) of each semicircular duct
- the vector components of the maximal response plane of each semicircular duct, in the scan frame of reference
- the center of rotation of each semicircular duct, in the scan frame of reference
- the ipsilateral angular relationships of the maximal response planes of the semicircular ducts
- the variance of the ipsilateral angular relationships of the maximal response planes of the semicircular ducts from orthogonality (90var[2])
- the average deviation of the ipsilateral angular relationships of the maximal response planes of the semicircular ducts from orthogonality (90dev[3])

MaximalResponseAxes also output 'lineset' files that can be opened in Avizo, and which graphically represent the excitation direction of the maximal response axis of each semicircular duct, following the right hand rule.

MaximalResponseAxes can either be used as stand-alone software by double-clicking on **MaximalResponseAxes.exe**, in the folder **/AriadneToolbox/**, and following instructions appearing in the console, or as part of the batch processes **Ariadne_SCDS.bat**, **Ariadne_SCDS_Lite.bat** or **MorphometryAuto.bat** (sections I.i & I.iii.)

b. Algorithm

In order to compute all above mentioned metrics, **MaximalResponseAxes** first reads-in the lineset file containing information about coordinates and connections of all central streamlines contained inside the upper part of the membranous labyrinth (section I.iii.3.). Then it applies the following formulae, in order to retrieve the length of each central streamline:

$$L_{n,k} = \sum_{i=1}^{N_{n,k}} \left\| \vec{r}_{n,k,i+1} - \vec{r}_{n,k,i} \right\|$$

and

$$L_k = \sum_{n=1}^{n_k} L_{n,k}$$

where $L_{n,k}$ corresponds to the length of the central streamline of the part $_n$ of the semicircular duct $_k$; and L_k corresponds to the length of the central streamline of the semicircular duct $_k$; where $N_{n,k}$ corresponds to the number of landmarks contained in the central streamline of the part $_n$ of the semicircular duct $_k$; where $\vec{r}_{n,k,i}$ corresponds to a vector running from the origin to the i-th landmark of the central streamline of the part $_n$ of the semicircular duct $_k$; and where n_k corresponds to the number of parts contained in the semicircular duct $_k$.

Enclosed areas and scaled maximal response axes are then computed applying the following formulae, derived from[4,6,8,9]:

$$\vec{\Lambda}_k = \frac{1}{2}\sum_{i=1}^{N_k}\left\|\vec{r}_{k,i+1} - \vec{r}_{k,i}\right\| \cdot \left(\frac{\left(\vec{r}_{k,i} + \vec{r}_{k,i+1}\right)}{2} \times \left(\frac{\vec{r}_{k,i+1} - \vec{r}_{k,i}}{\left\|\vec{r}_{k,i+1} - \vec{r}_{k,i}\right\|}\right)\right)$$

and

$$\Lambda_k = \left\|\vec{\Lambda}_k\right\|$$

where Λ_k corresponds to the area enclosed by the central streamline of the semicircular duct $_k$, as projected on its maximal response plane; where N_k corresponds to the number of landmarks contained in the central streamline the semicircular duct $_k$; and where $\vec{r}_{k,i}$ corresponds to a vector running from the origin to the i-th landmark of the central streamline of the semicircular duct $_k$.

Then, from these parameters, both radii of curvature and semicircular duct planes are computed by applying the formulae:

$$R_k = \frac{L_k + 2\sqrt{\pi\Lambda_k}}{4\pi}$$

and

$$\vec{u}_k = \frac{\vec{\Lambda}_k}{\left\|\vec{\Lambda}_k\right\|}$$

where R_k corresponds to the mean radius of curvature the semicircular duct $_k$; and where $\vec{u}_k$ corresponds to the unit vector that is normal to the maximal response plane of the semicircular duct $_k$ and that is oriented along its excitation direction following the right hand rule.

Next, the center of rotation of each semicircular duct is computed, applying the following formula:

$$\vec{C}_k = \frac{1}{L_k}\sum_{i=1}^{N_k}\frac{\left\|\vec{r}_{k,i+1} - \vec{r}_{k,i}\right\| \cdot \left(\vec{r}_{k,i} + \vec{r}_{k,i+1}\right)}{2}$$

where $\vec{C}_n$ corresponds to a vector running from the origin to the center of rotation of the semicircular duct $_k$.

From these results, 'lineset' files, which graphically represent the excitation direction of the maximal response axis of each semicircular duct, are created by writing the coordinates of both the center of rotation of each semicircular duct as well as the vector component of their maximal response plane, in Avizo Line Set format.

Afterward, **MaximalResponseAxes** computes the ipsilateral angular relationships of the semicircular duct planes, following conventions introduced by Blanks et al.[10], by applying the formulae:

$$\vec{A}_n \wedge \vec{P}_n = arc\cos(-\vec{A}_n \cdot -\vec{P}_n)$$

and

$$\vec{A}_n \wedge \vec{L}_n = arc\cos(-\vec{A}_n \cdot \vec{L}_n)$$

and

$$\vec{L}_n \wedge \vec{P}_n = arc\cos(\vec{L}_n \cdot \vec{P}_n)$$

where $\vec{A}_n \wedge \vec{P}_n$, $\vec{A}_n \wedge \vec{L}_n$ and $\vec{L}_n \wedge \vec{P}_n$ correspond to the angles between maximal response planes of the right (or left) anterior and posterior semicircular ducts, anterior and lateral semicircular ducts and lateral and posterior semicircular ducts, respectively; and where $\vec{A}_n$, $\vec{P}_n$ and $\vec{L}_n$ correspond to unit vectors that are normal to the maximal response planes of the right (or left) anterior, posterior and lateral semicircular ducts respectively, and oriented along their excitation direction.

Finally, **MaximalResponseAxes** computes the variance of the ipsilateral angular relationships between semicircular duct planes from orthogonality (90var[2]), as well as their average deviation from orthogonality (90dev[3]), by applying the formulae:

$$90_{\mathrm{var}} = \frac{\left(90 - \vec{A}_n \wedge \vec{P}_n\right)^2 + \left(90 - \vec{A}_n \wedge \vec{L}_n\right)^2 + \left(90 - \vec{L}_n \wedge \vec{P}_n\right)^2}{3}$$

and

$$90_{dev} = \frac{\sqrt{\left(90 - \vec{A}_n \wedge \vec{P}_n\right)^2} + \sqrt{\left(90 - \vec{A}_n \wedge \vec{L}_n\right)^2} + \sqrt{\left(90 - \vec{L}_n \wedge \vec{P}_n\right)^2}}{3}$$

where 90_{var} corresponds to the variance of the ipsilateral angular relationships between the maximal response planes of the semicircular ducts from orthogonality, as defined by Malinzak et al.[2]; and where 90_{dev} corresponds to the average deviation of the ipsilateral angular relationships between the maximal response planes of the semicircular ducts from orthogonality, as defined by Berlin et al.[3].

At the end of the procedure, **MaximalResponseAxes** output several files containing information on all computed metrics described above.

c. Input files

As input, **MaximalResponseAxes** needs the file:

SPECIESNAME_R.lineset

to be placed in the folder **/AriadneToolbox/Data/SPECIESNAME/** (see section I.iii.3.). Note that this input file is suppressed when **MaximalResponseAxes** work is over.

d. Output files

MaximalResponseAxes outputs the files:

SPECIESNAME_R_Raw.length **SPECIESNAME_R_Raw.rawvector**

SPECIESNAME_R_Raw.earea **SPECIESNAME_R_Raw.ipsiangle**

in the folder **/AriadneToolbox/Data/SPECIESNAME/**.

It also outputs the files:

rma_SPECIESNAME_Ant_SD.lineset **rma_SPECIESNAME_Lat_SD.lineset**

rma_SPECIESNAME_Post_SD.lineset

in the folder **/AriadneToolbox/Data/SPECIESNAME/ScanRef/MaxAxes/**, with '**lma_**' instead of '**rma_**' for a left labyrinth.

.length files contain information about the length of the central streamlines running inside each part of the semicircular duct system.

.earea files contain information about (1) the maximal area enclosed by the central streamline of each semicircular duct, and (2) the mean radius of curvature (R) of each semicircular duct.

.rawvector files contain information, based on a model of uncoupled ducts, about (1) the vector components of the maximal response plane of each semicircular duct, in the scan frame of reference, and (2) the center of rotation of each semicircular duct, in the scan frame of reference.

.ipsiangle files contain information, based on a model of uncoupled ducts, about (1) the ipsilateral angular relationships of the maximal response planes of the semicircular ducts, (2) the variance of the ipsilateral angular relationships of the maximal response planes of the semicircular ducts from orthogonality (90var), and (4) the average deviation of the ipsilateral angular relationships of the maximal response planes of the semicircular ducts from orthogonality (90dev).

.lineset files can be opened in Avizo 6 or 7 and can be visualized using [Display> Line Set View]. They graphically represent the excitation direction of the maximal response axis of each semicircular duct, following the right hand rule and based on a model of uncoupled ducts.

5. DuctForm

a. Description

DuctForm is an executable, written in C, which computes:

- the mean cross-sectional area of the endolymphatic volume contained inside each part of the semicircular duct system
- the mean cross-sectional radius (r) of the endolymphatic volume contained inside the slender part of each semicircular duct
- ratios between the mean cross-sectional radii of the endolymphatic volumes contained inside each part of a semicircular duct and that of its respective slender part
- the mean wall shape drag factors of the cross-sections of the endolymphatic volume contained inside each part of the semicircular duct system

DuctForm can either be used as stand-alone software by double-clicking on **DuctForm.exe**, in the folder **/AriadneToolbox/**, and following instructions appearing in the console, or as part of the batch processes **Ariadne_SCDS.bat**, **Ariadne_SCDS_Lite.bat** or **MorphometryAuto.bat** (sections I.i & I.iii.)

b. Algorithm

In order to compute all above mentioned metrics, **DuctForm** first reads-in files containing information about lengths, volumes and inner wall surface areas of the parts of the semicircular duct system (sections I.iii.1., I.iii.2. & I.iii.4.). Then it applies the following formulae, in order to retrieve the mean cross-sectional area of each part:

$$a_{n,k} = \frac{V_{n,k}}{L_{n,k}}$$

and

$$a_k = \frac{V_k}{L_k}$$

where $a_{n,k}$ corresponds to the mean cross-sectional area of the part $_n$ of the semicircular duct $_k$; and a_k corresponds to the mean cross-sectional area of the semicircular duct $_k$; where $V_{n,k}$ corresponds to the volume of the part $_n$ of the semicircular duct $_k$; and V_k corresponds to the volume of the semicircular duct $_k$; and where $L_{n,k}$ corresponds to the length of the central streamline of the part $_n$ of the semicircular duct $_k$; and L_k corresponds to the length of the central streamline of the semicircular duct $_k$.

Then, **DuctForm** applies the following formula, in order to retrieve the mean wall shape drag factors[8] of the cross-sections of the endolymphatic volume contained inside each part of the semicircular duct system:

$$\lambda_{\mu n,k} = \frac{2S_{n,k}^2}{L_{n,k}V_{n,k}}$$

where $\lambda_{\mu n,k}$ corresponds to the mean wall shape drag factor of the part $_n$ of the semicircular duct $_k$; and where $S_{n,k}$ corresponds to the surface of the inner walls of the part $_n$ of the semicircular duct $_k$.

Finally, from these parameters, the mean cross-sectional radius of the slender part of each semicircular duct, as well as the ratio they form with the mean cross-sectional radii of other parts are computed, by applying the formulae:

$$r_{s,k} = \frac{\sqrt{\frac{\lambda_{\mu s,k} a_{s,k}}{2}} + 2\sqrt{\pi a_{s,k}}}{4\pi}$$

,

$$\gamma_k = \frac{1}{r_{s,k}} \frac{\sqrt{\frac{\lambda_{\mu cc,k} a_{cc,k}}{2}} + 2\sqrt{\pi a_{cc,k}}}{4\pi}$$

,

$$\beta_k = \frac{1}{r_{s,k}} \frac{\sqrt{\frac{\lambda_{\mu u,k} a_{u,k}}{2}} + 2\sqrt{\pi a_{u,k}}}{4\pi}$$

and

$$\delta_k = \frac{1}{r_{s,k}} \frac{\sqrt{\frac{\lambda_{\mu a,k} a_{a,k}}{2}} + 2\sqrt{\pi a_{a,k}}}{4\pi}$$

where $r_{s,k}$ corresponds to the mean cross-sectional radius of the slender part $_s$ of the semicircular duct $_k$; and where γ_k , β_k and δ_k correspond respectively to the ratio between, on the one hand, the mean cross-sectional radius of the common crus, of the utricular part, and of the ampullar part of the semicircular duct $_k$, and, on the other hand, the mean cross-sectional radius of the slender part of the semicircular duct $_k$.

At the end of the procedure, **DuctForm** output several files containing information on all computed metrics described above.

c. Input files

As input, **DuctForm** needs the files:

SPECIESNAME_R_Raw.volume **SPECIESNAME_R_Raw.length**

SPECIESNAME_R_Raw.surface

to be placed in the folder **/AriadneToolbox/Data/SPECIESNAME/** (see sections I.iii.1, I.iii.2 & I.iii.4.).

d. Output files

DuctForm outputs the files:

SPECIESNAME_R_Raw.carea **SPECIESNAME_R_Raw.wallshape**

in the folder **/AriadneToolbox/Data/SPECIESNAME/**.

.carea files contain information about (1) the mean cross-sectional area of the endolymphatic volume contained inside each part of the semicircular duct system, (2) the mean cross-sectional radius (r) of the endolymphatic volume contained inside each slender part of the semicircular duct system, and (3) ratios between the mean cross-sectional radii of the endolymphatic volumes contained inside each part of the semicircular duct and that of their respective slender part.

.wallshape files contain information about the mean wall shape drag factors of the cross-sections of the endolymphatic volume contained inside each part of the semicircular duct system.

6. CupulaMorpho

a. Description

CupulaMorpho is an executable, written in C, which computes:

- the mean cross-sectional areas of the cupulae
- the central cross-sectional areas of the cupulae
- the mean thickness of the cupulae, testing three different assumptions on stereocilia/kinocilia length
- the thickness of the cupulae according to Rabbitt's empirical law[4].

CupulaMorpho can either be used as stand-alone software by double-clicking on **CupulaMorpho.exe**, in the folder **/AriadneToolbox/**, and following instructions appearing in the console, or as part of the batch processes **Ariadne_SCDS.bat**, **Ariadne_SCDS_Lite.bat** or **MorphometryAuto.bat** (sections I.i & I.iii.)

b. Algorithm

Just like **VolumeCalc** (section I.iii.1.), **CupulaMorpho** implements the divergence theorem in order to compute the volume of the inputted STL files of the cupulae (section I.iv. of the Ariadne Manual – Data preparation). To do so, it reads-in the coordinates of the vertices of each STL file and applies the following formula:

$$V_{c,k} = \frac{1}{6}\sum_{i=1}^{N_{Vc,k}} \vec{p}_{Vc,k,i,1} \cdot \left(\vec{p}_{Vc,k,i,2} \times \vec{p}_{Vc,k,i,3} - \vec{p}_{Vc,k,i,2} \times \vec{p}_{Vc,k,i,1} - \vec{p}_{Vc,k,i,1} \times \vec{p}_{Vc,k,i,3} \right)$$

where $V_{c,k}$ corresponds to the volume of the cupula of the semicircular duct $_k$; where $\vec{p}_{Vc,k,i,1}$, $\vec{p}_{Vc,k,i,2}$ and $\vec{p}_{Vc,k,i,3}$ correspond to vectors running from the origin to the first, second

and third vertices of the i-th triangle of the mesh of the cupula of the semicircular duct $_k$; and where $N_{Vc,k}$ corresponds to the number of triangles contained in the mesh of the cupula of the semicircular duct $_k$.

Then, just like **SurfaceCalc** (section I.iii.2.), **CupulaMorpho** uses properties of the cross product in order to compute (1) surfaces of the contact areas between the cupulae and the endolymph medium, on their slender duct and utricular sides, and (2) areas of middle-cross section of the cupulae (section I.iv. of the Ariadne Manual – Data preparation). To do so, it reads-in the coordinates of the vertices of each corresponding STL file and applies the following formulae:

$$a_{c\_duct,k} = \frac{1}{2} \sum_{i=1}^{N_{c\_duct,k}} \left\| \left(\vec{p}_{c\_duct,k,i,2} - \vec{p}_{c\_duct,k,i,1} \right) \times \left(\vec{p}_{c\_duct,k,i,3} - \vec{p}_{c\_duct,k,i,1} \right) \right\|$$

,

$$a_{c\_ut,k} = \frac{1}{2} \sum_{i=1}^{N_{c\_ut,k}} \left\| \left(\vec{p}_{c\_ut,k,i,2} - \vec{p}_{c\_ut,k,i,1} \right) \times \left(\vec{p}_{c\_ut,k,i,3} - \vec{p}_{c\_ut,k,i,1} \right) \right\|$$

and

$$a_{c\_mid,k} = \frac{1}{2} \sum_{i=1}^{N_{c\_mid,k}} \left\| \left(\vec{p}_{c\_mid,k,i,2} - \vec{p}_{c\_mid,k,i,1} \right) \times \left(\vec{p}_{c\_mid,k,i,3} - \vec{p}_{c\_mid,k,i,1} \right) \right\|$$

where $a_{c\_duct,k}$ corresponds to the surface of the contact area between the cupula of the semicircular duct $_k$ and the endolymph medium, on the slender duct side of the ampulla; $a_{c\_ut,k}$ corresponds to the surface of the contact area between the cupula of the semicircular duct $_k$ and the endolymph medium, on the utricle side of the ampulla; and $a_{c\_mid,k}$ corresponds to the surface area of the cross-section of the cupula of the semicircular duct $_k$, when cut at half its thickness; where $\vec{p}_{c\_duct,k,i,1}$, $\vec{p}_{c\_duct,k,i,2}$ and $\vec{p}_{c\_duct,k,i,3}$ correspond to vectors running from origin to the first, second and third vertices of the i-th triangle of the contact area between the cupula of the semicircular duct $_k$ and the endolymph medium, on the slender duct side of the ampulla; $\vec{p}_{c\_ut,k,i,1}$, $\vec{p}_{c\_ut,k,i,2}$ and $\vec{p}_{c\_ut,k,i,3}$ correspond to vectors running from origin to the first, second and third vertices of the i-th triangle of the contact area between the cupula of the semicircular duct $_k$ and the endolymph medium, on the utricle side of the ampulla; and $\vec{p}_{c\_mid,k,i,1}$, $\vec{p}_{c\_mid,k,i,2}$ and $\vec{p}_{c\_mid,k,i,3}$ correspond to vectors running from origin to the first, second and third vertices of the i-th triangle of the mesh of the middle cross-section of the cupula of the semicircular duct $_k$; and where $N_{c\_duct,k}$ corresponds to the number of triangles contained in the mesh of the contact area between the cupula of the semicircular duct $_k$ and the endolymph medium, on the slender duct side of the ampulla; $N_{c\_ut,k}$ corresponds to the number of triangles contained in the mesh of the contact area between the cupula of the semicircular duct $_k$ and the

endolymph medium, on the utricle side of the ampulla; and $N_{c\_mid,k}$ corresponds to the number of triangles contained in the mesh of the middle cross-section of the cupula of the semicircular duct $_k$.

From these results, **CupulaMorpho** then computes the mean cross-sectional area of each cupula by applying the following formula:

$$a_{c,k} = \left(\frac{a_{c\_duct,k} + 2a_{c\_mid,k} + a_{c\_ut,k}}{4} \right)$$

where $a_{c,k}$ corresponds to the mean cross-sectional area of the cupula of the semicircular duct $_k$.

Next, the mean thickness of each cupula is computed through the use of the following formula:

$$t_{c,k} = \frac{V_{c,k}}{a_{c,k}}$$

where $t_{c,k}$ corresponds to the mean thickness of the cupula of the semicircular duct $_k$.

As stated in section I.iv. of the Ariadne Manual – Data preparation, cupulae on which **CupulaMorpho** applied all previous formulae correspond to what can be called "baseline" models. Baseline models neglect how stereocilia and/or kinocilia affect cupula structure and picture cupulae as simple tridimensional extrusions of the shape of corresponding *crista ampullaris* into the above ampulla volume. However, as shown in Fig.21 & 22 of the section I.iv. of the Ariadne Manual – Data preparation, stereocilia/kinocilia length linearly affect cupula thickness, and thus also cupula volume. **CupulaMorpho** takes this information into account and then recomputes the thickness and volume of each cupula, assuming small (30µm), medium (60µm) and long (90µm) stereocilia/kinocilia and considering θ to be equal to 45° (Fig.22, section I.iv. of the Ariadne Manual – Data preparation). To do so, it applies the following formulae:

$$t_{c,k,h=30} = t_{c,k} + 0.042$$

,

$$t_{c,k,h=60} = t_{c,k} + 0.085$$

,

$$t_{c,k,h=90} = t_{c,k} + 0.127$$

and

$$V_{c,k,h=30} = a_{c,k} t_{c,k,h=30}$$

,

$$V_{c,k,h=60} = a_{c,k} t_{c,k,h=60}$$

,

$$V_{c,k,h=90} = a_{c,k} t_{c,k,h=90}$$

where $t_{c,k,h=30}$, $t_{c,k,h=60}$ and $t_{c,k,h=90}$ respectively correspond to the mean thickness of the cupula of the semicircular duct $_k$ when assuming the presence of long $_{h=90}$, medium $_{h=60}$ and short $_{h=30}$

stereocilia/kinocilia at the surface of the *crista ampullaris*; and where $V_{c,k,h=30}$, $V_{c,k,h=60}$ and $V_{c,k,h=90}$ correspond to assumed volumes of the cupula of the semicircular duct.

Aside from previous calculations and for information purposes only, **CupulaMorpho** also computes the mean thickness of each cupula by following the empirical equation provided by Rabbitt and colleagues[4]:

$$t_{c,k,Rabbitt} = 0.7.\sqrt{\frac{a_{c,k}}{\pi}}$$

where $t_{c,k,Rabbitt}$ corresponds to the mean thickness of the cupula of the semicircular duct $_k$ as computed following Rabbitt's equation.

At the end of the procedure, **CupulaMorpho** output one file (two if both right and left labyrinths are studied) containing information on all computed metrics described above.

c. Input files

As input, **CupulaMorpho** needs the files:

rv_SPECIESNAME_Ant_Cup.stl

rv_SPECIESNAME_Lat_Cup.stl

rv_SPECIESNAME_Post_Cup.stl

to be placed in the folder **/AriadneToolbox/Data/SPECIESNAME/ScanRef/Volumes/** (see section I.iv. of the Ariadne Manual – Data preparation).

It also needs the files:

rs_SPECIESNAME_Ant_Cup_Ut.stl

rs_SPECIESNAME_Ant_Cup_Duct.stl

rs_SPECIESNAME_Ant_Cup_Mid.stl

rs_SPECIESNAME_Post_Cup_Ut.stl

rs_SPECIESNAME_Post_Cup_Duct.stl

rs_SPECIESNAME_Post_Cup_Mid.stl

rs_SPECIESNAME_Lat_Cup_Ut.stl

rs_SPECIESNAME_Lat_Cup_Duct.stl

rs_SPECIESNAME_Lat_Cup_Mid.stl

to be placed in the folder **/AriadneToolbox/Data/SPECIESNAME/ScanRef/Surfaces/** (see section I.iv. of the Ariadne Manual – Data preparation).

d. Output files

CupulaMorpho outputs the file:

SPECIESNAME_R_Raw.cupula

in the folder **/AriadneToolbox/Data/SPECIESNAME/**.

.cupula files contain information about (1) the mean cross-sectional areas of the cupulae, (2) the central cross-sectional areas of the cupulae, (3) the mean thickness of the cupulae, testing three different assumptions on stereocilia/kinocilia length, and (4) the thickness of the cupulae according to Rabbitt's empirical law.

7. ShrinkCor

a. Description

ShrinkCor is an executable, written in C, which:

- computes shrinkage correction factors derived from empirical modelling of relative shrinkage inside the toadfish inner ear[5]
- computes resolution correction factors to adjust for potential errors caused by manual segmentation
- applies both factors to volumes, surfaces, lengths, areas, vector components and angles that were previously calculated by **VolumeCalc** (section I.iii.1.), **SurfaceCalc** (section I.iii.2.), **MaximalResponseAxes** (section I.iii.4.), **DuctForm** (section I.iii.5.) and **CupulaMorpho** (section I.iii.6.).

ShrinkCor can either be used as stand-alone software by double-clicking on **ShrinkCor.exe**, in the folder **/AriadneToolbox/**, and following instructions appearing in the console, or as part of the batch processes **Ariadne_SCDS.bat**, **Ariadne_SCDS_Lite.bat** or **MorphometryAuto.bat** (sections I.i & I.iii.).

b. Input parameters

The first parameter **ShrinkCor** asks the user to input is the segmentation error factor he has to use to provide an error range with outputted parameters. The segmentation error factor corresponds to the amount of voxel that needs to be added, or removed, everywhere on the surface of the reconstructed semicircular duct system. It is needed to take the potential errors that may have happen during the segmentation process into account. Based on manual segmentation techniques, the inter-observer test we provided in the companion paper[1] suggests a maximum value of 0.24 for this parameter. However, since the three participants of this inter-observer test are quite experienced with the process of segmenting labyrinths, it is very likely that this value would need to be increased in the case of users who are completely new to the procedure.

Next, **ShrinkCor** prompt the user to input the level of shrinkage correction he wants to use, to correct computed morphological and biomechanical parameters. This factor varies between "0" and "1", "0" being the absence of correction for shrinkage, whereas "1" corresponds to a full shrinkage correction. The way **ShrinkCor** corrects for shrinkage is explained in detail below, but the user has to understand here that using the full correction option may eventually leads to overcorrection of outputted parameters. In fact, we recommend first to visually checking for signs of shrinkage in the studied morphology before inputting this parameter. Visual signs of shrinkage include the presence of ridges or spikes on the surface of the reconstructed labyrinth, of obvious collapsed structures, and of "breaks" into the smooth trajectory at the surface of the ducts. If none of these signs are found, we suggest to keep the shrinkage correction factor small.

To work, **ShrinkCor** also needs information on how sensitive each semicircular duct part is to shrinkage, and in particular on how it differentially affects their inner cross-sectional radius and in their inner cross-section perimeter. Values do not need to describe how actually shrunk each part of the labyrinth is, since **ShrinkCor** independently derives shrinkage correction factors from geometrical analysis of the semicircular duct system. However, they have to accurately describe the relative difference existing between the sensitivities of each semicircular duct part to shrinkage, inside and between the parts. In total, 8 parameters are needed, describing the sensitivity of both the inner cross-sectional radius and perimeter of the slender part, of the common crus part, of the utricular part and of the ampullar part, to shrinkage.

Following[15], the sensitivity to shrinkage is expressed as:

$$Sh_p = 100 \frac{p - p_{shrink}}{p}$$

where p and p_{shrink} correspond respectively to the measurement of a given parameter in a living and a shrunk animal; and where Sh_p corresponds to the percent tissue shrinkage of the parameter p .

When using **ShrinkCor** executable, users are prompted by the console to manually input the 8 shrinkage parameters as they see fit. On the contrary, for the batch processes **Ariadne_SCDS.bat**, **Ariadne_SCDS_Lite.bat** or **MorphometryAuto.bat** (sections I.i & I.iii.), we directly implemented an empirical model derived from Ghanem et al. study on shrinkage of the toadfish inner ear[5]. By doing so, we necessarily assumed that the relative difference existing between the sensitivities of each semicircular duct part to shrinkage, as depicted in this study, was transposable to the studied semicircular duct system, and that, whether percent shrinkage of each part of the latest would actually be different than values depicted for the toadfish.

Based on raw measurements provided in this study, our model was built by taking the inner diameter of the cross-sections into account (removing the width of the membranes from the provided outer diameter), by assuming that a change in thickness of the membranes is comparable in size to a change in their cross-section perimeter, and by geometrically interpolating percent shrinkage across the semicircular duct, to empirically get mean estimates for the slender part, the ampullar part, the utricular part and the common crus part.

In our model, sensitivities of each semicircular duct part to shrinkage are described as follow:

	Inner cross-sectional radius shrinkage	Inner cross-section perimeter shrinkage
Slender part	16 %	15 %
Common crus	24 %	12 %
Utricle	31 %	9 %
Ampulla	14 %	18 %

As stated above, these values are not used as is by **ShrinkCor** to correct measurements done on studied semicircular duct systems. Instead, ratios between these values, inside and between the parts, along with morphometric aspects of the semicircular ducts, help computing actual shrinkage correction factors. It is worth noting that in Ghanem et al. study[5], from which the above model is

derived, depicted shrinkage occurred on membranous labyrinths that were both extracted from the otic capsule and embedded in resin. It is then not surprising that **ShrinkCor** generally computes shrinkage correction factors that are much smaller than the ones presented here when studying membranous labyrinth that have been visualized using our proposed visualization technique[1].

Aside from shrinkage parameters, **ShrinkCor** also requires the scan resolution of the corresponding membranous labyrinth to be inputted in micrometers. If the user runs **ShrinkCor** through the executable, scan resolution has to be inputted manually when prompted by the console. However, if the user runs **ShrinkCor** through any of the batch processes **Ariadne_SCDS.bat**, **Ariadne_SCDS_Lite.bat** or **MorphometryAuto.bat** (sections I.i & I.iii.), scan resolution is then read from the **TaxaInfo.txt** file, which has to be prepared beforehand (see section I.i., step (2)).

c. Algorithm

In order to compute shrinkage correction factors, **ShrinkCor** first reads-in files containing information about mean wall shape drag factors of the parts of the semicircular duct system (sections I.iii.5.). Then, it builds upon the properties of the mean wall shape drag factors, which are:

$$\lambda_{\mu n,k} = \frac{2S_{n,k}^2}{L_{n,k}V_{n,k}} = \frac{2P_{n,k}^2}{\pi r_{n,k}^2}$$

and

$$\lambda_{\mu n,k,Nat} \geq 8\pi$$

where $\lambda_{\mu n,k}$ and $\lambda_{\mu n,k,Nat}$ correspond respectively to the shrunk and native mean wall shape drag factor of the part $_n$ of the semicircular duct $_k$; where $S_{n,k}$ corresponds to the surface area of the inner walls of the part $_n$ of the semicircular duct $_k$; where $V_{n,k}$ corresponds to the volume of the part $_n$ of the semicircular duct $_k$; where $L_{n,k}$ corresponds to the length of the central streamline of the part $_n$ of the semicircular duct $_k$; where $r_{n,k}$ corresponds to the mean cross-sectional radius of the inner walls of the part $_n$ of the semicircular duct $_k$; and where $P_{n,k}$ corresponds to the mean cross-section perimeter of the inner walls of the part $_n$ of the semicircular duct $_k$.

Above mentioned equations clearly indicate that the native mean wall shape drag factor of each semicircular duct part (1) cannot be smaller than 8π, which corresponds to a perfectly circular cross-section shape, and (2) is affected by shrinkage of both the inner cross-sectional radius and perimeter of the corresponding part. It is possible to re-express the native mean wall shape drag factor, to allow the effect of the inputted shrinkage model to appear, by writing:

$$\lambda_{\mu n,k,Nat} = \lambda_{\mu n,k}\alpha_{n,k}^2$$

where

$$\alpha_{n,k}^2 = \frac{\lambda_{\mu n,k,Nat}}{\lambda_{\mu n,k}}$$

which leads to

$$\alpha_{n,k}^2 = \frac{2P_{n,k,Nat}^2}{\pi r_{n,k,Nat}^2} \cdot \frac{\pi r_{n,k}^2}{2P_{n,k}^2}$$

thus

$$\alpha_{n,k} = \frac{\dfrac{r_{n,k}}{r_{n,k,Nat}}}{\dfrac{P_{n,k}}{P_{n,k,Nat}}}$$

giving

$$\alpha_{n,k} = \frac{\dfrac{100r_{n,k} + 100r_{n,k,Nat} - 100r_{n,k,Nat}}{r_{n,k,Nat}}}{\dfrac{100P_{n,k} + 100P_{n,k,Nat} - 100P_{n,k,Nat}}{P_{n,k,Nat}}}$$

thus

$$\alpha_{n,k} = \frac{100 - 100\left(\dfrac{r_{n,k,Nat} - r_{n,k}}{r_{n,k,Nat}}\right)}{100 - 100\left(\dfrac{P_{n,k,Nat} - P_{n,k}}{P_{n,k,Nat}}\right)}$$

and finally

$$\alpha_{n,k} = \left(\frac{100 - Sh_{d,n}\beta_{n,k}}{100 - Sh_{w,n}\beta_{n,k}}\right)$$

where $Sh_{d,n}$ and $Sh_{w,n}$ correspond respectively to the percent shrinkage of the mean inner cross-sectional radius and of the mean inner cross-section perimeter of the part $_n$ of the semicircular duct, as defined in the inputted model; where $\alpha_{n,k}^2$ corresponds to the ratio between mean wall shape drag factors of the native and of the shrunk part $_n$ of the semicircular duct; and where $\beta_{n,k}$ corresponds to the strength of the shrinkage affecting the part $_n$ of the semicircular duct, relative to the inputted percent shrinkage values.

From these equations, it appears that if $Sh_{d,n}$ and $Sh_{w,n}$ have different values, which is always the case in the model we derived from Ghanem's study[5], we should be able to find a unique value for $\beta_{n,k}$ which satisfies the equations:

$$\lambda_{\mu n,k} \cdot \left(\frac{100 - Sh_{d,n}\beta_{n,k}}{100 - Sh_{w,n}\beta_{n,k}}\right)^2 = 8\pi$$

and

$$\beta_{n,k} \geq 0$$

To do so, we need to write:

$$\lambda_{\mu n,k}.\left(100 - Sh_{d,n}\beta_{n,k}\right)^2 = 8\pi\left(100 - Sh_{w,n}\beta_{n,k}\right)^2$$

which leads to

$$10000\lambda_{\mu n,k} - 200Sh_{d,n}\beta_{n,k}\lambda_{\mu n,k} + Sh_{d,n}^2\beta_{n,k}^2\lambda_{\mu n,k} = 8000\pi - 1600\pi Sh_{w,n}\beta_{n,k} + 8\pi Sh_{w,n}^2\beta_{n,k}^2$$

and then to

$$\beta_{n,k}(1600\pi Sh_{w,n} - 200Sh_{d,n}\lambda_{\mu n,k}) + \beta_{n,k}^2(Sh_{d,n}^2\lambda_{\mu n,k} - 8\pi Sh_{w,n}^2) + (10000\lambda_{\mu n,k} - 80000\pi) = 0$$

which corresponds to a quadratic equation that can be solved using the formula:

$$\beta_{n,k} = \frac{-(1600\pi Sh_{w,n} - 200Sh_{d,n}\lambda_{\mu n,k}) - \sqrt{\Delta_{n,k}}}{2(Sh_{d,n}^2\lambda_{\mu n,k} - 8\pi Sh_{w,n}^2)}$$

where

$$\Delta_{n,k} = (1600\pi Sh_{w,n,k} - 200Sh_{d,n}\lambda_{\mu n,k})^2 - 4(Sh_{d,n}^2\lambda_{\mu n,k} - 8\pi Sh_{w,n}^2).(10000\lambda_{\mu n,k} - 80000\pi)$$

Values of $\beta_{n,k}$ retrieved this way correspond to highest strength factors that can have been applied on both percent shrinkage values of the part $_n$ of the semicircular duct $_k$, as supported by the shrunk morphology. Higher values for $\beta_{n,k}$ would imply that native cross-section shape of corresponding semicircular duct parts would be more than circular, which is impossible. **ShrinkCor** takes this information into account and then compute $\beta_{n,k}$ for each part $_n$ of each semicircular duct $_k$ to get highest strength factors that can have been applied on both their percent shrinkage values. Note that to compute the highest strength factor for the common crus, **ShrinkCor** uses the average of the mean wall shape factor of its anterior and posterior trajectories. It also uses the average of the mean wall shape factor of anterior and lateral trajectories to compute the highest strength factor of the anterior utricle.

ShrinkCor assumes that the relative difference existing between the inputted percent shrinkage values is comparable to the relative difference existing between the percent shrinkage values that really affected the semicircular duct system. This implies that the strength parameter $\beta_{n,k}$ that should be used to compute the shrinkage correction factors has to be the same for every part of the semicircular duct system. To compute it, **ShrinkCor** neglects negative values of $\beta_{n,k}$ and applies the following formulae:

$$SMS_{Full} = \min\left(\beta_{n,k}\right)$$

and

$$SMS = \tau.SMS_{Full}$$

where SMS_{Full} corresponds to the strength that has to be applied to the inputted percent shrinkage values to compute the full shrinkage correction factors; where τ corresponds to the level of shrinkage correction the user inputted; and where SMS corresponds to the strength that will be

applied to the inputted percent shrinkage values to compute the shrinkage correction factors that will actually be used.

From this, **ShrinkCor** then computes the shrinkage correction factors according to the formulae:

$$Sh_{d,n,Cor} = SMS.Sh_{d,n}$$

,

$$Sh_{w,n,Cor} = SMS.Sh_{w,n}$$

where $Sh_{d,n,Cor}$ and $Sh_{\mathrm{w},n,Cor}$ correspond respectively to the shrinkage correction factors that will be applied to the mean inner cross-sectional radius and the mean inner cross-section perimeter of the part $_n$ of the semicircular duct.

Next, **ShrinkCor** reads-in files containing information about all parameters it has to correct for both potential shrinkage and manual segmentation errors (sections I.iii.1., I.iii.2. , I.iii.4., I.iii.5. & I.iii.6.). It then starts computing corrected mean cross-sectional areas by applying the following formulae:

$$a_{n,k,MinusCor} = \pi \left(\sqrt{\frac{a_{n,k}}{\pi \left(1 - \frac{Sh_{d,n,Cor}}{100}\right)^2}} - \upsilon\Gamma \right)^2$$

,

$$a_{n,k,Cor} = \pi \left(\sqrt{\frac{a_{n,k}}{\pi \left(1 - \frac{Sh_{d,n,Cor}}{100}\right)^2}} \right)^2$$

and

$$a_{n,k,PlusCor} = \pi \left(\sqrt{\frac{a_{n,k}}{\pi \left(1 - \frac{Sh_{d,n,Cor}}{100}\right)^2}} + \upsilon\Gamma \right)^2$$

where $a_{n,k,Cor}$ corresponds to the mean cross-sectional area of the part $_n$ of the semicircular duct $_k$, after shrinkage correction was applied; where $a_{n,k,MinusCor}$ corresponds to the mean cross-sectional area of the part $_n$ of the semicircular duct $_k$, after shrinkage correction was applied and after the part was contracted by an amount of voxels $\upsilon\Gamma$ perpendicular to its surface; where $a_{n,k,PlusCor}$ corresponds to the mean cross-sectional area of the part $_n$ of the semicircular duct $_k$, after shrinkage correction was applied and after the part was dilated by an amount of voxels $\upsilon\Gamma$ perpendicular to its surface; where $a_{n,k}$ corresponds to the raw mean cross-sectional area of the part $_n$ of the semicircular duct $_k$; where υ corresponds to the segmentation error factor inputted by the user; and

where Γ corresponds to the resolution of the scan from with the membranous labyrinth was segmented, re-expressed in millimeters.

Using these corrected mean cross-sectional areas, **ShrinkCor** then applies the same formulae that are used by **DuctForm** and **CupulaMorpho** to compute mean cross-sectional radii and ratios, empirical cupulae thicknesses and average cross-sectional areas of the cupulae, all corrected for potential shrinkage and segmentation errors (see sections I.iii.5. & I.iii.6.). **ShrinkCor** also computes radial correction factors, which allow correcting landmark position for above mentioned errors, by applying the following formula:

$$\varepsilon_{n,k,xCor} = \sqrt{\frac{a_{n,k,xCor}}{\pi}} - \sqrt{\frac{a_{n,k}}{\pi}}$$

where $\varepsilon_{n,k,xCor}$ corresponds to the radial correction factor for the coordinates of landmarks of the part$_n$ of the semicircular duct$_k$, after shrinkage and segmentation corrections were applied; and where $xCor$ can either be $MinusCor$, Cor or $PlusCor$.

Note that to compute the radial factor for the common crus, **ShrinkCor** uses the average of the mean cross-sectional areas of its anterior and posterior trajectories. It also uses the average of the mean cross-sectional area of anterior and lateral trajectories to compute the radial factor for the anterior utricle, and it uses the average of the mean cross-sectional areas of the anterior and lateral trajectories of the anterior utricle, and of the posterior utricle to compute the radial factor for the common utricle.

This leads to the correction of landmark coordinates, by application of the formulae:

$$x_{i,n,k,xCor} = x_{i,n,k} + \frac{\varepsilon_{n,k,xCor}\left(x_{center,k} - x_{i,n,k}\right)}{\sqrt{\left(x_{center,k} - x_{i,n,k}\right)^2 + \left(y_{center,k} - y_{i,n,k}\right)^2 + \left(z_{center,k} - z_{i,n,k}\right)^2}}$$

,

$$y_{i,n,k,xCor} = y_{i,n,k} + \frac{\varepsilon_{n,k,xCor}\left(y_{center,k} - y_{i,n,k}\right)}{\sqrt{\left(x_{center,k} - x_{i,n,k}\right)^2 + \left(y_{center,k} - y_{i,n,k}\right)^2 + \left(z_{center,k} - z_{i,n,k}\right)^2}}$$

,

$$z_{i,n,k,xCor} = z_{i,n,k} + \frac{\varepsilon_{n,k,xCor}\left(z_{center,k} - z_{i,n,k}\right)}{\sqrt{\left(x_{center,k} - x_{i,n,k}\right)^2 + \left(y_{center,k} - y_{i,n,k}\right)^2 + \left(z_{center,k} - z_{i,n,k}\right)^2}}$$

where $\left\{x_{i,n,k}, y_{i,n,k}, z_{i,n,k}\right\}$ correspond to the raw three-dimensional coordinates of the i-th landmark of the part$_n$ of the semicircular duct$_k$, in the scan frame of reference; $\left\{x_{center,k}, y_{center,k}, z_{center,k}\right\}$ correspond to the raw three-dimensional coordinates of the center of rotation of the semicircular duct$_k$, in the scan frame of reference; and where $\left\{x_{i,n,k,xCor}, y_{i,n,k,xCor}, z_{i,n,k,xCor}\right\}$ correspond to the three-dimensional coordinates of the i-th

landmark of the part $_n$ of the semicircular duct $_k$, in the scan frame of reference, after shrinkage and segmentation corrections were applied.

Using corrected landmark sets, **ShrinkCor** then applies the same formulae that are used by **MaximalResponseAxes** to compute lengths, enclosed areas, radii of curvature, semicircular duct plane components, center of rotation, ipsilateral angular relationships, 90var and 90dev, all corrected for potential shrinkage and segmentation errors (see section I.iii.4.).

Next, correction of the volume of endolymph that is contained inside each semicircular duct part is mostly done by applying the formula:

$$V_{n,k,xCor} = a_{n,k,xCor} L_{n,k,xCor}$$

where $V_{n,k,xCor}$ corresponds to the volume of the part $_n$ of the semicircular duct $_k$, after shrinkage and segmentation corrections were applied; and where $L_{n,k,xCor}$ corresponds to the length of the central streamline of the part $_n$ of the semicircular duct $_k$, after shrinkage and segmentation corrections were applied.

This step is followed by the correction of the mean wall shape drag factors, through the application of the following formula:

$$\lambda_{\mu n,k,xCor} = \frac{\lambda_{\mu n,k}\left(1-\frac{Sh_{d,n,xCor}}{100}\right)^2}{\left(1-\frac{Sh_{w,n,xCor}}{100}\right)^2}$$

where $\lambda_{\mu n,k,xCor}$ corresponds to the mean wall shape drag factors of the part $_n$ of the semicircular duct $_k$, after shrinkage and segmentation corrections were applied.

Finally, **ShrinkCor** corrects the surface areas of the inner walls of each semicircular duct part, by applying the following formula:

$$S_{n,k,xCor} = \sqrt{\frac{V_{n,k,xCor} L_{n,k,xCor} \lambda_{\mu n,k,xCor}}{2}}$$

where $S_{n,k,xCor}$ corresponds to the surface areas of the inner walls of the part $_n$ of the semicircular duct $_k$, after shrinkage and segmentation corrections were applied.

At the end of the procedure, **ShrinkCor** output several files containing information on all computed metrics described above.

d. Input files

As input, **ShrinkCor** needs the files:

rcs_SPECIESNAME_Sa.landmarkAscii

rcs_SPECIESNAME_Sp.landmarkAscii

rcs_SPECIESNAME_Sl.landmarkAscii

rcs_SPECIESNAME_CC.landmarkAscii

rcs_SPECIESNAME_Aa.landmarkAscii

rcs_SPECIESNAME_Ap.landmarkAscii

rcs_SPECIESNAME_Al.landmarkAscii

rcs_SPECIESNAME_Ua.landmarkAscii

rcs_SPECIESNAME_Up.landmarkAscii

rcs_SPECIESNAME_SC.landmarkAscii

rcs_SPECIESNAME_Uc.landmarkAscii

to be placed in the folder **/AriadneToolbox/Data/SPECIESNAME/ScanRef/Landmarks/** (see section I.iii. of the Ariadne Manual – Data preparation).

It also needs the files:

SPECIESNAME_R_Raw.volume

SPECIESNAME_R_Raw.length

SPECIESNAME_R_Raw.carea

SPECIESNAME_R_Raw.wallshape

SPECIESNAME_R_Raw.cupula

SPECIESNAME_R_Raw.surface

SPECIESNAME_R_Raw.earea

SPECIESNAME_R_Raw.rawvector

SPECIESNAME_R_Raw.ipsiangle

to be placed in the folder **/AriadneToolbox/Data/SPECIESNAME/** (see sections I.iii.1., I.iii.4. ., I.iii.5. & I.iii.6.). Note that these input files are suppressed when **ShrinkCor** work is over.

e. Output files

ShrinkCor outputs the files:

SPECIESNAME_R_Suffix.volume

SPECIESNAME_R_Suffix.length

SPECIESNAME_R_Suffix.carea

SPECIESNAME_R_Suffix.wallshape

SPECIESNAME_R_Suffix.cupula

SPECIESNAME_R_Suffix.surface

SPECIESNAME_R_Suffix.earea

SPECIESNAME_R_Suffix.rawvector

SPECIESNAME_R_Suffix.ipsiangle

in the folder **/AriadneToolbox/Data/SPECIESNAME/**, with **Suffix** replaced by **Cor**, **MinusCor** and by **PlusCor** in three sets of files.

Cor, **MinusCor** and **PlusCor** sets of files contain information similar to what can be found in the corresponding **Raw** set of files (see sections I.iii.1., I.iii.2., I.iii.4. & I.iii.5.), but corrected for potential errors due to shrinkage and manual segmentation for the latter two. These files also contain

information about the shrinkage correction factors that have been applied to morphometric parameters.

8. SCDSReferenceFrame

a. Description

SCDSReferenceFrame is an executable, written in C, which computes, based on a model of uncoupled ducts, and each time with three levels of correction for potential shrinkage and segmentation errors:

- the vector components of the maximal response plane of each semicircular duct, in the vestibular frame of reference[6]
- the center of rotation of each semicircular duct, in the vestibular frame of reference
- the synergistic angular relationships of the maximal response planes of the semicircular ducts
- the angular relationships of the maximal response planes of the semicircular ducts, with the three vestibular reference planes of the head
- the variance of the synergistic angular relationships of the maximal response planes of the semicircular ducts from planarity (180var)
- the average deviation of the synergistic angular relationships of the maximal response planes of the semicircular ducts from planarity (180dev)

SCDSReferenceFrame also output 'matrix' files that can be used by **DataVestibularReference** (section I.vi.1.) and **STLVestibularReference** (section I.vi.2.) to mirror data objects (linesets, landmarks, STLs) across the mid-sagittal plane of the head.

SCDSReferenceFrame can either be used as stand-alone software by double-clicking on **SCDSReferenceFrame.exe**, in the folder **/AriadneToolbox/**, and following instructions appearing in the console, or as part of the batch processes **Ariadne_SCDS.bat**, **Ariadne_SCDS_Lite.bat** or **MorphometryAuto.bat** (sections I.i & I.iii.)

b. Algorithm

In order to compute all above mentioned metrics, **SCDSReferenceFrame** first needs a complete vector set of the semicircular duct system to work on. If the user provides **SCDSReferenceFrame** with data about the right or the left labyrinth only, the software then needs to rebuild the complete vector set before proceeding further. To do so, **SCDSReferenceFrame** reads-in files containing information about vector components of the maximal response planes of studied semicircular ducts, as well as a file containing information about the mid-sagittal plane of the vestibular frame of reference (section I.vi. of the Ariadne Manual – Data preparation, and sections I.iii.4. & I.iii.7. of this manual). Then it applies the following formula, in order to rebuild the missing parts of the vector set of the semicircular duct system:

$$\vec{V}_k' = S\vec{V}_k$$

where

$$S = \begin{bmatrix} \left(MP_x^2 - MP_y^2 - MP_z^2\right) & 2MP_xMP_y & 2MP_xMP_z \\ 2MP_xMP_y & \left(-MP_x^2 + MP_y^2 - MP_z^2\right) & 2MP_yMP_z \\ 2MP_xMP_z & 2MP_yMP_z & \left(-MP_x^2 - MP_y^2 + MP_z^2\right) \end{bmatrix}$$

corresponds to the matrix of reflection across the mid-sagittal plane of the vestibular frame of reference, which is corrected to get adequate excitation direction for reflected vectors; where $\overrightarrow{MP} = \left(MP_x, MP_y, MP_z\right)$ corresponds to the unit vector that is normal to the mid-sagittal plane of the head, in the scan frame of reference; and where

$$\vec{V}_k = \begin{pmatrix} V_{x,k} \\ V_{y,k} \\ V_{z,k} \end{pmatrix}$$

and

$$\vec{V}_k^{'} = \begin{pmatrix} V_{x,k}^{'} \\ V_{y,k}^{'} \\ V_{z,k}^{'} \end{pmatrix}$$

correspond respectively to unit activatory vectors that are normal to the inputted and reflected maximal response planes of the semicircular duct $_k$ of the membranous labyrinth, in the scan frame of reference.

After having rebuilt the complete vector set of the semicircular duct system, **SCDSReferenceFrame** computes the coordinates of reflected centers of rotation of the semicircular ducts, by applying the following formula:

$$C_k^{'} = S(MPP - C_k) + MPP$$

where

$$MPP = \begin{pmatrix} MPP_x \\ MPP_y \\ MPP_z \end{pmatrix}$$

corresponds to any point located on the mid-sagittal plane of the head; and where

$$C_k = \begin{pmatrix} C_{x,k} \\ C_{y,k} \\ C_{z,k} \end{pmatrix}$$

and

$$C_k^{'} = \begin{pmatrix} C_{x,k}^{'} \\ C_{y,k}^{'} \\ C_{z,k}^{'} \end{pmatrix}$$

correspond respectively to the inputted and reflected centers of rotation of the k-th semicircular duct, in the scan frame of reference.

SCDSReferenceFrame then uses the complete vector set of the semicircular duct system to compute the synergistic angular relationships of semicircular duct planes, applying the formulae:

$$\vec{A}_r \wedge \vec{P}_l = arc\cos(\vec{A}_r \cdot \vec{P}_l)$$

,

$$\vec{A}_l \wedge \vec{P}_r = arc\cos(\vec{A}_l \cdot \vec{P}_r)$$

and

$$\vec{L}_r \wedge \vec{L}_l = arc\cos(\vec{L}_r \cdot \vec{L}_l)$$

where $\vec{A}_r \wedge \vec{P}_l$, $\vec{A}_l \wedge \vec{P}_r$ and $\vec{L}_r \wedge \vec{L}_l$ correspond to the angles between maximal response planes of the right anterior and left posterior semicircular ducts, left anterior and right posterior semicircular ducts and right lateral and left lateral semicircular ducts, respectively; and where $\vec{A}_n$, $\vec{P}_n$ and $\vec{L}_n$ correspond to unit vectors that are normal to the maximal response planes of the right $_r$ or left $_l$ anterior, posterior and lateral semicircular ducts respectively, oriented along their excitation direction.

From this, **SCDSReferenceFrame** computes the variance of the synergistic angular relationships between semicircular duct planes from planarity (180var[2]), as well as their average deviation from planarity (180dev), by applying the formulae:

$$180_{\mathrm{var}} = \frac{\left(180 - \vec{A}_r \wedge \vec{P}_l\right)^2 + \left(180 - \vec{A}_l \wedge \vec{P}_r\right)^2 + \left(180 - \vec{L}_r \wedge \vec{L}_l\right)^2}{3}$$

and

$$180_{dev} = \frac{\sqrt{\left(180 - \vec{A}_r \wedge \vec{P}_l\right)^2} + \sqrt{\left(180 - \vec{A}_l \wedge \vec{P}_r\right)^2} + \sqrt{\left(180 - \vec{L}_r \wedge \vec{L}_l\right)^2}}{3}$$

where 180_{var} corresponds to the variance of the synergistic angular relationships between the maximal response planes of the semicircular ducts from planarity, as defined by Malinzak et al.[2]; and where 180_{dev} corresponds to the average deviation of the synergistic angular relationships between the maximal response planes of the semicircular ducts from planarity.

To be able to compare the spatial biomechanics between various species, the complete vector set of the semicircular duct system has to be transferred from the native, randomly built, scan frame of reference $B = \{\vec{x}, \vec{y}, \vec{z}\}$, into a more meaningful and comparison-friendly frame of reference, which we called the vestibular frame of reference[4] $B' = \{\vec{X}, \vec{Y}, \vec{Z}\}$. The vestibular frame of reference is a head fixed frame of reference that is primarily defined as the combination of (1) the axis that is normal to the symmetry plane of the semicircular duct system, (2) the axis around which a rotation,

of a given magnitude, elicits the maximal joint response of the lateral semicircular ducts, and (3) the axis that is perpendicular to axes (1) and (2). These axes, by definition, correspond to the (1) pitch, (2) yaw, and (3) roll vestibular axes of rotation. As a frame of reference is rather defined by vectors than by axes, we arbitrarily chose to define the vestibular frame of reference by the combination of the (1) nose-down pitch $\vec{Y}$, (2) left yaw $\vec{Z}$ and (3) right roll $\vec{X}$ unit vectors of rotation. **SCDSReferenceFrame** then builds the orthonormal basis of the vestibular frame of reference by applying the following formulae:

$$\vec{Z} = \frac{\vec{L}_l - \vec{L}_r}{\left\| \vec{L}_l - \vec{L}_r \right\|} = \begin{pmatrix} Z_x \\ Z_y \\ Z_z \end{pmatrix}$$

,

$$\vec{Y} = \frac{\vec{A}_r + \vec{A}_l - \vec{P}_r - \vec{P}_l}{\left\| \vec{A}_r + \vec{A}_l - \vec{P}_r - \vec{P}_l \right\|} = \begin{pmatrix} Y_x \\ Y_y \\ Y_z \end{pmatrix}$$

and

$$\vec{X} = \frac{\vec{Y} \times \vec{Z}}{\left\| \vec{Y} \times \vec{Z} \right\|} = \begin{pmatrix} X_x \\ X_y \\ X_z \end{pmatrix}$$

where $\vec{X}$, $\vec{Y}$ and $\vec{Z}$ form an orthonormal basis of three dimensions $B' = \{\vec{X}, \vec{Y}, \vec{Z}\}$, and where $\vec{Y}$ corresponds to the natural vestibular nose-down pitch rotation, $\vec{X}$ corresponds to the natural vestibular right roll rotation and $\vec{Z}$ corresponds to the natural vestibular left yaw rotation.

SCDSReferenceFrame then transfers the vector set of the semicircular duct system, as well as the coordinates of the centers of rotation, from the scan frame of reference to the vestibular frame of reference by applying the following formula:

$$D'_{k,n} = P_{B'}^{B} D_{k,n}$$

where

$$P_{B'}^{B} = \frac{1}{\det \begin{bmatrix} X_x & Y_x & Z_x \\ X_y & Y_y & Z_y \\ X_z & Y_z & Z_z \end{bmatrix}} \begin{bmatrix} X_x & X_y & X_z \\ Y_x & Y_y & Y_z \\ Z_x & Z_y & Z_z \end{bmatrix}$$

corresponds to the passage matrix from B to B'; and where

$$D_k = \begin{pmatrix} D_{x,k} \\ D_{y,k} \\ D_{z,k} \end{pmatrix}$$

and

$$D'_k = \begin{pmatrix} D'_{X,k} \\ D'_{Y,k} \\ D'_{Z,k} \end{pmatrix}$$

correspond to the unit vector that are normal to the maximal response plane of the semicircular duct $_k$, or to the coordinates of its center of rotation, respectively in the scan and vestibular frames of reference.

Finally, **SCDSReferenceFrame** computes the angular relationships of the maximal response planes of the semicircular ducts with the three vestibular reference planes of the head, by applying the following formulae:

$$\vec{A}_n \wedge \overrightarrow{MidSag} = arc\cos(\vec{A}_n \cdot \vec{Y})$$

,

$$\vec{P}_n \wedge \overrightarrow{MidSag} = arc\cos(-\vec{P}_n \cdot \vec{Y})$$

and

$$\vec{L}_n \wedge \overrightarrow{MidSag} = arc\cos(-\vec{L}_n \cdot \vec{Y})$$

where $\vec{A}_n \wedge \overrightarrow{MidSag}$, $\vec{P}_n \wedge \overrightarrow{MidSag}$ and $\vec{L}_n \wedge \overrightarrow{MidSag}$ correspond respectively to the angles between the maximal response planes of the right (or left) anterior, posterior and lateral semicircular ducts and the mid-sagittal plane of the head, as defined in the vestibular frame of reference.

$$\vec{A}_r \wedge \overrightarrow{Cor} = arc\cos(\vec{A}_r \cdot \vec{X})$$

,

$$\vec{P}_r \wedge \overrightarrow{Cor} = arc\cos(\vec{P}_r \cdot \vec{X})$$

,

$$\vec{L}_r \wedge \overrightarrow{Cor} = arc\cos(\vec{L}_r \cdot \vec{X})$$

,

$$\vec{A}_l \wedge \overrightarrow{Cor} = arc\cos(-\vec{A}_l \cdot \vec{X})$$

,

$$\vec{P}_l \wedge \overrightarrow{Cor} = arc\cos(-\vec{P}_l \cdot \vec{X})$$

and

$$\vec{L}_l \wedge \overrightarrow{Cor} = arc\cos(-\vec{L}_l \cdot \vec{X})$$

where $\vec{A}_n \wedge \overrightarrow{Cor}$, $\vec{P}_n \wedge \overrightarrow{Cor}$ and $\vec{L}_n \wedge \overrightarrow{Cor}$ correspond respectively to the angles between the maximal response planes of the right (or left) anterior, posterior and lateral semicircular ducts and the coronal plane of the head, as defined in the vestibular frame of reference.

$$\vec{A}_r \wedge \overrightarrow{Hor} = arc\cos(-\vec{A}_r \cdot \vec{Z})$$

,

$$\vec{P}_r \wedge \overrightarrow{Hor} = arc\cos(\vec{P}_r \cdot \vec{Z})$$

,

$$\vec{L}_r \wedge \overrightarrow{Hor} = arc\cos(-\vec{L}_r \cdot \vec{Z})$$

,

$$\vec{A}_l \wedge \overrightarrow{Hor} = arc\cos(\vec{A}_l \cdot \vec{Z})$$

,

$$\vec{P}_l \wedge \overrightarrow{Hor} = arc\cos(-\vec{P}_l \cdot \vec{Z})$$

and

$$\vec{L}_l \wedge \overrightarrow{Hor} = arc\cos(\vec{L}_l \cdot \vec{Z})$$

where $\vec{A}_n \wedge \overrightarrow{Hor}$, $\vec{P}_n \wedge \overrightarrow{Hor}$ and $\vec{L}_n \wedge \overrightarrow{Hor}$ correspond respectively to the angles between the maximal response planes of the right (or left) anterior, posterior and lateral semicircular ducts and the horizontal plane of the head, as defined in the vestibular frame of reference.

At the end of the procedure, **SCDSReferenceFrame** output several files containing information on all computed metrics described above.

c. Input files

As input, **SCDSReferenceFrame** needs the files:

SPECIESNAME_R_Suffix.rawvector

with **Suffix** replaced by **Cor**, **MinusCor** and **PlusCor** in successive sets of files, to be placed in the folder **/AriadneToolbox/Data/SPECIESNAME/** (see sections I.iii.4. & I.iii.7.). Note that these input files are suppressed when **SCDSReferenceFrame** work is over.

It also potentially needs the file:

SPECIESNAME.midplane

to be placed in the folder **/AriadneToolbox/Data/SPECIESNAME/Matrix/** (see section I.vi. of the Ariadne Manual – Data preparation), if only the left or the right labyrinth has been segmented.

d. Output files

SCDSReferenceFrame outputs the files:

SPECIESNAME_Suffix.vector **SPECIESNAME_Suffix.synangle**

SPECIESNAME_R_Suffix.functionangle

in the folder "**/AriadneToolbox/Data/SPECIESNAME/**", with **Suffix** replaced by **Cor**, **MinusCor** and **PlusCor** in successive sets of files.

It also outputs the files:

SPECIESNAME_uncoupled.transmat **SPECIESNAME.symmat**

in the folder **/AriadneToolbox/Data/SPECIESNAME/Matrix/**.

.vector files contain information, based on a model of uncoupled ducts, about (1) the vector components of the maximal response plane of each semicircular duct, in the vestibular frame of reference, and (2) the center of rotation of each semicircular duct, in the vestibular frame of reference, each time with three levels of correction for potential shrinkage and segmentation errors.

.synangle files contain information, based on a model of uncoupled ducts, about (1) the synergistic angular relationships of the maximal response planes of the semicircular ducts, and (2) the variance of the synergistic angular relationships of the maximal response planes of the semicircular ducts from planarity (180var), each time with three levels of correction for potential shrinkage and segmentation errors.

.functionangle files contain information, based on a model of uncoupled ducts, about the angular relationships of the maximal response planes of the semicircular ducts, with the three vestibular reference planes of the head, each time with three levels of correction for potential shrinkage and segmentation errors.

.transmat and **.symmat** files correspond to square matrices that can be used by **DataVestibularReference** (section I.vi.1.) and **STLVestibularReference** (section I.vi.2.) to reflect data objects (linesets, landmarks, STLs) across the mid-sagittal plane of the head, and to place them in the vestibular frame of reference. The "**_uncoupled.transmat**" file outputted by **SCDSReferenceFrame** is used by **BiomechaParam** (section I.v.1.) to build the actual **.transmat** file that is used to transfer object in the vestibular frame of reference, based on a model of coupled ducts.

9. SCDSMorphometry

a. Description

SCDSMorphometry is an executable, written in C, which compiles morphological information provided by the modules **VolumeCalc** (section I.iii.1.), **SurfaceCalc** (section I.iii.2.), **MaximalResponse Axes** (section I.iii.4.), **DuctForm** (section I.iii.5.), **CupulaMorpho** (section I.iii.6.), **ShrinkCor** (section I.iii.7.) and **SCDSReferenceFrame** (section I.iii.8.) into few summary files, which are much easier to read and to handle than files initially outputted by these modules.

SCDSMorphometry can either be used as stand-alone software by double-clicking on **SCDSMorphometry.exe**, in the folder **/AriadneToolbox/**, and following instructions appearing in the console, or as part of the batch processes **Ariadne_SCDS.bat**, **Ariadne_SCDS_Lite.bat** or **MorphometryAuto.bat** (sections I.i & I.iii.).

b. Algorithm

SCDSMorphometry simply reads-in information contained in files outputted by above mentioned modules and appends morphological data into three summary files (**Cor**, **MinusCor** and **PlusCor**).

When both left and right membranous labyrinths are studied, and when applicable, averages between both sides are registered in output summary files.

c. Input files

As input, **SCDSMorphometry** needs the files:

SPECIESNAME_R_Suffix.volume

SPECIESNAME_R_Suffix.surface

SPECIESNAME_R_Suffix.length

SPECIESNAME_R_Suffix.earea

SPECIESNAME_R_Suffix.ipsiangle

SPECIESNAME_R_Suffix.carea

SPECIESNAME_R_Suffix.wallshape

SPECIESNAME_R_Suffix.cupula

SPECIESNAME_R_Suffix.functionangle

SPECIESNAME_Suffix.vector

SPECIESNAME_Suffix.synangle

with **Suffix** replaced by **Cor**, **MinusCor** and **PlusCor** in successive sets of files, to be placed in the folder **/AriadneToolbox/Data/SPECIESNAME/** (see sections II.iii.1., II.iii.2., II.iii.4., II.iii.5., II.iii.6., II.iii.7. & II.iii.8.). Note that these input files are suppressed when **SCDSMorphometry** work is over.

d. Output files

SCDSMorphometry outputs the files:

SPECIESNAME_Suffix.morph

in the folder **/AriadneToolbox/Data/SPECIESNAME/**, with **Suffix** successively replaced by **Cor**, **MinusCor** and **PlusCor**.

.morph files contain information about all morphological data derived from the modules **VolumeCalc**, **MaximalResponseAxes**, **DuctForm**, **CupulaMorpho** and **SCDSReferenceFrame**, with correction against shrinkage and manual segmentation effects.

iv. *FEAAuto*

FEAAuto is a batch file that automatically executes **ElmerPreparation** (section I.iv.1.) and **ElmerSolver** (section I.iv.2.), in that order. To use it, double-click it, in the folder **/AriadneToolbox/Batch/**, and answer the question that is asked.

FEAAuto is coded in such a way that it will seek most information needed by its modules by reading a **TaxaInfo.txt** file that has to be placed at the root of the **Data** folder (see section I.i., step (2), and by looking into the name of files and folders that are contained in that same folder. It is then essential, to use this module, to strictly follow the naming convention for input files as described in Ariadne Manual – Data preparation, for files pertaining to the **ElmerPreparation** and **ElmerSolver** modules, and to structure the **Data** folder accordingly.

It should be noted that **FEAAuto** is not limited to analyze only one specimen at a time but can read in every specimen found in **TaxaInfo.txt**. This makes analysis of multiple specimens possible as long as naming conventions are rigorously followed.

1. ElmerPreparation

a. Description

ElmerPreparation is an executable, written in C, which writes '.sif' instruction files for the module **ElmerSolver** (section I.iv.2.). These instruction files order **ElmerSolver** to (1) map the transverse displacement at the level of the middle cross section of each cupula, which follows from the application of a maintained homogeneous pressure onto their surface, and to (2) analyse the deflection of areas of the cupula where stereocilia/kinocilia with various lengths can be found (section I.v. of Ariadne Manual – Data preparation).

ElmerPreparation can either be used as stand-alone software by double-clicking on **ElmerPreparation.exe**, in the folder **/AriadneToolbox/**, and following instructions appearing in the console, or as part of the batch processes **Ariadne_SCDS.bat**, **Ariadne_SCDS_Lite.bat** or **FEAAuto.bat** (sections I.i & I.iv.).

b. Input parameters

To work, **ElmerPreparation** needs information on the Young modulus, the Poisson ratio and on the density of studied cupulae, as well as information about the pressure the user wants to apply on their surface during the simulation. **ElmerPreparation** also needs to know whether the user considers the cupula (1) to be clamped at the level of both the *crista ampullaris* and the walls of the ampulla, which is the current most common assumption[4,11], (2) to be clamped at the level of the *crista ampullaris* only and supported at the level of the walls of the ampulla, which is our favored assumption, or (3) to be clamped at the level of the *crista ampullaris* only and loose at the level of the walls of the ampulla, which was often proposed in past studies[12].

When using **ElmerPreparation** executable, users are prompted by the console to manually input all above mentioned parameters as they see fit. On the contrary, for the batch processes **Ariadne_SCDS.bat**, **Ariadne_SCDS_Lite.bat** or **FEAAuto.bat** (sections I.i & I.iv.), the user is only prompted by the console to answer the question about cupula mode of deflection. Depending on the answer, following values are directly inputted through the batch files for above mentioned parameters:

	Value
Young modulus (assumption (1)	3.26 Pa
Young modulus (assumption (2)	4.258 Pa
Young modulus (assumption (3)	36.03 Pa
Poisson ratio	0.48
Density of the cupula	1000 $kg.m^{-3}$
Pressure applied	0.05 Pa

Values inputted for Poisson ratio, the density of the cupula and the applied pressure follow Selva et al.[11]. Values inputted for the Young modulus, however, where empirically found by matching the average mechanical long time constant (section I.v.1.) computed from the FEA analysis of the cupulae of a Squirrel monkey specimen, to the non-adapted neurophysiological average long time constant of 5.73s that has been measured in the peripheral neurons of this same species[13], and which is supposed to reflect the mechanical one.

c. Instructions

ElmerPreparation writes following instructions in corresponding '.sif' files:

```
!!! CHECK BOUNDARIES LABELS !!!

$t = THICKNESSe-3 ! Input cupula thickness (Length cilia) in meter
$cs = UNIT ! 1e-6 for mesh in um, 1e-3 for mesh in mm, 1e-2 for
mesh in cm
$E = YOUNG ! Young modulus
$p = POISSON ! Poisson ratio
$D = DENSITY ! Density
$P = PRESSURE ! Pressure

CHECK KEYWORDS Warn

Header
  Mesh DB "." "xfea_SPECIESNAME_Duct_Cup"
End

Simulation
  Coordinate System = Cartesian 2D
  Simulation Type = Steady state
  Steady State Max Iterations = 1
  Output Intervals = 1
  Post File = "../xfea_SPECIESNAME_Duct_Cilia_Length.ep"
  Coordinate scaling = $cs
End

Body 1
  Target Bodies(1) = 2
  Name = "Cupula"
Target Bodies(1) = 3
  Name = "Long Cilia"
  Equation = 1
  Material = 2
  Body Force = 1
End

Body 3
  Target Bodies(1) = 4
  Name = "Medium Cilia"
  Equation = 1
  Material = 3
  Body Force = 1
End

Body 4
  Target Bodies(1) = 1
  Name = "Short Cilia"
  Equation = 1
  Material = 4
  Body Force = 1
End

Body Force 1
  Pressure = $P
End

Material 1
```

```
  Equation = 1
  Material = 1
  Body Force = 1
End

Body 2
  Medium Cilia = Real 0
  Short Cilia = Real 0
End

Material 2
  Name = "Long Cilia"
  Thickness = $t
  Poisson ratio = $p
  Youngs modulus = $E
  Density = $D
  Long Cilia = Real 1
  Medium Cilia = Real 0
  Short Cilia = Real 0
End

Material 3
  Name = "Medium Cilia"
  Thickness = $t
  Poisson ratio = $p
  Youngs modulus = $E
  Density = $D
  Long Cilia = Real 0
  Medium Cilia = Real 1
  Short Cilia = Real 0
End

Material 4
  Name = "Short Cilia"
  Thickness = $t
  Poisson ratio = $p
  Youngs modulus = $E
  Density = $D
  Long Cilia = Real 0
  Medium Cilia = Real 0
  Short Cilia = Real 1
End

Solver 1
  Equation = "SmitcSolver"
  Procedure = "Smitc" "SmitcSolver"
  Variable = -dofs 3 Deflection
  Exec Solver = Always
  Variable = Deflection
  Name = "Cupula"
  Thickness = $t
  Poisson ratio = $p
  Youngs modulus = $E
  Density = $D
  Long Cilia = Real 0
  Variable DOFs = 3
  Eigen Analysis = False
  Hole Correction = False
  Linear System Solver = Direct
  Linear System Direct Method = umfpack
End

Solver 2
  Equation = SaveScalars
  Procedure = "SaveData" "SaveScalars"
  Exec Solver = After Simulation
  Filename = "xfea_SPECIESNAME_Duct_Cilia_Length.deflection"
  Variable 1 = String Deflection 1
  Operator 1 = max
  Variable 2 = String Deflection 1
  Operator 2 = int mean
  Variable 3 = String Deflection 1
  Operator 3 = int
  Coefficient 3 = String Long Cilia
  Variable 4 = String Deflection 1
  Operator 4 = int
  Coefficient 4 = String Medium Cilia
  Variable 5 = String Deflection 1
  Operator 5 = int
  Coefficient 5 = String Short Cilia
  Variable 6 = String Deflection 1
  Operator 6 = volume
  Coefficient 6 = String Long Cilia
  Variable 7 = String Deflection 1
  Operator 7 = volume
  Coefficient 7 = String Medium Cilia
  Variable 8 = String Deflection 1
  Operator 8 = volume
  Coefficient 8 = String Short Cilia
  Variable 9 = String Deflection 1
  Operator 9 = volume
  Variable 10 = String Deflection 1
  Operator 10 = area
End

Equation 1
  Active Solvers(2) = 1 2
End
```

with boundary conditions for the first assumption on cupula mode of deflection corresponding to:

```
Boundary Condition 1
  Target Boundaries = CRISTALABELS
  Name = "Crista"
  Deflection 1 = 0
  Deflection 2 = 0
  Deflection 3 = 0
  Save Scalars = Logical True
End

Boundary Condition 2
  Target Boundaries = AMPULLALABELS
  Name = "Ampulla"
  Deflection 1 = 0
  Deflection 2 = 0
  Deflection 3 = 0
  Save Scalars = Logical True
End
```

boundary conditions for the second assumption on cupula mode of deflection corresponding to:

```
Boundary Condition 1
  Target Boundaries = CRISTALABELS
  Name = "Crista"
  Deflection 1 = 0
  Deflection 2 = 0
  Deflection 3 = 0
  Save Scalars = Logical True
End
```

```
Boundary Condition 2
  Target Boundaries = AMPULLALABELS
  Name = "Ampulla"
  Deflection 1 = 0
  Save Scalars = Logical True
End
```

boundary conditions for the third assumption on cupula mode of deflection corresponding to:

```
Boundary Condition 1
  Target Boundaries = CRISTALABELS
  Name = "Crista"
  Deflection 1 = 0
  Deflection 2 = 0
  Deflection 3 = 0
  Save Scalars = Logical True
End
```

```
Boundary Condition 2
  Target Boundaries = AMPULLALABELS
  Name = "Ampulla"
  Save Scalars = Logical True
End
```

and where **xfea** is replaced by **rfea** for a right labyrinth and **lfe**a for a left labyrinth; where **SPECIESNAME** is replaced by the name used for the studied specimen in every other sections; where **Duct** is either replaced by **Ant**, **Post** or **Lat** depending on which cupula is studied; where **Length** is either replaced by **Long**, **Medium** or **Short** depending on which assumption on stereocilia/kinocilia length is considered; where **THICKNESS** is replaced by the value as read in the **SPECIESNAME_Cor.morph** file (section I.iii.9.) for the corresponding assumption on stereocilia/kinocilia length; where **UNIT** is either replaced by '**1e-6**', '**1e-3**' or '**1e-2**' depending on what is manually inputted by the user, or on what is written in the **TaxaInfo.txt** file, for coordinate units (see section I.i., step (2); where **YOUNG**, **POISSON**, **DENSITY** and **PRESSURE** are respectively replaced by corresponding values described in section I.v.2.b.; and where **CRISTALABELS** and **AMPULLALABELS** are respectively replaced by labels of the boundaries of the *crista ampullaris* and of the walls of the ampulla of corresponding cupula, as read in the **xfea_SPECIESNAME_Boundaries.labels** file (section I.ii.3.).

These instructions order **ElmerSolver** (section I.iv.2.) to solve Reissner-Mindlin equations, which describe the shear deformation of thick plates, by representing the cupula domain as MITC-plate elements (see section I.v. of the Ariadne Manual – Data preparation), and by using boundary conditions that mimic the assumed cupula deflection mode. They theoretically lead **ElmerSolver** to find the exact solution of the pattern of deformation of the given cupula that follows the application of a maintained homogeneous pressure on its surface.

These instructions also order **ElmerSolver** to output files containing information pertaining to the visualization of the pattern of deformation of the given cupula (see section I.i.), as well as files containing information about deflection metrics at the level of the whole cupula, but also at the level of areas of the cupula where stereocilia/kinocilia with various lengths can be found.

d. Input files

As input, **ElmerPreparation** needs the file:

SPECIESNAME_Cor.morph

to be placed in the folder **/AriadneToolbox/Data/SPECIESNAME/** (see section I.iii.9.).

It also needs the file:

rfea_SPECIESNAME_boundaries.labels

to be placed in the folder **/AriadneToolbox/Data/SPECIESNAME/FEA/** (see section I.ii.3.).

e. Output files

ElmerPreparation outputs the files:

rfea_SPECIESNAME_Ant_Cilia_Long.sif

rfea_SPECIESNAME_Ant_Cilia_Medium.sif

rfea_SPECIESNAME_Ant_Cilia_Short.sif

rfea_SPECIESNAME_Post_Cilia_Long.sif

rfea_SPECIESNAME_Post_Cilia_Medium.sif

rfea_SPECIESNAME_Post_Cilia_Short.sif

rfea_SPECIESNAME_Lat_Cilia_Long.sif

rfea_SPECIESNAME_Lat_Cilia_Medium.sif

rfea_SPECIESNAME_Lat_Cilia_Short.sif

in the folder **/AriadneToolbox/Data/SPECIESNAME/FEA/**.

.sif files contain instructions for **ElmerSolver** (section I.iv.2.) to analyze and map the pattern of deflection of the given cupula after a maintained homogeneous pressure was applied on its surface.

2. ElmerSolver

a. Description

ElmerSolver is a software, developed by CSC – IT Center for Science (CSC), which has not been developed as part of Ariadne Toolbox, but which is nevertheless needed for it to be fully operational. **ElmerSolver** can be downloaded as part of **Elmer FEM** on the website https://www.csc.fi/web/elmer. As suggested by its name, **ElmerSolver** is the software that actually solves the partial differential equations that describe a given physical problem across the provided domain. It does so by strictly following instructions provided in '.sif' files generated by **ElmerPreparation** (section I.iv.1.). Inside Ariadne framework, **ElmerSolver** is used to:

- map the transverse displacement at the level of the middle cross section of each cupula, which follows from the application of a maintained homogeneous pressure on their surface
- analyze the deflection of areas of the cupula where stereocilia/kinocilia with various lengths can be found (section I.v. of the Ariadne Manual – Data preparation).

ElmerSolver can either be used as stand-alone software by following the procedure described below, or as part of the batch processes **Ariadne_SCDS.bat**, **Ariadne_SCDS_Lite.bat** or **FEAAuto.bat** (sections I.i & I.iv.).

b. Procedure

1.
    a. Open the folder **/AriadneToolbox/Data/SPECIESNAME/FEA/**.
    b. Right-click anywhere and select [New>Text Document].
    c. Name the file **ELMERSOLVER_STARTINFO**.
    d. Open **ELMERSOLVER_STARTINFO** with the any notepad software.
    e. In **ELMERSOLVER_STARTINFO**, on the first line, type in **rfea_SPECIESNAME_ Ant_Cilia_Long.sif**.
    f. Press the [Spacebar] key.
    g. In **ELMERSOLVER_STARTINFO**, type in '1'.
    h. Close **ELMERSOLVER_STARTINFO**.
2.
    a. Click the [Start] button of Windows.
    b. In the Search textbox, type 'cmd'. The Windows Command Prompt should open.
    c. In the Command Prompt, type 'cd user_computer_specific_path\AriadneToolbox\ Data\SPECIESNAME\FEA'.
    d. In the Command Prompt, type '**ElmerSolver**'.
    e. From **ElmerSolver** log, check that no message saying: "ERROR:: ElementMetric: Degenerate 2D element" popped up.
3.
    a. Open **ELMERSOLVER_STARTINFO** with any notepad software.
    b. In **ELMERSOLVER_STARTINFO**, replace **rfea_SPECIESNAME_Ant_Cilia_Long.sif** with **rfea_SPECIESNAME_Ant_Cilia_Medium.sif**.
    c. Close **ELMERSOLVER_STARTINFO**.
4.
    a. In the Command Prompt, type '**ElmerSolver**'.
    b. From **ElmerSolver** log, check that no message saying: "ERROR:: ElementMetric: Degenerate 2D element" popped up.
5.
    a. Open **ELMERSOLVER_STARTINFO** with any notepad software.
    b. In **ELMERSOLVER_STARTINFO**, replace **rfea_SPECIESNAME_Ant_Cilia_Medium.sif** with **rfea_SPECIESNAME_Ant_Cilia_Short.sif**.
    c. Close **ELMERSOLVER_STARTINFO**.
6.
    a. In the Command Prompt, type '**ElmerSolver**'.
    b. From **ElmerSolver** log, check that no message saying: "ERROR:: ElementMetric: Degenerate 2D element" popped up.
7.
    a. Open **ELMERSOLVER_STARTINFO** with any notepad software.
    b. In **ELMERSOLVER_STARTINFO**, replace **rfea_SPECIESNAME_Ant_Cilia_Short.sif** with **rfea_SPECIESNAME_Post_Cilia_Long.sif**.
    c. Close **ELMERSOLVER_STARTINFO**.
8.
    a. In the Command Prompt, type '**ElmerSolver**'.

b. From **ElmerSolver** log, check that no message saying: ”ERROR:: ElementMetric: Degenerate 2D element” popped up.

9.
   a. Open **ELMERSOLVER_STARTINFO** with any notepad software.
   b. In **ELMERSOLVER_STARTINFO**, replace **rfea_SPECIESNAME_Post_Cilia_Long.sif** with **rfea_SPECIESNAME_Post_Cilia_Medium.sif**.
   c. Close **ELMERSOLVER_STARTINFO**.

10.
   a. In the Command Prompt, type ‘**ElmerSolver**’.
   b. From **ElmerSolver** log, check that no message saying: ”ERROR:: ElementMetric: Degenerate 2D element” popped up.

11.
   a. Open **ELMERSOLVER_STARTINFO** with any notepad software.
   b. In **ELMERSOLVER_STARTINFO**, replace **rfea_SPECIESNAME_Post_Cilia_Medium.sif** with **rfea_SPECIESNAME_Post_Cilia_Short.sif**.
   c. Close **ELMERSOLVER_STARTINFO**.

12.
   a. In the Command Prompt, type ‘**ElmerSolver**’.
   b. From **ElmerSolver** log, check that no message saying: ”ERROR:: ElementMetric: Degenerate 2D element” popped up.

13.
   a. Open **ELMERSOLVER_STARTINFO** with any notepad software.
   b. In **ELMERSOLVER_STARTINFO**, replace **rfea_SPECIESNAME_Post_Cilia_Short.sif** with **rfea_SPECIESNAME_Lat_Cilia_Long.sif**.
   c. Close **ELMERSOLVER_STARTINFO**.

14.
   a. In the Command Prompt, type ‘**ElmerSolver**’.
   b. From **ElmerSolver** log, check that no message saying: ”ERROR:: ElementMetric: Degenerate 2D element” popped up.

15.
   a. Open **ELMERSOLVER_STARTINFO** with any notepad software.
   b. In **ELMERSOLVER_STARTINFO**, replace **rfea_SPECIESNAME_Lat_Cilia_Long.sif** with **rfea_SPECIESNAME_Lat_Cilia_Medium.sif**.
   c. Close **ELMERSOLVER_STARTINFO**.

16.
   a. In the Command Prompt, type ‘**ElmerSolver**’.
   b. From **ElmerSolver** log, check that no message saying: ”ERROR:: ElementMetric: Degenerate 2D element” popped up

17.
   a. Open **ELMERSOLVER_STARTINFO** with any notepad software.
   b. In **ELMERSOLVER_STARTINFO**, replace **rfea_SPECIESNAME_Lat_Cilia_Medium.sif** with **rfea_SPECIESNAME_Lat_Cilia_Short.sif**.
   c. Close **ELMERSOLVER_STARTINFO**.

18.
   a. In the Command Prompt, type ‘**ElmerSolver**'.

b. From **ElmerSolver** log, check that no message saying: ”ERROR:: ElementMetric: Degenerate 2D element” popped up.

This full procedure allows **ElmerSolver** to compute the patterns of deformation of given cupulae that follow the application of a maintained homogeneous pressure on their surface. It also allows **ElmerSolver** to output files containing information pertaining to the visualization of these patterns of deformation (see section I.i.), as well as files containing information about deflection metrics at the level of the whole cupulae, but also at the level of areas of the cupulae where stereocilia/kinocilia with various lengths can be found. These last metrics can be further processed by **BiomechaParam** (section I.v.1.) in order to compute stiffness parameters of the cupulae, as well as mechanical sensitivities of the semicircular ducts, expressed in terms of deflection of areas of the cupulae where stereocilia/kinocilia with various lengths can be found.

Note: It can sometimes happen that error messages pop up during the **ElmerSolver** procedure. This is linked to the inability of current algorithms implemented into **GMSH** to triangulate meshes from STL files containing very poor triangles (high aspect ratios, intersections). If that happens, redo the preparation of the FEA model of the problematic cupula(e) by making sure that all steps presented in section I.v. of the Ariadne Manual – Data preparation, particularly the steps 7-13, were rigorously followed. Increasing the number of triangles in step 17 of section I.v. of the Ariadne Manual – Data preparation can also potentially help.

c. Input files

As input, **ElmerSolver** needs the files:

rfea_SPECIESNAME_Ant_Cilia_Long.sif

rfea_SPECIESNAME_Ant_Cilia_Medium.sif

rfea_SPECIESNAME_Ant_Cilia_Short.sif

rfea_SPECIESNAME_Post_Cilia_Long.sif

rfea_SPECIESNAME_Post_Cilia_Medium.sif

rfea_SPECIESNAME_Post_Cilia_Short.sif

rfea_SPECIESNAME_Lat_Cilia_Long.sif

rfea_SPECIESNAME_Lat_Cilia_Medium.sif

rfea_SPECIESNAME_Lat_Cilia_Short.sif

ELMERSOLVER_STARTINFO

to be placed in the folder **/AriadneToolbox/Data/SPECIESNAME/FEA/** (see sections I.iv.1. & I.iv.2.b.).

It also needs the folders:

rfea_SPECIESNAME_Ant_Cup

rfea_SPECIESNAME_Lat_Cup

rfea_SPECIESNAME_Post_Cup

containing the files:

mesh.boundary

mesh.header

mesh.elments

mesh.nodes

to be placed in the folder **/AriadneToolbox/Data/SPECIESNAME/FEA/** (see section I.ii.3.).

d. Output files

ElmerSolver procedure leads to output the files:

rfea_SPECIESNAME_Ant_Cilia_Long.ep

rfea_SPECIESNAME_Ant_Cilia_Medium.ep

rfea_SPECIESNAME_Ant_Cilia_Short.ep

rfea_SPECIESNAME_Post_Cilia_Long.ep

rfea_SPECIESNAME_Post_Cilia_Medium.ep

rfea_SPECIESNAME_Post_Cilia_Short.ep

rfea_SPECIESNAME_Lat_Cilia_Long.ep

rfea_SPECIESNAME_Lat_Cilia_Medium.ep

rfea_SPECIESNAME_Lat_Cilia_Short.ep

rfea_SPECIESNAME_Ant_Cilia_Long.deflection

rfea_SPECIESNAME_Ant_Cilia_Medium.deflection

rfea_SPECIESNAME_Ant_Cilia_Short.deflection

rfea_SPECIESNAME_Post_Cilia_Long.deflection

rfea_SPECIESNAME_Post_Cilia_Medium.deflection

rfea_SPECIESNAME_Post_Cilia_Short.deflection

rfea_SPECIESNAME_Lat_Cilia_Long.deflection

rfea_SPECIESNAME_Lat_Cilia_Medium.deflection

rfea_SPECIESNAME_Lat_Cilia_Short.deflection

in the folder **/AriadneToolbox/Data/SPECIESNAME/FEA/**.

.**ep** files contain information that are needed by **ElmerPost** to allow visualizing the deflection patterns of corresponding cupulae (see tutorial in section I.i. and accompanying Fig.3).

.**deflection** files contain information about deflection metrics at the whole cupulae level, as well as at the level of areas of the cupulae where stereocilia/kinocilia with various lengths can be found. These metrics can be further processed by **BiomechaParam** (section I.v.1.) in order to compute stiffness parameters of the cupulae, as well as the mechanical sensitivity of the semicircular ducts, expressed in terms of deflection of areas of the cupulae where stereocilia/kinocilia with various lengths can be found.

v. BiomechanicsAuto

BiomechanicsAuto is a batch file that automatically executes **BiomechaParam** (section I.v.1.), **BodePlotsGenerator** (section I.v.2.), and **SensitivityMapping** (section I.v.3.), in that order. To use it, double-click it, in the folder **/AriadneToolbox/Batch/**, and press any key.

BiomechanicsAuto is coded in such a way that it will seek most information needed by its modules by reading a **TaxaInfo.txt** file that has to be placed at the root of the **Data** folder (see section I.i., step (2), and by looking into the name of files and folders contained in that same folder. It is then essential, to use this module, to strictly follow the naming convention for input files as described in Ariadne Manual – Data preparation, for files pertaining to the **BiomechaParam**, **BodePlotsGenerator**, and **SensitivityMapping** modules, and to structure the **Data** folder accordingly.

It should be noted that **BiomechanicsAuto** is not limited to analyze only one specimen at a time but can read in every specimen found in **TaxaInfo.txt**. This makes analysis of multiple specimens possible as long as naming conventions are rigorously followed.

1. BiomechaParam

a. Description

BiomechaParam is an executable, written in C, which computes the viscosity of both the endolymph and the cupulae from body temperature, following a modified version of the empirical law of Ten Kate and Kuiper[7].

It also computes, based on a model of coupled ducts, each time with three levels of correction for potential shrinkage and segmentation errors, and with three different assumptions on stereocilia/kinocilia length, when applicable:

- the vector components of the maximal response plane of each semicircular duct, in the scan frame of reference (uncoupled values are also outputted),
- the center of rotation of each semicircular duct, in the scan frame of reference
- the vector components of the prime direction of each semicircular duct, in the vestibular frame of reference
- the ipsilateral angular relationships of the maximal response planes of the semicircular ducts
- the synergistic angular relationships of the maximal response planes of the semicircular ducts
- the angular relationships of the maximal response planes of the semicircular ducts, with the three vestibular reference planes of the head
- the variance of the ipsilateral angular relationships of the maximal response planes of the semicircular ducts from orthogonality ($90var^2$)
- the average deviation of the ipsilateral angular relationships of the maximal response planes of the semicircular ducts from orthogonality ($90dev^3$)
- the variance of the synergistic angular relationships of the maximal response planes of the semicircular ducts from planarity (180var)
- the average deviation of the synergistic angular relationships of the maximal response planes of the semicircular ducts from planarity (180dev).

- the displacement profile factor of each cupula
- the deflection of the stereocilia/kinocilia areas of each cupula
- the mass parameter of each part of the semicircular duct system
- the damping parameter of each part of the semicircular duct system
- the stiffness parameter of each cupula
- the inertial forcing parameter of each semicircular duct torus
- the long time constant of each semicircular duct (uncoupled values are also outputted)
- the short time constant of each semicircular duct (uncoupled values are also outputted)
- the lower corner frequency of each semicircular duct (uncoupled values are also outputted)
- the upper corner frequency of each semicircular duct (uncoupled values are also outputted)
- the natural frequency of each semicircular duct (only uncoupled values are outputted)
- the fractional bandwidth of the middle frequency range of each semicircular duct below and above their natural frequency (only uncoupled values are outputted)
- the in-plane mechanical sensitivity of each semicircular duct, relative to angular rotation (uncoupled values are also outputted)
- the in-plane mechanical velocity gain of each semicircular duct, relative to angular velocity (uncoupled values are also outputted)
- the in-plane mechanical acceleration gain of each semicircular duct, relative to angular acceleration).

The latter three parameters are expressed in units of cupula volumic displacement, cupula average linear displacement and cilia average deflection.

BiomechaParam also output 'matrix' files that can be used by **DataVestibularReference** (section I.vi.1.) and **STLVestibularReference** (section I.vi.2.) to place objects in the vestibular frame of reference, as well as 'lineset' files that can be opened in Avizo, and which graphically represent the excitation direction of the maximal response axis of each semicircular duct, following the right hand rule.

BiomechaParam can either be used as stand-alone software by double-clicking on **BiomechaParam.exe**, in the folder **/AriadneToolbox/**, and following instructions appearing in the console, or as part of the batch processes **Ariadne_SCDS.bat**, **Ariadne_SCDS_Lite.bat** or **BiomechanicsAuto.bat** (sections I.i & I.v.).

Note: **BiomechaParam** was partially built using the GNU Scientific Library for eigenvalues computation.

b. Input parameters

To work, **BiomechaParam** needs information on the body temperature of the studied organism, on the density of both its endolymph and cupulae, as well as on the endolymph viscosity factor that will be used to compute endolymph viscosity.

When using **BiomechaParam** executable, users are prompted by the console to manually input all above mentioned parameters as they see fit. On the contrary, for the batch processes **Ariadne_SCDS.bat**, **Ariadne_SCDS_Lite.bat** or **BiomechanicsAuto.bat** (sections I.i & I.v.), following values are directly inputted through the batch files for above mentioned parameters:

	Value
Body temperature	See TaxaInfo.txt
Endolymph viscosity factor	$2.85e^{-5}$ Pa.s
Density of the endolymph	1000 kg.m^{-3}
Density of the cupula	1000 kg.m^{-3}

Values inputted for the density of the endolymph and of the cupulae follow Rabbitt[9]. The value inputted for the endolymph viscosity factor is based on the value of 0.00085 Pa.s at 35°c for endolymph viscosity[4]. Values inputted for the body temperature, however, are read from the **TaxaInfo.txt** file (see section I.i., step (2).

c. Algorithm

In order to compute all above mentioned metrics, **BiomechaParam** first compute the viscosity of both the endolymph and the cupulae, by applying the following formulae:

$$\mu_{e,T} = \mu_e 10^{\left(\frac{247.8}{T+133.15}\right)}$$

and

$$\mu_{c,T} = 10\mu_{e,T}$$

where $\mu_{e,T}$ and $\mu_{c,T}$ respectively correspond to the viscosity of the endolymph and of the cupulae; where μ_e corresponds to the viscosity factor of the endolymph; and where T corresponds to the body temperature of the studied organism.

In these formulae, endolymph viscosity is computed from body temperature following a modified version of the empirical law of Ten Kate and Kuiper[7] In this version, the relationship between endolymph viscosity and temperature follow a pattern similar to that of water, but with a different viscosity factor. The viscosity of the cupulae is assumed to be ten times higher than the value retrieved for endolymph viscosity, based on empirical observation from the literature[9].

Next, **BiomechaParam** reads-in files containing information about semicircular ducts morphometrics (section I.iii.9.), as well as files containing information about cupulae deflection patterns (section I.iv.2.). From this, it computes the structural parameters of the torsion pendulum equation that governs semicircular ducts biomechanics, mostly by following the work of Oman, Rabbitt and Damiano[4,8,9]. Thus, it starts by computing the mass parameter of each semicircular duct part, by applying the following formulae:

$$m_{s,k} = \rho_e \frac{L_{s,k}}{a_{s,k}}$$

,

$$m_{cc/sc,k} = \rho_e \frac{L_{cc/sc,k}}{a_{cc/sc,k}}$$

,

$$m_{u,k} = \rho_e \frac{L_{u,k}}{a_{u,k}}$$

,

$$m_{a,k,h} = \rho_e \frac{L_{a,k} - t_{c,k,h}}{a_{a,k}}$$

,

$$m_{c,k,h} = \rho_c \frac{t_{c,k,h}}{a_{c,k}}$$

and

$$m_{k,h} = m_{s,k} + m_{a,k,h} + m_{c,k,h} + m_{u,k} + m_{cc/sc,k}$$

where $m_{s,k}$,and $m_{u,k}$ respectively correspond to the mass parameter of the slender $_s$ and utricular $_u$ parts of the semicircular duct $_k$; $m_{cc/sc,k}$ corresponds either to the mass parameter of the common crus part $_{cc}$ of the anterior and posterior semicircular ducts, or to the mass parameter of the simple crus part $_{sc}$ of the lateral semicircular duct; $m_{a,k,h}$,and $m_{c,k,h}$ respectively correspond to the mass parameter of the ampullar $_a$ and cupula $_c$ parts of the semicircular duct $_k$, assuming a length $_h$ for the longest stereocilia/kinocilia; and $m_{k,h}$ corresponds to the mass parameter of the semicircular duct $_k$; where $L_{s,k}$, $L_{u,k}$, $L_{cc/sc,k}$ and $L_{a,k}$ correspond to the length of the central streamline of respective parts of the semicircular duct $_k$; where $t_{c,k,h}$ corresponds to the mean thickness of the cupula of the semicircular duct $_k$; where $a_{s,k}$, $a_{u,k}$, $a_{cc/sc,k}$, $a_{a,k}$ and $a_{c,k}$ correspond to the mean cross-sectional area of respective parts of the semicircular duct $_k$; and where ρ_e and ρ_c respectively correspond to the density of the endolymph and of the cupulae.

These parameters are used, based on a model of coupled ducts, to build the matrix:

$$M_h = \begin{bmatrix} M_{1,h} & m_{12} & 0 \\ m_{12} & M_{2,h} & m_{23} \\ 0 & m_{23} & M_{3,h} \end{bmatrix}$$

where $M_{1,h}$ corresponds to the mass parameter of the lateral semicircular duct; $M_{2,h}$ corresponds to the mass parameter of the anterior semicircular duct; and $M_{3,h}$ corresponds to the mass parameter of the posterior semicircular duct; where m_{12} corresponds to the mass parameter of the part of the anterior utricle that is shared between the anterior and lateral semicircular ducts; and where m_{23} corresponds to the mass parameter of the part of the common crus that is shared between the anterior and posterior semicircular ducts.

Then, in order to compute the damping parameter of each semicircular duct part, **BiomechaParam** applies the following formulae:

$$c_{s,k} = \mu_e \frac{\lambda_{\mu,s,k} L_{s,k}}{a_{s,k}^2}$$

,

$$c_{cc/sc,k} = \mu_e \frac{\lambda_{\mu,cc/sc,k} L_{cc/sc,k}}{a_{cc/sc,k}^2}$$

,

$$c_{u,k} = \mu_e \frac{\lambda_{\mu,u,k} L_{u,k}}{a_{u,k}^2}$$

,

$$c_{a,k,h} = \mu_e \frac{\lambda_{\mu,a,k} \left(L_{a,k} - t_{c,k,h} \right)}{a_{a,k}^2}$$

,

$$c_{c,k,h} = \mu_e \frac{\lambda_{\mu,c,k} t_{u,k,h}}{a_{c,k}^2}$$

and

$$c_{k,h} = c_{s,k} + c_{a,k,h} + c_{c,k,h} + c_{u,k} + c_{cc/ul,k}$$

where $c_{s,k}$, $c_{u,k}$, $c_{cc/sc,k}$, $c_{a,k,h}$ and $c_{c,k,h}$ correspond to the damping parameter of respective parts of the semicircular duct $_k$; and $c_{k,h}$ corresponds to the damping parameter of the semicircular duct $_k$; and where $\lambda_{\mu,s,k}$, $\lambda_{\mu,u,k}$, $\lambda_{\mu,cc/sc,k}$, $\lambda_{\mu,a,k}$ and $\lambda_{\mu,c,k}$ correspond to the mean wall shape drag factor of respective parts of the semicircular duct $_k$.

These parameters are used, based on a model of coupled ducts, to build the matrix:

$$C_h = \begin{bmatrix} C_{1,h} & c_{12} & 0 \\ c_{12} & C_{2,h} & c_{23} \\ 0 & c_{23} & C_{3,h} \end{bmatrix}$$

where $C_{1,h}$ corresponds to the damping parameter of the lateral semicircular duct; $C_{2,h}$ corresponds to the damping parameter of the anterior semicircular duct; and $C_{3,h}$ corresponds to the damping parameter of the posterior semicircular duct; where c_{12} corresponds to the damping parameter of the part of the anterior utricle that is shared between the anterior and lateral semicircular ducts; and where c_{23} corresponds to the damping parameter of the part of the common crus that is shared between the anterior and posterior semicircular ducts.

Next, the stiffness parameter of each cupula is computed by applying the following formula:

$$k_{c,k,h} = \frac{P}{a_{c,k} u_{c,k,h}}$$

where $k_{c,k,h}$ corresponds to the stiffness parameter of the cupula of the semicircular duct $_k$; where $u_{c,k,h}$ corresponds to the mean transversal displacement of the middle cross-section of the cupula of the semicircular duct $_k$; and where P corresponds to an homogeneous pressure of 0.05 Pa, applied on the surface of each cupula.

These parameters are used, based on a model of coupled ducts, to build the matrix:

$$K_h = \begin{bmatrix} K_{1,h} & 0 & 0 \\ 0 & K_{2,h} & 0 \\ 0 & 0 & K_{3,h} \end{bmatrix}$$

where $K_{1,h}$ corresponds to the stiffness parameter of the lateral cupula; $K_{2,h}$ corresponds to the stiffness parameter of the anterior cupula; and $K_{3,h}$ corresponds to the stiffness parameter of the posterior cupula.

Finally, **BiomechaParam** computes the inertial forcing parameter of each semicircular duct torus by applying the following formula:

$$g_k = 2\rho_e \Lambda_k$$

where Λ_k corresponds to the area enclosed by the central streamline of the semicircular duct $_k$, as projected on its maximal response plane.

These parameters are used to construct the vectors:

$$\overrightarrow{G_a} = g_a \cdot {}_u\vec{u}_a$$

,

$$\overrightarrow{G_p} = g_p \cdot {}_u\vec{u}_p$$

and

$$\overrightarrow{G_l} = g_l \cdot - {}_u\vec{u}_l$$

where ${}_u\vec{u}_a$, ${}_u\vec{u}_p$ and ${}_u\vec{u}_l$ respectively correspond to unit vectors that are normal to the maximal response plane of the anterior $_a$, posterior $_p$ and lateral $_l$ semicircular ducts, based on a model of uncoupled ducts, and are oriented along their excitation direction, following the right hand rule.

From these parameters, **BiomechaParam** computes, based on a model of coupled ducts, the temporal parameters that govern the behavior of semicircular duct system over the physiological frequency range of head motion, mostly by following the work of Oman, Rabbitt and Damiano[4,8,9]. Thus, it starts by computing the six time constants $\tau_{n,k,h}$ of the semicircular duct system by finding the reciprocal opposites of the real part of the eigenvalues of the 6x6 matrix (see the Theoretical Appendix[1]):

$$A_h = \begin{bmatrix} -M_h^{-1}C_h & -M_h^{-1}K_h \\ 1 & 0 \end{bmatrix}$$

where

$$M_h^{-1} = \begin{bmatrix} M'_{1,h} & m'_{12,h} & m'_{13,h} \\ m'_{21,h} & M'_{2,h} & m'_{23,h} \\ m'_{31,h} & m'_{32,h} & M'_{3,h} \end{bmatrix}$$

It also computes the long and short time constants of each semicircular duct, based on a model of uncoupled ducts, by applying the following formulae:

$$\tilde{\tau}_{1,k,h} = \frac{c_{k,h}}{k_{c,k,h}}$$

and

$$\tilde{\tau}_{2,k,h} = \frac{m_{k,h}}{c_{k,h}}$$

where $\tilde{\tau}_{1,k,h}$ and $\tilde{\tau}_{2,k,h}$ respectively correspond to the long and short time constants of the semicircular duct k .

From these results, **BiomechaParam** then computes the six eigen frequencies of the semicircular duct system, by applying the following formula:

$$\omega_{n,k,h} = \frac{1}{2\pi\tau_{n,k,h}}$$

where $\omega_{n,k,h}$ corresponds to the n-th eigen frequency of the semicircular duct system; and where $\tau_{n,k,h}$ corresponds to the n-th time constant of the semicircular duct system.

It also computes, based on a model of uncoupled ducts, the lower and upper corner frequencies of each semicircular duct, by applying the following formulae:

$$\tilde{\omega}_{1,k,h} = \frac{1}{2\pi\tilde{\tau}_{1,k,h}}$$

and

$$\tilde{\omega}_{2,k,h} = \frac{1}{2\pi\tilde{\tau}_{2,k,h}}$$

where $\omega_{1,k,h}$ and $\omega_{2,k,h}$ respectively correspond to the lower and upper corner frequencies of the semicircular duct k .

Next, **BiomechaParam** computes, based on a model of uncoupled ducts, the natural frequency of each semicircular duct, i.e. the head motion frequency for which semicircular duct's response is totally in phase with angular velocity, by applying the following formula:

$$\tilde{\omega}_{0,k,h} = \frac{1}{2\pi\sqrt{\tilde{\tau}_{1,k,h}\tilde{\tau}_{2,k,h}}}$$

where $\tilde{\omega}_{0,k,h}$ correspond to the natural frequency of the semicircular duct $_k$.

Finally, **BiomechaParam** computes, based on a model of uncoupled ducts, the fractional bandwidth of the middle frequency range of each semicircular duct, i.e. the logarithmically equivalent extension of the frequency bandwidth between the lower corner and natural frequencies of a semicircular duct, by applying the following formula:

$$\tilde{\sigma}_{k,h} = \frac{\tilde{\omega}_{0,k,h}}{\tilde{\omega}_{1,k,h}}$$

where $\tilde{\sigma}_{k,h}$ correspond to the fractional bandwidth of the middle frequency range of the semicircular duct $_k$.

Aside from temporal parameters *per se*, the in-plane mechanical sensitivity of each semicircular duct corresponds to the last major parameter that constrains their biomechanics over the physiological frequency range of head motion. Based on the work of Oman, Rabbitt and Damiano[4,8,9], **BiomechaParam** then computes, based on a model of coupled ducts, the in-plane mechanical sensitivity of each semicircular duct, by applying the following formulae:

$$\overrightarrow{X}_{x=e,k=a,h} = M'_{2,h}\overrightarrow{G_a} + m'_{23,h}\overrightarrow{G_p} + m'_{21,h}\overrightarrow{G_l}$$

,

$$\overrightarrow{X}_{x=e,k=p,h} = M'_{3,h}\overrightarrow{G_p} + m'_{32,h}\overrightarrow{G_a} + m'_{31,h}\overrightarrow{G_l}$$

,

$$\overrightarrow{X}_{x=e,k=l,h} = -M'_{1,h}\overrightarrow{G_l} - m'_{12,h}\overrightarrow{G_a} - m'_{13,h}\overrightarrow{G_p}$$

and

$$X_{x=e,k,h} = \left\|\overrightarrow{X}_{x=e,k,h}\right\|$$

and

$$X_{x=c,k,h} = \frac{X_{x=e,k,h}}{a_{c,k}}$$

and

$$\vec{u}_{k,h} = \frac{\overrightarrow{X}_{x=e,k,h}}{X_{x=e,k,h}}$$

where $X_{x=e,k,h}$ corresponds to the in-plane mechanical sensitivity of the semicircular duct $_k$, expressed in terms of total volume displacement of the cupula $_{x=e}$ [4,9]; where $X_{x=c,k,h}$ corresponds to the in-plane mechanical sensitivity of the semicircular duct $_k$, expressed in terms of average transverse displacement of the cupula $_{x=c}$ [8]; and where $\vec{u}_{k,h}$ corresponds to the unit vector that is normal to the maximal response plane of the semicircular duct $_k$, based on a model of coupled ducts, and is oriented along its excitation direction, following the right hand rule.

BiomechaParam also computes, based on a model of uncoupled ducts, the in-plane mechanical sensitivity of each semicircular duct, by applying the following formulae:

$$\tilde{X}_{x=e,k,h} = \frac{g_k}{m_{k,h}}$$

and

$$\tilde{X}_{x=c,k,h} = \frac{g_k}{a_{c,k} m_{k,h}}$$

where $\tilde{X}_{x=e,k,h}$ corresponds to the in-plane mechanical sensitivity of the semicircular duct $_k$, expressed in terms of total volume displacement of the cupula $_{x=e}$ [4,9]; and where $\tilde{X}_{x=c,k,h}$ corresponds to the in-plane mechanical sensitivity of the semicircular duct $_k$, expressed in terms of average transverse displacement of the cupula $_{x=c}$ [8].

Opposite to the above two metrics, respectively supported by Rabbitt and colleagues[4] and Oman and colleagues[8], we propose to use the average deflection of areas of the cupulae where stereocilia/kinocilia can be found as the main metrics for in-plane mechanical sensitivity of semicircular ducts (see sections I.v. of the Ariadne Manual – Data preparation & section I.iv.2. of this manual). As "shear strain between adjacent hair bundles […] is the key mechanical quantity leading to opening of transduction channels" (Ref 4, p.178), and then to the modulation of afferent fibers firing rate, we strongly think that this new metrics is the most precise biomechanical proxy, to date, for estimating semicircular duct sensitivity.

As expected, **BiomechaParam** implement this new metrics. However, to be able to compute it, it first needs to apply the following formulae, in order to compute the mean deflection of areas of the cupula where stereocilia/kinocilia with various lengths can be found, taking the three assumptions on stereocilia/kinocilia length into account (section I.v. of the Ariadne Manual – Data preparation):

$$d_{30,k,h=90} = \frac{u_{30,k,h=90}}{15 a_{c,k} u_{c,k,h=90}}$$

,

$$d_{60,k,h=90} = \frac{u_{60,k,h=90}}{45 a_{c,k} u_{c,k,h=90}}$$

,

$$d_{90,k,h=90} = \frac{u_{90,k,h=90}}{75 a_{c,k} u_{c,k,h=90}}$$

and

$$d_{30,k,h=60} = \frac{u_{30,k,h=60}}{15 a_{c,k} u_{c,k,h=60}}$$

,

$$d_{60,k,h=60} = \frac{u_{60,k,h=60}}{45 a_{c,k} u_{c,k,h=60}}$$

and

$$d_{30,k,h=30} = \frac{u_{30,k,h=30}}{15 a_{c,k} u_{c,k,h=30}}$$

where $u_{30,k,h=90}$, $u_{30,k,h=60}$, $u_{30,k,h=30}$, $u_{60,k,h=90}$, $u_{60,k,h=60}$ and $u_{90,k,h=90}$ correspond to assumed mean transversal displacements of respective areas of the cupula of the semicircular duct $_k$, after the application of an homogeneous pressure of 0.05 Pa on the surface of the cupula, and under the long $_{h=90}$, medium $_{h=60}$ and short $_{h=30}$ stereocilia/kinocilia assumptions; where $u_{c,k,h=30}$, $u_{c,k,h=60}$ and $u_{c,k,h=90}$ respectively correspond to the mean transversal displacement the middle cross-section of the cupula of the semicircular duct $_k$ under the long $_{h=90}$, medium $_{h=60}$ and short $_{h=30}$ stereocilia/kinocilia assumptions; and where $d_{30,k,h=90}$, $d_{30,k,h=60}$, $d_{30,k,h=30}$, $d_{60,k,h=90}$, $d_{60,k,h=60}$ and $d_{90,k,h=90}$ correspond to assumed mean deflections of respective areas of the cupula of the semicircular duct $_k$.

Note that in the above formulae, the mean transversal displacement of areas of the k-th cupula that are situated between 0 and 30 µm, between 30 and 60 µm and between 60 and 90 µm from the *crista ampullaris* are respectively divided by 15 µm, 45 µm and 75 µm in order to compute the average deflection (not displacement) of the stereocilia/kinocilia that run from the floor of the *crista ampullaris* and up to these areas of the subcupular space.

Then, **BiomechaParam** computes, based on a model of coupled ducts, the in-plane mechanical sensitivity of each semicircular duct, expressed in terms of average deflection of areas of the cupulae where stereocilia/kinocilia can be found, by applying the following formulae:

$$X_{x=s,k,h=90} = \frac{X_{x=e,k,h=90} \cdot \left(d_{90,k,h=90} + d_{60,k,h=90} + d_{30,k,h=90}\right)}{3}$$

,

$$X_{x=s,k,h=60} = \frac{X_{x=e,k,h=60} \cdot \left(d_{60,k,h=60} + d_{30,k,h=60}\right)}{2}$$

and

$$X_{x=s,k,h=30} = X_{x=e,k,h=30} . d_{30,k,h=30}$$

where $X_{x=s,k,h=30}$, $X_{x=s,k,h=60}$ and $X_{x=s,k,h=90}$ correspond to assumed in-plane mechanical sensitivities of the semicircular duct $_k$, expressed in terms of average deflection of areas of the cupulae where stereocilia/kinocilia can be found $_{x=s}$.

BiomechaParam also computes, based on a model of uncoupled ducts, the in-plane mechanical sensitivity of each semicircular duct, expressed in terms of average deflection of areas of the cupulae where stereocilia/kinocilia can be found, by applying the following formulae:

$$\tilde{X}_{x=s,k,h=90} = \frac{g_k\left(d_{90,k,h=90} + d_{60,k,h=90} + d_{30,k,h=90}\right)}{3.m_{k,h=90}}$$

,

$$\tilde{X}_{x=s,k,h=60} = \frac{g_k\left(d_{60,k,h=60} + d_{30,k,h=60}\right)}{2.m_{k,h=60}}$$

and

$$\tilde{X}_{x=s,k,h=30} = \frac{g_k d_{30,k,h=30}}{m_{k,h=30}}$$

where $\tilde{X}_{x=s,k,h=30}$, $\tilde{X}_{x=s,k,h=60}$ and $\tilde{X}_{x=s,k,h=90}$ correspond to assumed in-plane mechanical sensitivities of the semicircular duct $_k$, expressed in terms of average deflection of areas of the cupulae where stereocilia/kinocilia can be found $_{x=s}$.

Technical note: BiomechaParam corrects the deflection parameters d for potential segmentation errors. To do so, it implements the following regressions that where retrieved via the study of 37 mammal specimens:

$$d_{90,k,h,xCor} = 10^{(-1.59936\log_{10} a_{c,k,xCor} + 0.18422\log_{10} t_{c,k,h} + \varepsilon_{d,k,h})}$$

,

$$d_{60,k,h,xCor} = 10^{(-1.65462\log_{10} a_{c,k,xCor} + 0.28299\log_{10} t_{c,k,h} + \varepsilon_{d,k,h})}$$

and

$$d_{30,k,h,xCor} = 10^{(-1.69464\log_{10} a_{c,k,xCor} + 0.36984\log_{10} t_{c,k,h} + \varepsilon_{d,k,h})}$$

where $t_{c,k,h}$ corresponds to the mean thickness of the cupula of the semicircular duct $_k$ when assuming the presence of stereocilia/kinocilia of various length at the surface of the *crista ampullaris*; where $\varepsilon_{d,k,h}$ corresponds to difference between the actual value of the $\log_{10}$ deflection parameter d and its estimated $\log_{10}$ value, for the uncorrected state; and where $xCor$ can either be $MinusCor$, Cor or $PlusCor$.

The effect in-plane mechanical sensitivity has on the biomechanical attunement of each semicircular duct, over the physiological frequency range of head motion, is expressed by in-plane mechanical gains, which relate the magnitude of responses of each semicircular duct to the magnitude of rotational stimuli at given frequencies. Semicircular ducts are mechanically in phase with angular

acceleration for head motion below their lower corner frequency, and mechanically in phase with angular velocity for head motion comprised between their lower corner frequency and their upper corner frequency (between their lower corner frequency and around their natural frequency for neurophysiological gain). For these portions of the head motion frequency bandwidth, semicircular ducts then present two different in-plane mechanical gains that are respectively relative to angular acceleration and to angular velocity. These mechanical gains are computed by **BiomechaParam** for each semicircular duct, based on a model of coupled ducts, by applying the following formulae:

$$\overrightarrow{G}_{\mathrm{V},x=e,k=a,h} = C'_{2,h}\overrightarrow{G_a} + c'_{23,h}\overrightarrow{G_p} + c'_{21,h}\overrightarrow{G_l}$$

,

$$\overrightarrow{G}_{\mathrm{V},x=e,k=p,h} = C'_{3,h}\overrightarrow{G_p} + c'_{32,h}\overrightarrow{G_a} + c'_{31,h}\overrightarrow{G_l}$$

,

$$\overrightarrow{G}_{\mathrm{V},x=e,k=l,h} = -C'_{1,h}\overrightarrow{G_l} - c'_{12,h}\overrightarrow{G_a} - c'_{13,h}\overrightarrow{G_p}$$

where

$$C_h^{-1} = \begin{bmatrix} C'_{1,h} & c'_{12,h} & c'_{13,h} \\ c'_{21,h} & C'_{2,h} & c'_{23,h} \\ c'_{31,h} & c'_{32,h} & C'_{3,h} \end{bmatrix}$$

and

$$G_{\mathrm{V},x=e,k,h} = \left\| \overrightarrow{G}_{\mathrm{V},x=e,k,h} \right\|$$

as well as

$$G_{\mathrm{A},x=e,k,h} = \frac{g_k}{k_{k,h}}$$

and

$$G_{\mathrm{V|A},x=c,k,h} = \frac{G_{\mathrm{V|A},x=e,k,h}}{a_{c,k}}$$

but also

$$G_{\mathrm{V|A},x=s,k,h=90} = \frac{G_{\mathrm{V|A},x=e,k,h=90}\cdot\left(d_{90,k,h=90} + d_{60,k,h=90} + d_{30,k,h=90}\right)}{3}$$

,

$$G_{\mathrm{V|A},x=s,k,h=60} = \frac{G_{\mathrm{V|A},x=e,k,h=60}\cdot\left(d_{60,k,h=60} + d_{30,k,h=60}\right)}{2}$$

and

$$G_{\mathrm{V|A},x=s,k,h=30} = G_{\mathrm{V|A},x=e,k,h=30}.d_{30,k,h=30}$$

where $G_{\mathrm{V},x,k,h}$ and $G_{\mathrm{A},x,k,h}$ respectively correspond to the in-plane mechanical gain of the semicircular duct $_k$ relative to head angular velocity and to head angular acceleration, expressed either in terms of total volume displacement of the cupulae $_{x=e}$, average transverse displacement of

the cupulae $_{x=c}$ or average deflection of areas of the cupulae where stereocilia/kinocilia can be found $_{x=s}$.

BiomechaParam also computes, based on a model of uncoupled ducts, the in-plane mechanical gain of each semicircular duct, relative to angular velocity, by applying the following formula:

$$\tilde{G}_{\mathrm{V},x,k,h} = \tilde{\tau}_{2,k,h}\tilde{X}_{x,k,h}$$

where $\tilde{G}_{\mathrm{V},x,k,h}$ corresponds to the in-plane mechanical gain of the semicircular duct $_k$ relative to head angular velocity and to head angular acceleration, expressed either in terms of total volume displacement of the cupulae $_{x=e}$, average transverse displacement of the cupulae $_{x=c}$ or average deflection of areas of the cupulae where stereocilia/kinocilia can be found $_{x=s}$.

Note that the in-plane mechanical gains mentioned above also stand for the maximal cupula volume displacement, the maximal cupula average transverse displacement and the maximal average deflection of areas of the cupulae where stereocilia/kinocilia can be found, that follows a step change in in-plane head angular velocity and in in-plane head angular acceleration, respectively[4].

Another aim of **BiomechaParam** is to allow transferring semicircular duct system parameters from the uncoupled vestibular frame of reference $B' = \left\{\vec{X}, \vec{Y}, \vec{Z}\right\}$ to the coupled vestibular frame of reference $B'' = \left\{\vec{X}', \vec{Y}', \vec{Z}'\right\}$. To do so, **BiomechaParam** computes the passage matrix:

$$P_{B''}^{B'} = \begin{bmatrix} \cos(\theta) & 0 & \sin(\theta) \\ 0 & 1 & 0 \\ -\sin(\theta) & 0 & \cos(\theta) \end{bmatrix}$$

where

$$\theta = \arccos(\vec{Z} \cdot {}_{B'}\vec{Z}')$$

where

$${}_{B'}\vec{Z}' = \frac{{}_{B'}\vec{L}_l - {}_{B'}\vec{L}_r}{\left\| {}_{B'}\vec{L}_l - {}_{B'}\vec{L}_r \right\|} = \begin{pmatrix} {}_{B'}Z'_x \\ {}_{B'}Z'_y \\ {}_{B'}Z'_z \end{pmatrix}$$

and where $\vec{Z}$ corresponds to the natural vestibular left yaw rotation, in the uncoupled vestibular frame of reference; where ${}_{B'}\vec{Z}'$ corresponds to the natural vestibular left yaw rotation, in the coupled vestibular frame of reference, with coordinates expressed in the uncoupled vestibular frame of reference; and where ${}_{B'}\vec{L}_n$ correspond to unit vectors that are normal to the maximal response planes of the right $_r$ or left $_l$ lateral semicircular ducts, based on a model of coupled ducts, oriented along their excitation direction and with coordinates expressed in the uncoupled vestibular frame of reference.

BiomechaParam then transfers the vector sets of the semicircular duct system, as well as the coordinates of the centers of rotation, from the uncoupled vestibular frame of reference, to the coupled vestibular frame of reference, by applying the following formula:

$$\vec{D}^{'}_{k,n} = P^{B'}_{B"} \vec{D}_{k,n}$$

where

$$D_k = \begin{pmatrix} D_{x,k} \\ D_{y,k} \\ D_{z,k} \end{pmatrix}$$

and

$$D^{'}_k = \begin{pmatrix} D^{'}_{X,k} \\ D^{'}_{Y,k} \\ D^{'}_{Z,k} \end{pmatrix}$$

correspond either to the unit vector that are normal to the maximal response plane of the semicircular duct $_k$, based on models of coupled and uncoupled ducts, or to the coordinates of its center of rotation, respectively in the uncoupled and coupled vestibular frames of reference.

From these results, 'lineset' files, which graphically represent the excitation direction of the maximal response axis of each semicircular duct, based on models of coupled ducts, are created by writing the coordinates of both the center of rotation of each semicircular duct as well as the vector component of their maximal response plane, in the coupled vestibular frame of reference, in Avizo Line Set format.

BiomechaParam also output the matrix:

$$P^{B}_{B"} = P^{B}_{B'} P^{B'}_{B"}$$

where $P^{B}_{B'}$ corresponds to the passage matrix from the scan frame of reference, to the uncoupled vestibular frame of reference; which allows to directly transfer objects from the scan frame of reference, to the coupled vestibular frame of reference.

Afterward, **BiomechaParam** computes the prime direction of each semicircular duct[4,9], by applying the following formulae:

$$\vec{p}_a = \vec{u}_p \times \vec{u}_l$$

,

$$\vec{p}_p = \vec{u}_l \times \vec{u}_a$$

and

$$\vec{p}_l = \vec{u}_a \times \vec{u}_p$$

where $\vec{u}_a$, $\vec{u}_p$ and $\vec{u}_l$ respectively correspond to unit vectors that are normal to the maximal response plane of the anterior $_a$, posterior $_p$ and lateral $_l$ semicircular ducts, based on a model of coupled

ducts, and are oriented along their excitation direction, following the right hand rule; and where $\vec{p}_a$, $\vec{p}_p$ and $\vec{p}_l$ respectively correspond to the prime directions of the anterior $_a$, posterior $_p$ and lateral $_l$ semicircular ducts, and are oriented along their excitation direction.

Next it computes the ipsilateral angular relationships of the semicircular duct planes, following conventions introduced by Blanks et al.[10], by applying the formulae:

$$\vec{A}_r \wedge \vec{P}_r = arc\cos(-\vec{A}_r \cdot -\vec{P}_r)$$

and

$$\vec{A}_r \wedge \vec{L}_r = arc\cos(-\vec{A}_r \cdot \vec{L}_r)$$

and

$$\vec{L}_r \wedge \vec{P}_r = arc\cos(\vec{L}_r \cdot \vec{P}_r)$$

where $\vec{A}_r \wedge \vec{P}_r$, $\vec{A}_r \wedge \vec{L}_r$ and $\vec{L}_r \wedge \vec{P}_r$ correspond to the angles between maximal response planes of the right anterior and posterior semicircular ducts, anterior and lateral semicircular ducts and lateral and posterior semicircular ducts, respectively; and where $\vec{A}_n$, $\vec{P}_n$ and $\vec{L}_n$ correspond to unit vectors that are normal to the maximal response planes of the right $_r$ or left $_l$ anterior, posterior and lateral semicircular ducts respectively, based on a model of coupled ducts, and oriented along their excitation direction.

Then, it uses the complete vector set of the semicircular duct system to compute the synergistic angular relationships of semicircular duct planes, applying the formulae:

$$\vec{A}_r \wedge \vec{P}_l = arc\cos(\vec{A}_r \cdot \vec{P}_l)$$

and

$$\vec{L}_r \wedge \vec{L}_l = arc\cos(\vec{L}_r \cdot \vec{L}_l)$$

where $\vec{A}_r \wedge \vec{P}_l$ and $\vec{L}_r \wedge \vec{L}_l$ correspond to the angles between maximal response planes of the right anterior and left posterior semicircular ducts, and right lateral and left lateral semicircular ducts, respectively.

After this, **BiomechaParam** computes the angular relationships of the maximal response planes of the right semicircular ducts with the three vestibular reference planes of the head, by applying the following formulae:

$$\vec{A}_r \wedge \overrightarrow{MidSag} = arc\cos(\vec{A}_r \cdot \vec{Y}')$$

,

$$\vec{P}_r \wedge \overrightarrow{MidSag} = arc\cos(-\vec{P}_r \cdot \vec{Y}')$$

and

$$\vec{L}_r \wedge \overrightarrow{MidSag} = arc\cos(-\vec{L}_r \cdot \vec{Y}')$$

where $\vec{A}_n \wedge \overrightarrow{MidSag}$, $\vec{P}_n \wedge \overrightarrow{MidSag}$ and $\vec{L}_n \wedge \overrightarrow{MidSag}$ correspond respectively to the angles between the maximal response planes of the right anterior, posterior and lateral semicircular ducts and the mid-sagittal plane of the head, as defined in the coupled vestibular frame of reference.

$$\vec{A}_r \wedge \overrightarrow{Cor} = arc\cos(\vec{A}_r \cdot \vec{X}')$$

,

$$\vec{P}_r \wedge \overrightarrow{Cor} = arc\cos(\vec{P}_r \cdot \vec{X}')$$

and

$$\vec{L}_r \wedge \overrightarrow{Cor} = arc\cos(\vec{L}_r \cdot \vec{X}')$$

where $\vec{A}_n \wedge \overrightarrow{Cor}$, $\vec{P}_n \wedge \overrightarrow{Cor}$ and $\vec{L}_n \wedge \overrightarrow{Cor}$ correspond respectively to the angles between the maximal response planes of the right anterior, posterior and lateral semicircular ducts and the coronal plane of the head, as defined in the coupled vestibular frame of reference.

$$\vec{A}_r \wedge \overrightarrow{Hor} = arc\cos(-\vec{A}_r \cdot \vec{Z}')$$

,

$$\vec{P}_r \wedge \overrightarrow{Hor} = arc\cos(\vec{P}_r \cdot \vec{Z}')$$

and

$$\vec{L}_r \wedge \overrightarrow{Hor} = arc\cos(-\vec{L}_r \cdot \vec{Z}')$$

where $\vec{A}_n \wedge \overrightarrow{Hor}$, $\vec{P}_n \wedge \overrightarrow{Hor}$ and $\vec{L}_n \wedge \overrightarrow{Hor}$ correspond respectively to the angles between the maximal response planes of the right anterior, posterior and lateral semicircular ducts and the horizontal plane of the head, as defined in the coupled vestibular frame of reference.

Next, **BiomechaParam** computes the variance of the ipsilateral angular relationships between semicircular duct planes from orthogonality (90var[2]), as well as their average deviation from orthogonality (90dev[3]), by applying the formulae:

$$90_{\mathrm{var}} = \frac{\left(90 - \vec{A}_r \wedge \vec{P}_r\right)^2 + \left(90 - \vec{A}_r \wedge \vec{L}_r\right)^2 + \left(90 - \vec{L}_r \wedge \vec{P}_r\right)^2}{3}$$

and

$$90_{dev} = \frac{\sqrt{\left(90 - \vec{A}_r \wedge \vec{P}_r\right)^2} + \sqrt{\left(90 - \vec{A}_r \wedge \vec{L}_r\right)^2} + \sqrt{\left(90 - \vec{L}_r \wedge \vec{P}_r\right)^2}}{3}$$

where 90_{var} corresponds to the variance of the ipsilateral angular relationships between the maximal response planes of the semicircular ducts from orthogonality, as defined by Malinzak et al.[2]; and where 90_{dev} corresponds to the average deviation of the ipsilateral angular relationships between the maximal response planes of the semicircular ducts from orthogonality, as defined by Berlin et al.[3].

Then, **BiomechaParam** computes the variance of the synergistic angular relationships between semicircular duct planes from planarity (180var[2]), as well as their average deviation from planarity (180dev), by applying the formulae:

$$180_{\mathrm{var}} = \frac{\left(180 - \vec{A}_r \wedge \vec{P}_l\right)^2 + \left(180 - \vec{A}_l \wedge \vec{P}_r\right)^2 + \left(180 - \vec{L}_r \wedge \vec{L}_l\right)^2}{3}$$

and

$$180_{dev} = \frac{\sqrt{\left(180 - \vec{A}_r \wedge \vec{P}_l\right)^2} + \sqrt{\left(180 - \vec{A}_l \wedge \vec{P}_r\right)^2} + \sqrt{\left(180 - \vec{L}_r \wedge \vec{L}_l\right)^2}}{3}$$

where 180_{var} corresponds to the variance of the synergistic angular relationships between the maximal response planes of the semicircular ducts from planarity, as defined by Malinzak et al.[2]; and where 180_{dev} corresponds to the average deviation of the synergistic angular relationships between the maximal response planes of the semicircular ducts from planarity.

Finally, **BiomechaParam** computes the displacement profile factor of each cupula by applying the following formulae derived from[4]:

$$\gamma = \frac{E}{2(1+\nu)}$$

,

$$\lambda_{\gamma,k,h} = \frac{k_{c,k,h} a_{c,k}^2}{\gamma t_{c,k,h}}$$

where γ corresponds to the shear modulus of the cupula; E corresponds to the young modulus of the cupula; ν corresponds to the Poisson ratio of the cupula; and where $\lambda_{\gamma,k,h}$ corresponds to the displacement profile factor of the cupula of the semicircular duct.

Technical note: BiomechaParam corrects the displacement profile factors $\lambda_{\gamma,k,h}$ for potential segmentation errors. To do so, it implements the following regression that was retrieved via the study of 37 mammal specimens:

$$\lambda_{\gamma,k,h,xCor} = 10^{\left(-0.20786 \log_{10} a_{c,k,xCor} + 0.31842 \log_{10} t_{c,k,h} + \varepsilon_{\gamma,k,h}\right)}$$

where $\varepsilon_{\gamma,k,h}$ corresponds to difference between the actual value of the $\log_{10}$ displacement profile factor $\lambda_{\gamma,k,h}$ and its estimated $\log_{10}$ value, for the uncorrected state.

Using these corrected factors, **BiomechaParam** also correct the stiffness parameter of each cupula $k_{c,k,h}$ for potential segmentation error, by following the formula:

$$k_{c,k,h,xCor} = \frac{\gamma . \lambda_{\gamma,k,h,xCor} . t_{c,k,h}}{a_{c,k,xCor}^2}$$

At the end of the procedure, **BiomechaParam** output several files containing information on all computed parameters and metrics described above.

d. Input Files

As input, **BiomechaParam** needs the files:

SPECIESNAME_Suffix.morph

with **Suffix** successively replaced by **Cor**, **MinusCor** and **PlusCor**, to be placed in the folder **/AriadneToolbox/Data/SPECIESNAME/** (see section I.iii.9.).

The files:

rfea_SPECIESNAME_Ant_Cilia_Long.deflection **rfea_SPECIESNAME_Ant_Cilia_Medium.deflection**

rfea_SPECIESNAME_Ant_Cilia_Short.deflection **rfea_SPECIESNAME_Post_Cilia_Long.deflection**

rfea_SPECIESNAME_Post_Cilia_Medium.deflection **rfea_SPECIESNAME_Post_Cilia_Short.deflection**

rfea_SPECIESNAME_Lat_Cilia_Long.deflection **rfea_SPECIESNAME_Lat_Cilia_Medium.deflection**

rfea_SPECIESNAME_Lat_Cilia_Short.deflection **rfea_SPECIESNAME_Ant_Cilia_Long.sif**

to be placed in the folder **/AriadneToolbox/Data/SPECIESNAME/FEA/** (see sections I.iv.1. & I.iv.2.).

And finally the file:

SPECIESNAME_uncoupled.transmat

To be placed in the folder **/AriadneToolbox/Data/SPECIESNAME/Matrix/** (see section I.iii.8.). Note that this input file is suppressed when **BiomechaParam** work is over.

e. Output Files

BiomechaParam outputs the files:

SPECIESNAME_Suffix.biomecha **SPECIESNAME_Suffix_Extended.biomecha**

in the folder **/AriadneToolbox/Data/SPECIESNAME/**, with **Suffix** successively replaced by **Cor**, **MinusCor** and **PlusCor**.

The files:

rma_SPECIESNAME_Ant.lineset **rma_SPECIESNAME_Lat.lineset**

rma_SPECIESNAME_Post.lineset **lma_SPECIESNAME_Ant.lineset**

lma_SPECIESNAME_Post.lineset **lma_SPECIESNAME_Lat.lineset**

in the folder **/AriadneToolbox/Data/SPECIESNAME/VestRef/MaxAxes/**.

And finally the file:

SPECIESNAME.transmat

in the folder **/AriadneToolbox/Data/SPECIESNAME/Matrix/**.

.biomecha files contain information, based on a model of coupled ducts, about (1) the vector components of the maximal response plane of each semicircular duct, in the scan frame of reference, (2) the center of rotation of each semicircular duct, in the scan frame of reference, (3) the vector components of the prime direction of each semicircular duct, in the vestibular frame of reference, (4) the ipsilateral angular relationships of the maximal response planes of the semicircular ducts, (5) the synergistic angular relationships of the maximal response planes of the semicircular ducts, (6) the angular relationships of the maximal response planes of the semicircular ducts, with the three vestibular reference planes of the head, (7) the variance of the ipsilateral angular relationships of the maximal response planes of the semicircular ducts from orthogonality (90var[2]), (8) the average deviation of the ipsilateral angular relationships of the maximal response planes of the semicircular ducts from orthogonality (90dev[3]), (9) the variance of the synergistic angular relationships of the maximal response planes of the semicircular ducts from planarity (180var), (10) the average deviation of the synergistic angular relationships of the maximal response planes of the semicircular ducts from planarity (180dev), (11) the displacement profile factor of each cupula, (12) the deflection of the stereocilia/kinocilia areas of each cupula, (13) the mass parameters of each semicircular duct and common parts, (14) the damping parameters of each semicircular duct and common parts, (15) the stiffness parameter of each cupula, (16) the inertial forcing parameter of each semicircular duct torus, (17) the long time constant of each semicircular duct, (18) the short time constant of each semicircular duct, (19) the lower corner frequency of each semicircular duct, (20) the upper corner frequency of each semicircular duct, (21) the uncoupled natural frequency of each semicircular duct, and (22) the uncoupled fractional bandwidth of the middle frequency range of each semicircular duct below and above their natural frequency, all with three levels of correction for potential shrinkage and segmentation errors and, when applicable, with three different assumptions on stereocilia/kinocilia length.
.biomecha files also contain information about (1) the in-plane mechanical sensitivity of each semicircular duct, relative to angular rotation, (2) the in-plane velocity gain of each semicircular duct, relative to angular velocity, and (3) the in-plane acceleration gain of each semicircular duct, relative to angular acceleration, all expressed in terms of average deflection of areas of the cupulae where stereocilia/kinocilia can be found, and each time with three levels of correction for potential shrinkage and segmentation errors, and with three different assumptions on stereocilia/kinocilia length.

Finally, **.biomecha** files contain information about the viscosity of the endolymph and the cupulae, as computed from body temperature by **BiomechaParam**, as well as information about the other physiological parameters that were used to compute the biomechanics (see sections I.iv.1.b. & I.v.1.b.).

Extended **.biomecha** files contain similar information but also (1) additionally provide biomechanical parameters based on a model of uncoupled ducts, (2) detail the mass and damping parameters for each part of the semicircular duct system and (3) additionally express the mechanical sensitivity, the

velocity gain and the acceleration gain in terms of cupula volume displacement and cupula average transverse displacement.

The **.transmat** file corresponds to a square matrix that can be used by **DataVestibularReference** (section I.vi.1.) and **STLVestibularReference** (section I.vi.2.) to transfer data objects (linesets, landmarks, STLs) in the vestibular frame of reference, based on a model of coupled ducts.

.lineset files can be opened in Avizo 6 or 7 and can be visualized using [Display> Line Set View]. They graphically represent the excitation direction of the maximal response axis of each semicircular duct, following the right hand rule and based on a model of coupled ducts.

2. BodePlotsGenerator

a. Description

BodePlotsGenerator is an executable, written in C, which generates data, based on a model of coupled ducts, that can be visualized in the form of bode plots using **SciLab** 5.4.0 (see tutorial in section I.i. and accompanying Fig.4). These bode plots describe the continuous variation of both mechanical phase and gain of each semicircular duct across the whole frequency bandwidth of head motion, relative to angular velocity. **BodePlotsGenerator** produce data for in-plane, for pitch, for roll and for yaw rotations, each time with three different assumptions on stereocilia/kinocilia length, with three levels of correction for potential shrinkage and segmentation errors, and expressed in units of cupula volumic displacement, cupula average linear displacement and cilia average deflection.

BodePlotsGenerator can either be used as stand-alone software by double-clicking on **BodePlotsGenerator.exe**, in the folder **/AriadneToolbox/**, and following instructions appearing in the console, or as part of the batch processes **Ariadne_SCDS.bat**, **Ariadne_SCDS_Lite.bat** or **BiomechanicsAuto.bat** (sections I.i & I.v.).

b. Algorithm

In order to compute the continuous variation of both mechanical phase and gain of each semicircular duct across the whole head motion frequency bandwidth, relative to angular velocity, **BodePlotsGenerator** first reads-in files containing information about semicircular ducts biomechanics (section I.v.1.).

Then, it builds, based on a model of coupled ducts and across the head motion frequency bandwidth (0.01 – 100 Hz), the matrices:

$$L_h(f) = K_h + i\omega C_h - \omega^2 M_h$$

where

$$M_h = \begin{bmatrix} M_{1,h} & m_{12} & 0 \\ m_{12} & M_{2,h} & m_{23} \\ 0 & m_{23} & M_{3,h} \end{bmatrix}$$

,

$$C_h = \begin{bmatrix} C_{1,h} & c_{12} & 0 \\ c_{12} & C_{2,h} & c_{23} \\ 0 & c_{23} & C_{3,h} \end{bmatrix}$$

and

$$K_h = \begin{bmatrix} K_{1,h} & 0 & 0 \\ 0 & K_{2,h} & 0 \\ 0 & 0 & K_{3,h} \end{bmatrix}$$

where

$$\omega = 2\pi f$$

and where $M_{1,h}$ corresponds to the mass parameter of the lateral semicircular duct; $M_{2,h}$ corresponds to the mass parameter of the anterior semicircular duct; and $M_{3,h}$ corresponds to the mass parameter of the posterior semicircular duct, assuming a length $_h$ for the longest stereocilia/kinocilia; where m_{12} corresponds to the mass parameter of the part of the anterior utricle that is shared between the anterior and lateral semicircular ducts; and m_{23} corresponds to the mass parameter of the part of the common crus that is shared between the anterior and posterior semicircular ducts; where $C_{1,h}$ corresponds to the damping parameter of the lateral semicircular duct; $C_{2,h}$ corresponds to the damping parameter of the anterior semicircular duct; and $C_{3,h}$ corresponds to the damping parameter of the posterior semicircular duct; where c_{12} corresponds to the damping parameter of the part of the anterior utricle that is shared between the anterior and lateral semicircular ducts; and c_{23} corresponds to the damping parameter of the part of the common crus that is shared between the anterior and posterior semicircular ducts; where $K_{1,h}$ corresponds to the stiffness parameter of the lateral cupula; $K_{2,h}$ corresponds to the stiffness parameter of the anterior cupula; and $K_{3,h}$ corresponds to the stiffness parameter of the posterior cupula; where f corresponds to head motion frequencies; and where i corresponds to the imaginary unit.

Next, **BodePlotsGenerator** construct the vectors:

$$\overrightarrow{G_a} = g_a \cdot_u \vec{u}_a$$

,

$$\overrightarrow{G_p} = g_p \cdot_u \vec{u}_p$$

and

$$\overrightarrow{G_l} = g_l \cdot -_u \vec{u}_l$$

where ${}_u\vec{u}_a$, ${}_u\vec{u}_p$ and ${}_u\vec{u}_l$ respectively correspond to unit vectors that are normal to the maximal response plane of the anterior $_a$, posterior $_p$ and lateral $_l$ semicircular ducts, based on a model of uncoupled ducts, and are oriented along their excitation direction, following the right hand rule; and where g_k corresponds to the inertial forcing parameter of the semicircular duct $_k$.

From this, **BodePlotsGenerator** computes, based on a model of coupled ducts and across the head motion frequency bandwidth (0.01 – 100 Hz), complex maximal response vectors relative to angular velocity, for each semicircular duct, by applying the following formulae:

$$\overrightarrow{R}_{x=e,k=a,h,f} = \omega(L'_{2,h,f}\overrightarrow{G_a} + l'_{23,h,f}\overrightarrow{G_p} + l'_{21,h,f}\overrightarrow{G_l})$$

,

$$\overrightarrow{R}_{x=e,k=p,h,f} = \omega(L'_{3,h,f}\overrightarrow{G_p} + l'_{32,h,f}\overrightarrow{G_a} + l'_{31,h,f}\overrightarrow{G_l})$$

,

$$\overrightarrow{R}_{x=e,k=l,h,f} = -\omega(L'_{1,h,f}\overrightarrow{G_l} + l'_{12,h,f}\overrightarrow{G_a} + l'_{13,h,f}\overrightarrow{G_p})$$

where

$$L_h^{-1}(f) = \begin{bmatrix} L'_{1,h,f} & l'_{12,h,f} & l'_{13,h,f} \\ l'_{21,h,f} & L'_{2,h,f} & l'_{23,h,f} \\ l'_{31,h,f} & l'_{32,h,f} & L'_{3,h,f} \end{bmatrix}$$

and where $\overrightarrow{R}_{x,k,h,f}$ corresponds to in-plane complex maximal response vectors of the anterior $_a$, posterior $_p$ and lateral $_l$ semicircular ducts, relative to head angular velocity, for head motion frequencies f, and expressed in terms of total volume displacement of the cupulae $_{x=e}$.

From these vectors, **BodePlotsGenerator** computes the continuous variation of the in-plane mechanical gain of each semicircular duct across the head motion frequency bandwidth, relative to angular velocity, by applying the following formula:

$$G_{\mathrm{V}}\left(f\right)_{x=e,k,h} = \sqrt{\sum_j \mathrm{Re}\left({}_j R_{x=e,k,h,f}\right)^2 + \mathrm{Im}\left({}_j R_{x=e,k,h,f}\right)^2}$$

where $G_{\mathrm{V}}\left(f\right)_{x=e,k,h}$ corresponds to the in-plane mechanical gain of the semicircular duct $_k$ relative to head angular velocity, at head motion frequencies f; where $_j$ corresponds to vector components in the pitch $_y$, roll $_x$ or yaw $_z$ axes; and where Re and Im respectively stand for the real and the imaginary part of the components of the complex vector $\overrightarrow{R}_{x,k,h,f}$.

Also, **BodePlotsGenerator** computes the continuous variation of the mechanical gain of each semicircular duct across the head motion frequency bandwidth, relative to angular velocity, for pitch roll and yaw rotations, by applying the following formula:

$$ {}_{j}G_{\mathrm{V}}\left(f\right)_{x=e,k,h}=\sqrt{\mathrm{Re}\left({}_{j}R_{x=e,k,h,f}\right)^{2}+\mathrm{Im}\left({}_{j}R_{x=e,k,h,f}\right)^{2}} $$

where ${}_{j}G_{\mathrm{V}}\left(f\right)_{x,k,h}$ corresponds to the mechanical gain of the semicircular duct $_{k}$ relative to head angular velocity, for pitch $_{j=y}$, roll $_{j=x}$, and yaw $_{j=z}$ rotations, at head motion frequencies f.

Above mentioned gain parameters are re-expressed in terms of average transverse displacement of the cupulae $_{x=c}$ or average deflection of areas of the cupulae where stereocilia/kinocilia can be found $_{x=s}$, by applying the formulae:

$$ G_{\mathrm{V}}\left(f\right)_{x=c,k,h}=\frac{G_{\mathrm{V}}\left(f\right)_{x=e,k,h}}{a_{c,k}} $$

but also

$$ G_{\mathrm{V}}\left(f\right)_{x=s,k,h=90}=\frac{G_{\mathrm{V}}\left(f\right)_{x=e,k,h=90}.\left(d_{90,k,h=90}+d_{60,k,h=90}+d_{30,k,h=90}\right)}{3} $$

,

$$ G_{\mathrm{V}}\left(f\right)_{x=s,k,h=60}=\frac{G_{\mathrm{V}}\left(f\right)_{x=e,k,h=60}.\left(d_{60,k,h=60}+d_{30,k,h=60}\right)}{2} $$

and

$$ G_{\mathrm{V}}\left(f\right)_{x=s,k,h=30}=G_{\mathrm{V}}\left(f\right)_{x=e,k,h=30}.d_{30,k,h=30} $$

where $a_{c,k}$ correspond to the mean cross-sectional area of the cupula of the semicircular duct $_{k}$; and where $d_{30,k,h=90}$, $d_{30,k,h=60}$, $d_{30,k,h=30}$, $d_{60,k,h=90}$, $d_{60,k,h=60}$ and $d_{90,k,h=90}$ correspond to assumed mean deflections of respective areas of the cupula of the semicircular duct $_{k}$.

In order to retrieve the continuous variation of the mechanical phase of each semicircular duct across the head motion frequency bandwidth (0.01 – 100 Hz), **BodePlotsGenerator** first computes unit maximal response vectors relative to angular velocity, for each semicircular duct, by following the formula:

$$ \vec{u}\left(f\right)_{k,h}=\begin{bmatrix}\dfrac{\mathrm{Re}\left({}_{x}R_{x=e,k,h,f}\right)}{\sqrt{\sum_{j}\mathrm{Re}\left({}_{j}R_{x=e,k,h,f}\right)^{2}}}\\ \dfrac{\mathrm{Re}\left({}_{y}R_{x=e,k,h,f}\right)}{\sqrt{\sum_{j}\mathrm{Re}\left({}_{j}R_{x=e,k,h,f}\right)^{2}}}\\ \dfrac{\mathrm{Re}\left({}_{z}R_{x=e,k,h,f}\right)}{\sqrt{\sum_{j}\mathrm{Re}\left({}_{j}R_{x=e,k,h,f}\right)^{2}}}\end{bmatrix} $$

where $\vec{u}(f)_{k,h}$ corresponds to the unit maximal response vectors of the semicircular duct $_k$, relative to head angular velocity, for head motion frequencies f; and where $_j$ corresponds to vector components in the pitch $_y$, roll $_x$ or yaw $_z$ axes.

From this, it computes the complex responses $_uR_{x,k,h,f}$ of each semicircular duct $_k$, relative to angular velocity, for rotations around these unit maximal response vectors and for head motion frequencies f, by following the formulae:

$$_uR_{x=e,k=a,h,f} = \omega(L'_{2,h,f}\overrightarrow{G_a}\cdot\vec{u}(f)_{a,h} + l'_{23,h,f}\overrightarrow{G_p}\cdot\vec{u}(f)_{a,h} + l'_{21,h,f}\overrightarrow{G_l}\cdot\vec{u}(f)_{a,h})$$

,

$$_uR_{x=e,k=p,h,f} = \omega(L'_{3,h,f}\overrightarrow{G_p}\cdot\vec{u}(f)_{p,h} + l'_{32,h,f}\overrightarrow{G_a}\cdot\vec{u}(f)_{p,h} + l'_{31,h,f}\overrightarrow{G_l}\cdot\vec{u}(f)_{p,h})$$

,

$$_uR_{x=e,k=l,h,f} = \omega(L'_{1,h,f}\overrightarrow{G_l}\cdot\vec{u}(f)_{l,h} + l'_{12,h,f}\overrightarrow{G_a}\cdot\vec{u}(f)_{l,h} + l'_{13,h,f}\overrightarrow{G_p}\cdot\vec{u}(f)_{l,h})$$

Finally, the mechanical phase $\Phi_V(f)_{k,h}$ of each semicircular duct $_k$ relative to head angular velocity, at head motion frequencies f, is computed following the formula:

$$\Phi_V(f)_{k,h} = \frac{\pi}{2} - \arccos\left(\frac{\overrightarrow{G_k}\cdot\vec{u}(f)_{k,h}}{\left\|\overrightarrow{G_k}\cdot\vec{u}(f)_{k,h}\right\|}\cdot\frac{\mathrm{Re}(_uR_{x=e,k,h,f})}{\sqrt{\mathrm{Re}(_uR_{x=e,k,h,f})^2 + \mathrm{Im}(_uR_{x=e,k,h,f})^2}}\right)$$

At the end of the procedure, **BodePlotsGenerator** output several files that can be visualized as bode plots using **SciLab** 5.4.0 (see tutorial in section I.i. and accompanying Fig.4).

c. Input Files

As input, **BodePlotsGenerator** needs the files:

SPECIESNAME_Suffix_Extended.biomecha

with **Suffix** successively replaced by **Cor**, **MinusCor** and **PlusCor**, to be placed in the folder **/AriadneToolbox/Data/SPECIESNAME/** (see section I.v.1.).

d. Output Files

BodePlotsGenerator outputs the files:

SPECIESNAME_Suffix_Volume.bode **SPECIESNAME_Suffix_Cupula.bode**

SPECIESNAME_Suffix_Cilia.bode

in the folder **/AriadneToolbox/Data/SPECIESNAME/BodePlots/**, with **Suffix** successively replaced by **Cor**, **MinusCor** and **PlusCor**.

.bode files contain data, based on a model of coupled ducts, that can be visualized in the form of bode plots using **SciLab** 5.4.0 (see tutorial in section I.i. and accompanying Fig.4). Bode plots are produced for in-plane, pitch, roll and yaw rotations and show the effects the three different assumptions on stereocilia/kinocilia length have on both mechanical phase and gain of the semicircular ducts. To visualize the data, the user can choose to apply three different levels of correction for potential shrinkage and segmentation errors, and to express the mechanical gain relative to angular velocity either as cupula volume displacement, as cupula average transverse displacement or as average deflection of areas of the cupulae where stereocilia/kinocilia can be found, per degree per second.

3. SensitivityMapping

a. Description

SensitivityMapping is an executable, written in C, which generates data, based on a model of coupled ducts, which can be visualized in the form of Mercator projections using **SciLab** 5.4.0 (see tutorial in section I.i. and accompanying Fig.5). These maps describe, each time with three levels of correction for potential shrinkage and segmentation errors, three different assumptions on stereocilia/kinocilia length, two assumption on gain summation, and with up to three different sensitivity metrics, the spatial distribution of:

- the average mechanical gain
- the standard deviation of the mechanical gain
- the maximal mechanical gain
- the saturating mechanical gain
- the standardized mechanical gain
- the 'Hullar sensitivity[2]' of the semicircular duct system

covering the full directional range of head motion.

SensitivityMapping also outputs files summarizing these maps by providing statistical information including the global mean, the global standard deviation, the maximum and the minimum of the corresponding sensitivity values across the whole spatial distribution, as well as information about the specific sensitivity values that are retrieved for pitch, roll and yaw rotations.

SensitivityMapping can either be used as stand-alone software by double-clicking on **SensitivityMapping.exe**, in the folder **/AriadneToolbox/**, and following instructions appearing in the console, or as part of the batch processes **Ariadne_SCDS.bat**, **Ariadne_SCDS_Lite.bat** or **BiomechanicsAuto.bat** (sections I.i & I.v.).

b. Algorithm

In order to compute the various sensitivity maps described above, along with their corresponding statistical summaries, **SensitivityMapping** first defines a sphere of radius 1, which is centered on the origin of the vestibular frame of reference, and whose surface represent the set of the tips of all possible unit head angular acceleration/velocity vectors $\vec{\Omega}_{(\theta,\varphi)}$ running from its center. Any point at

the surface of this sphere, and by extension any unit head rotation vector, is identified by the combination of an azimuth coordinate θ and of an altitude coordinate φ, such that (1) right roll unit head rotation vector points toward the point of coordinates θ = 0° or 360° and φ = 90°, (2) nose-down pitch unit head rotation vector points toward the point of coordinates θ = 90° and φ = 90°, and (3) left yaw unit head rotation vector points toward the point of coordinates θ = [0° : 360°] and φ = 0° (remapped to φ = 180° in visualizations). Coordinates of any unit head rotation vector, in the vestibular frame of reference, are obtained from the spherical coordinates by applying the formula:

$$\vec{\Omega}_{(\theta,\varphi)} = \begin{bmatrix} \cos(\theta)\sin(\varphi) \\ \sin(\theta)\sin(\varphi) \\ \cos(\varphi) \end{bmatrix}$$

From there, **SensitivityMapping** reads-in files containing information about semicircular ducts biomechanics (section I.vi.1.) and begins the mapping process, focusing first on the average of absolute mechanical gains of the semicircular duct system, per rotation (abridged as 'average mechanical gain'). To produce the sensitivity map, **SensitivityMapping** (1) retrieves the values of the average mechanical gain that result from each of 64442 equally spaced unit head rotation vectors, covering the full directional range of head motion, and (2) registers each of these values in a double entry table, under the spherical coordinates (θ,φ) of the corresponding head rotation vector that elicited it. To do so, **SensitivityMapping** apply the following formulae, for each of the 64442 head rotation vectors being considered:

$$G_{Av,x,\mathrm{M},h,(\theta,\varphi)} = \sum_{k=1}^{6} \frac{\left\| G_{\mathrm{M},x,k,h,(\theta,\varphi)} \right\|}{6}$$

and

$$G_{SynAv,x,\mathrm{M},h,(\theta,\varphi)} = \frac{\left\| G_{\mathrm{M},x,Ar,h,(\theta,\varphi)} - G_{\mathrm{M},x,Pl,h,(\theta,\varphi)} \right\| + \left\| G_{\mathrm{M},x,Al,h,(\theta,\varphi)} - G_{\mathrm{M},x,Pr,h,(\theta,\varphi)} \right\| + \left\| G_{\mathrm{M},x,Lr,h,(\theta,\varphi)} - G_{\mathrm{M},x,Ll,h,(\theta,\varphi)} \right\|}{3}$$

where

$$G_{\mathrm{M},x,k,h,(\theta,\varphi)} = G_{\mathrm{M},x,k,h}\left(\vec{\Omega}_{(\theta,\varphi)} \cdot \vec{u}_k\right)$$

and where $G_{Av,x,\mathrm{M},h,(\theta,\varphi)}$ corresponds to the average $_{Av}$ of absolute mechanical gains that are elicited in all six semicircular ducts by the unit head rotation vector $\vec{\Omega}_{(\theta,\varphi)}$, relative to either head angular acceleration $_{\mathrm{M=A}}$ or head angular velocity $_{\mathrm{M=V}}$, expressed either in terms of total volume displacement of the cupulae $_{x=e}$, average transverse displacement of the cupulae $_{x=c}$ or average deflection of areas of the cupulae where stereocilia/kinocilia can be found $_{x=s}$, and assuming a length $_{h}$ for the longest stereocilia/kinocilia; where $G_{\mathrm{M},x,k,h,(\theta,\varphi)}$ corresponds to the signed mechanical gain that is elicited in either the right anterior $_{k=Ar}$, left anterior $_{k=Al}$, right posterior $_{k=Pr}$, left posterior $_{k=Pl}$, right lateral $_{k=Lr}$ or left lateral $_{k=Ll}$ semicircular duct, by the unit head rotation vector $\vec{\Omega}_{(\theta,\varphi)}$; where $\vec{u}_k$ corresponds to the unit vector that is normal to the maximal response plane

of the semicircular duct $_k$, and which is oriented along the excitation direction following the right hand rule; where $G_{\mathrm{M},x,k,h}$ corresponds to the in-plane mechanical gain of the semicircular duct $_k$; and where $G_{Syn,x,\mathrm{M},h,(\theta,\varphi)}$ corresponds to the average of absolute combined mechanical gains that are elicited in all three synergistic $_{SynAv}$ semicircular duct pairs by the unit head rotation vector $\vec{\Omega}_{(\theta,\varphi)}$.

When all 64442 head rotation vectors have been processed, **SensitivityMapping** start summarizing the spatial maps of the average mechanical gain by registering the values that were retrieved for unit nose-down pitch $\vec{\Omega}_{(90,90)}$, right roll $\vec{\Omega}_{(0,90)}$ and left yaw $\vec{\Omega}_{(0,0)}$ head rotations. **SensitivityMapping** continues the summarization process by registering the maximum and minimum values of the average mechanical gain that were retrieved during the creation of the spatial maps. Finally, **SensitivityMapping** finishes summarizing the spatial maps by computing and registering the global mean, and the global standard deviation, of the values of the average mechanical gain that were retrieved from the 64442 analyzed head rotations. To do so, it applies the following formulae:

$$G_{Map,x,\mathrm{M},h,Mean} = \sum_{i=1}^{64442} \frac{G_{Map,x,\mathrm{M},h,i}}{64442}$$

and

$$G_{Map,x,\mathrm{M},h,Sdv} = \sqrt{\sum_{i=1}^{64442} \frac{\left(G_{Map,x,\mathrm{M},h,i} - G_{Map,x,\mathrm{M},h,Mean}\right)^2}{64442}}$$

where $G_{Map,x,\mathrm{M},h,Mean}$ corresponds to the global mean $_{Mean}$ of either (1) the values that were retrieved, from the 64442 analyzed head rotation vectors, for the average of absolute mechanical gains that are elicited in all six semicircular ducts $_{Map=Av}$, or of (2) the values that were instead retrieved for the average of absolute combined mechanical gains that are elicited in all three synergistic semicircular duct pairs $_{Map=SynAv}$; where $G_{Map,x,\mathrm{M},h,Sdv}$ corresponds to the global standard deviation $_{Sdv}$ of the values of the average mechanical gain that were retrieved from the 64442 analyzed head rotation vectors; and where $G_{Map,x,\mathrm{M},h,i}$ corresponds to the value of the average mechanical gain that was retrieved for the i-th tested head rotation vector.

Next, **SensitivityMapping** focuses on mapping the standard deviation of absolute mechanical gains of the semicircular duct system, per rotation (abridged as 'standard deviation of the mechanical gain'). To produce the corresponding sensitivity map, it applies the following formulae, for each of the 64442 head rotation vectors being considered:

$$G_{SD,x,\mathrm{M},h,(\theta,\varphi)} = \sqrt{\sum_{k=1}^{6} \frac{\left(\left\|G_{\mathrm{M},x,k,h,(\theta,\varphi)}\right\| - G_{Av,x,\mathrm{M},h,(\theta,\varphi)}\right)^2}{6}}$$

and

$$G_{SynSD,x,\mathrm{M},h,(\theta,\varphi)} = \sqrt{\frac{{}_aG_{SynSD,x,\mathrm{M},h,(\theta,\varphi)} + {}_bG_{SynSD,x,\mathrm{M},h,(\theta,\varphi)} + {}_cG_{SynSD,x,\mathrm{M},h,(\theta,\varphi)}}{3}}$$

where

$$_{a}G_{SynSD,x,\mathrm{M},h,(\theta,\varphi)} = \left(\left\|G_{\mathrm{M},x,Ar,h,(\theta,\varphi)} - G_{\mathrm{M},x,Pl,h,(\theta,\varphi)}\right\| - G_{SynAv,x,\mathrm{M},h,(\theta,\varphi)}\right)^2$$

,

$$_{b}G_{SynSD,x,\mathrm{M},h,(\theta,\varphi)} = \left(\left\|G_{\mathrm{M},x,Al,h,(\theta,\varphi)} - G_{\mathrm{M},x,Pr,h,(\theta,\varphi)}\right\| - G_{SynAv,x,\mathrm{M},h,(\theta,\varphi)}\right)^2$$

and

$$_{c}G_{SynSD,x,\mathrm{M},h,(\theta,\varphi)} = \left(\left\|G_{\mathrm{M},x,Lr,h,(\theta,\varphi)} - G_{\mathrm{M},x,Ll,h,(\theta,\varphi)}\right\| - G_{SynAv,x,\mathrm{M},h,(\theta,\varphi)}\right)^2$$

and where $G_{SD,x,\mathrm{M},h,(\theta,\varphi)}$ corresponds to the standard deviation $_{SD}$ of absolute mechanical gains that are elicited in all six semicircular ducts by the unit head rotation vector $\vec{\Omega}_{(\theta,\varphi)}$; and where $G_{SynSD,x,\mathrm{M},h,(\theta,\varphi)}$ corresponds to the standard deviation of absolute combined mechanical gains that are elicited in all three synergistic $_{SynSD}$ semicircular duct pairs by the unit head rotation vector $\vec{\Omega}_{(\theta,\varphi)}$.

When all 64442 head rotation vectors have been processed, **SensitivityMapping** start summarizing the spatial maps of the standard deviation of the mechanical gain by registering the values that were retrieved for unit nose-down pitch $\vec{\Omega}_{(90,90)}$, right roll $\vec{\Omega}_{(0,90)}$ and left yaw $\vec{\Omega}_{(0,0)}$ head rotations. **SensitivityMapping** continues the summarization process by registering the maximum and minimum values of the standard deviation of the mechanical gain that were retrieved during the creation of the spatial maps. Finally, **SensitivityMapping** finishes summarizing the spatial maps by computing and registering the global mean, and the global standard deviation, of the values of the standard deviation of the mechanical gain that were retrieved from the 64442 analyzed head rotations. To do so, it applies the following formulae:

$$G_{Map,x,\mathrm{M},h,Mean} = \sum_{i=1}^{64442} \frac{G_{Map,x,\mathrm{M},h,i}}{64442}$$

and

$$G_{Map,x,\mathrm{M},h,Sdv} = \sqrt{\sum_{i=1}^{64442} \frac{\left(G_{Map,x,\mathrm{M},h,i} - G_{Map,x,\mathrm{M},h,Mean}\right)^2}{64442}}$$

where $G_{Map,x,\mathrm{M},h,Mean}$ corresponds to the global mean $_{Mean}$ of either (1) the values that were retrieved, from the 64442 analyzed head rotation vectors, for the standard deviation of absolute mechanical gains that are elicited in all six semicircular ducts $_{Map=SD}$, or of (2) the values that were instead retrieved for the standard deviation of absolute combined mechanical gains that are elicited in all three synergistic semicircular duct pairs $_{Map=SynSD}$; where $G_{Map,x,\mathrm{M},h,Sdv}$ corresponds to the global standard deviation $_{Sdv}$ of the values of the standard deviation of the mechanical gain that were retrieved from the 64442 analyzed head rotation vectors; and where $G_{Map,x,\mathrm{M},h,i}$ corresponds to the value of the standard deviation of the mechanical gain that was retrieved for the i-th tested head rotation vector.

Afterward, **SensitivityMapping** focuses on mapping the maximum among absolute mechanical gains of the semicircular duct system, per rotation (abridged as 'maximal mechanical gain'). To produce the corresponding sensitivity map, along with another map that identify which semicircular duct generated the maximal mechanical gain that was recorded for each of the 64442 head rotation vectors being considered, it applies the following formulae:

$$G_{Max,x,\mathrm{M},h,(\theta,\varphi)} = \max_{k=1:k=6} \left\| G_{\mathrm{M},x,k,h,(\theta,\varphi)} \right\|$$

and

$$LB_{Max,x,\mathrm{M},h,(\theta,\varphi)} = \arg\max_{k=1:k=6} \left\| G_{\mathrm{M},x,k,h,(\theta,\varphi)} \right\|$$

where $G_{Max,x,\mathrm{M},h,(\theta,\varphi)}$ corresponds to the maximum $_{Max}$ among absolute mechanical gains that are elicited in all six semicircular ducts by the unit head rotation vector $\vec{\Omega}_{(\theta,\varphi)}$; and where $LB_{Max,x,\mathrm{M},h,(\theta,\varphi)}$ corresponds to the label $_k$ of the semicircular duct that generated the maximum absolute mechanical gain that was recorded for the unit head rotation vector $\vec{\Omega}_{(\theta,\varphi)}$.

When all 64442 head rotation vectors have been processed, **SensitivityMapping** start summarizing the spatial maps of the maximal mechanical gain by registering the values that were retrieved for unit nose-down pitch $\vec{\Omega}_{(90,90)}$, nose-up pitch $\vec{\Omega}_{(270,90)}$, right roll $\vec{\Omega}_{(0,90)}$ and left yaw $\vec{\Omega}_{(0,0)}$ head rotations. **SensitivityMapping** continues the summarization process by registering the maximum and minimum values of the maximal mechanical gain that were retrieved during the creation of the spatial maps. Finally, **SensitivityMapping** finishes summarizing the spatial maps by computing and registering the global mean, and the global standard deviation, of the values of the maximal mechanical gain that were retrieved from the 64442 analyzed head rotations. To do so, it applies the following formulae:

$$G_{Max,x,\mathrm{M},h,Mean} = \sum_{i=1}^{64442} \frac{G_{Max,x,\mathrm{M},h,i}}{64442}$$

and

$$G_{Max,x,\mathrm{M},h,Sdv} = \sqrt{\sum_{i=1}^{64442} \frac{\left(G_{Max,x,\mathrm{M},h,i} - G_{Max,x,\mathrm{M},h,Mean}\right)^2}{64442}}$$

where $G_{Max,x,\mathrm{M},h,Mean}$ corresponds to the global mean $_{Mean}$ of the values of the maximal mechanical gain that were retrieved from the 64442 analyzed head rotation vectors; where $G_{Max,x,\mathrm{M},h,Sdv}$ corresponds to the global standard deviation $_{Sdv}$ of the values of the maximal mechanical gain that were retrieved from the 64442 analyzed head rotation vectors; and where $G_{Max,x,\mathrm{M},h,i}$ corresponds to the value of the maximal mechanical gain that was retrieved for the i-th tested head rotation vector.

Next, **SensitivityMapping** focuses on mapping the maximum among absolute mechanical gains of the semicircular duct system that are weighted to take into account the fact that inhibition-wise rotations lead to saturate semicircular duct nerve activity roughly twice faster than excitation-wise

rotations[14], per rotation (abridged as 'saturating mechanical gain'). To produce the corresponding sensitivity map, along with another map that identify which semicircular duct generated the saturating mechanical gain that was recorded for each of the 64442 head rotation vectors being considered, it applies the following formulae:

$$G'_{\mathrm{M},x,k,h,(\theta,\varphi)} = \begin{cases} G_{\mathrm{M},x,k,h,(\theta,\varphi)} & if \quad G_{\mathrm{M},x,k,h,(\theta,\varphi)} < 0 \\ \dfrac{G_{\mathrm{M},x,k,h,(\theta,\varphi)}}{2} & if \quad G_{\mathrm{M},x,k,h,(\theta,\varphi)} \geq 0 \end{cases}$$

,

$$G_{Sat,x,\mathrm{M},h,(\theta,\varphi)} = \max_{k=1:k=6} \left\| G'_{\mathrm{M},x,k,h,(\theta,\varphi)} \right\|$$

and

$$LB_{Sat,x,\mathrm{M},h,(\theta,\varphi)} = \arg\max_{k=1:k=6} \left\| G'_{\mathrm{M},x,k,h,(\theta,\varphi)} \right\|$$

where $G_{Sat,x,\mathrm{M},h,(\theta,\varphi)}$ corresponds to the maximum among absolute weighted $_{Sat}$ mechanical gains that are elicited in all six semicircular ducts by the unit head rotation vector $\vec{\Omega}_{(\theta,\varphi)}$; and where $LB_{Sat,x,\mathrm{M},h,(\theta,\varphi)}$ corresponds to the label $_k$ of the semicircular duct that generated the maximum absolute weighted mechanical gain that was recorded for the unit head rotation vector $\vec{\Omega}_{(\theta,\varphi)}$.

When all 64442 head rotation vectors have been processed, **SensitivityMapping** start summarizing the spatial maps of the saturating mechanical gain by registering the values that were retrieved for unit nose-down pitch $\vec{\Omega}_{(90,90)}$, nose-up pitch $\vec{\Omega}_{(270,90)}$, right roll $\vec{\Omega}_{(0,90)}$ and left yaw $\vec{\Omega}_{(0,0)}$ head rotations. **SensitivityMapping** continues the summarization process by registering the maximum and minimum values of the saturating mechanical gain that were retrieved during the creation of the spatial maps. Finally, **SensitivityMapping** finishes summarizing the spatial maps by computing and registering the global mean, and the global standard deviation, of the values of the saturating mechanical gain that were retrieved from the 64442 analyzed head rotations. To do so, it applies the following formulae:

$$G_{Sat,x,\mathrm{M},h,Mean} = \sum_{i=1}^{64442} \frac{G_{Sat,x,\mathrm{M},h,i}}{64442}$$

and

$$G_{Sat,x,\mathrm{M},h,Sdv} = \sqrt{\sum_{i=1}^{64442} \frac{\left(G_{Sat,x,\mathrm{M},h,i} - G_{Sat,x,\mathrm{M},h,Mean}\right)^2}{64442}}$$

where $G_{Sat,x,\mathrm{M},h,Mean}$ corresponds to the global mean $_{Mean}$ of the values of the saturating mechanical gain that were retrieved from the 64442 analyzed head rotation vectors; where $G_{Sat,x,\mathrm{M},h,Sdv}$ corresponds to the global standard deviation $_{Sdv}$ of the values of the saturating mechanical gain that were retrieved from the 64442 analyzed head rotation vectors; and

where $G_{Max,x,\mathrm{M},h,i}$ corresponds to the value of the saturating mechanical gain that was retrieved for the i-th tested head rotation vector.

Then, **SensitivityMapping** focuses on mapping the standard deviation from a null gain of absolute mechanical gains of the semicircular duct system, per rotation (abridged as 'standardized mechanical gain'). To produce the corresponding sensitivity map, it applies the following formulae, for each of the 64442 head rotation vectors being considered:

$$G_{SMG,x,\mathrm{M},h,(\theta,\varphi)} = \sqrt{\sum_{k=1}^{6} \frac{G^2_{\mathrm{M},x,k,h,(\theta,\varphi)}}{6}}$$

and

$$G_{SynSMG,x,\mathrm{M},h,(\theta,\varphi)} = \sqrt{\frac{\left(G_{\mathrm{M},x,Ar,h,(\theta,\varphi)} - G_{\mathrm{M},x,Pl,h,(\theta,\varphi)}\right)^2 + \left(G_{\mathrm{M},x,Al,h,(\theta,\varphi)} - G_{\mathrm{M},x,Pr,h,(\theta,\varphi)}\right)^2 + \left(G_{\mathrm{M},x,Lr,h,(\theta,\varphi)} - G_{\mathrm{M},x,Ll,h,(\theta,\varphi)}\right)^2}{3}}$$

where $G_{SMG,x,\mathrm{M},h,(\theta,\varphi)}$ corresponds to the standard deviation from a null gain $_{SMG}$ of absolute mechanical gains that are elicited in all six semicircular ducts by the unit head rotation vector $\vec{\Omega}_{(\theta,\varphi)}$; and where $G_{SynSMG,x,\mathrm{M},h,(\theta,\varphi)}$ corresponds to the standard deviation from a null gain of absolute combined mechanical gains that are elicited in all three synergistic $_{SynSMG}$ semicircular duct pairs by the unit head rotation vector $\vec{\Omega}_{(\theta,\varphi)}$.

When all 64442 head rotation vectors have been processed, **SensitivityMapping** start summarizing the spatial maps of the standardized mechanical gain by registering the values that were retrieved for unit nose-down pitch $\vec{\Omega}_{(90,90)}$, right roll $\vec{\Omega}_{(0,90)}$ and left yaw $\vec{\Omega}_{(0,0)}$ head rotations. **SensitivityMapping** continues the summarization process by registering the maximum and minimum values of the standardized mechanical gain that were retrieved during the creation of the spatial maps. Finally, **SensitivityMapping** finishes summarizing the spatial maps by computing and registering the global mean, and the global standard deviation, of the values of the standardized mechanical gain that were retrieved from the 64442 analyzed head rotations. To do so, it applies the following formulae:

$$G_{Map,x,\mathrm{M},h,Mean} = \sum_{i=1}^{64442} \frac{G_{Map,x,\mathrm{M},h,i}}{64442}$$

and

$$G_{Map,x,\mathrm{M},h,Sdv} = \sqrt{\sum_{i=1}^{64442} \frac{\left(G_{Map,x,\mathrm{M},h,i} - G_{Map,x,\mathrm{M},h,Mean}\right)^2}{64442}}$$

where $G_{Map,x,\mathrm{M},h,Mean}$ corresponds to the global mean $_{Mean}$ of either (1) the values that were retrieved, from the 64442 analyzed head rotation vectors, for the standard deviation of absolute mechanical gains that are elicited in all six semicircular ducts $_{Map=SMG}$, or of (2) the values that were instead retrieved for the standard deviation of absolute combined mechanical gains that are elicited in all

three synergistic semicircular duct pairs $_{Map=SynSMG}$; where $G_{Map,x,\mathrm{M},h,Sdv}$ corresponds to the global standard deviation $_{Sdv}$ of the values of the standardized mechanical gain that were retrieved from the 64442 analyzed head rotation vectors; and where $G_{Map,x,\mathrm{M},h,i}$ corresponds to the value of the standardized mechanical gain that was retrieved for the i-th tested head rotation vector.

Finally, **SensitivityMapping** focuses on mapping the neurophysiological sensitivity of the semicircular duct system, *sensu* Hullar's Bubbles script[2], per rotation (abridged as 'Hullar sensitivity'). To produce the corresponding sensitivity map, it applies the following formulae, for each of the 64442 head rotation vectors being considered:

$$G_{Hu,(\theta,\varphi)} = \left\| \vec{G}_{Ar,(\theta,\varphi)} + \vec{G}_{Pr,(\theta,\varphi)} + \vec{G}_{Lr,(\theta,\varphi)} \right\| + \left\| \vec{G}_{Al,(\theta,\varphi)} + \vec{G}_{Pl,(\theta,\varphi)} + \vec{G}_{Ll,(\theta,\varphi)} \right\|$$

where

$$\vec{G}_{k,(\theta,\varphi)} = R_k \left(\vec{\Omega}_{(\theta,\varphi)} \cdot \vec{u}_k \right) \cdot \vec{u}_k$$

and where $G_{Hu,(\theta,\varphi)}$ corresponds to the sensitivity of the semicircular duct system, *sensu* Hullar's Bubbles script $_{Hu}$, expressed in terms of the effective radius of curvature that has to be accounted to estimate the total neurophysiological gain of the semicircular duct system[15], relative to the unit head angular velocity vector $\vec{\Omega}_{(\theta,\varphi)}$; where $\vec{u}_k$ corresponds to the unit vector that is normal to the maximal response plane of the semicircular duct $_k$, and which is oriented along the excitation direction following the right hand rule; where R_k corresponds to the mean radius of curvature of the semicircular duct $_k$; and where $\vec{G}_{k,(\theta,\varphi)}$ corresponds to the vector that is normal to the maximal response plane of the semicircular duct $_k$, and which has been scaled by the effective radius of curvature that was computed in either the right anterior $_{k=Ar}$, left anterior $_{k=Al}$, right posterior $_{k=Pr}$, left posterior $_{k=Pl}$, right lateral $_{k=Lr}$ or left lateral $_{k=Ll}$ semicircular duct, relative to the unit head angular velocity vector $\vec{\Omega}_{(\theta,\varphi)}$.

When all 64442 head rotation vectors have been processed, **SensitivityMapping** start summarizing the spatial maps of the Hullar sensitivity by registering the values that were retrieved for unit nose-down pitch $\vec{\Omega}_{(90,90)}$, right roll $\vec{\Omega}_{(0,90)}$ and left yaw $\vec{\Omega}_{(0,0)}$ head rotations. **SensitivityMapping** continues the summarization process by registering the maximum and minimum values of the Hullar sensitivity that were retrieved during the creation of the spatial maps. Finally, **SensitivityMapping** finishes summarizing the spatial maps by computing and registering the global mean, and the global standard deviation, of the values of the Hullar sensitivity that were retrieved from the 64442 analyzed head rotations. To do so, it applies the following formulae:

$$G_{Hu,Mean} = \sum_{i=1}^{64442} \frac{G_{Hu,i}}{64442}$$

and

$$G_{Hu,Sdv} = \sqrt{\sum_{i=1}^{64442} \frac{\left(G_{Hu,i} - G_{Hu,Mean}\right)^2}{64442}}$$

where $G_{Hu,Mean}$ corresponds to the global mean $_{Mean}$ of the values of the Hullar sensitivity that were retrieved from the 64442 analyzed head rotation vectors; where $G_{Hu,Sdv}$ corresponds to the global standard deviation $_{Sdv}$ of the values of the Hullar sensitivity that were retrieved from the 64442 analyzed head rotation vectors; and where $G_{Hu,i}$ corresponds to the value of the Hullar sensitivity that was retrieved for the i-th tested head rotation vector.

At the end of the procedure, **SensitivityMapping** output several files corresponding to the above mentioned sensitivity maps, which can be visualized using **SciLab** 5.4.0 (see tutorial in section I.i. and accompanying Fig.5). It also outputs several files containing summarized statistical information about the structure of these maps, as described above.

c. Input files

As input, **SensitivityMapping** needs the files:

SPECIESNAME_Suffix_Extended.biomecha

SPECIESNAME_Suffix.morph

with **Suffix** successively replaced by **Cor**, **MinusCor** and **PlusCor**, to be placed in the folder **/AriadneToolbox/Data/SPECIESNAME/** (see section I.v.1.).

d. Output files

SensitivityMapping outputs the files:

SPECIESNAME_Suffix.sensitivity

SPECIESNAME_Suffix_Extended.sensitivity

in the folder **/AriadneToolbox/Data/SPECIESNAME/**, with **Suffix** successively replaced by **Cor**, **MinusCor** and **PlusCor**.

It also outputs the files:

SPECIESNAME_A_B_C_D.AverageGain

SPECIESNAME_A_B_C_D_Synergistic.AverageGain

SPECIESNAME_A_B_C_D.DeviationGain

SPECIESNAME_A_B_C_D_Synergistic.DeviationGain

SPECIESNAME_A_B_C_D.MaxGain

SPECIESNAME_A_B_C_D_Mapping.MaxGain

SPECIESNAME_A_B_C_D.SatGain

SPECIESNAME_A_B_C_D_Mapping.SatGain

SPECIESNAME_A_B_C_D.StandardizedGain

SPECIESNAME_A_B_C_D_Synergistic.StandardizedGain

SPECIESNAME_A.HullarResponse

in the folder **/AriadneToolbox/Data/SPECIESNAME/SensitivityMap/**, with **A** successively replaced by **Cor**, **MinusCor** and **PlusCor**, with **B** successively replaced by **Long**, **Medium** and **Short**, with **C**

successively replaced by **Velocity** and **Acceleration**, and with **D** successively replaced by **Volume**, **Cupula** and **Cilia**.

.AverageGain files contain data, based on a model of coupled ducts, that can be visualized in the form of a Mercator projection using **SciLab** 5.4.0 (see tutorial in section I.i. and accompanying Fig.5). The resulting maps picture the spatial distribution of the average of absolute mechanical gains of the semicircular duct system, per rotation (abridged as 'average mechanical gain'), by covering the full directional range of head motion. To visualize the data, the user can choose to express the average mechanical gain in units of (1) total volume displacement of the cupulae, (2) average transverse displacement of the cupulae, or (3) average deflection of areas of the cupulae where stereocilia/kinocilia can be found, all relative to either unit angular velocity or unit angular acceleration of the head. The user can also choose to apply (1) three different lengths for the longest stereocilia/kinocilia of each *crista ampullaris*, (2) three different levels of correction for potential shrinkage and segmentation errors, and to use (3) either the mechanical gain of each semicircular duct or the combined mechanical gain of each synergistic functional pair as the basis for the computation of the average mechanical gain.

.DeviationGain files contain data, based on a model of coupled ducts, that can be visualized in the form of a Mercator projection using **SciLab** 5.4.0 (see tutorial in section I.i. and accompanying Fig.5). The resulting maps picture the spatial distribution of the standard deviation of absolute mechanical gains of the semicircular duct system, per rotation (abridged as 'standard deviation of the mechanical gain'), by covering the full directional range of head motion. To visualize the data, the user can choose to express the standard deviation of the mechanical gain in units of (1) total volume displacement of the cupulae, (2) average transverse displacement of the cupulae, or (3) average deflection of areas of the cupulae where stereocilia/kinocilia can be found, all relative to either unit angular velocity or unit angular acceleration of the head. The user can also choose to apply (1) three different lengths for the longest stereocilia/kinocilia of each *crista ampullaris*, (2) three different levels of correction for potential shrinkage and segmentation errors, and to use (3) either the mechanical gain of each semicircular duct or the combined mechanical gain of each synergistic functional pair as the basis for the computation of the standard deviation of the mechanical gain.

.MaxGain files contain data, based on a model of coupled ducts, that can be visualized in the form of a Mercator projection using **SciLab** 5.4.0 (see tutorial in section I.i. and accompanying Fig.5). The resulting maps picture the spatial distribution of the maximum among absolute mechanical gains of the semicircular duct system, per rotation (abridged as 'maximal mechanical gain'), by covering the full directional range of head motion. To visualize the data, the user can choose to express the maximal mechanical gain in units of (1) total volume displacement of the cupulae, (2) average transverse displacement of the cupulae, or (3) average deflection of areas of the cupulae where stereocilia/kinocilia can be found, all relative to either unit angular velocity or unit angular acceleration of the head. The user can also choose to apply (1) three different lengths for the longest stereocilia/kinocilia of each *crista ampullaris*, and (2) three different levels of correction for potential shrinkage and segmentation errors. In addition, '**_Mapping.MaxGain**' files contain data that can also be visualized in the form of a Mercator projection, and which identify which semicircular duct has generated the maximal mechanical gain for each of the 64442 mapped head rotation vectors.

.SatGain files contain data, based on a model of coupled ducts, that can be visualized in the form of a Mercator projection using **SciLab** 5.4.0 (see tutorial in section I.i. and accompanying Fig.5). The resulting maps picture the spatial distribution of the maximum among absolute mechanical gains of the semicircular duct system that are weighted to take into account the fact that inhibition-wise rotations lead to saturate semicircular duct nerve activity roughly twice faster than excitation-wise rotations[14], per rotation (abridged as 'saturating mechanical gain'), by covering the full directional range of head motion. To visualize the data, the user can choose to express the saturating mechanical gain in units of (1) total volume displacement of the cupulae, (2) average transverse displacement of the cupulae, or (3) average deflection of areas of the cupulae where stereocilia/kinocilia can be found, all relative to either unit angular velocity or unit angular acceleration of the head. The user can also choose to apply (1) three different lengths for the longest stereocilia/kinocilia of each *crista ampullaris*, and (2) three different levels of correction for potential shrinkage and segmentation errors. In addition, '**_Mapping.SatGain**' files contain data that can also be visualized in the form of a Mercator projection, and which identify which semicircular duct has generated the saturating mechanical gain for each of the 64442 mapped head rotation vectors.

.StandardizedGain files contain data, based on a model of coupled ducts, that can be visualized in the form of a Mercator projection using **SciLab** 5.4.0 (see tutorial in section I.i. and accompanying Fig.5). The resulting maps picture the spatial distribution of the standard deviation from a null gain of absolute mechanical gains of the semicircular duct system, per rotation (abridged as 'standardized mechanical gain'), by covering the full directional range of head motion. To visualize the data, the user can choose to express the standardized mechanical gain in units of (1) total volume displacement of the cupulae, (2) average transverse displacement of the cupulae, or (3) average deflection of areas of the cupulae where stereocilia/kinocilia can be found, all relative to either unit angular velocity or unit angular acceleration of the head. The user can also choose to apply (1) three different lengths for the longest stereocilia/kinocilia of each *crista ampullaris*, (2) three different levels of correction for potential shrinkage and segmentation errors, and to use (3) either the mechanical gain of each semicircular duct or the combined mechanical gain of each synergistic functional pair as the basis for the computation of the standardized mechanical gain.

.HullarResponse files contain data, based on a model of coupled ducts, that can be visualized in the form of a Mercator projection using **SciLab** 5.4.0 (see tutorial in section I.i. and accompanying Fig.5). The resulting maps picture the spatial distribution of the mechanical sensitivity of the semicircular duct system *sensu* Hullar's Bubbles script[2], per rotation (abridged as 'Hullar sensitivity'), by covering the full directional range of head motion. In these maps, the 'Hullar sensitivity' is expressed in units of the effective radius of curvature that has to be accounted to estimate the total neurophysiological gain of the semicircular duct system, per head angular velocity vector.

.sensitivity files contain information, based on a model of coupled ducts, about (1) the global mean, (2) the global standard deviation, (3) the maximum, and (4) the minimum of the values of (1) the saturating mechanical gain and (2) the standardized mechanical gain that were retrieved from the 64442 analyzed head rotations. All these sensitivity metrics are expressed in units of average deflection of areas of the cupulae where stereocilia/kinocilia can be found, relative to either unit angular velocity or unit angular acceleration of the head. Several sets of results are computed based on (1) three different lengths for the longest stereocilia/kinocilia of each *crista ampullaris*, and (2) three different levels of correction for potential shrinkage and segmentation errors. The

standardized mechanical gain is only built from the combined gain of each of the 3 synergistic functional pairs in these files.

.sensitivity files also specifically contain information about (1) the saturating mechanical gain and (2) the standardized mechanical gain that were retrieved from nose-down pitch, right roll and left yaw rotations, with the addition of nose-up pitch rotation for the saturating mechanical gain.

Extended versions of the **.sensitivity** files contain information, based on a model of coupled ducts, about (1) the global mean, (2) the global standard deviation, (3) the maximum, and (4) the minimum of the values of (1) the average mechanical gain, (2) the standard deviation of the mechanical gain, (3) the maximal mechanical gain, (4) the saturating mechanical gain, (5) the standardized mechanical gain, and (6) the Hullar sensitivity that were retrieved from the 64442 analyzed head rotations.

Extended versions of the **.sensitivity** files also specifically contain information about (1) the average mechanical gain, (2) the standard deviation of the mechanical gain, (3) the maximal mechanical gain, (4) the saturating mechanical gain, (5) the standardized mechanical gain, and (6) the Hullar sensitivity that were retrieved from nose-down pitch, right roll and left yaw rotations, with the addition of nose-up pitch rotation for the maximal mechanical gain, and the saturating mechanical gain.

In these extended files, when applicable, the various sensitivity metrics are either expressed in units of (1) total volume displacement of the cupulae, (2) average transverse displacement of the cupulae, (3) average deflection of areas of the cupulae where stereocilia/kinocilia can be found, or (4) effective radius of curvature that has to be accounted to estimate the total neurophysiological gain of the semicircular duct system, all relative to either unit angular velocity or unit angular acceleration of the head. Several sets of results are computed, based on (1) three different lengths for the longest stereocilia/kinocilia of each *crista ampullaris*, (2) three different levels of correction for potential shrinkage and segmentation errors, and (3) building either from the gain of each of the 6 semicircular ducts or from the combined gain of each of the 3 synergistic functional pairs.

vi. VestibularRefAuto

VestibularRefAuto is a batch file that automatically executes **DataVestibularReference** (section I.vi.1.) and **STLVestibularReference** (section I.vi.2.), in that order. To use it, double-click it, in the folder **/AriadneToolbox/Batch/**, and press any key.

VestibularRefAuto is coded in such a way that it will seek most information needed by its modules by reading a **TaxaInfo.txt** file that has to be placed at the root of the **Data** folder (see section I.i., step (2), and by looking into the name of files and folders contained in that same folder. It is then essential, to use this module, to strictly follow the naming convention for input files as described in Ariadne Manual – Data preparation, for files pertaining to the **DataVestibularReference** and **STLVestibularReference** modules, and to structure the **Data** folder accordingly.

It should be noted that **VestibularRefAuto** is not limited to analyze only one specimen at a time but can read in every specimen found in **TaxaInfo.txt**. This makes analysis of multiple specimens possible as long as naming conventions are rigorously followed.

1. DataVestibularReference

a. Description

DataVestibularReference is an executable, written in C, which uses transformation matrices outputted by **SCDSReferenceFrame** (section I.iii.8.) in order to:

- reflect landmarks, linesets and STL files describing a semicircular duct system, across the mid-sagittal plane of the head (Fig.6c)
- transfer the complete set of landmarks, linesets and STL files from the scan frame of reference to the vestibular frame of reference[6], based on a model of coupled ducts (Fig.6b).

DataVestibularReference can either be used as stand-alone software by double-clicking on **DataVestibularReference.exe**, in the folder **/AriadneToolbox/**, and following instructions appearing in the console, or as part of the batch processes **Ariadne_SCDS.bat** or **VestibularRefAuto.bat** (sections I.i & I.vi.).

b. Algorithm

Before transferring landmarks, linesets and STL files to the vestibular frame of reference, **DataVestibularReference** first needs their respective data sets to be complete for both right and left sides of the head. If the user provides **DataVestibularReference** with landmarks, linesets and STL files from only one side of the head, the software then rebuild the data sets of the other side before proceeding further. To do so, **DataVestibularReference** first reads-in the three-dimensional coordinates $\left\{ i_{i,n}, j_{i,n}, k_{i,n} \right\}$ of each point i of the inputted landmarks, linesets and STL files n. It also reads-in information about the mid-sagittal plane of the head (section I.vi. of the Ariadne Manual – Data preparation), as well as the symmetry matrix outputted by **SCDSReferenceFrame** (section I.iii.8.). Then it applies the following formula to all read points, in order to rebuild the missing sets of files:

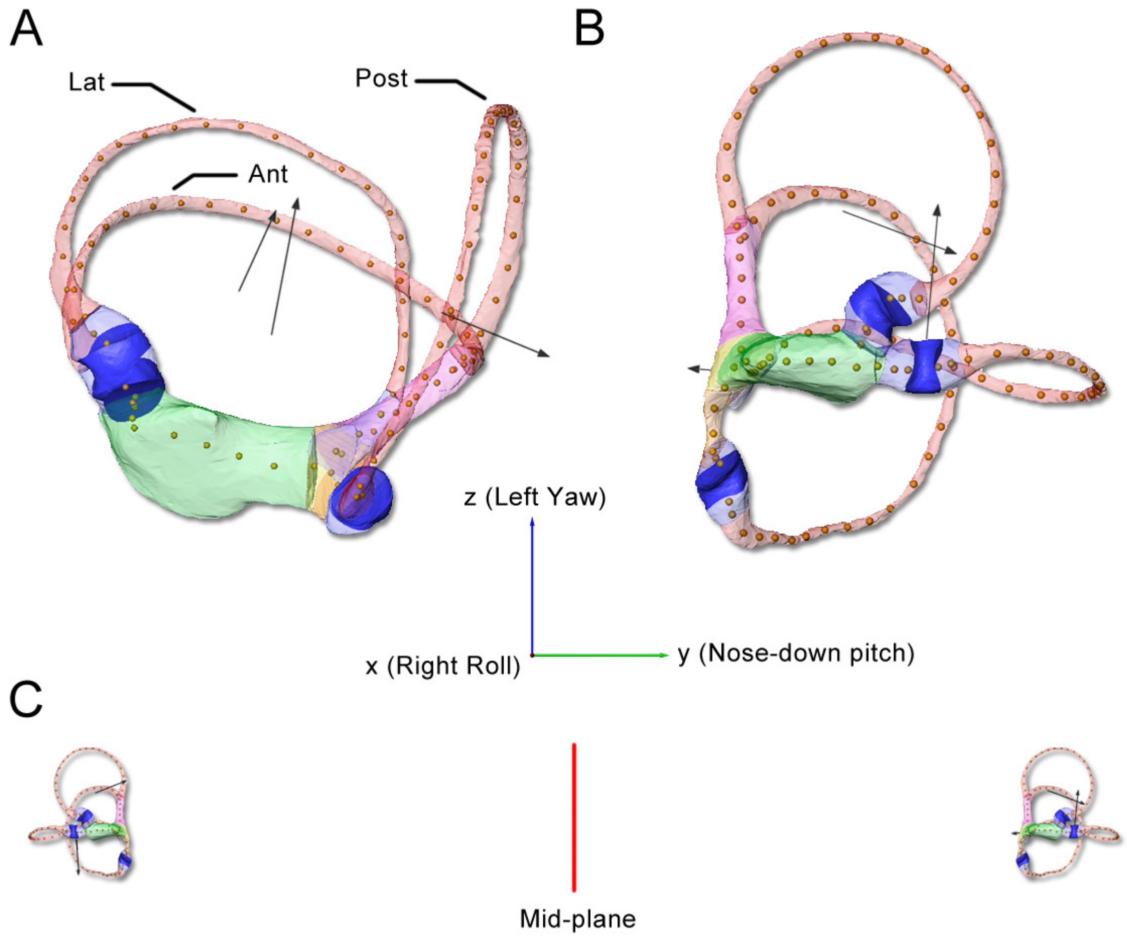

Figure 6: Left semicircular duct system of a human, composed of its STL parts, landmarks and linesets, in the scan frame of reference (A), and in the vestibular frame of reference (B), illustrating the effect of DataVestibularReference. Note that the orthonormal basis (x,y,z) corresponding to the standard viewer basis of Avizo was left unchanged between these two views, and corresponds to vestibular right roll (x), nose-down pitch (y) and left yaw (z) rotations in the vestibular frame of reference. Reflection of STL files, landmarks and linesets across the mid-sagittal plane is also illustrated (C).

$$p'_{i,n} = S(MPP - p_{i,n}) + MPP$$

where

$$S = \begin{bmatrix} \left(MP_x^2 - MP_y^2 - MP_z^2\right) & 2MP_xMP_y & 2MP_xMP_z \\ 2MP_xMP_y & \left(-MP_x^2 + MP_y^2 - MP_z^2\right) & 2MP_yMP_z \\ 2MP_xMP_z & 2MP_yMP_z & \left(-MP_x^2 - MP_y^2 + MP_z^2\right) \end{bmatrix}$$

corresponds to the matrix of reflection across the mid-sagittal plane of the head, as outputted by **SCDSReferenceFrame** (section I.iii.8.); where $\overrightarrow{MP} = \left(MP_x, MP_y, MP_z\right)$ corresponds to the unit vector that is normal to the mid-sagittal plane of the head, in the scan frame of reference; where

$$MPP = \begin{pmatrix} MPP_x \\ MPP_y \\ MPP_z \end{pmatrix}$$

corresponds to any point located on the mid-sagittal plane of the head; and where

$$p_{i,n} = \begin{pmatrix} i_{i,n} \\ j_{i,n} \\ k_{i,n} \end{pmatrix}$$

and

$$p'_{i,n} = \begin{pmatrix} i'_{i,n} \\ j'_{i,n} \\ k'_{i,n} \end{pmatrix}$$

respectively correspond to inputted and reflected i-th points of the n-th landmark, lineset or STL file, in the scan frame of reference. Note that for STL files, normal vectors are inverted after this step to retain the same inside-outside orientation as the triangles of native files.

After having rebuilt the complete sets of landmarks, linesets and STL files, **DataVestibularReference** reads the transfer matrix outputted by **BiomechaParam** (section I.v.1.) and transfers all points of every data sets from the scan frame of reference to the coupled vestibular frame of reference, by applying the following formula:

$$C'_{i,n} = P_{B''}^{B} C_{i,n}$$

where $P_{B''}^{B}$ corresponds to the passage matrix from the native scan frame of reference $B = \{\vec{x}, \vec{y}, \vec{z}\}$, to the coupled vestibular frame of reference $B'' = \{\vec{X}, \vec{Y}, \vec{Z}\}$, as outputted by **BiomechaParam** (section I.v.1.); and where

$$C_{i,n} = \begin{pmatrix} C_{x,i,n} \\ C_{y,i,n} \\ C_{z,i,n} \end{pmatrix}$$

and

$$C'_{k} = \begin{pmatrix} C'_{X,i,n} \\ C'_{Y,i,n} \\ C'_{Z,i,n} \end{pmatrix}$$

respectively correspond to the i-th points of the n-th landmark, lineset or STL file, in the scan and vestibular frames of reference.

Finally, **DataVestibularReference** creates new landmarks, linesets and STLs by registering the newly computed coordinates $\{C'_{X,i,n}, C'_{Y,i,n}, C'_{Z,i,n}\}$ of all transferred points in new files.

c. Input files

As input, **DataVestibularReference** needs the files:

rv_SPECIESNAME_Sa.stl
rv_SPECIESNAME_Sp.stl
rv_SPECIESNAME_Sl.stl
rv_SPECIESNAME_CC.stl
rv_SPECIESNAME_Aa.stl
rv_SPECIESNAME_Ap.stl
rv_SPECIESNAME_Post_Cup.stl
rv_SPECIESNAME_Al.stl
rv_SPECIESNAME_Ua.stl
rv_SPECIESNAME_Up.stl
rv_SPECIESNAME_SC.stl
rv_SPECIESNAME_Uc.stl
rv_SPECIESNAME_Ant_Cup.stl
rv_SPECIESNAME_Lat_Cup.stl

to be placed in the folder **/AriadneToolbox/Data/SPECIESNAME/ScanRef/Volumes/** (see sections I.ii. & I.iv. of the Ariadne Manual – Data preparation).

The files:

rs_SPECIESNAME_Sa.stl
rs_SPECIESNAME_Sp.stl
rs_SPECIESNAME_Sl.stl
rs_SPECIESNAME_CCa.stl
rs_SPECIESNAME_CCp.stl
rs_SPECIESNAME_Aa.stl
rs_SPECIESNAME_Ant_Cup_Ut.stl
rs_SPECIESNAME_Ant_Cup_Duct.stl
rs_SPECIESNAME_Ant_Cup_Mid.stl
rs_SPECIESNAME_Ant_Cup_Wall.stl
rs_SPECIESNAME_Post_Cup_Ut.stl
rs_SPECIESNAME_Post_Cup_Duct.stl
rs_SPECIESNAME_Ap.stl
rs_SPECIESNAME_Al.stl
rs_SPECIESNAME_UCa.stl
rs_SPECIESNAME_UCp.stl
rs_SPECIESNAME_UCl.stl
rs_SPECIESNAME_SC.stl
rs_SPECIESNAME_Post_Cup_Mid.stl
rs_SPECIESNAME_Post_Cup_Wall.stl
rs_SPECIESNAME_Lat_Cup_Ut.stl
rs_SPECIESNAME_Lat_Cup_Duct.stl
rs_SPECIESNAME_Lat_Cup_Mid.stl
rs_SPECIESNAME_Lat_Cup_Wall.stl

to be placed in the folder **/AriadneToolbox/Data/SPECIESNAME/ScanRef/Surfaces/** (see sections I.ii. & I.iv. of the Ariadne Manual – Data preparation).

The files:

rcs_SPECIESNAME_Sa.landmarkAscii
rcs_SPECIESNAME_Sp.landmarkAscii
rcs_SPECIESNAME_Sl.landmarkAscii
rcs_SPECIESNAME_CC.landmarkAscii
rcs_SPECIESNAME_Aa.landmarkAscii
rcs_SPECIESNAME_Al.landmarkAscii
rcs_SPECIESNAME_Ua.landmarkAscii
rcs_SPECIESNAME_Up.landmarkAscii
rcs_SPECIESNAME_SC.landmarkAscii
rcs_SPECIESNAME_Uc.landmarkAscii

rcs_SPECIESNAME_Ap.landmarkAscii

to be placed in the folder **/AriadneToolbox/Data/SPECIESNAME/ScanRef/Landmarks/** (see section I.iii. of the Ariadne Manual – Data preparation).

And finally the files:

SPECIESNAME.transmat

SPECIESNAME.symmat

and potentially

SPECIESNAME.midplane

to be placed in the folder **/AriadneToolbox/Data/SPECIESNAME/Matrix/** (see section I.vi. of the Ariadne Manual – Data preparation & sections I.iii.8. & I.v.1. of this manual).

d. Output files

DataVestibularReference outputs the files:

rv_SPECIESNAME_Sa.stl

rv_SPECIESNAME_Sp.stl

rv_SPECIESNAME_Sl.stl

rv_SPECIESNAME_CC.stl

rv_SPECIESNAME_Aa.stl

rv_SPECIESNAME_Ap.stl

rv_SPECIESNAME_Post_Cup.stl

lv_SPECIESNAME_Sa.stl

lv_SPECIESNAME_Sp.stl

lv_SPECIESNAME_Sl.stl

lv_SPECIESNAME_CC.stl

lv_SPECIESNAME_Aa.stl

lv_SPECIESNAME_Ap.stl

lv_SPECIESNAME_Post_Cup.stl

rv_SPECIESNAME_Al.stl

rv_SPECIESNAME_Ua.stl

rv_SPECIESNAME_Up.stl

rv_SPECIESNAME_SC.stl

rv_SPECIESNAME_Uc.stl

rv_SPECIESNAME_Ant_Cup.stl

rv_SPECIESNAME_Lat_Cup.stl

lv_SPECIESNAME_Al.stl

lv_SPECIESNAME_Ua.stl

lv_SPECIESNAME_Up.stl

lv_SPECIESNAME_SC.stl

lv_SPECIESNAME_Uc.stl

lv_SPECIESNAME_Ant_Cup.stl

lv_SPECIESNAME_Lat_Cup.stl

in the folder **/AriadneToolbox/Data/SPECIESNAME/VestRef/Volumes/**.

The files:

rs_SPECIESNAME_Sa.stl

rs_SPECIESNAME_Sp.stl

rs_SPECIESNAME_Sl.stl

rs_SPECIESNAME_CCa.stl

rs_SPECIESNAME_Ap.stl

rs_SPECIESNAME_Al.stl

rs_SPECIESNAME_UCa.stl

rs_SPECIESNAME_UCp.stl

rs_SPECIESNAME_CCp.stl

rs_SPECIESNAME_Aa.stl

rs_SPECIESNAME_Ant_Cup_Ut.stl

rs_SPECIESNAME_Ant_Cup_Duct.stl

rs_SPECIESNAME_Ant_Cup_Mid.stl

rs_SPECIESNAME_Ant_Cup_Wall.stl

rs_SPECIESNAME_Post_Cup_Ut.stl

rs_SPECIESNAME_Post_Cup_Duct.stl

ls_SPECIESNAME_Sa.stl

ls_SPECIESNAME_Sp.stl

ls_SPECIESNAME_Sl.stl

ls_SPECIESNAME_CCa.stl

ls_SPECIESNAME_CCp.stl

ls_SPECIESNAME_Aa.stl

ls_SPECIESNAME_Ant_Cup_Ut.stl

ls_SPECIESNAME_Ant_Cup_Duct.stl

ls_SPECIESNAME_Ant_Cup_Mid.stl

ls_SPECIESNAME_Ant_Cup_Wall.stl

ls_SPECIESNAME_Post_Cup_Ut.stl

ls_SPECIESNAME_Post_Cup_Duct.stl

rs_SPECIESNAME_UCl.stl

rs_SPECIESNAME_SC.stl

rs_SPECIESNAME_Post_Cup_Mid.stl

rs_SPECIESNAME_Post_Cup_Wall.stl

rs_SPECIESNAME_Lat_Cup_Ut.stl

rs_SPECIESNAME_Lat_Cup_Duct.stl

rs_SPECIESNAME_Lat_Cup_Mid.stl

rs_SPECIESNAME_Lat_Cup_Wall.stl

ls_SPECIESNAME_Ap.stl

ls_SPECIESNAME_Al.stl

ls_SPECIESNAME_UCa.stl

ls_SPECIESNAME_UCp.stl

ls_SPECIESNAME_UCl.stl

ls_SPECIESNAME_SC.stl

ls_SPECIESNAME_Post_Cup_Mid.stl

ls_SPECIESNAME_Post_Cup_Wall.stl

ls_SPECIESNAME_Lat_Cup_Ut.stl

ls_SPECIESNAME_Lat_Cup_Duct.stl

ls_SPECIESNAME_Lat_Cup_Mid.stl

ls_SPECIESNAME_Lat_Cup_Wall.stl

in the folder **/AriadneToolbox/Data/SPECIESNAME/VestRef/Surfaces/**.

And finally the files:

rcs_SPECIESNAME_Sa.landmarkAscii

rcs_SPECIESNAME_Sp.landmarkAscii

rcs_SPECIESNAME_Sl.landmarkAscii

rcs_SPECIESNAME_CC.landmarkAscii

rcs_SPECIESNAME_Aa.landmarkAscii

rcs_SPECIESNAME_Ap.landmarkAscii

lcs_SPECIESNAME_Sp.landmarkAscii

lcs_SPECIESNAME_Sl.landmarkAscii

lcs_SPECIESNAME_CC.landmarkAscii

lcs_SPECIESNAME_Aa.landmarkAscii

r**cs_SPECIESNAME_Al.landmarkAscii**

rcs_SPECIESNAME_Ua.landmarkAscii

rcs_SPECIESNAME_Up.landmarkAscii

rcs_SPECIESNAME_SC.landmarkAscii

rcs_SPECIESNAME_Uc.landmarkAscii

lcs_SPECIESNAME_Sa.landmarkAscii

lcs_SPECIESNAME_Al.landmarkAscii

lcs_SPECIESNAME_Ua.landmarkAscii

lcs_SPECIESNAME_Up.landmarkAscii

lcs_SPECIESNAME_SC.landmarkAscii

lcs_SPECIESNAME_Ap.landmarkAscii **lcs_SPECIESNAME_Uc.landmarkAscii**

in the folder **/AriadneToolbox/Data/SPECIESNAME/VestRef/Landmarks/**.

Outputted landmark, lineset and STL files are identical to inputted files in both shape and size aspects, except for potential reflection across the mid-sagittal plane of the head. However, their coordinates are here registered in the vestibular frame of reference, based on a model of coupled ducts, instead of the native scan frame of reference.

2. STLVestibularReference

a. Description

STLVestibularReference is an executable, written in C, which transfers any STL file registered in the scan frame of reference to the vestibular frame of reference[6], based on a model of coupled ducts.

STLVestibularReference can either be used as stand-alone software by double-clicking on **STLVestibularReference.exe**, in the folder **/AriadneToolbox/**, and following instructions appearing in the console, or as part of the batch processes **Ariadne_SCDS.bat** or **VestibularRefAuto.bat** (sections I.i & I.vi.).

b. Algorithm

In order to transfer inputted STL files from the scan frame of reference to the vestibular frame of reference, **STLVestibularReference** first reads-in the three-dimensional coordinates $\{i_i, j_i, k_i\}$ of each point i of the inputted STL files, as well as the transfer matrix outputted by **BiomechaParam** (section I.v.1.). Then it applies the following formula to all read points, in order to transfer the inputted STL file to the vestibular frame of reference:

$$p_i^{'} = P_{B"}^{B} p_i$$

where $P_{B"}^{B}$ corresponds to the passage matrix from the native scan frame of reference $B = \{\vec{x}, \vec{y}, \vec{z}\}$, to the coupled vestibular frame of reference $B" = \{\vec{X}, \vec{Y}, \vec{Z}\}$, as outputted by **BiomechaParam** (section I.v.1.); and where

$$p_{i,n} = \begin{pmatrix} p_{x,i} \\ p_{y,i} \\ p_{z,i} \end{pmatrix}$$

and

$$p_{i,n}^{'} = \begin{pmatrix} p_{X,i}^{'} \\ p_{Y,i}^{'} \\ p_{Z,i}^{'} \end{pmatrix}$$

respectively correspond to the i-th points of the inputted STL file, in the scan and vestibular frames of reference.

Finally, **STLVestibularReference** creates new STLs by registering the newly computed coordinates $\left\{ p'_{X,i}, p'_{Y,i}, p'_{Z,i} \right\}$ of all transferred points in new files.

c. Input files

As input, **STLVestibularReference** needs the file(s):

FILENAME(S).stl

to be placed in the folder **/AriadneToolbox/Data/SPECIESNAME/ScanRef/Other/**.

It also needs the file:

SPECIESNAME.transmat

to be placed in the folder **/AriadneToolbox/Data/SPECIESNAME/Matrix/** (see section I.v.1.).

d. Output files

STLVestibularReference outputs the file(s):

FILENAME(S).stl

in the folder **/AriadneToolbox/Data/SPECIESNAME/VestRef/Other/**.

Outputted STL files are identical to inputted STL files in both shape and size aspects, but their coordinates are here registered in the vestibular frame of reference, based on a model of coupled ducts, instead of the native scan frame of reference.

vii. Miscellaneous

1. DataToTable

DataToTable is a batch file that automatically launches the executable file of the same name (see below). To use it, one needs to double-click it, in the folder **/AriadneToolbox/Batch/**, and to follow instructions.

DataToTable batch is coded in such a way that it will seek information needed by the executable by reading the **TaxaInfo.txt** file and looking into the name of files and folders contained in the **Data** folder. It is then essential, if one wants to use this module, to strictly follow the naming convention for input files as described below, and to structure the **Data** folder accordingly.

It should be noted that **DataToTable** is not limited to process only one specimen at a time but instead can reads in every specimen that are found in **TaxaInfo.txt**. This makes processing of multiple specimens possible as long as naming conventions are rigorously followed.

a. Description

DataToTable is an executable, written in C, which compiles morphological information provided by the module **SCDSMorphometry** (section I.iii.9.), as well as biomechanical information provided by the modules **BiomechaParam** (section I.v.1.) and **SensitivityMapping** (section I.v.3.), into a table that is much easier to handle for statistical analyzes than files initially outputted by these modules.

DataToTable can either be used as stand-alone software by double-clicking on **DataToTable.exe**, in the folder **/AriadneToolbox/**, and following instructions appearing in the console, or as part of the batch process **DataToTable.bat**.

b. Algorithm

DataToTable simply reads-in information contained in files outputted by above mentioned modules and appends morphological and biomechanical data into a summary table file. It creates nine rows per specimen, which reflect the combination between (1) three different levels of correction for potential shrinkage and segmentation errors, and (2) three different lengths for the longest kinocilia of each *crista ampullaris*. **DataToTable** also assign a specific numeric label to each specimen.

c. Abbreviations

The abbreviations used to structure the table outputted by **DataToTable** are described below:

Taxa: Name of the specimen

Label: Label assigned to the specimen

Correction: Level of correction for manual segmentation errors (Cor, MinusCor or PlusCor)

Cilia: Length of the longest stereocilia/kinocilia (Long: 90µm, Medium: 60µm, Short: 30µm)

V: Volume of endolymph contained in the part

.**Sa**: Slender part of the anterior semicircular duct

.**Sp**: Slender part of the posterior semicircular duct

.**Sl**: Slender part of the lateral semicircular duct

.**CCa**: Common crus part (anterior trajectory)

.**CCp**: Common crus part (posterior trajectory)

.**CC**: Common crus part

.**SC**: Simple crus part

.**UCa**: Utricle part of the anterior semicircular duct

.**UCp**: Utricle part of the posterior semicircular duct

.**UCl**: Utricle part of the lateral semicircular duct

.**UC**: Common part of the anterior utricle

.**Aa**: Ampulla part of the anterior semicircular duct

.**Ap**: Ampulla part of the posterior semicircular duct

.**Al**: Ampulla part of the lateral semicircular duct

.**SDa**: Anterior semicircular duct

.**SDp**: Posterior semicircular duct

.**SDl**: Lateral semicircular duct

.**Tot**: Total of all parts

SErr: Smoothing effect on volume

DErr: Splitting error on volume

L: Length of the streamline contained in the part

A: Area enclosed by the semicircular duct

R: Radius of curvature of the semicircular duct

a: Mean cross-sectional area of the part

r: Mean cross-sectional radius of the part

gma_a, **gma_p**: Ration between cross-sectional radii of the common crus and that of the slender parts of the anterior and posterior semicircular ducts

bta_a, **bta_p**, **bta_l**: Ration between cross-sectional radii of the utricular parts and that of the slender parts of the anterior, posterior and lateral semicircular ducts

dta_a, **dta_p**, **dta_l**: Ration between cross-sectional radii of the ampulla parts and that of the slender parts of the anterior, posterior and lateral semicircular ducts

ws: Wall shape drag factor of the part and displacement profile factor for the cupulae

.**Ca**: Anterior cupula

.**Cp**: Posterior cupula

.**Cl**: Lateral cupula

aC._**Md**: Surface area of the middle section of the cupula

t: Thickness of the cupula

t._**R**: Thickness of the cupula according to Rabbitt's empirical equation

d: Deflection of the stereocilia/kinocilia areas

.**l**: Long stereocilia/kinocilia region

.**m**: Medium stereocilia/kinocilia region

.**s**: Short stereocilia/kinocilia region

x.DT, **y.DT**, **z.DT**: x,y and z components of the vector normal to the semicircular duct plane

_R: Right semicircular duct

_L: Left semicircular duct

_SD: Single duct model

x.DTc, **y.DTc**, **z.DTc**: x,y and z components of the center of rotation of the semicircular duct

i.: Ipsilateral angle

s.: Synergistic angle

p.: Pitch angle

r.: Roll angle

y.: Yaw angle

var90: Variance of ipsilateral angles from 90°

dev90: Average deviation of ipsilateral angles from 90°

var180: Variance of synergistic angles from 180°

dev180: Average deviation of synergistic angles from 180°

P.: Prime direction of the semicircular duct

M: Mass parameter of the semicircular duct part

C: Damping parameter of the semicircular duct part

K: Stiffness parameter of the cupula

G: Inertial forcing parameter of the semicircular duct

T1_: Long time constant of the semicircular duct

.1, .2, .3: First, second and third values

T2_: Short time constant of the semicircular duct

w1_: Lower corner frequency of the semicircular duct

w2_: Upper corner frequency of the semicircular duct

w0_: Natural frequency of the semicircular duct

Bdw_: Fractional bandwidth of the semicircular duct

X_: Mechanical gain of the semicircular duct

VG_ or **_V**: Velocity gain of the semicircular duct

AG_ or **_A**: Acceleration gain of the semicircular duct

Q_: Gain expressed in terms of cupula volumic displacement

u_: Gain expressed in terms of cupula average transverse displacement

s_: Gain expressed in terms of stereocilia/kinocilia average deflection

p_: Sensitivity metric measured for pitch rotation

ndp_: Sensitivity metric measured for nose-down pitch rotation

nup_: Sensitivity metric measured for nose-up pitch rotation

r_: Sensitivity metric measured for roll rotation

y_: Sensitivity metric measured for yaw rotation

6_: Sensitivity metric based on the mechanical gain of each individual semicircular duct

3_: Sensitivity metric based on the combined mechanical gain of each functional pair

GA_: Spatial average of the sensitivity metric

GS_: Spatial standard deviation of the sensitivity metric

GM_: Spatial maximum of the sensitivity metric

Gm_: Spatial minimum of the sensitivity metric

AvG_: Average mechanical gain

SdvG_: Standard deviation of the mechanical gain

MaxG_: Maximal mechanical gain

SatG_: Saturating mechanical gain

StdG_: Standardized mechanical gain

HuS_: 'Hullar Sensitivity'

Shd_S and **shw_S**: Shrinkage factor of the slender ducts cross-section diameter and perimeter

Shd_A and **shw_A**: Shrinkage factor of the ampullae cross-section diameter and perimeter

Shd_U and **shw_U**: Shrinkage factor of the utricular part cross-section diameter and perimeter

Shd_C and **shw_C**: Shrinkage factor of the common crus cross-section diameter and perimeter

SMS: Strength of the shrinkage correction

T: Body temperature

rho_e and **rho_c**: Density of the endolymph and cupulae

mu_e and **mu_c**: Viscosity of the endolymph and cupulae

muA_e: Viscosity factor of the endolymph

Poisson: Poisson ratio of the cupulae

Young: Young modulus of the cupulae

Shear: Shear modulus of the cupulae

d. Input files

As input, **DataToTable** needs the files:

SPECIESNAME_Suffix.morph

SPECIESNAME_Suffix_Extended.biomecha

SPECIESNAME_Suffix_Extended.sensitivity

with **Suffix** replaced by **Cor**, **MinusCor** and **PlusCor** in successive sets of files, to be placed in the folder **/AriadneToolbox/Data/SPECIESNAME/** (see sections I.iii.9., I.v.1. & I.v.3.).

e. Output files

DataToTable outputs the file:

Results_Table.txt

in the folder **/AriadneToolbox/Data/**.

This file is a table that contains information about all morphological and biomechanical data derived from the modules **SCDSMorphometry**, **BiomechaParam** and **SensitivityMapping**, and which considers the combination of (1) three different levels of correction for potential shrinkage and segmentation errors, and of (2) three different lengths for the longest stereocilia/kinocilia of each *crista ampullaris* for each specimen.

References

1. David, R., Stoessel A., Berthoz, A., Spoor, F. & Bennequin, D. Assessing morphology and function of the semicircular duct system, introducing new in-situ visualization and software toolbox.
2. Malinzak, M. D., Fay, R. F. & Hullar, T., E. Locomotor head movements and semicircular canal morphology in primates. *Proc. Natl. Acad. Sci. U.S.A*. (2012) doi:10.1073/pnas.1206139109
3. Berlin, J.C., Kirk, E.C. & Rowe, T.B. Functional implications of ubiquitous semicircular canal non-orthogonality in mammals. PloS ONE. **8(11)**: e79585. doi:10.1371/journal.pone.0079585
4. Highstein, S.M., Fay, R.R., & Popper, A.N. *The Vestibular System*. (Springer-Verlag, New York, 2004).
5. Ghanem, T.A., Rabbitt, R.D. & Tresco, P.A. Three-dimensional reconstruction of the membranous vestibular labyrinth in the toadfish, *Opsanus tau*. Hearing Research. **124**, 27-43 (1998).
6. David, R. et al. Motion from the past. A new method to infer vestibular capacities of extinct species. *C. R. Palevol*, **9**, 397-410 (2010).
7. Macdonald, J. A. & Montgomery, J. C. The Sensory Biology of Notothenioid Fish. In *Biology of Antarctic Fish* (di Prisco, G. et al, eds.). Springer-Verlag Berlin Heidelberg. 145-162 (1991).
8. Oman, C.M., Marcus, E.N., & Curthoys, I.S. The influence of semicircular canal morphology on endolymph flow dynamics. An anatomically descriptive mathematical model. *Acta Otolaryngol*. **103**, 1-13 (1987).
9. Rabbitt, R.D. Directional coding of three-dimensional movements by the vestibular semicircular canals. *Biol Cybern*. **80**, 417-431 (1999).
10. Blanks, R.H.I., Curthoys, I.S. & Markham, C.H. Planar relationships of the semicircular canals in man. *Acta Otolaryngol*. **80**, 185-196 (1975).
11. Selva, P., Oman, C. M., & Stone, H. A. Mechanical properties and motion of the cupula of the human semicircular canal. *J Vestib Res*. **19**, 95-110 (2009).
12. Dohlman, G. F. The shape and function of the cupula. *J Laryngol Otol*. **83**, 43-53 (1969).
13. Fernandez, C., & Goldberg, J.M. Physiology of peripheral neurons innervating semicircular canals of the squirrel monkey. II. Response to sinusoidal stimulation and dynamics of peripheral vestibular system. J. Neurophysiol. **34**, 661-675 (1971).
14. Rüsch, A. & Thurm, U. Cupula displacement, hair bundle deflection, and physiological responses in the transparent semicircular canal of young eel. *Pflugers Arch*. **413**, 533-545 (1989).

15. Yang, A. & Hullar, T. E. Relationship of semicircular canal size to vestibular-nerve afferent sensitivity in mammals. *J Neurophysiol*. **98**, 3197-3205 (2007).

Ariadne Manual

Data preparation

Version 1.0

Romain David, Alexander Stoessel, Alain Berthoz, Fred Spoor and Daniel Bennequin

Max Planck Institute for Evolutionary Anthropology, Leipzig, Germany
Laboratoire de Physiologie de la Perception et de l'Action, Paris, France
Museum National d'Histoire Naturelle, Paris, France
University College London, United Kingdom

July 1th, 2016

First published as Supplementary Information of David et al (submitted)[1].

About this document

The Ariadne Manual is part of the documentation of Ariadne Toolbox, and assumes a basic understanding of semicircular duct morphology and function. It presents step-by-step tutorials to help the user preparing raw input data, explains how to use each module of Ariadne Toolbox, and provides detailed information about algorithms and equations implemented in these modules, including structure and format of related files. Information presented in this manual corresponds to Ariadne Toolbox 1.0 for Windows NT platforms. Latest documentations and executables of Ariadne are available at:

http://www.earbank.org

Copyright information

Ariadne Toolbox is distributed free of charge if used for non-commercial purposes, and is provided without any warranty. To be fully functional, Ariadne needs the software packages Elmer, Gmsh and Scilab to be installed on the same computer. These can be downloaded without costs from their respective websites:

Elmer: https://www.csc.fi/web/elmer

Gmsh: http://geuz.org/gmsh/

Scilab: http://www.scilab.org/fr

Information about the software can be found in their respective manuals. Ariadne Toolbox is known to work with Gmsh 2.8.5, Elmer 8.0 and Scilab 5.4.0. It may also work with more recent versions but this has not been tested. Information and specifications given in this document are believed to be true and accurate at the time of writing, but reports of any errors and typos as well as general comments are welcome, and can be submitted at http://www.earbank.org.

Table of Contents

I. Preparation of input data 4

i. Segmenting the semicircular duct system 4

ii. Splitting the semicircular duct system into parts 7

iii. Registering the coordinates of the central streamlines 17

iv. Building three-dimensional models of the cupulae 31

v. Preparing the finite element analyses of the cupulae 50

vi. Registering the mid-sagittal plane of the vestibular frame of reference 59

References 64

I. Preparation of input data

In order to analyze semicircular duct morphology and function using Ariadne Toolbox, input data have to be prepared following specific protocols. The tutorials presented below describe these protocols, and are formatted to be used with Geomagic Studio 12 and Avizo 7.1. However, other software may also be used as long as the main guidelines and naming conventions are followed.

i. Segmenting the semicircular duct system

As the first step the semicircular duct system has to be segmented, isolating the membranous labyrinth from the surrounding tissues, and excluding the saccule and the cochlear duct. After CT scanning a stained petrosal or head[1] the areas representing endolymph have to be delineated in each cross-sectional image of the stack, being careful to not include surrounding membranes into the selection. This can be done with any segmentation software, but the instructions given here are based on Avizo 7.1, using its lasso tool in conjunction with a Wacom screen-tablet for manual segmentation.

Two problems should be avoided when segmenting the semicircular duct system. The first problem concerns filling of the full endolymphatic volume (Fig.1). When segmentation is done manually this is rarely an issue. However, areas may remain unfilled in the case of automatic segmentation using isosurface and/or threshold values. Such unfilled areas result in triangulated surfaces inside the three-dimensional (3D) model of the semicircular duct system and these corrupt endolymph volume calculations, leading to incorrect morphological and biomechanical results.

The second potential problem concerns the segmentation of the areas around the *crista ampullaris* of each semicircular duct. The cupula is often not visible in images of stained petrosals and often shrunk when shown. Therefore, we rather use the extrusion (3D upward projection) of the shape of the *crista ampullaris* towards the opposite side of the ampulla as the model of the cupula for the analyses (see section I.iv.). For this method to work, the cupula (if any) as well as the subcupular space (including the stereocilia/kinocilia layer) need to be added to the endolymphatic volume around the *crista ampullaris* (Fig.2). Poorly filled areas around the *crista ampullaris* lead to errors when estimating the mechanical sensitivity of the deflecting cupula and the long time constant of the corresponding semicircular duct (see section I.vi.1. of Ariadne Manual – Modules description).

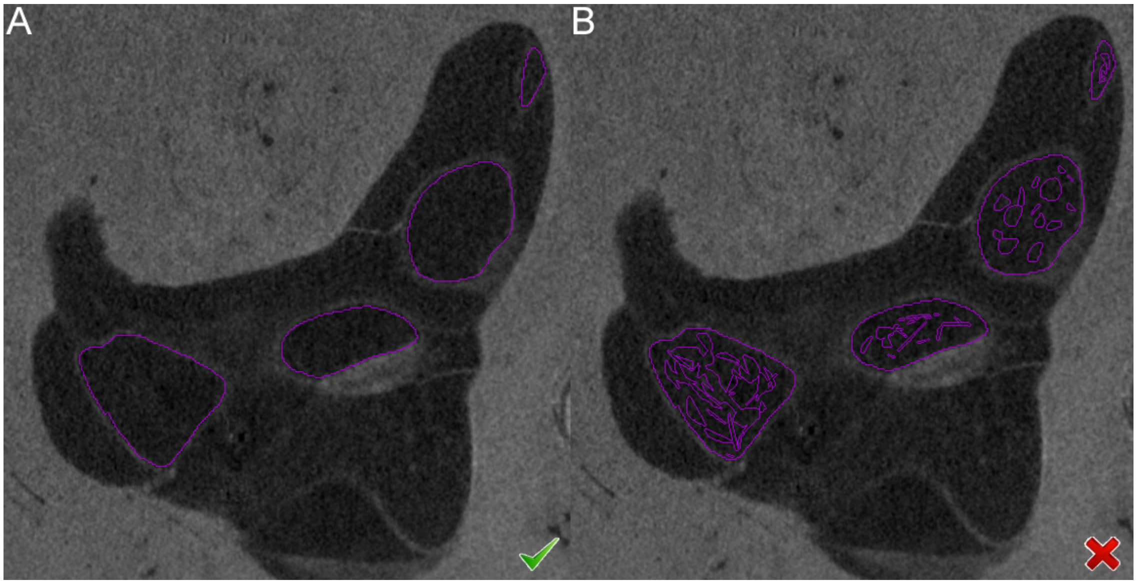

Figure 1. Full (A) and incomplete (B) filling of the endolymphatic volume of the semicircular duct system, as seen on a CT image of a stained inner ear. The unfilled areas are the consequence of automatic, threshold-based segmentation.

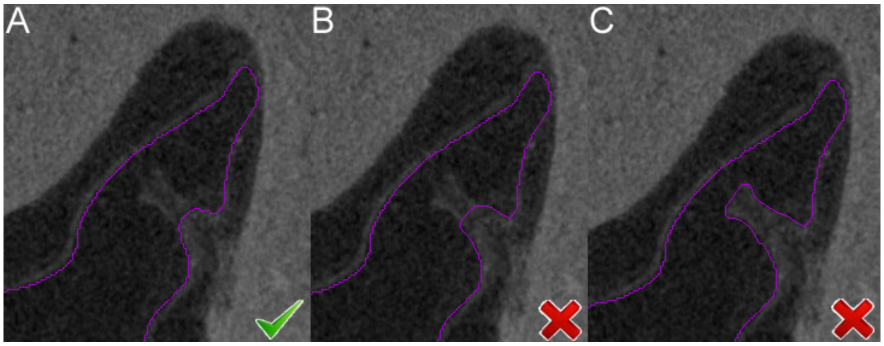

Figure 2. Segmentation of the ampullary endolymphatic volume. A. the correct area is selected. B. the subcupular space is erroneously excluded. C. the cupula and subcupular space are incorrectly excluded. The cupula shown here is artificially shrunk because of dehydration and *in vivo* reached the top of the ampulla.

When segmentation of areas filled with endolymph is complete, a raw 3D surface of the semicircular duct system needs to be computed (i.e. smoothing or triangle reduction procedures are not allowed at this stage) and saved as a '**_Control.stl**' file in ASCII format (Fig.3). In Avizo 7.1 this is done following the following steps (menu selections in brackets):

1.
   a. Right-click the [Label Field] object that represents the segmented semicircular duct system.
   b. Select [Compute>Generate Surface].
   c. In [Smoothing Type], select [None] and click [Apply].

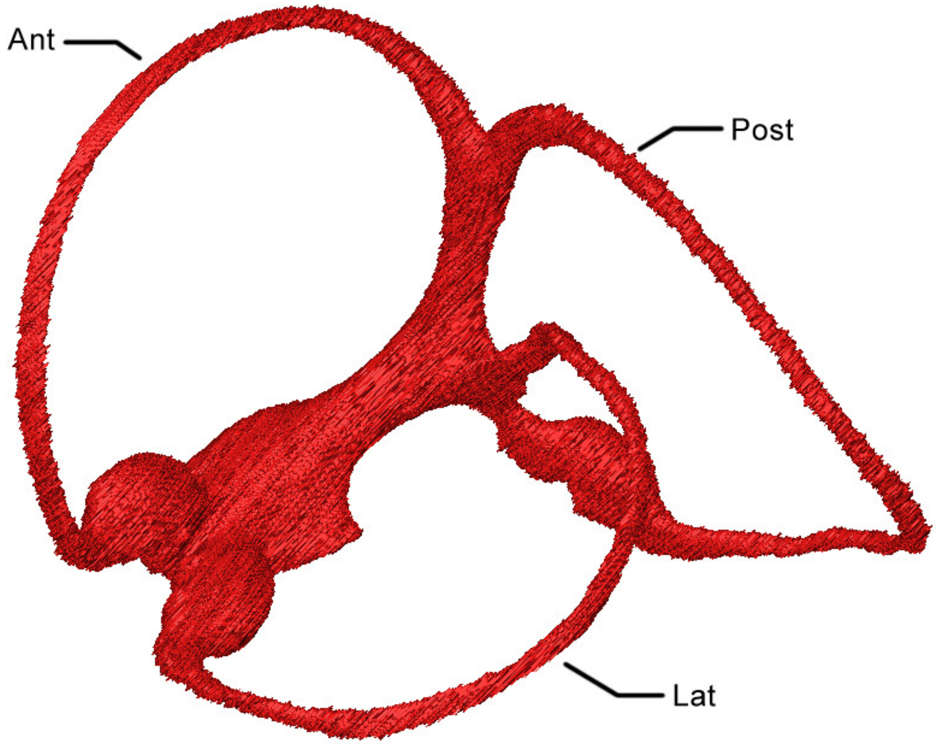

Figure 3. Three-dimensional (3D) segmentation of the left semicircular duct system of a human, as registered in the '_Control.stl' file. Ant: anterior semicircular duct, Post: posterior semicircular duct, Lat: lateral semicircular duct.

2. Save the generated surface as a STL ascii file, named as **rv_SPECIESNAME_Control.stl** ('**lv_**' instead of '**rv_**' for a left labyrinth), in folder **/AriadneToolbox/Data/SPECIESNAME/ScanRef/Volumes/**, where **SPECIESNAME** corresponds to a folder referring to the specimen.

ii. Splitting the semicircular duct system into parts

The second step of data preparation consists of dividing the semicircular duct system into the eleven parts that will be used to create 12 surface files and 11 volume files saved in STL format. These are: the slender parts of the anterior, posterior and lateral ducts, the common crus, the simple crus (*Crus membranaceum simplex*), the common, anterior and posterior utricular parts, and the anterior, posterior and lateral ampullary parts (Fig. 4).

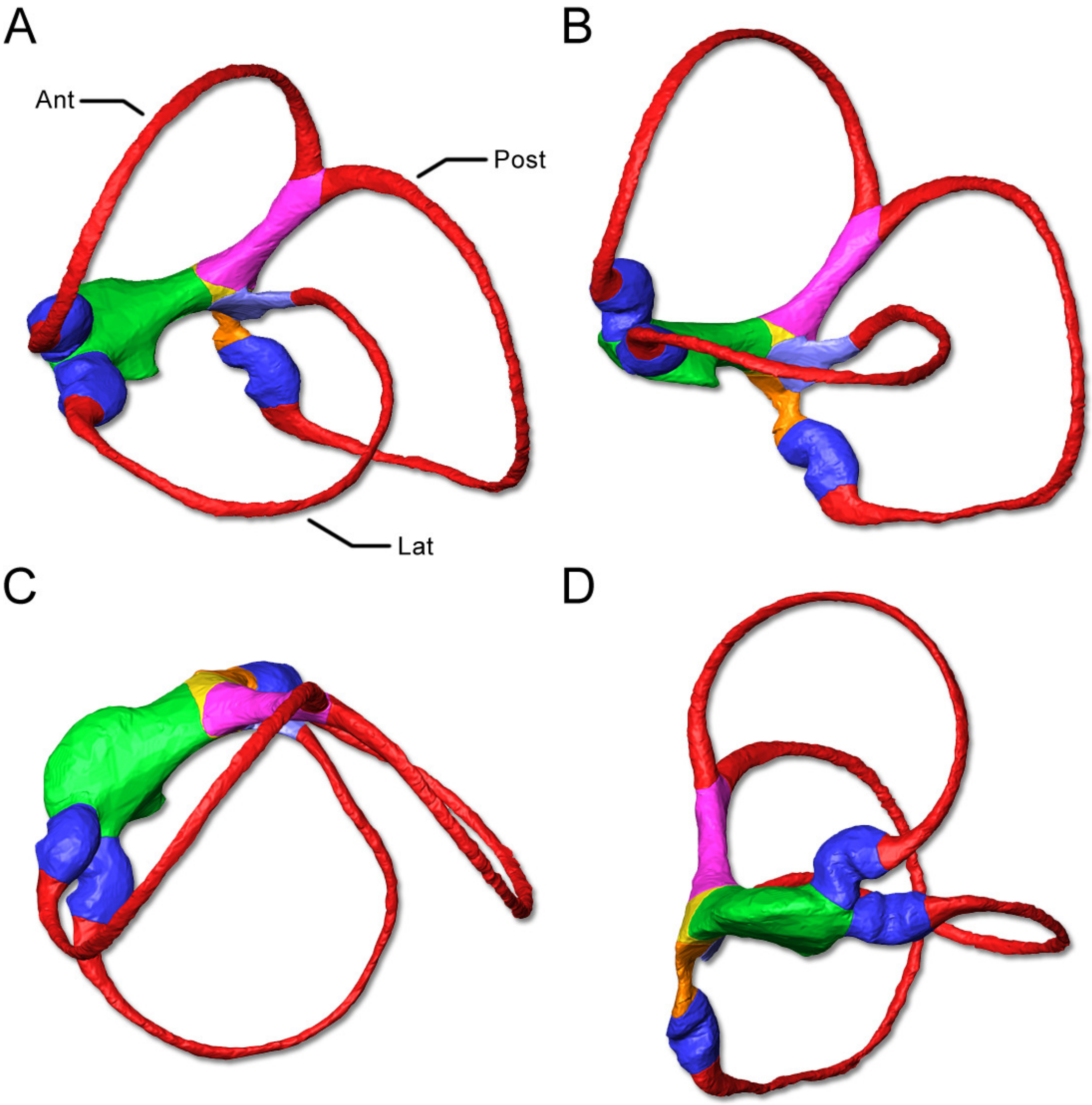

Figure 4. Isometric (A), lateral (B), dorsal (C) and frontal (D) views of the left semicircular duct system of a human, color-coding the various volume parts that need to be defined. The slender ducts are given color in red, the ampullae color in blue, the common crus color in pink, the simple crus in light blue, the anterior utricle color in green, the posterior utricle color in orange and the common utricle color in yellow. Ducts identified as in Figure 3.

As indicated by their names, the volume STL files will be used to compute how the volume of endolymph is distributed inside the semicircular duct system (see section I.iii.1. of Ariadne Manual – Modules description), which in turn will be used to compute the mean cross-sectional area of each part of the semicircular ducts (see section I.iii.5 of Ariadne Manual – Modules description). The surface STL files will be used to compute the surface areas of the inner walls of the semicircular duct parts (see section I.iii.2. of Ariadne Manual – Modules description), which in turn will be used to compute the mean wall shape drag factor[3] of each semicircular duct part (see section I.iii.5. of Ariadne Manual – Modules description). The STL volume and surface files can be obtained in Geomagic Studio 12 using the following procedure:

1.
   a. Click [Geomagic>Import], select the file **/AriadneToolbox/Data/SPECIESNAME/ScanRef/Volumes/rv_SPECIESNAME_Control.stl** and click [Open].
   b. Specify the units that were used during the segmentation process and click [OK].
   c. Decline using the [Mesh Doctor].
2.
   a. Click [Polygons>Repair>Decimate].
   b. In [Target Triangle Count] enter '250000'.
   c. Deselect [Fix Boundaries].
   d. Select [Curvature Priority], using [Max].
   e. Select [Mesh Priority] using [Max].
   f. In [Maximum Aspect Ratio], select [Edge/Edge] and enter '3.0' in the textbox.
   g. In [Maximum Aspect Ratio], select [Edge/Height] and enter '3.5' in the textbox; click [Apply], then [OK].
3.
   a. Click [Polygons>Smooth>Relax].
   b. In [Parameters], set [Smoothness Level] and [Curvature Priority] at [Max] and [Strength] at [Min].
   c. Deselect [Fix Boundaries].
   d. In the textbox of [Deviation Tolerance], enter the highest possible number and click [Apply], then [OK] (deviation statistics can be checked in [Deviations]).
4.
   a. Click [Polygons>Smooth>Remove Spikes].
   b. In [Parameters], set [Smoothness Level] at '25' and click [Apply].
   c. If the smoothing is too aggressive, reduce [Smoothness Level] (never increase it).
   d. When the smoothing level seems right, click [OK].
5.
   a. Click [Polygons>Repair>Mesh Doctor], select [Non-Manifold Edges], [Self-Intersections], [Highly Creased Edges] and [Small Holes].
   b. Deselect the rest and click [Apply], then [OK].
6.

   In the [Model Manager], right-click on the smoothed surface model and rename it as **SCDS** (Fig. 5).

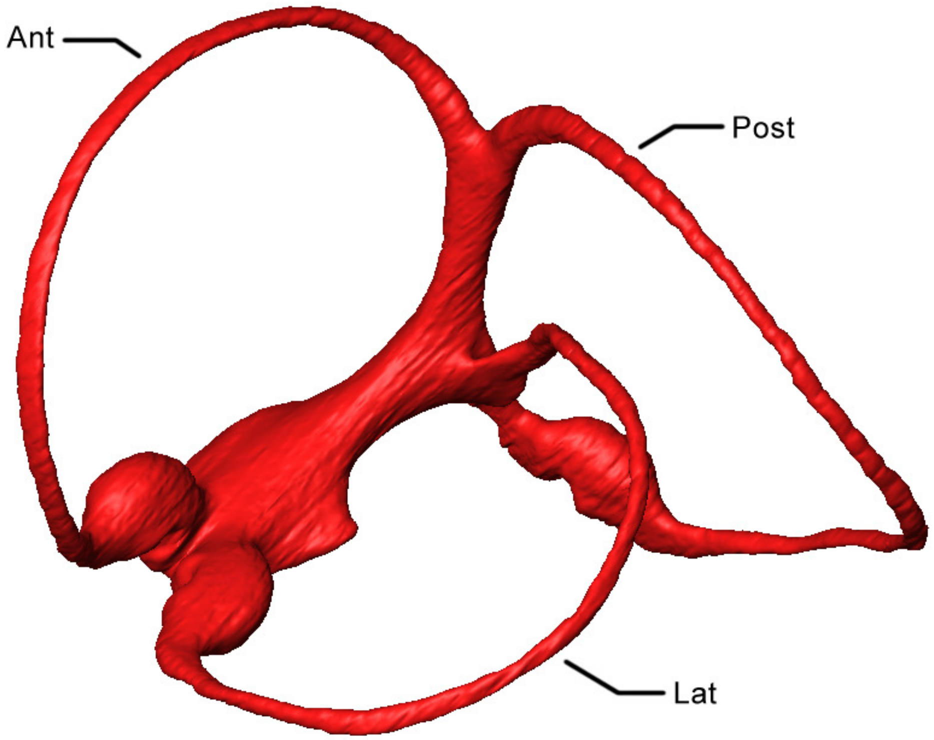

Figure 5. Smoothed 3D model of the left semicircular duct system of a human as registered in the '_SCDS.stl' file. Ducts identified as in Figure 3. The potential volume loss induced by smoothing is controlled by the use of the '_Control.stl' file in the subsequent analyses.

7. Save the generated surface as a STL ascii file, named as **r_SPECIESNAME_SCDS.stl** ('**l_**' instead of '**r_**' for a left labyrinth), in folder **/AriadneToolbox/Data/SPECIESNAME/ScanRef/Other/**.

8.
   a. Click [Polygons>Boundaries>Create>Boundary From Spline].
   b. In the [Graphics] window, define the boundary between the slender part of the anterior duct and the anterior ampulla (Fig.6A) and click [Apply].
   c. Define the boundary between the slender part of the posterior duct and the posterior ampulla (Fig.6B) and click [Apply].
   d. Define the boundary between the slender part of the lateral duct and the lateral ampulla (Fig.6C) and click [Apply].
   e. Define the boundary between the slender part of the lateral duct and the simple crus (Fig.6D) and click [Apply].
   f. Define the boundary between the slender part of the posterior duct and the common crus (Fig.6E) and click [Apply].
   g. Define the boundary between the slender part of the anterior duct and the common crus (Fig.6F) and click [Apply].
   h. Define the boundary between the anterior ampulla and the anterior utricle (Fig.6G) and click [Apply].

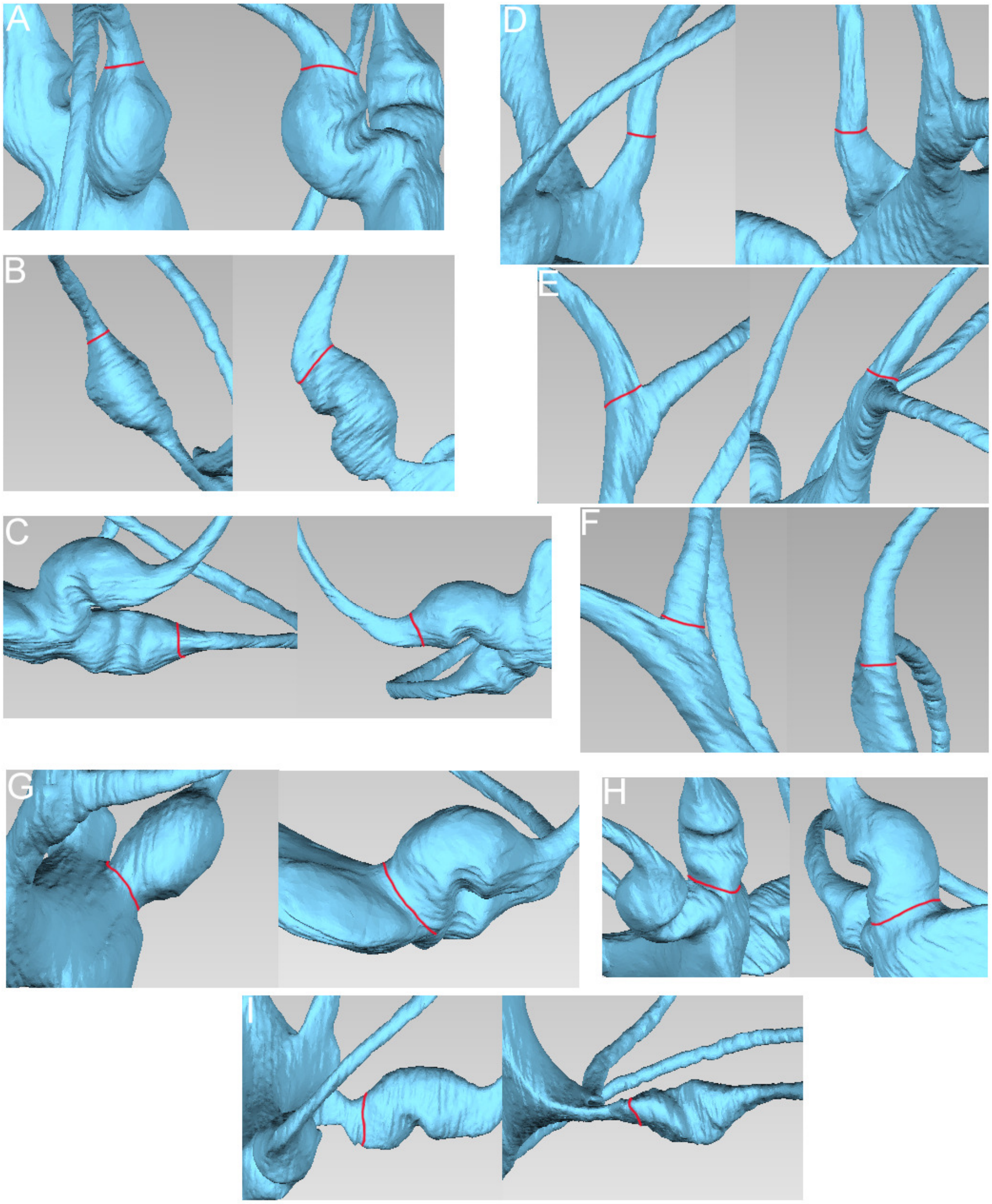

Figure 6. Details of a 3D model of the left semicircular duct system of a human, with red lines indicating the position of the boundaries used to divide the semicircular duct system in parts. (A-C) between the ampullae and the slender parts of the anterior, posterior and lateral semicircular ducts, respectively; (D) between the lateral slender duct part and the simple crus; (E, F) between the common crus and the slender parts of the anterior and posterior semicircular ducts respectively; (G, H) between the anterior utricle and the ampullae of the anterior and lateral semicircular ducts respectively; (I) between the posterior ampulla and the posterior utricle.

i. Define the boundary between the posterior ampulla and the posterior utricle (Fig.6H) and click [Apply].
j. Define the boundary between the lateral ampulla and the anterior utricle (Fig.6I) and click [Apply], then [OK].

9.
a. In the [Model Manager], right-click on the **SCDS** model and select [Duplicate].
b. Do it six times.
c. Right-click on each of the six copied models and rename them as **Sa**, **Sp**, **Sl**, **Aa**, **Ap** and **Al** respectively.

10.
Click [Select>Mode>Selection Mode>Select Visible Only] and make sure to deselect [Select>Mode>Selection Mode>Select Backfaces].

11.
a. In the [Model Manager], select the **Sa** model.
b. In the [Graphics] window, click anywhere on the slender part of the anterior semicircular duct.
c. Click [Select>Data>Select Components>Bounded Components].
d. Click [Select>Data>Reverse] then press the [Del] key (Fig.7).
e. Do the same for the **Sp**, **Sl**, **Aa**, **Ap** and **Al** models by clicking anywhere on the slender part of the posterior and lateral semicircular ducts and on the anterior, posterior and lateral ampullae respectively.

12.
a. In the [Model Manager], select the **SCDS** model.
b. In the [Graphics] window, click anywhere except on the slender parts and ampullae.
c. Click [Select>Data>Select Components>Bounded Components].
d. Click [Select>Data>Reverse] then press the [Del] key.

13.
a. In the [Model Manager], right-click on all surface models and hide them except for the **SCDS** model.
b. Select the **SCDS** model.
c. Click [Polygons>Boundaries>Create>Boundary From Spline].
d. In the [Graphics] window, define the boundary between the anterior utricle and the common utricle (Fig.8A) and click [Apply].
e. Define the boundary between the simple crus and the common utricle (Fig.8B) and click [Apply].
f. Define the boundary between the common crus and the common utricle (Fig.8C) and click [Apply].
g. Define the boundary between the posterior utricle and the common utricle (Fig.8D) and click [Apply], then [OK].
h. If needed, click [Polygons>Boundaries>Remove>Remove Boundary] and click on the intersecting boundary parts in the [Graphics] window to clean the boundaries (Fig.9).

14.
a. In the [Model Manager], right-click on the **SCDS** model and select [Duplicate].
b. Do it four times.

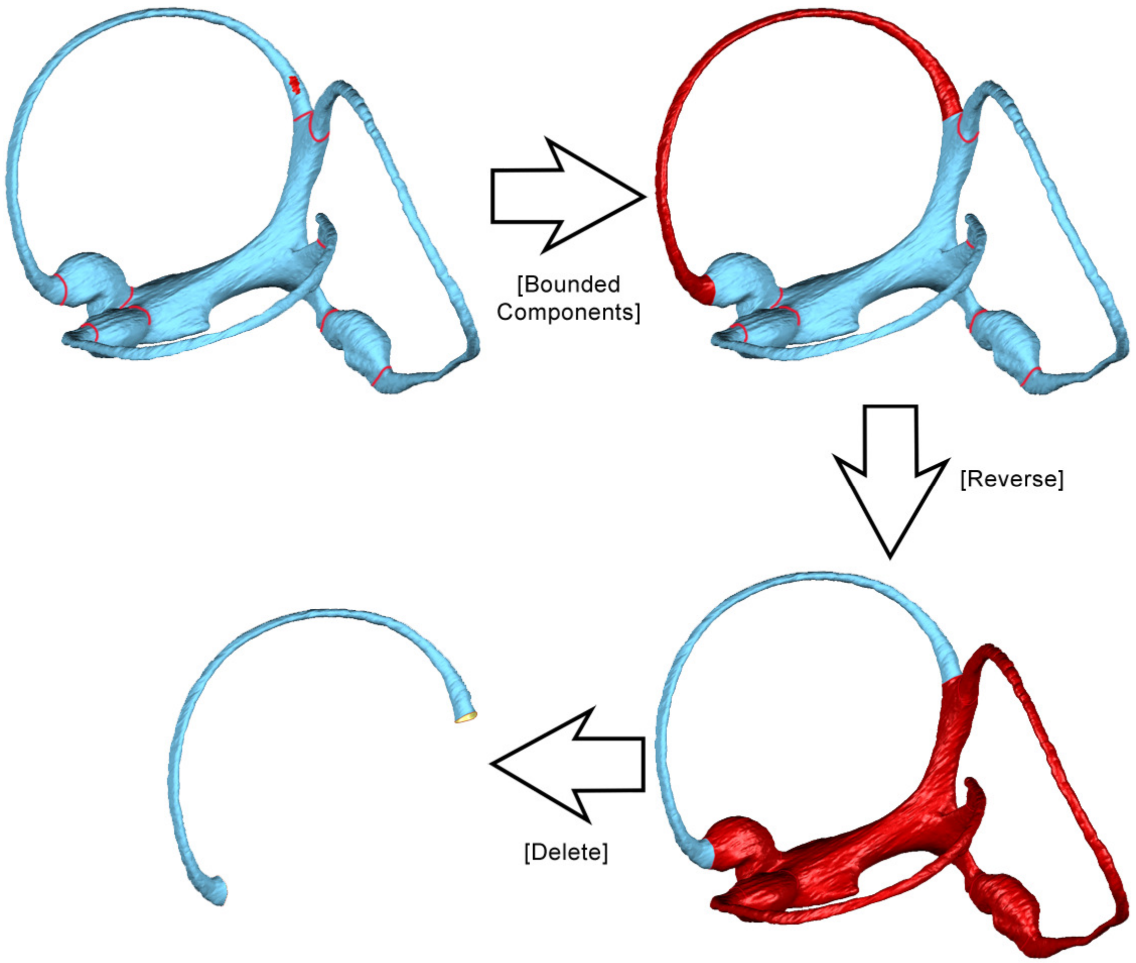

Figure 7. 3D model of the left semicircular duct system of a human, illustrating the basic procedure to obtain individual, non-overlapping parts.

c. Right-click on the original and the four copied models and rename them as **CC**, **Ua**, **Up**, **SC** and **Uc** successively.

15.

a. In the [Model Manager], select the **Ua** model.
b. In the [Graphics] window, click anywhere on the anterior utricle.
c. Click [Select>Data>Select Components>Bounded Components].
d. Click [Select>Data>Reverse] then press the [Del] key.
e. Do the same for the **Up**, **SC**, **Uc** and **CC** models by clicking anywhere on the posterior utricle, on the simple crus, on the common utricle and on the common crus respectively.

16.

a. In the [Model Manager], right-click on the **Sa** model and rename it as **Sa_V**.
b. Do the same for the 10 other models by renaming them **Sp_V**, **Sl_V**, **Aa_V**, **Ap_V**, **Al_V**, **Ua_V**, **Up_V**, **SC_V**, **Uc_V** and **CC_V** respectively.

17.

a. In the [Model Manager], right-click on the **Sa_V** model and select [Duplicate].

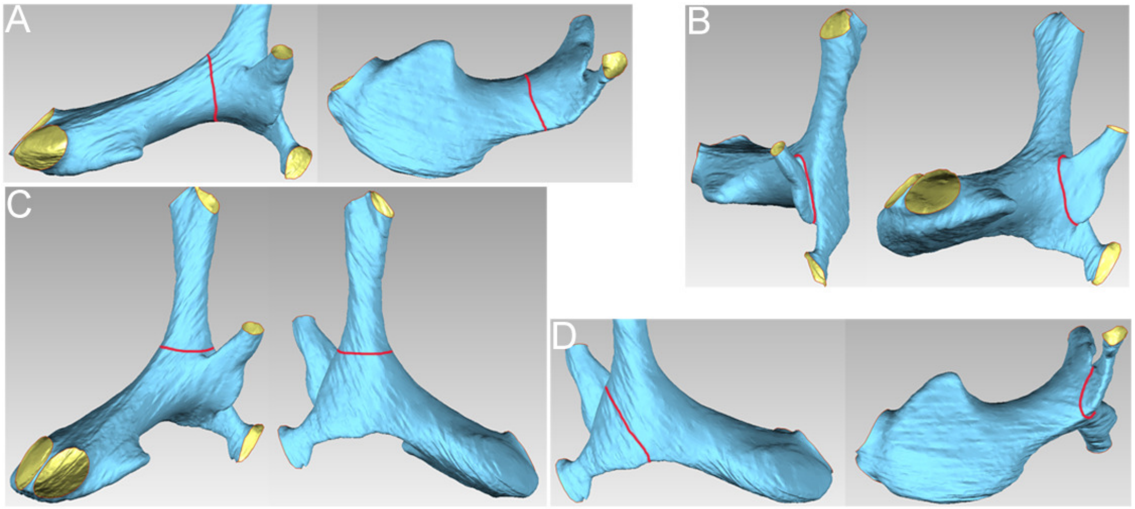

Figure 8. Details of a 3D model of the left semicircular duct system of a human, without the slender ducts and the ampullae. Red lines indicate the position of the boundaries between (A) the anterior utricle and the common utricle; (B) the simple crus and the common utricle; (C) the common crus and the common utricle; (D) the posterior utricle and the common utricle.

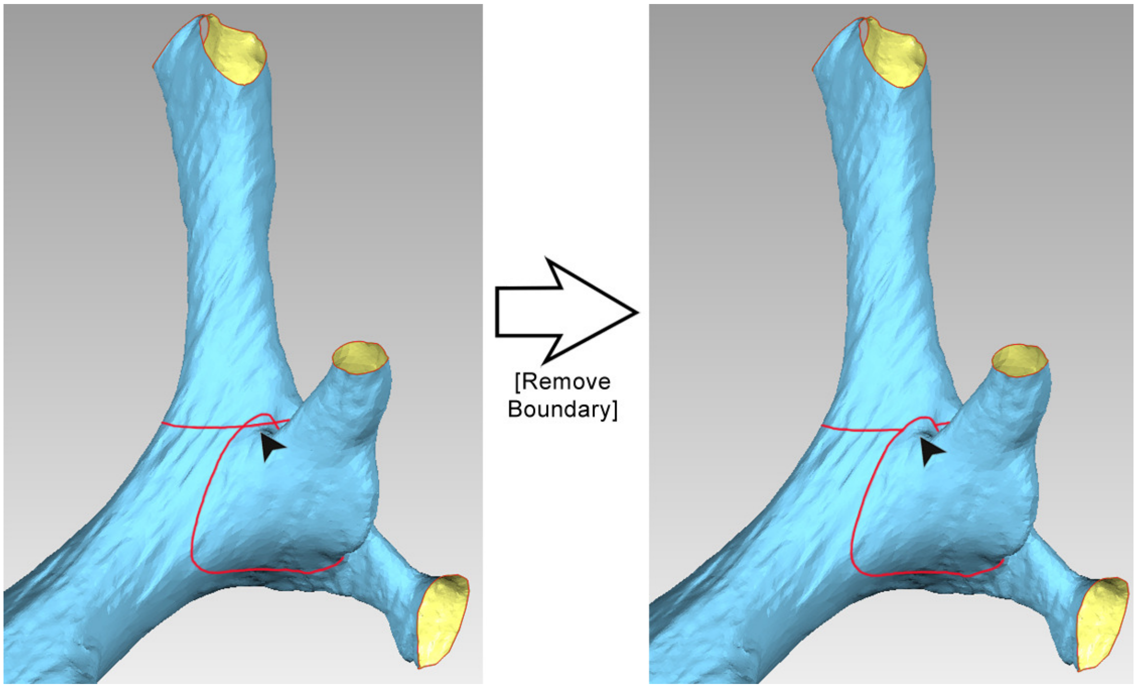

Figure 9. 3D model of a left semicircular duct system of a human, showing the boundaries between the common utricle, the common crus and the simple crus, and how to remove the overlapping section.

b. Do the same for the **Sp_V**, **Sl_V**, **Aa_V**, **Ap_V**, **Al_V** and **SC_V** models.
c. Right-click on each of the seven copied models and rename them as **Sa_S**, **Sp_S**, **Sl_S**, **Aa_S**, **Ap_S**, **Al_S** and **SC_S** respectively.

18.
a. In the [Model Manager], right-click on the **CC_V** model and select [Duplicate].
b. Do it twice.
c. Right-click on both copied models and rename them as **CCa_S** and **CCp_S** respectively.

19.
a. In the [Model Manager], select both the **Ua_V** and the **Uc_V** models (Ctrl + Left click).
b. Click [Polygons>Combine>Boolean].
c. In [Operation], enter **UCa_S** in [Name] and select [Union].
d. In [Options], select everything and click [Apply], then [OK].

20.
a. In the [Model Manager], select both the **Up_V** and the **Uc_V** models ([Ctrl] + [Left click]).
b. Click [Polygons>Combine>Boolean].
c. In [Operation], enter **UCp_S** in the [Name] textbox and select [Union].
d. In [Options], select everything and click [Apply], then [OK].

21.
a. In the [Model Manager], right-click on the **UCa_S** model and select [Duplicate].
b. Right-click on the copied model and rename it as **UCl_S**.

22.
a. Save the **Sa_S** model as a STL ascii file, named as **rs_SPECIESNAME_Sa.stl** ('**ls_**' instead of '**rs_**' for a left labyrinth), in folder **/AriadneToolbox/Data/SPECIESNAME/ScanRef/Surfaces/**.
b. Do the same for the **Sp_S**, **Sl_S**, **Aa_S**, **Ap_S**, **Al_S** and **SC_S** models, by replacing the filename suffix '**_Sa**' by '**_Sp**', '**_Sl**', '**_Aa**', '**_Ap**', '**_Al**' and '**_SC**' respectively.

23.
a. In the [Model Manager], select the **CCa_S** model.
b. Click [Polygons>Fill Holes>Fill Single] with [Flat] and [Complete] options.
c. In the [Graphics] window, click on the hole leading to the posterior slender duct (Fig.10) and click [Fill Single].

24.
a. In the [Model Manager], select the **CCp_S** model.
b. Click [Polygons>Fill Holes>Fill Single] with [Flat] and [Complete] options.
c. In the [Graphics] window, click on the hole leading to the anterior slender duct and click [Fill Single].

25.
a. In the [Model Manager], select the **UCa_S** model.
b. Click [Polygons>Fill Holes>Fill Single] with [Flat] and [Complete] options.
c. In the [Graphics] window, click on the holes leading to the lateral ampulla, the simple crus and the posterior utricle and click [Fill Single].

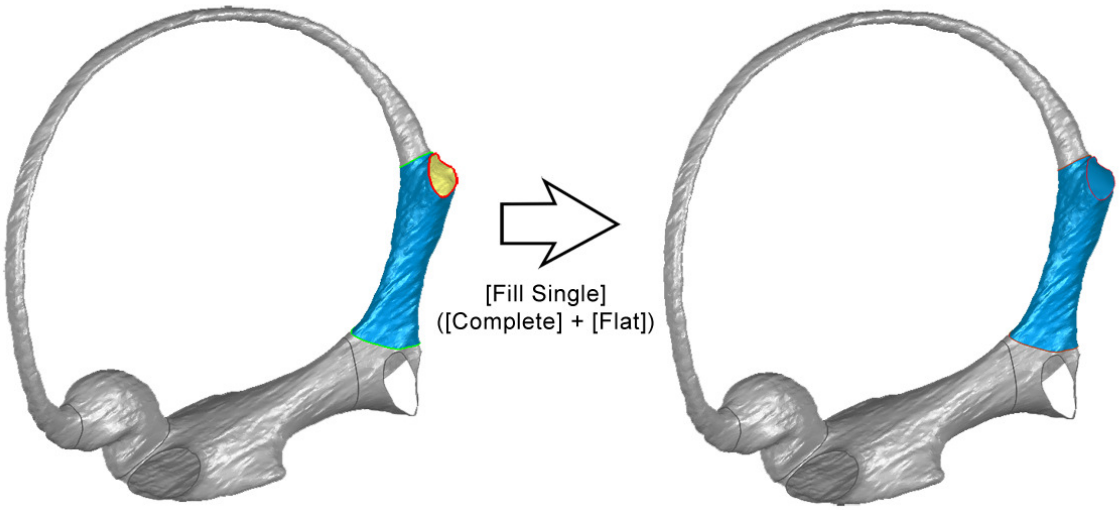

Figure 10: 3D model of a left anterior semicircular duct of a human, illustrating the filing of holes of volume and surface meshes.

26.
   a. In the [Model Manager], select the **UCl_S** model.
   b. Click [Polygons>Fill Holes>Fill Single] with [Flat] and [Complete] options.
   c. In the [Graphics] window, click on the holes leading to the anterior ampulla, the common crus and the posterior utricle and click [Fill Single].

27.
   a. In the [Model Manager], select the **UCp_S** model.
   b. Click [Polygons>Fill Holes>Fill Single] with [Flat] and [Complete] options.
   c. In the [Graphics] window, click on the holes leading to the simple crus and the anterior utricle and click [Fill Single].

28.
   a. Save the **CCa_S** model as a STL ascii file, named as **rs_SPECIESNAME_CCa.stl**, in folder **/AriadneToolbox/Data/SPECIESNAME/ScanRef/Surfaces/**.
   b. Do the same for the **CCp_S**, **UCa_S**, **UCl_S** and **UCp_S** models, by replacing the filename suffix '**_Cca**' by '**_CCp**', '**_UCa**', '**_UCl**' and '**_UCp**' respectively.

29.
   a. In the [Model Manager], select the **Sa_V**, **Sp_V**, **Sl_V**, **Aa_V**, **Ap_V**, **Al_V**, **Ua_V**, **Up_V**, **SC_V**, **Uc_V** and **CC_V** models ([Ctrl] + [Left click]).
   b. Click [Polygons>Fill Holes>Fill All] with [Flat] option and click [Apply], then [OK].

30.
   a. Save the **Sa_V** model as a STL ascii file, named as **rv_SPECIESNAME_Sa.stl**, in folder **/AriadneToolbox/Data/SPECIESNAME/ScanRef/Volumes/**.
   b. Do the same for the **Sp_V**, **Sl_V**, **Aa_V**, **Ap_V**, **Al_V**, **Ua_V**, **Up_V**, **SC_V**, **Uc_V** and **CC_V** models, by replacing the filename suffix '**_Sa**' by '**_Sp**', '**_Sl**', '**_Aa**', '**_Ap**', '**_Al**', '**_Ua**', '**_Up**', '**_SC**', '**_Uc**' and '**_CC**' respectively.

This procedure results in 11 volume and 12 surface meshes, corresponding to the main divisions of the semicircular duct system (Fig.11).

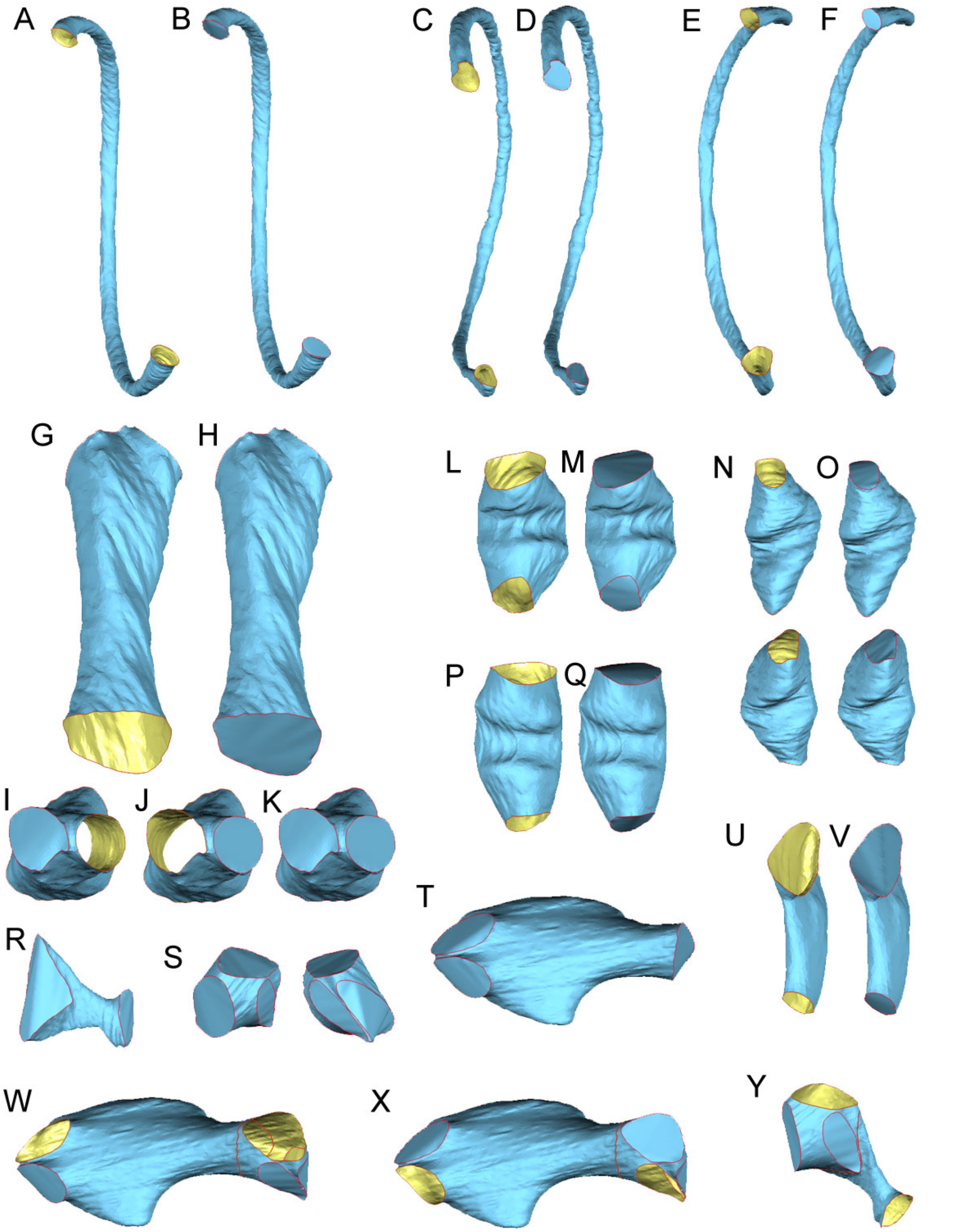

Figure 11. The 23 volume and surface STL meshes of the left semicircular duct system of a human, showing which holes were closed. Surface (A, C, E) and volume (B, D, F) meshes of the slender part of the anterior, posterior and lateral semicircular ducts respectively. Surface (I, J) and volume (K) meshes of the common crus seen in dorsal and lateral view (G, H). Surface (L, N, P) and volume (M, O, Q) meshes of the anterior, posterior and lateral ampullae respectively. Volume (R, S, T) meshes of the posterior, common and anterior utricles respectively. Surface (U) and volume (V) meshes of the simple crus. Surface (W, X, Y) meshes of the utricular part of the anterior, lateral and posterior semicircular ducts respectively.

iii. Registering the coordinates of the central streamlines

The third step of data preparation consists of registering the coordinates of the central streamlines passing through each semicircular duct. Central streamlines reflect the path of endolymph motion inside semicircular ducts under the assumption of laminar flow, and are thus essential to the computation of their 3D biomechanics[2-5]. Central streamline coordinates will be used to compute the maximal areas enclosed by the semicircular ducts, the length of each of their parts, their maximal response axes and their corresponding ipsilateral and synergistic angular relationships (see sections I.iii.3., I.iii.4. & I.iii.8. of Ariadne Manual – Modules description). Central streamlines can be computed using software for medial axis calculation (a process also known as 3D skeletonization[5]), or can be defined manually by positioning 3D landmarks inside the meshes of the semicircular ducts system (Fig.12). Here we describe how to place these 3D landmarks using Avizo 7.1:

1.
    a. Click [Project View>Open Data], select the file **/AriadneToolbox/Data/SPECIESNAME/ScanRef/Other/r_SPECIESNAME_SCDS.stl** and click [Open].
2.
    a. In [Project View], right-click the **SPECIESNAME_SCDS.stl** object and select [Display>Surface View].
    b. Click the [Surface View] object.
    c. In the [Draw Style] scroll of the [Properties] panel, select [transparent].
3.
    a. In [Project View], right-click the **SPECIESNAME_SCDS.stl** object and select [Convert>Scan Surface To Volume].
    b. Click the [Scan Surface To Volume] object.
    c. In the three [Dimensions] textboxes of the [Properties] panel, enter the number '250' and click [Apply].
4.
    a. In [Project View], right-click the **SPECIESNAME_SCDS.scanConverted** object and select [Image Morphology>Auto Skeleton].
    b. Click the [Auto Skeleton] object and click [Apply] (Fig. 13).
    c. If the skeleton looks too noisy, try imputing higher values in the [Dimensions] textboxes of the [Properties] panel of the [Scan Surface To Volume] object and redo steps 3-4.
5.
    In [Project View], delete every object by clicking on them and pressing the [Del] key, except for the **SPECIESNAME_SCDS.Smt.SptGraph** object.
6.
    a. Click [Project View>Open Data].
    b. In the folder **/AriadneToolbox/Data/SPECIESNAME/ScanRef/Volumes/**, select the files **rv_SPECIESNAME_Sa.stl**, **rv_SPECIESNAME_Sp.stl**, **rv_SPECIESNAME_Sl.stl**, **rv_SPECIESNAME_Aa.stl**, **rv_SPECIESNAME_Ap.stl**, **rv_SPECIESNAME_Al.stl**, **rv_SPECIESNAME_CC.stl**, **rv_SPECIESNAME_Ua.stl**, **rv_SPECIESNAME_Up.stl**, **rv_SPECIESNAME_SC.stl** and **rv_SPECIESNAME_Uc.stl** and click [Open].

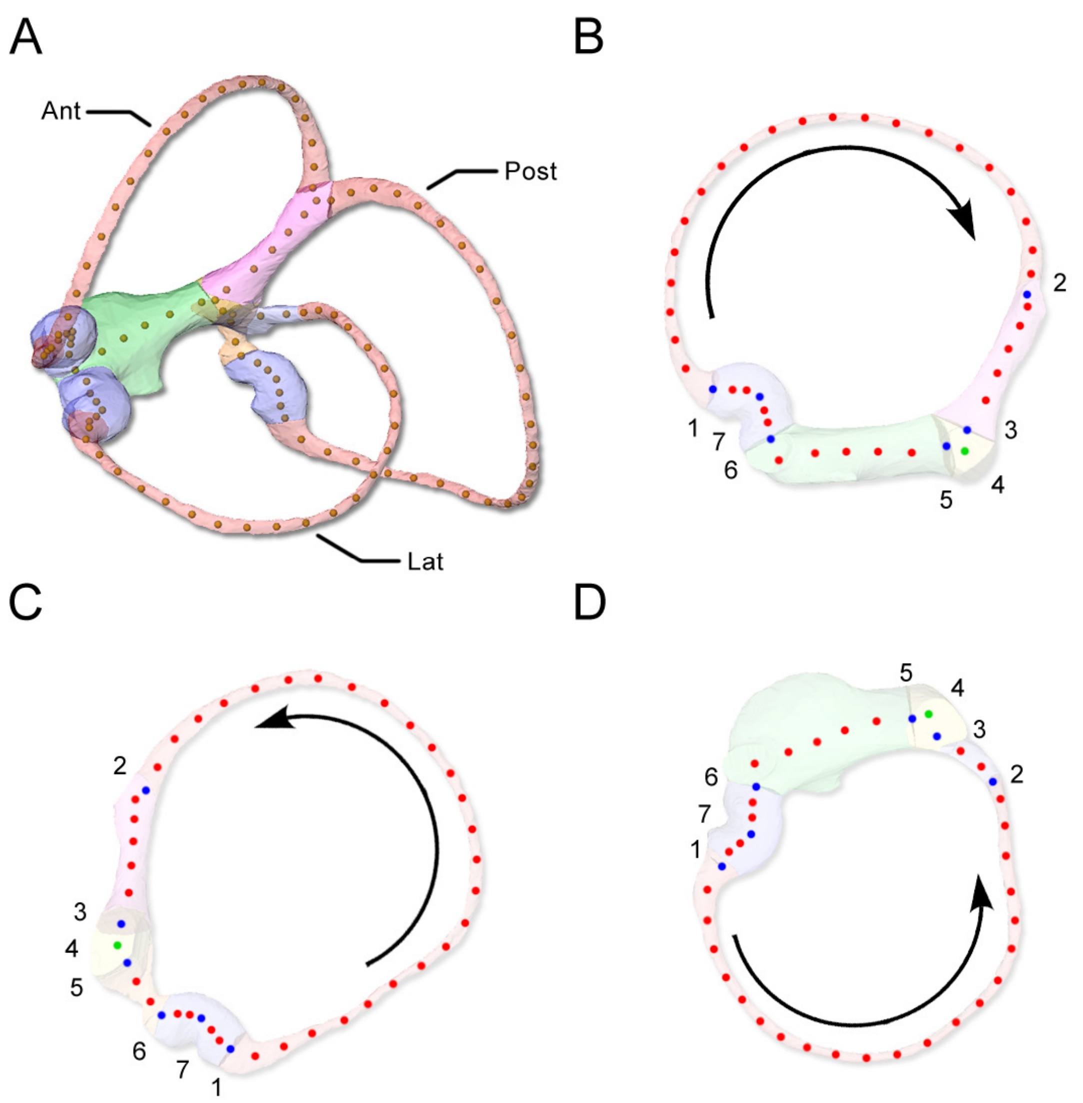

Figure 12. The left semicircular duct system of a human shown in isometric view (A), and planar views of the anterior (B), posterior (C) and lateral (D) semicircular ducts, rendered transparent to show the registration of the central streamlines using 3D landmarks. Colors codes as in Figure 4 and ducts identified as in Figure 3. The arrows indicate in which order the landmarks have to be placed. The numbers indicate type I landmarks shown in blue and green.

7.

  a. In [Project View], right-click the **rv_SPECIESNAME_Sa.stl** object and select [Display>Surface View].
  b. Do the same for all the other objects, except for the **SPECIESNAME_SCDS.Smt.SptGraph** object.
  c. Click the [Surface View] object.
  d. In the [Draw Style] scroll of the [Properties] panel, select [transparent].
  e. In the [Colors] scroll of the [Properties] panel, select [constant].
  f. In the [Colormap] section of the [Properties] panel, double click on the color box, pick a color and click [Apply].

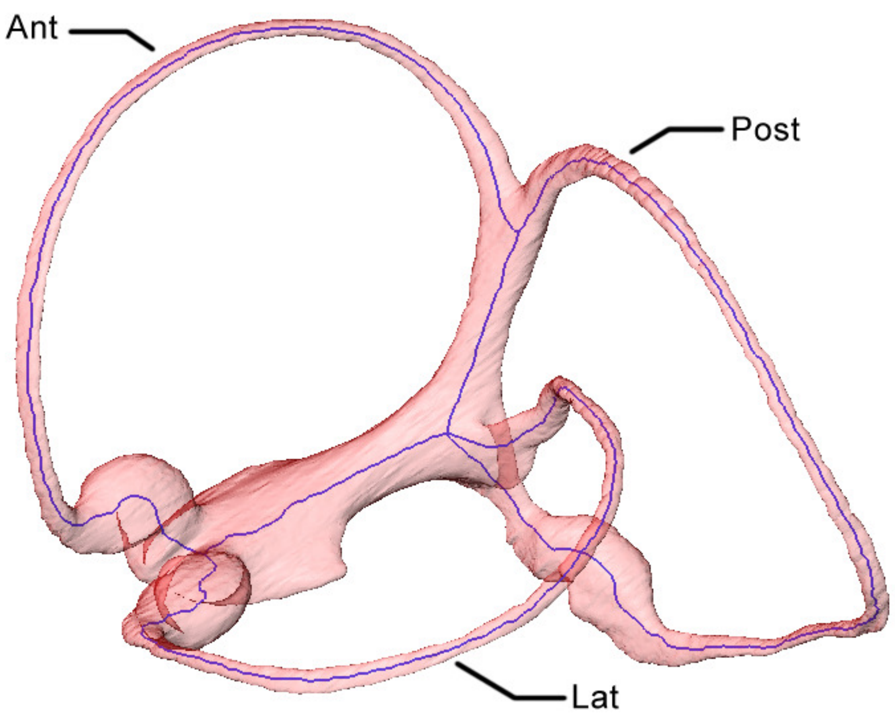

Figure 13. 3D model of the left semicircular duct system of a human, highlighting the skeleton (in blue) computed in Avizo 7.1. Ducts identified as in Figure 3.

g. Do this for the objects [Surface View 2] to [Surface View 11] by picking different colors to make the visualization of the 11 semicircular duct parts easier.

8.

a. Right-click on [Project View] and select [Create>Data>Landmarks].
b. Do it 11 times.
c. Click the [Landmarks] object and press the [F2] key.
d. In the textbox, enter **rcs_SPECIESNAME_Sa** ('**lcs_**' instead of **'rcs_'** for a left labyrinth) and click [OK].
e. Name the other [Landmarks] objects **rcs_SPECIESNAME_Sp**, **rcs_SPECIESNAME_Sl**, **rcs_SPECIESNAME_Aa**, **rcs_SPECIESNAME_Ap**, **rcs_SPECIESNAME_Al**, **rcs_ SPECIESNAME_CC**, **rcs_SPECIESNAME_Ua**, **rcs_SPECIESNAME_Up**, **rcs_ SPECIESNAME_SC** and **rcs_SPECIESNAME_Uc**.
f. Right-click the **rcs_SPECIESNAME_Sa** object and select [Display>Landmark View].
g. Do the same for all the other [Landmarks] objects.

9.

a. In [Project View], click the small orange box of all [Surface View] objects except for the **rv_SPECIESNAME_Sa.stl** object (the small boxes should become grey). This will hide all the meshes except for the **rv_SPECIESNAME_Sa.stl** mesh.
b. Click the small orange box of all [Landmark View] objects except for the **rcs_SPECIESNAME_Sa** object. This will hide all the landmarks except for the **rcs_SPECIESNAME_Sa** landmarks.

10.

a. In [Project View], click the **rcs_SPECIESNAME_Sa** object.

b. In the [Properties] panel, click the [Landmark Editor] button.
c. In the [Edit Mode] scroll, select [Add].
d. In the [Visualization] space, use the [Hand] to rotate the object and the [Arrow] to place landmarks. When interacting with the [Visualization] space, simply press the [Esc] key to switch between the [Hand] and the [Arrow].
e. With the [Arrow], place the first landmark at the intersection between the skeleton and the ampullary face of the slender part of the anterior semicircular duct (Fig. 14).

11.

In [Project View], hide the **rv_SPECIESNAME_Sa.stl** object by clicking on the small orange box of the [Surface View] that is attached to it.

12.

In the [Visualization] space, place ~25 more landmarks on the skeleton of the slender part of the anterior semicircular duct, starting at the ampulla and ending at the common crus. Stop shortly before the intersection between the skeleton and the common crus face of the slender part of the anterior semicircular duct (Fig. 14). As a general rule, the number of landmarks can vary and they do not need to be equally spaced. However, landmark order should be absolutely kept untouched (i.e. never place a new landmark in-between two previously placed landmarks).

13.

If landmarks need to be moved alongside the skeleton:
a. Click the landmark set object in [Project View].
b. In the [Properties] panel, click the [Landmark Editor] button.
c. In the [Edit Mode] scroll, select [Move].
d. In the [Visualization] space, with the [Arrow], click on the landmark that need to be moved, then click on its new position on the skeleton.

14.

For freely moving landmarks in space to make them better fit the central streamline trajectory:
a. In the [Visualization] space, orient the object in a way that facilitates landmark repositioning along the viewing plane.
b. In [Project View], click the landmark set object.
c. In the [Properties] panel, click the [Landmark Editor] button.
d. In the [Edit Mode] scroll, select [Transform].
e. In the [Visualization] space, with the [Arrow], click on the landmarks to move. Click on the green dragger pointing outside of the screen and drag it to place it in between the four other green draggers (Fig.15). That way, the sides of the bounding box should overlap from the viewing point and a square should be visible instead of a cube.
f. Click on the bounding box and drag the landmark where it has to be, along the viewing plane.
g. Explore the perpendicular views from the current viewing plane to precisely place the landmark in 3D space.

15.

In [Project View], show the **rv_SPECIESNAME_Sa.stl** object by clicking on the small grey box of the [Surface View] that is attached to it. The small box should become orange.

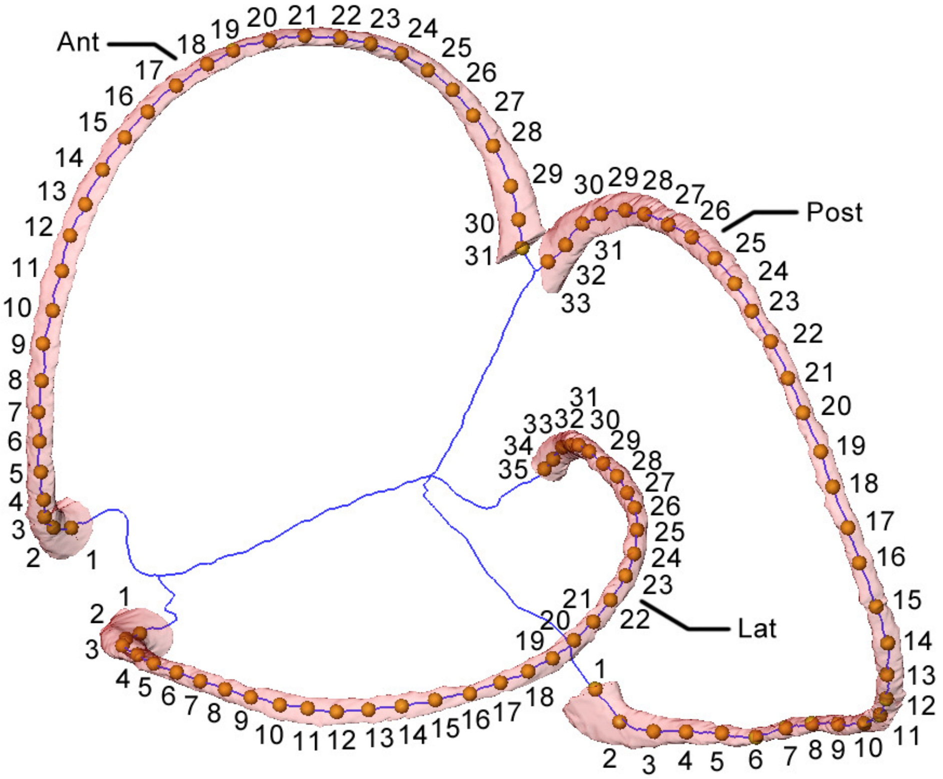

Figure 14. The template for landmark registration of the central streamlines of the slender semicircular duct parts, shown by the landmark sets lcs_SPECIESNAME_Sa, lcs_SPECIESNAME_Sp and lcs_SPECIESNAME_Sl. Ducts identified as in Figure 3.

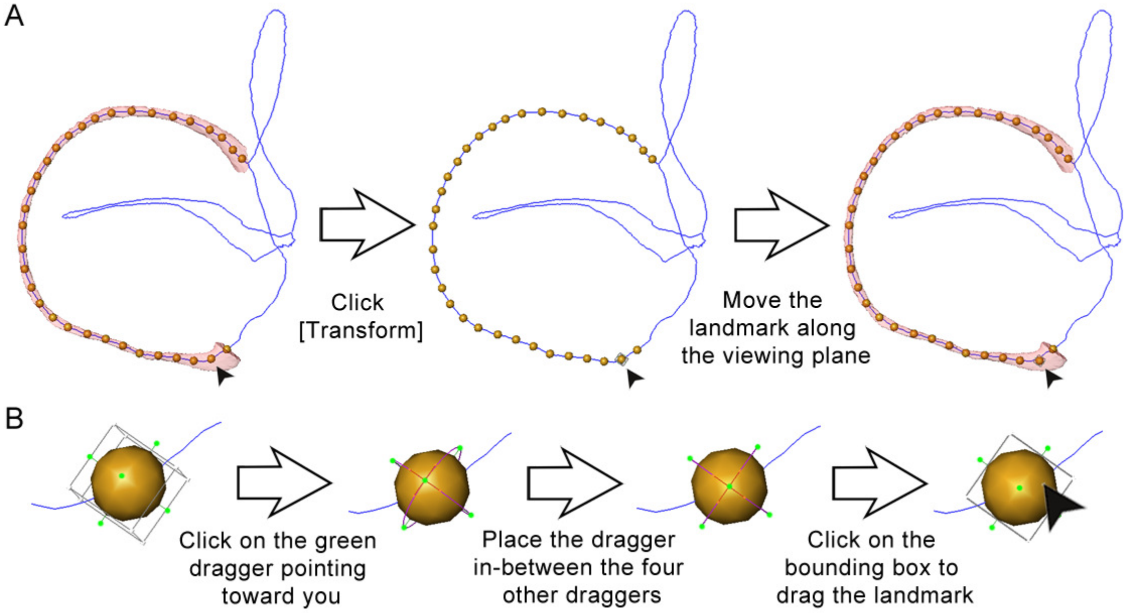

Figure 15. A. The procedure to move a 3D landmark anywhere in space along the viewing plane in Avizo 7.1. B. Close-up of a landmark, showing how its bounding box should be repositioned. The landmark (arrow) was moved from its initial location on the skeleton (blue line) to the middle of the semicircular duct lumen, better matching the location of the central streamline.

16.

In the [Visualization] space, with the [Arrow], place the last landmark at the intersection between the skeleton and the common crus face of the slender part of the anterior semicircular duct (Fig. 14).

17.

a. In [Project View], hide the **rv_SPECIESNAME_Sa.stl** object by clicking on the small orange box of the [Surface View] that is attached to it.
b. Show the **rv_SPECIESNAME_Sp.stl** object by clicking on the small grey box of the [Surface View] that is attached to it.
c. Hide the **rcs_SPECIESNAME_Sa** landmarks by clicking on the small orange box of the [Landmark View] that is attached to them
d. Show the **rcs_SPECIESNAME_Sp** landmarks by clicking on the small grey box of the [Landmark View] that is attached to them.

18.

a. In [Project View], click the **rcs_SPECIESNAME_Sp** object.
b. In the [Properties] panel, click the [Landmark Editor] button.
c. In the [Edit Mode] scroll, select [Add].
d. In the [Visualization] space, with the [Arrow], place the first landmark at the intersection between the skeleton and the ampullary face of the slender part of the posterior semicircular duct (Fig. 14).

19.

In [Project View], hide the **rv_SPECIESNAME_Sp.stl** object by clicking on the small orange box of the [Surface View] that is attached to it.

20.

In the [Visualization] space, place ~25 more landmarks on the skeleton of the slender part of the posterior semicircular duct, starting at the ampulla and ending at the common crus. Stop shortly before the intersection between the skeleton and the common crus face of the slender part of the posterior semicircular duct (Fig. 14).

21.

In [Project View], show the **rv_SPECIESNAME_Sp.stl** object by clicking on the small grey box of the [Surface View] that is attached to it.

22.

In the [Visualization] space, with the [Arrow], place the last landmark at the intersection between the skeleton and the common crus face of the slender part of the posterior semicircular duct (Fig. 14).

23.

a. In [Project View], hide the **rv_SPECIESNAME_Sp.stl** object by clicking on the small orange box of the [Surface View] that is attached to it.
b. Show the **rv_SPECIESNAME_Sl.stl** object by clicking on the small grey box of the [Surface View] that is attached to it.
c. Hide the **rcs_SPECIESNAME_Sp** landmarks by clicking on the small orange box of the [Landmark View] that is attached to them.
d. Show the **rcs_SPECIESNAME_Sl** landmarks by clicking on the small grey box of the [Landmark View] that is attached to them.

24.

a. In [Project View], click the **rcs_SPECIESNAME_Sl** object.
b. In the [Properties] panel, click the [Landmark Editor] button.
c. In the [Edit Mode] scroll, select [Add].
d. In the [Visualization] space, with the [Arrow], place the first landmark at the intersection between the skeleton and the ampullary face of the slender part of the lateral semicircular duct (Fig. 14).

25.

In [Project View], hide the **rv_SPECIESNAME_Sl.stl** object by clicking on the small orange box of the [Surface View] that is attached to it.

26.

In the [Visualization] space, place ~25 more landmarks on the skeleton of the slender part of the lateral semicircular duct, going from the ampulla to the simple crus. Stop slightly before the intersection between the skeleton and the simple crus face of the slender part of the lateral semicircular duct (Fig. 14).

27.

In [Project View], show the **rv_SPECIESNAME_Sl.stl** object by clicking on the small grey box of the [Surface View] that is attached to it.

28.

In the [Visualization] space, with the [Arrow], place the last landmark at the intersection between the skeleton and the simple crus face of the slender part of the lateral semicircular duct (Fig. 14).

29.

a. In [Project View], hide the **rv_SPECIESNAME_Sl.stl** object by clicking on the small orange box of the [Surface View] that is attached to it.
b. Hide the **rcs_SPECIESNAME_Sl** landmarks by clicking on the small orange box of the [Landmark View] that is attached to them.
c. Show the **rcs_SPECIESNAME_CC** landmarks by clicking on the small grey box of the [Landmark View] that is attached to them.

30.

a. In [Project View], click the **rcs_SPECIESNAME_CC** object.
b. In the [Properties] panel, click the [Landmark Editor] button.
c. In the [Edit Mode] scroll, select [Add].
d. In the [Visualization] space, with the [Arrow], place the first landmark at the junction of the trajectories of the central streamlines of the anterior and posterior slender duct and of the common crus (Fig.16). This location often corresponds to a trifurcation point of the skeleton but this is not a rule.
e. In the [Visualization] space, place ~5 more landmarks on the skeleton of the common crus, starting from the top and going to the common utricle. Stop shortly before the intersection between the skeleton and the common utricular face of the common crus (Fig. 16).

31.

In [Project View], show the **rv_SPECIESNAME_CC.stl** object by clicking on the small grey box of the [Surface View] that is attached to it.

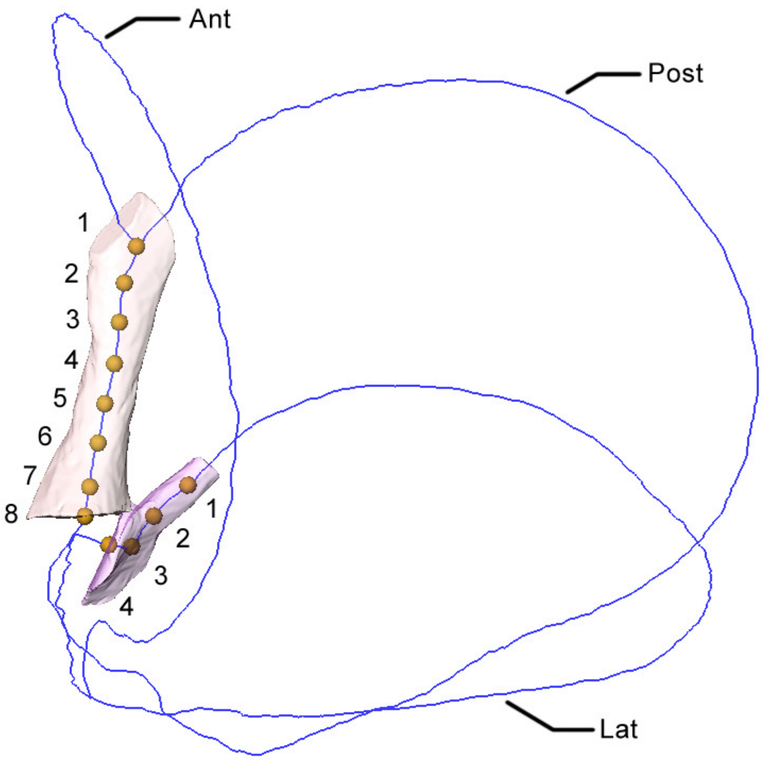

Figure 16. The template for landmark registration of the central streamlines of both the common crus and the simple crus, shown by the landmark sets lcs_SPECIESNAME_CC and lcs_SPECIESNAME_SC. Ducts identified as in Figure 3.

32.

In the [Visualization] space, with the [Arrow], place the last landmark at the intersection between the skeleton and the common utricular face of the common crus (Fig. 16).

33.

a. In [Project View], hide the **rv_SPECIESNAME_CC.stl** object by clicking on the small orange box of the [Surface View] that is attached to it.
b. Hide the **rcs_SPECIESNAME_CC** landmarks by clicking on the small orange box of the [Landmark View] that is attached to them.
c. Show the **rcs_SPECIESNAME_SC** landmarks by clicking on the small grey box of the [Landmark View] that is attached to them.

34.

a. In [Project View], click the **rcs_SPECIESNAME_SC** object.
b. In the [Properties] panel, click the [Landmark Editor] button.
c. In the [Edit Mode] scroll, select [Add].
d. In the [Visualization] space, with the [Arrow], place the first landmark slightly after the intersection between the skeleton and the lateral slender duct face of the simple crus (Fig.16).
e. Place ~3 more landmarks on the skeleton of the simple crus, starting from the lateral slender duct face and going to the common utricle. Stop shortly before the

intersection between the skeleton and the common utricular face of the simple crus (Fig. 16).

35.

In [Project View], show the **rv_SPECIESNAME_SC.stl** object by clicking on the small grey box of the [Surface View] that is attached to it.

36.

In the [Visualization] space, with the [Arrow], place the last landmark at the intersection between the skeleton and the common utricular face of the simple crus (Fig. 16).

37.

a. In [Project View], hide the **rv_SPECIESNAME_SC.stl** object by clicking on the small orange box of the [Surface View] that is attached to it.
b. Hide the **rcs_SPECIESNAME_SC** landmarks by clicking on the small orange box of the [Landmark View] that is attached to them.
c. Show the **rcs_SPECIESNAME_Uc** landmarks by clicking on the small grey box of the [Landmark View] that is attached to them.

38.

a. In [Project View], click the **rcs_SPECIESNAME_Uc** object.
b. In the [Properties] panel, click the [Landmark Editor] button.
c. In the [Edit Mode] scroll, select [Add].
d. In the [Visualization] space, with the [Arrow], place a single landmark at the junction of the trajectories of the central streamlines of the anterior and posterior utricles, of the common crus and of the simple crus (Fig.17). This location often corresponds to a multifurcation point of the skeleton but this is not a rule. If the landmark is difficult to place, increase its size until it fills the mesh model of the common utricle. This will point out its actual location. To do so:
    i. In [Project View], click the [Landmark View] object that is attached to the **rcs_SPECIESNAME_Uc** object.
    ii. In the [Properties] panel, click the [Increase] button and play with the [Size] dragger until the landmark completely fills the common utricle.

39.

a. In [Project View], show the **rv_SPECIESNAME_Ua.stl** object by clicking on the small grey box of the [Surface View] that is attached to it.
b. Hide the **rcs_SPECIESNAME_Uc** landmarks by clicking on the small orange box of the [Landmark View] that is attached to them.
c. Show the **rcs_SPECIESNAME_Ua** landmarks by clicking on the small grey box of the [Landmark View] that is attached to them.

40.

a. In [Project View], click the **rcs_SPECIESNAME_Ua** object.
b. In the [Properties] panel, click the [Landmark Editor] button.
c. In the [Edit Mode] scroll, select [Add].
d. In the [Visualization] space, with the [Arrow], place the first landmark at the intersection between the skeleton and the common utricular face of the anterior utricle (Fig. 18).

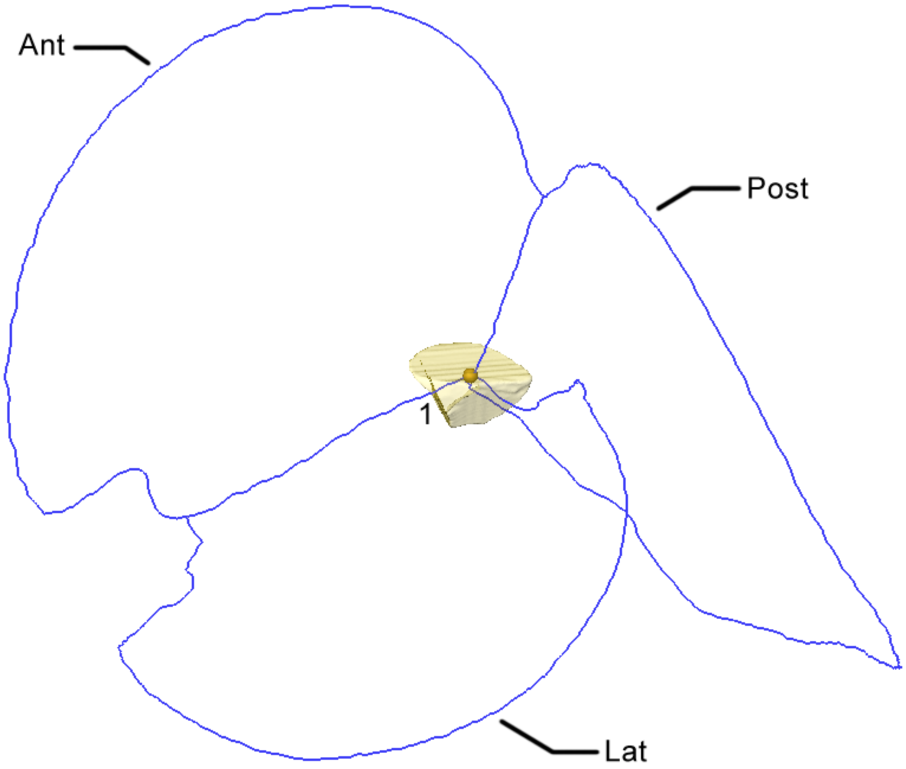

Figure 17. The template for landmark registration of the streamline of the common utricle as shown by landmark set lcs_SPECIESNAME_Uc. Ducts identified as in Figure 3.

41. In [Project View], hide the **rv_SPECIESNAME_Ua.stl** object by clicking on the small orange box of the [Surface View] that is attached to it.

42. In the [Visualization] space, place ~5 more landmarks on the skeleton of the anterior utricle, starting at the common utricle and ending at the anterior ampulla. Stop at the junction of the trajectories of the central streamlines of the anterior and lateral ampullae and of the anterior utricle (Fig.18). This location often corresponds to a trifurcation point of the skeleton but this is not a rule.

43.
    a. In [Project View], show the **rv_SPECIESNAME_Up.stl** object by clicking on the small grey box of the [Surface View] that is attached to it.
    b. Hide the **rcs_SPECIESNAME_Ua** landmarks by clicking on the small orange box of the [Landmark View] that is attached to them.
    c. Show the **rcs_SPECIESNAME_Up** landmarks by clicking on the small grey box of the [Landmark View] that is attached to them.

44.
    a. In [Project View], click the **rcs_SPECIESNAME_Up** object.
    b. In the [Properties] panel, click the [Landmark Editor] button.
    c. In the [Edit Mode] scroll, select [Add].

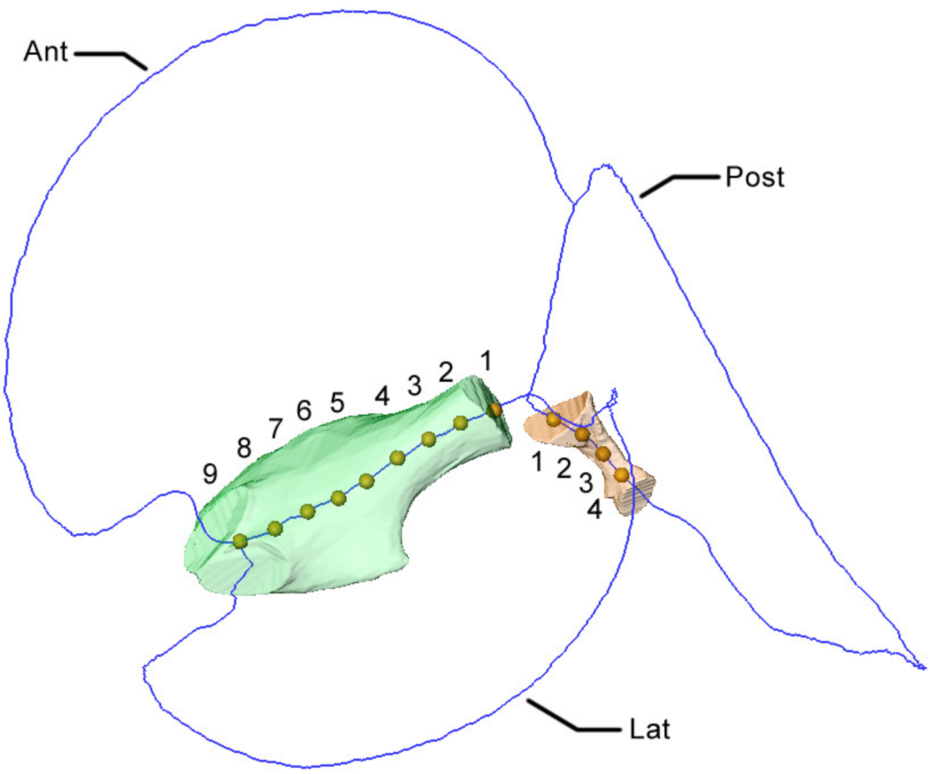

Figure 18. The template for landmark registration of the central streamlines of both the anterior and posterior utricles, shown by landmark sets lcs_SPECIESNAME_Ua and lcs_SPECIESNAME_Up. Ducts identified as in Figure 3.

d. In the [Visualization] space, with the [Arrow], place the first landmark at the intersection between the skeleton and the common utricular face of the posterior utricle (Fig. 18).

45.

In [Project View], hide the **rv_SPECIESNAME_Up.stl** object by clicking on the small orange box of the [Surface View] that is attached to it.

46.

In the [Visualization] space, place ~3 more landmarks on the skeleton of the posterior utricle, starting at the common utricle and ending at the posterior ampulla. Stop shortly before the intersection between the skeleton and the ampullary face of the posterior utricle (Fig.18).

47.

a. In [Project View], show the **rv_SPECIESNAME_Aa.stl** object by clicking on the small grey box of the [Surface View] that is attached to it.
b. Hide the **rcs_SPECIESNAME_Up** landmarks by clicking on the small orange box of the [Landmark View] that is attached to them.
c. Show the **rcs_SPECIESNAME_Aa** landmarks by clicking on the small grey box of the [Landmark View] that is attached to them.

48.

a. In [Project View], click the **rcs_SPECIESNAME_Aa** object.

b. In the [Properties] panel, click the [Landmark Editor] button.
c. In the [Edit Mode] scroll, select [Add].
d. In the [Visualization] space, with the [Arrow], place the first landmark at the intersection between the skeleton and the anterior utricular face of the anterior ampulla (Fig. 19).

49.

In [Project View], hide the **rv_SPECIESNAME_Aa.stl** object by clicking on the small orange box of the [Surface View] that is attached to it.

50.

In the [Visualization] space, place ~5 more landmarks on the skeleton of the anterior ampulla, going from the anterior utricle to the anterior slender duct. Stop slightly before the intersection between the skeleton and the slender duct face of the anterior ampulla (Fig.19). The fourth landmark should absolutely be placed above the *crista ampullaris* of the anterior ampulla if these landmarks sets are also planned to be used for geometric morphometrics analyses.

51.

a. In [Project View], show the **rv_SPECIESNAME_Ap.stl** object by clicking on the small grey box of the [Surface View] that is attached to it.
b. Hide the **rcs_SPECIESNAME_Aa** landmarks by clicking on the small orange box of the [Landmark View] that is attached to them.
c. Show the **rcs_SPECIESNAME_Ap** landmarks by clicking on the small grey box of the [Landmark View] that is attached to them.

52.

a. In [Project View], click the **rcs_SPECIESNAME_Ap** object.
b. In the [Properties] panel, click the [Landmark Editor] button.
c. In the [Edit Mode] scroll, select [Add].
d. In the [Visualization] space, with the [Arrow], place the first landmark at the intersection between the skeleton and the posterior utricular face of the posterior ampulla (Fig. 19).

53.

In [Project View], hide the **rv_SPECIESNAME_Ap.stl** object by clicking on the small orange box of the [Surface View] that is attached to it.

54.

In the [Visualization] space, place ~5 more landmarks on the skeleton of the posterior ampulla, going from the posterior utricle to the posterior slender duct. Stop slightly before the intersection between the skeleton and the slender duct face of the posterior ampulla (Fig.19). The fourth landmark should absolutely be placed above the *crista ampullaris* of the posterior ampulla if these landmarks sets are also planned to be used for geometric morphometrics analyses.

55.

a. In [Project View], show the **rv_SPECIESNAME_Al.stl** object by clicking on the small grey box of the [Surface View] that is attached to it.
b. Hide the **rcs_SPECIESNAME_Ap** landmarks by clicking on the small orange box of the [Landmark View] that is attached to them.

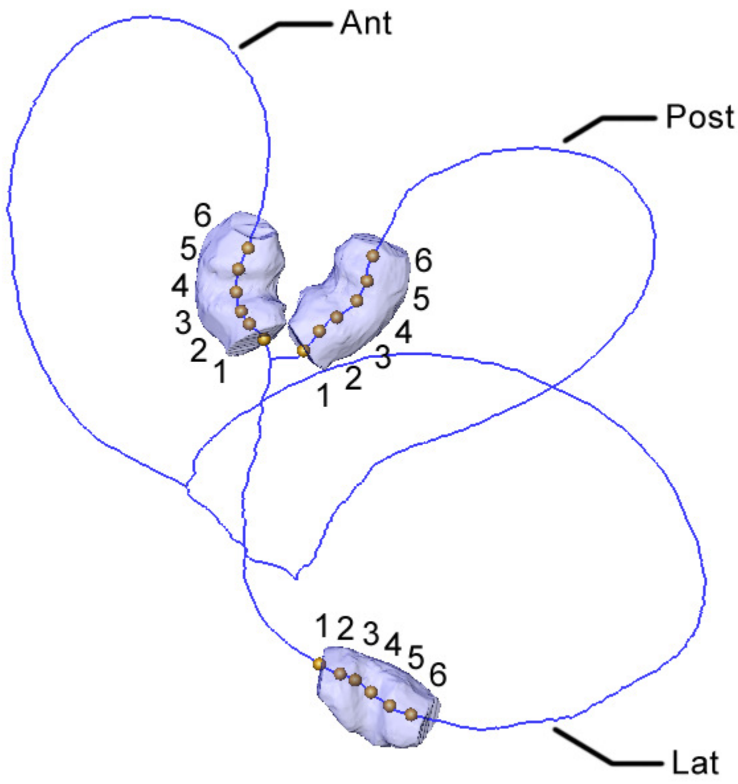

Figure 19. The template for landmark registration of the central streamlines of the ampullary parts, as shown by landmark sets lcs_SPECIESNAME_Aa, lcs_SPECIESNAME_Ap and lcs_SPECIESNAME_Al. Ducts identified as in Figure 3.

c. Show the **rcs_SPECIESNAME_Al** landmarks by clicking on the small grey box of the [Landmark View] that is attached to them.

56.

a. In [Project View], click the **rcs_SPECIESNAME_Al** object.
b. In the [Properties] panel, click the [Landmark Editor] button.
c. In the [Edit Mode] scroll, select [Add].
d. In the [Visualization] space, with the [Arrow], place the first landmark at the intersection between the skeleton and the anterior utricular face of the lateral ampulla (Fig. 19).

57.

In [Project View], hide the **rv_SPECIESNAME_Al.stl** object by clicking on the small orange box of the [Surface View] that is attached to it.

58.

In the [Visualization] space, place ~5 more landmarks on the skeleton of the lateral ampulla, going from the anterior utricle to the lateral slender duct. Stop slightly before the intersection between the skeleton and the slender duct face of the lateral ampulla (Fig.19). The fourth landmark should absolutely be placed above the *crista ampullaris* of the lateral ampulla if these landmarks sets are also planned to be used for geometric morphometrics analyses.

59.

a. Save the **rcs_SPECIESNAME_Sa** object as a LandmarkSet ascii file, in folder **/AriadneToolbox/Data/SPECIESNAME/ScanRef/Landmarks/**.
b. Do the same for the objects **rcs_SPECIESNAME_Sp**, **rcs_SPECIESNAME_Sl**, **rcs_SPECIESNAME_Aa**, **rcs_SPECIESNAME_Ap**, **rcs_SPECIESNAME_Al**, **rcs_ SPECIESNAME_CC**, **rcs_SPECIESNAME_Ua**, **rcs_SPECIESNAME_Up**, **rcs_ SPECIESNAME_SC**, and **rcs_SPECIESNAME_Uc**.

Tip: After loading an Avizo project that contains previously created [Landmarks] objects, the [Transform] option can occasionally not be used to move individual landmarks. A workaround is to delete the problematic [Landmarks] objects from [Project View] and to reload them using [Open Data…]. The [Transform] option should then work again.

The full procedure provides the user with 11 landmark sets corresponding to the main divisions of the central streamlines of the semicircular duct system. An overview of these 11 landmark sets is provided in Figure 20.

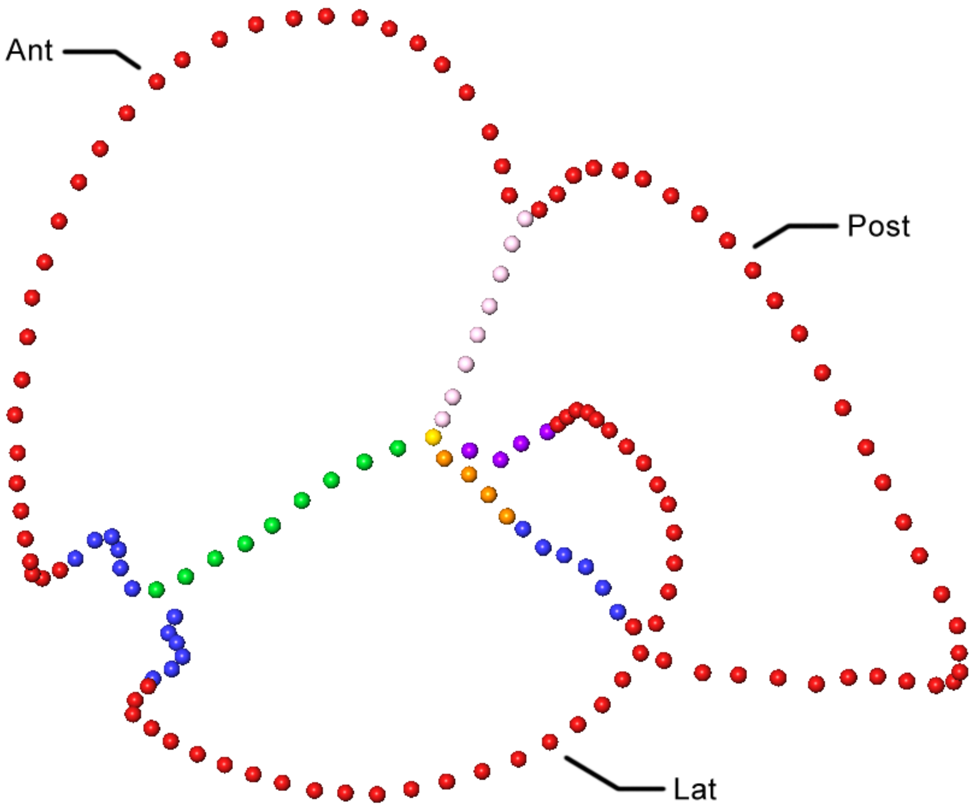

Figure 20. Overview of all landmark sets registering the central streamlines of the left semicircular duct system of a human. Landmark colors: slender ducts color in red, ampullae color in blue, common crus color in pink, simple crus color in purple, anterior utricle color in green, posterior utricle color in orange and the common utricle color in yellow. Ducts identified as in Figure 3.

iv. Building three-dimensional models of the cupulae

The fourth step of data preparation consists of building a 3D model for the cupula of each semicircular duct from which volume and surface STL files can be extracted. The cupula is a viscoelastic structure that completely fills the ampulla above the *crista ampullaris* and deflects when differential pressure, generated by endolymph motion, is exerted on its faces towards the utricle and slender duct. The cupula is mechanically coupled to sensory hair cells at the surface of the *crista ampullaris* and strongly affects both the sensitivity and the long time constant of each semicircular duct[2-5]. The cupula has historically been a difficult structure to visualize, in 3D in particular. In previous biomechanical models the cross-section of the ampulla above the crista *ampullaris* was therefore used as a proxy for its dimensions. Under this assumption, cupula cross-section shape was considered as being circular or elliptical and cupula thickness was generally calculated based on an empirical law elaborated on the toad fish (*Opsanus tau*)[2-4, 6].

Using X-ray micro tomography after staining the inner ear the cupula can be completely visualized in-situ[1], in some cases without shrinkage (Fig. 21). Based on the information thus acquired the Ariadne Toolbox takes the full 3D morphology of the cupula into account in the biomechanical analyses. This approach is based on the observation that cupula shape corresponds to an extrusion of the shape of the *crista ampullaris* towards the ampulla roof. This finding is consistent with the idea that cupula components are secreted by support cells surrounding the hair cells covering the *crista ampullaris*[7]. While being shaped similarly to the *crista ampullaris* in transverse cross-section, the cupula is actually slightly thicker because it does not directly rise from the surface of the *crista ampullaris* but rather from the top of the so called “subcupular space”, the thickness of which equals the length of the longest stereocilia[7] (Fig.22). When the cupula itself is not visible, it is thus possible to build a representative model by extruding the shape of the *crista ampullaris* toward the roof of the ampulla. The thickness of cupula models obtained that way are later adjusted using Ariadne (see section I.iii.6. of the Ariadne Manual – Modules description) by assuming a range of plausible stereocilia lengths.

Building the cupula models provides us with 3 volume and 12 surface STL files. These files will be used to compute the mean cross-sectional areas and the mean thicknesses of the three cupulae (see section I.iii.6. of the Ariadne Manual – Modules description). They will also be used to analyze cupula deflection using the finite element method (see section I.v. of this manual and sections I.ii. and I.v. of the Ariadne Manual – Modules description). Here we describe how to build the 3D cupula models, and extract the volume and surface STL files using Geomagic Studio 12 and Avizo 7.1.:

1.
    a. In Geomagic Studio 12, click [Geomagic>Import], select the file **/AriadneToolbox/Data/SPECIESNAME/ScanRef/Surfaces/rs_SPECIESNAME_Aa.stl** and click [Open].
    b. Specify the units that were used during the segmentation process and click [OK].
    c. Decline using the [Mesh Doctor].
    d. Do the same for the files **rs_SPECIESNAME_Ap.stl** and **rs_SPECIESNAME_Al.stl**.

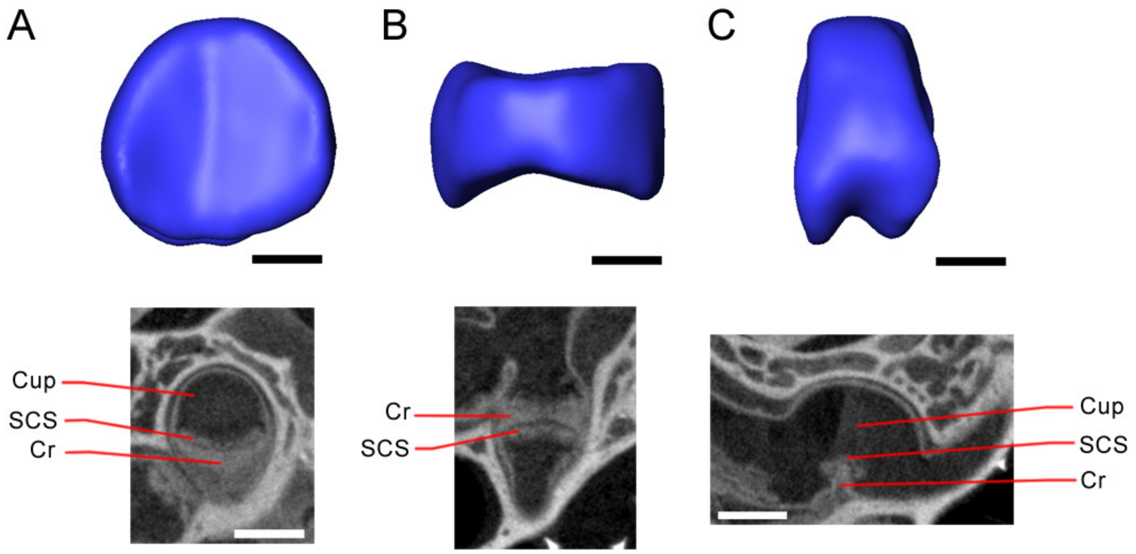

Figure 21. CT images of the anterior cupula of *Galago senegalensis* showing its actual shape *in situ*, as well as 3D reconstructions (blue) seen in the same orientation: (A) from the duct lumen, (B) from the ampullary roof and (C) from the side of the ampulla. Black scale bar: 200µm. White scale bar: 500µm. Cup: cupula, SCS: subcupular space, Cr: *crista ampullaris*.

2.
   a. In the [Model Manager], right-click the **rs_SPECIESNAME_Aa** model.
   b. Click [Polygons>Repair>Remesh].
   c. In the [Target Edge Length] textbox enter '5' µm.
   d. Select [Use Existing Boundaries Only] and click [Apply], then [OK].
   e. If Geomagic 12 prevents from using '5' µm as the [Target Edge Length], click [Polygons>Repair>Remesh>Refine], select [4X Subdivision], click [Apply], then [OK] and retry the [Remesh] operation.
   f. Do the same for the files **rs_SPECIESNAME_Ap.stl** and **rs_SPECIESNAME_Al.stl**.
3.
   a. Save the **rs_SPECIESNAME_Aa** model as a STL ascii file, named as **rs_SPECIESNAME_Ant_Cup_Wall.stl**, in folder **/AriadneToolbox/Data/SPECIESNAME/ScanRef/Surfaces/**.
   b. Do the same for the models **rs_SPECIESNAME_Ap** and **rs_SPECIESNAME_Al** by replacing the '**_Ant_**' suffix in the filename by their own respective suffixes **'_Post_'** and **'_Lat_'**.

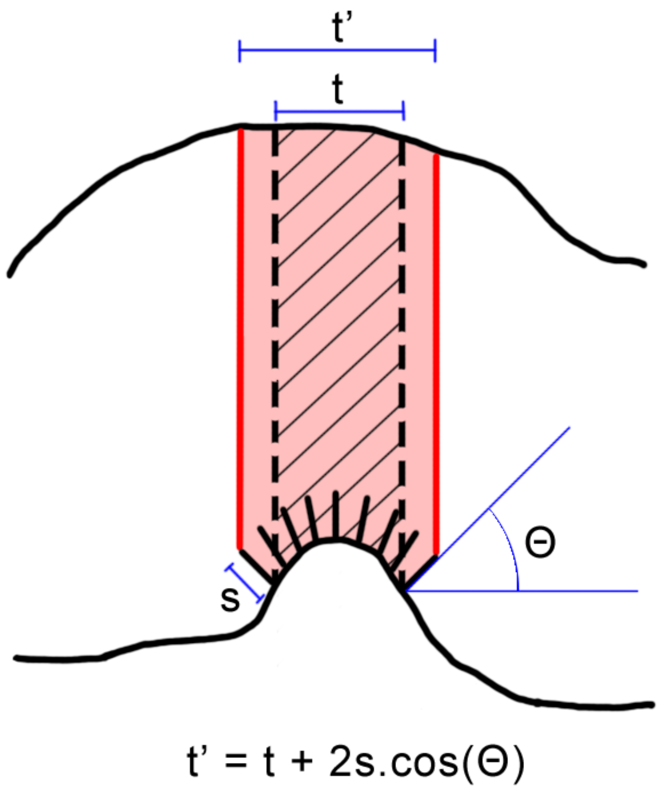

Figure 22. Sketch showing how the thickness t' of the cupula (in red) can be linked to the thickness t of the upwards projected *crista ampullaris* (dashed lines) using the length s of the peripheral stereocilia and their angle Θ with the base of the *crista ampullaris*.

4.

   a. Save the **rs_SPECIESNAME_Aa** model as a PLY file, named as **rs_SPECIESNAME_Ant_Cup_Wall_Temp.ply**, in folder **/AriadneToolbox/Data/SPECIESNAME/ScanRef/Surfaces/**.
   b. Do the same for the models **rs_SPECIESNAME_Ap** and **rs_SPECIESNAME_Al** by replacing the '**_Ant_**' suffix in the filename by their own respective suffixes **'_Post_'** and **'_Lat_'**.

5.

   a. In Avizo 7.1, click [Project View>Open Data].
   b. In the folder **/AriadneToolbox/Data/SPECIESNAME/ScanRef/Surfaces/**, select the files **rs_SPECIESNAME_Ant_Cup_Wall_Temp.ply**, **rs_SPECIESNAME_Post_Cup_Wall_Temp.ply** and **rs_SPECIESNAME_Lat_Cup_Wall_Temp.ply** and click [Open].

6.

a. In [Project View], right-click the **rs_SPECIESNAME_Ant_Cup_Wall_Temp.ply** object and select [Display>Surface View].
b. Do the same for all other objects.
c. Click the [Surface View] object.
d. In the [Colors] scroll of the [Properties] panel, select [constant].
e. In the [Colormap] section of the [Properties] panel, click on the color box, pick a dark color to make the object looks less shiny and click [Apply].
f. Do this for all the objects.

7.

a. Right-click on [Project View] and select [Create>Data>Landmarks].
b. Do it 3 times.
c. Click the [Landmarks] object and press the [F2] key.
d. In the textbox, enter **r_SPECIESNAME_Cra** and click [OK].
e. Name the other [Landmarks] objects **r_SPECIESNAME_Crp** and **r_SPECIESNAME_Crl**.
f. Right-click the **r_SPECIESNAME_Cra** object and select [Display>Landmark View].
g. Do the same for all the other [Landmarks] objects.

8.

a. In [Project View], click the small orange box of all [Surface View] objects except for the **rs_SPECIESNAME_Ant_Cup_Wall_Temp.ply** object to hide them.
b. Click the small orange box of all [Landmark View] objects except for the **r_SPECIESNAME_Cra** object to hide them.

9.

a. In [Project View], click the **r_SPECIESNAME_Cra** object.
b. In the [Properties] panel, click the [Landmark Editor] button.
c. In the [Edit Mode] scroll, select [Add].
d. In the [Visualization] space, with the [Arrow], place several landmarks on the surface of the ampulla at the level of the lateral margins of the *crista ampullaris* (Fig. 23). Landmark number and order are not important here.

10.

In [Project View], hide the **rs_SPECIESNAME_Ant_Cup_Wall_Temp.ply** object by clicking on the small orange box of the [Surface View] that is attached to it.

11.

In the [Visualization] space, with the [Hand], orient the landmark set **r_SPECIESNAME_Cra** such that the landmarks of the two margins best overlap. Sometimes, the landmarks won't overlap when the *crista ampullaris* is asymmetric. In this case, orient the landmark set such that the distance between the landmarks of the two margins looks homogeneous in the view plane (Fig. 23).

12.

In [Project View], show the **rs_SPECIESNAME_Ant_Cup_Wall_Temp.ply** object by clicking on the small grey box of the [Surface View] that is attached to it.

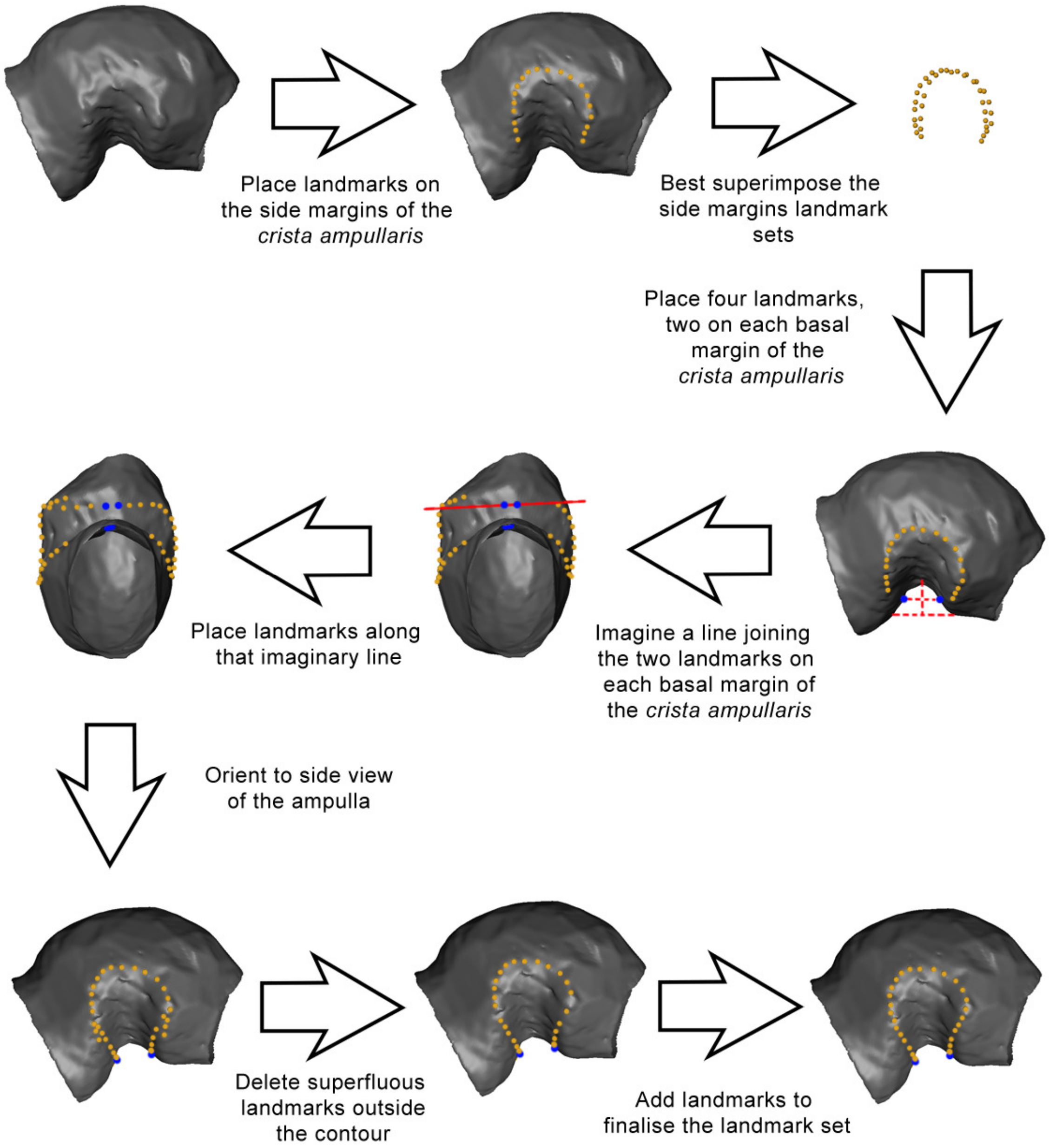

Figure 23. 3D model of the left anterior ampulla of a human, illustrating the procedure to landmark the shape of the *crista ampullaris* in order to build the cupula model.

13. In the [Visualization] space, with the [Arrow], place one landmark on each side of the *crista ampullaris*, equidistantly spaced from its roof and its base (Fig. 23, see dashed lines).

14. In the [Console], type 'viewer rotate 90' and press the [Enter] key. Do it twice. The [↑] key can be pressed the second time instead of retyping the whole command.

15.

In the [Visualization] space, with the [Arrow], place again one landmark on each side of the *crista ampullaris*, equidistantly spaced from its roof and its base (Fig. 23).

16.

a. In the [Visualization] space, with the [Hand], orient the ampulla in such a way that both landmarks that were previously placed on one side of the *crista ampullaris* become visible.
b. Draw a virtual line joining them and place landmarks along that line until reaching the landmarks that were placed on the margin of the *crista ampullaris* (Fig. 23).
c. Do the same for the other side of the *crista ampullaris*.

17.

a. In [Project View], click the **r_SPECIESNAME_Cra** object.
b. In the [Properties] panel, click the [Landmark Editor] button.
c. In the [Edit Mode] scroll, select [Remove].
d. In the [Visualization] space, with the [Arrow], remove the landmarks that cross the margin of the *crista ampullaris* (Fig. 23).

18.

a. In [Project View], click the **r_SPECIESNAME_Cra** object.
b. In the [Properties] panel, click the [Landmark Editor] button.
c. In the [Edit Mode] scroll, select [Add].
d. In the [Visualization] space, with the [Arrow], add some landmarks between the margins and the sides of the *crista ampullaris* if necessary (Fig. 23).

19.

Save the **r_SPECIESNAME_Cra** model as a LandmarkSet ascii file in folder **/AriadneToolbox/Data/SPECIESNAME/ScanRef/Landmarks/**.

20.

In the [Visualization] space, with the [Hand], orient the ampulla in such a way that the landmarks on only one side of the *crista ampullaris* can be seen (Fig. 24).

21.

a. In [Project View], click the **rs_SPECIESNAME_Ant_Cup_Wall_Temp.ply** object.
b. In the [Properties] panel, click the [Surface Editor] button.
c. In the [GUI control] of the [Surface Editor] that just appeared, click the button [Draw contour to highlight faces]. Do not select [Visible triangles only].
d. In the [Visualization] space, with the [Arrow], enclose all triangles that are ‘above’ the landmarks on the visible side of the *crista ampullaris* (Fig. 24).
e. Orient the ampulla in such a way that only the other side of the *crista ampullaris* can be seen.
f. Enclose again all triangles that are ‘above’ the landmarks on the visible side of the *crista ampullaris* (Fig.24).
g. In the topmost [GUI Ribbon], select [Surface>Edit>Delete highlighted faces] (Fig.24).
h. In the [Properties] panel, click the [Surface Editor] button.

22.

In [Project View], hide the **rs_SPECIESNAME_Ant_Cup_Wall_Temp.ply** object by clicking on the small orange box of the [Surface View] that is attached to it.

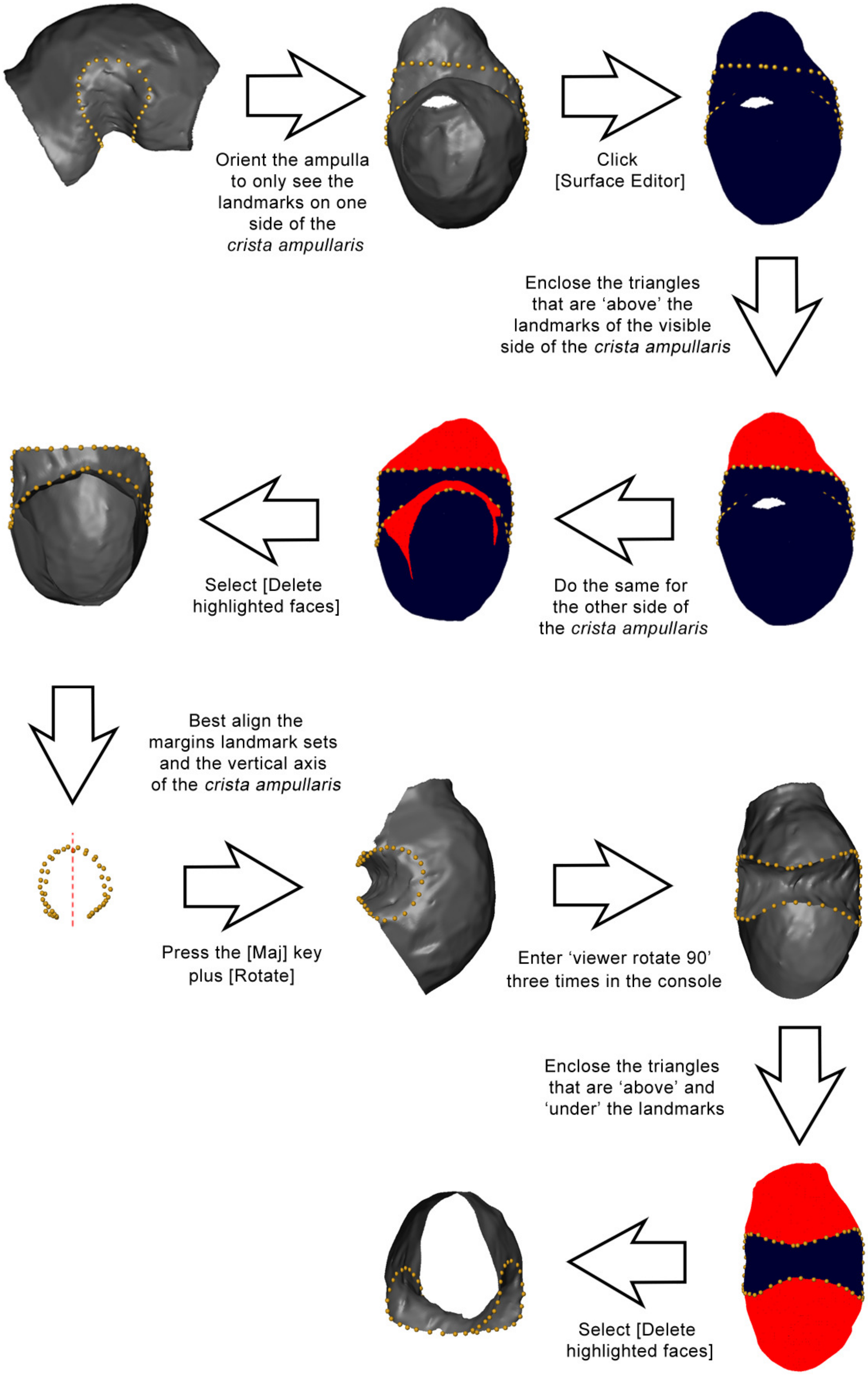

Figure 24. 3D model of the left anterior ampulla of a human, illustrating the procedure to project the shape of the *crista ampullaris* upwards in order to build the ampullary walls of the cupula model.

23.

In the [Visualization] space, with the [Hand], orient the landmark set **r_SPECIESNAME_Cra** such that the landmarks of the two margins best overlap.

24.

In [Project View], show the **rs_SPECIESNAME_Ant_Cup_Wall_Temp.ply** object by clicking on the small grey box of the [Surface View] that is attached to it.

25.

a. In the [Visualization GUI], press the [Rotate] button until the vertical axis of the *crista ampullaris* is aligned to the vertical axis of the screen (Fig. 24). The [Maj] key plus the [Rotate] button can be pressed to rotate the view 90° clockwise, whereas the [Ctrl] key plus the [Rotate] button can be pressed to rotate counter clockwise.
b. Press the [Maj] key plus the [Rotate] button (Fig.24).

26.

a. In the [Console], type 'viewer rotate 90' and press the [Enter] key.
b. Do it three times (Fig. 24).

27.

a. In [Project View], click the **rs_SPECIESNAME_Ant_Cup_Wall_Temp.ply** object.
b. In the [Properties] panel, click the [Surface Editor] button.
c. In the [GUI control] of the [Surface Editor], click the button [Draw contour to highlight faces].
d. In the [Visualization] space, with the [Arrow], enclose all triangles that are 'above' and 'under' the landmarks of the *crista ampullaris* (Fig. 24).
e. In the topmost [GUI Ribbon], select [Surface>Edit>Delete highlighted faces] (Fig.24).
f. In the [Properties] panel, click the [Surface Editor] button.

28.

Save the **rs_SPECIESNAME_Ant_Cup_Wall_Temp.ply** model as a STL ascii file, named as **rs_SPECIESNAME_Ant_Cup_Wall_Temp.stl**, in folder **/AriadneToolbox/Data/SPECIESNAME/ScanRef/Surfaces/**.

29.

a. In [Project View], hide the **rs_SPECIESNAME_Ant_Cup_Wall_Temp.ply** object by clicking on the small orange box of the [Surface View] that is attached to it.
b. Show the **rs_SPECIESNAME_Post_Cup_Wall_Temp.ply** object by clicking on the small grey box of the [Surface View] that is attached to it.
c. Hide the **r_SPECIESNAME_Cra** landmarks by clicking on the small orange box of the [Landmark View] that is attached to them.
d. Show the **r_SPECIESNAME_Crp** landmarks by clicking on the small grey box of the [Landmark View] that is attached to them.

30.

Reiterate the steps 9 to 18 by replacing the **rs_SPECIESNAME_Ant_Cup_Wall_Temp.ply** object by the **rs_SPECIESNAME_Post_Cup_Wall_Temp.ply** object and the **r_SPECIESNAME_Cra** landmarks by the **r_SPECIESNAME_Crp** landmarks.

31.

Save the **r_SPECIESNAME_Crp** model as a LandmarkSet ascii file in folder **/AriadneToolbox/Data/SPECIESNAME/ScanRef/Landmarks/**.

32. Reiterate the steps 20 to 27 by replacing the **rs_SPECIESNAME_Ant_Cup_Wall_Temp.ply** object by the **rs_SPECIESNAME_Post_Cup_Wall_Temp.ply** object and the **r_SPECIESNAME_Cra** landmarks by the **r_SPECIESNAME_Crp** landmarks.

33. Save the **rs_SPECIESNAME_Post_Cup_Wall_Temp.ply** model as a STL ascii file, named as **rs_SPECIESNAME_Post_Cup_Wall_Temp.stl**, in folder **/AriadneToolbox/Data/SPECIESNAME/ScanRef/Surfaces/**.

34.
    a. In [Project View], hide the **rs_SPECIESNAME_Post_Cup_Wall_Temp.ply** object by clicking on the small orange box of the [Surface View] that is attached to it.
    b. Show the **rs_SPECIESNAME_Lat_Cup_Wall_Temp.ply** object by clicking on the small grey box of the [Surface View] that is attached to it.
    c. Hide the **r_SPECIESNAME_Crp** landmarks by clicking on the small orange box of the [Landmark View] that is attached to them.
    d. Show the **r_SPECIESNAME_Crl** landmarks by clicking on the small grey box of the [Landmark View] that is attached to them.

35. Reiterate the steps 9 to 18 by replacing the **rs_SPECIESNAME_Ant_Cup_Wall_Temp.ply** object by the **rs_SPECIESNAME_Lat_Cup_Wall_Temp.ply** object and the **r_SPECIESNAME_Cra** landmarks by the **r_SPECIESNAME_Crl** landmarks.

36. Save the **r_SPECIESNAME_Crl** model as a LandmarkSet ascii file in folder **/AriadneToolbox/Data/SPECIESNAME/ScanRef/Landmarks/**.

37. Reiterate the steps 20 to 27 by replacing the **rs_SPECIESNAME_Ant_Cup_Wall_Temp.ply** object by the **rs_SPECIESNAME_Lat_Cup_Wall_Temp.ply** object and the **r_SPECIESNAME_Cra** landmarks by the **r_SPECIESNAME_Crl** landmarks.

38. Save the **rs_SPECIESNAME_Lat_Cup_Wall_Temp.ply** model as a STL ascii file, named as **rs_SPECIESNAME_Lat_Cup_Wall_Temp.stl**, in folder **/AriadneToolbox/Data/SPECIESNAME/ScanRef/Surfaces/**.

39.
    a. <u>In Geomagic Studio 12</u>, click [Geomagic>Import].
    b. Select the file **/AriadneToolbox/Data/SPECIESNAME/ScanRef/Surfaces/rs_SPECIESNAME_Ant_Cup_Wall.stl** and click [Open].
    c. Specify the units that were used during the segmentation process and click [OK].
    d. Decline using the [Mesh Doctor].
    e. Do the same for the files **rs_SPECIESNAME_Post_Cup_Wall.stl**, **rs_SPECIESNAME_Lat_Cup_Wall.stl**, **rs_SPECIESNAME_Ant_Cup_Wall_Temp.stl**, **rs_SPECIESNAME_Post_Cup_Wall_Temp.stl** and **rs_SPECIESNAME_Lat_Cup_Wall_Temp.stl**.

40.

In the [Model Manager], right-click on all surface models and hide them except for the **rs_SPECIESNAME_Ant_Cup_Wall** and **rs_SPECIESNAME_Ant_Cup_Wall_Temp** models.

41.

a. In the [Model Manager], select the **rs_SPECIESNAME_Ant_Cup_Wall** model.
b. Click [Polygons>Boundaries>Create>Boundary From Spline].
c. In the [Graphics] window, define the first boundary of the cupula model using the overlaid **rs_SPECIESNAME_Ant_Cup_Wall_Temp** model as a guide (Fig.25) and click [Apply].
d. Define the second boundary of the cupula model using again the overlaid **rs_SPECIESNAME_Ant_Cup_Wall_Temp** model as a guide and click [Apply], then [OK].

42.

a. In the [Model Manager], select the **rs_SPECIESNAME_Ant_Cup_Wall** model.
b. In the [Graphics] window, click on the cupula model.
c. Click [Select>Data>Select Components>Bounded Components]. Click [Select>Data>Reverse] then press the [Del] key (Fig.25).

43.

In the [Model Manager], right-click the **rs_SPECIESNAME_Ant_Cup_Wall_Temp** model and select [Delete], then click the [Yes] button.

44.

a. In the [Model Manager], select the **rs_SPECIESNAME_Ant_Cup_Wall** model.
b. Click [Polygons>Repair>Decimate].
c. In the [Target Triangle Count] textbox enter ‘10000’.
d. Deselect [Fix Boundaries].
e. Select [Curvature Priority], using [Max].
f. Select [Mesh Priority], using [Max].
g. In [Maximum Aspect Ratio], select [Edge/Edge] and enter ‘3.0’ in the textbox.
h. Select [Edge/Height] and enter ‘3.5’ in the textbox and click [Apply], then [OK].

45.

Save the **rs_SPECIESNAME_Ant_Cup_Wall** model as a STL ascii file in folder **/AriadneToolbox/Data/SPECIESNAME/ScanRef/Surfaces/**.

46.

a. In the [Model Manager], right-click the **rs_SPECIESNAME_Ant_Cup_Wall** model and select [Duplicate].
b. Right-click the copied model and rename it as **rv_SPECIESNAME_Ant_Cup**.

47.

In the [Model Manager], right-click the **rs_SPECIESNAME_Ant_Cup_Wall** model and hide it.

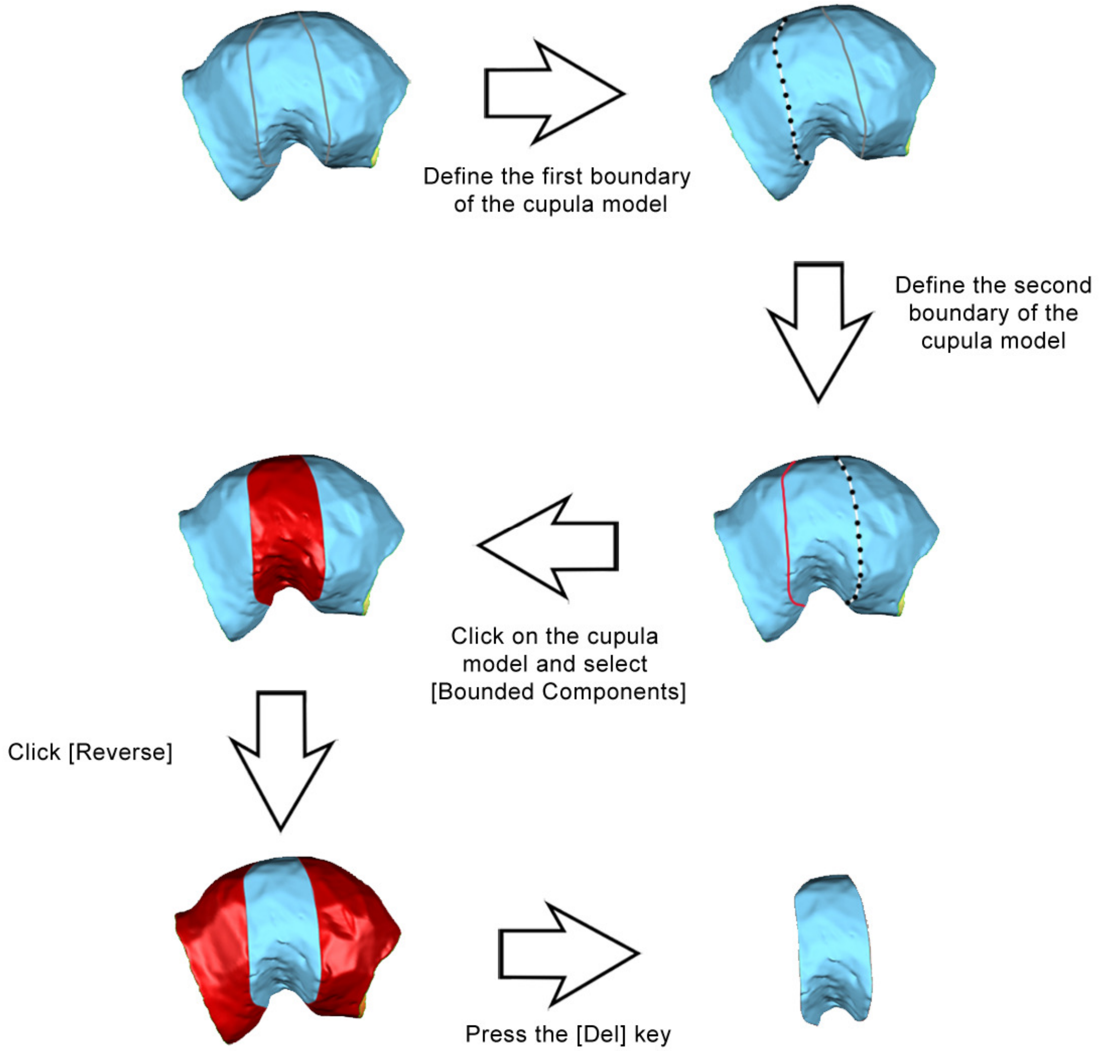

Figure 25. 3D model of the left anterior ampulla of a human, illustrating the procedure to build the ampullary walls of the cupula model in Geomagic Studio 12.

48.

a. In the [Model Manager], select the **rv_SPECIESNAME_Ant_Cup** model.
b. Click [Polygons>Fill Holes>Fill Single] with [Flat] and [Partial] options.
c. In the [Graphics] window, partially fill the first hole of the cupula model by creating triangulated surfaces running from the *crista ampullaris* up to the ampulla roof, until a 'rectangular' hole is left at the center.
d. Select the [Complete] option and click on the 'rectangular' hole.
e. Select the [Partial] option and repeat the procedure for the second hole of the cupula model.
f. Click [Fill Single] when the process is over (Fig.26).

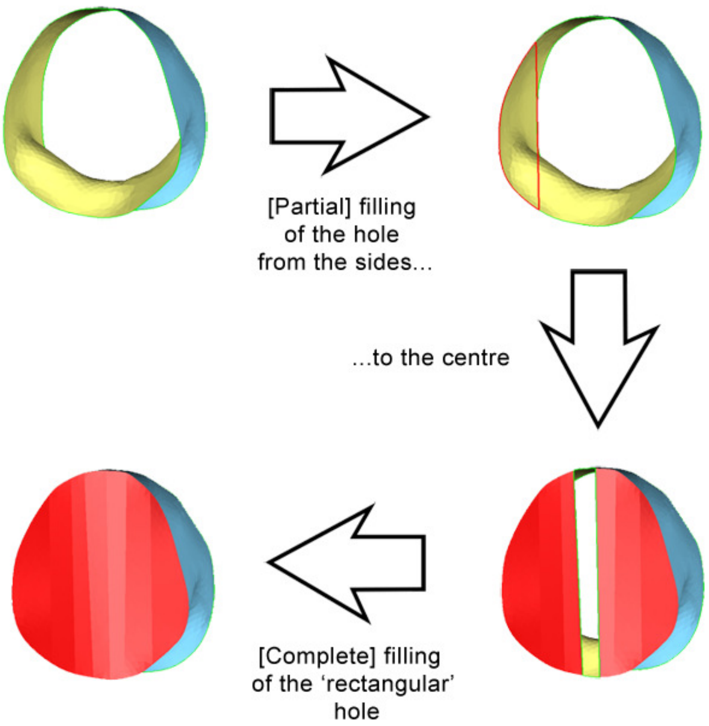

Figure 26. 3D model of the ampullary walls of the left anterior cupula model of a human, illustrating the procedure to fill the utricular and slender duct faces in Geomagic Studio 12.

49.

If filled holes intersect with the *crista ampullaris*:

a. Click [Select>Mode>Selection Mode>Select Backfaces].
b. In the [Graphics] window select the intersecting backfaces.
c. Click [Polygons>Offset>Offset>Offset Selection].
d. Increase the [Distance] in [Settings] and click [Apply] until no intersecting triangles can be seen, then click [OK] (Fig.27).
e. Make sure to deselect [Select>Mode>Selection Mode>Select Backfaces].

50.

Save the **rv_SPECIESNAME_Ant_Cup.stl** model as a STL ascii file in folder **/AriadneToolbox/Data/SPECIESNAME/ScanRef/Volumes/**.

51.

a. In the [Model Manager], right-click the **rv_SPECIESNAME_Ant_Cup** model and select [Duplicate].
b. Right-click the original and copied models and rename them as **rs_SPECIESNAME_Ant_Cup_Duct** and **rs_SPECIESNAME_Ant_Cup_Ut** respectively.

52.

a. Click [Geomagic>Import].
b. In the folder **/AriadneToolbox/Data/SPECIESNAME/ScanRef/Volumes/**, select the files **rv_SPECIESNAME_Ua.stl** and **rv_SPECIESNAME_Up.stl** and click [Open].
c. Specify the units that were used during the segmentation process and click [OK].

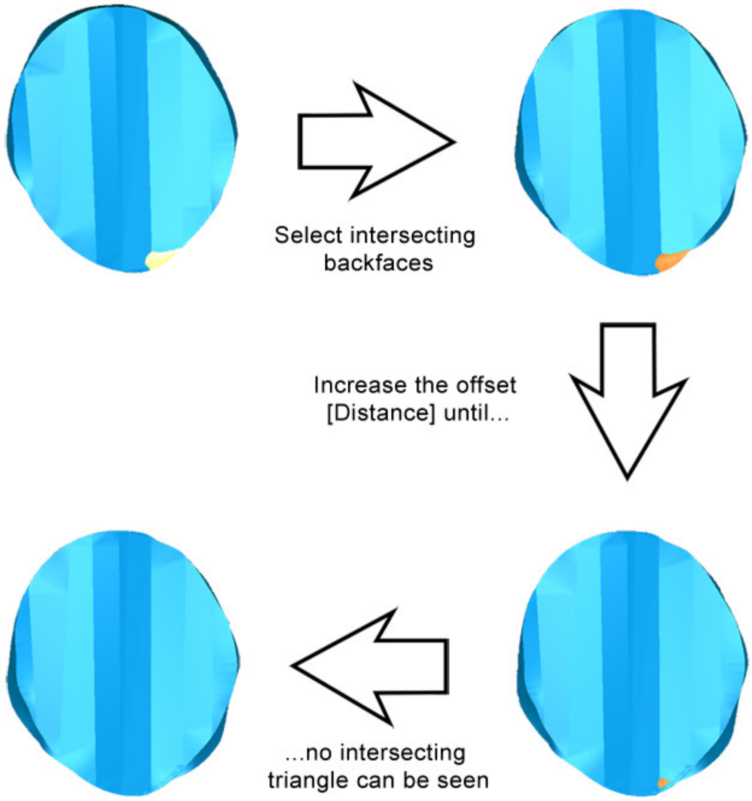

Figure 27. 3D model of the right anterior cupula model of a squirrel monkey, illustrating the procedure in Geomagic Studio 12 to remove intersecting triangles.

53. In the [Model Manager], right-click the **rs_SPECIESNAME_Ant_Cup_Ut** and **rv_SPECIESNAME_Up** models and hide them.

54.
    a. In the [Model Manager], select the **rs_SPECIESNAME_Ant_Cup_Duct** model.
    b. Click [Select>Select By>Crease Angle].
    c. In the [Graphics] window, click on the slender duct face of the cupula model.
    d. In the [Settings], adjust the [Angle] such that the whole slender duct face of the cupula model is selected and click [OK].
    e. Click [Select>Data>Reverse] then press the [Del] key (Fig.28).

55. Save the **rs_SPECIESNAME_Ant_Cup_Duct** model as a STL ascii file in folder **/AriadneToolbox/Data/SPECIESNAME/ScanRef/Surfaces/**.

56. In the [Model Manager], right-click the **rs_SPECIESNAME_Ant_Cup_Duct** model and hide it.

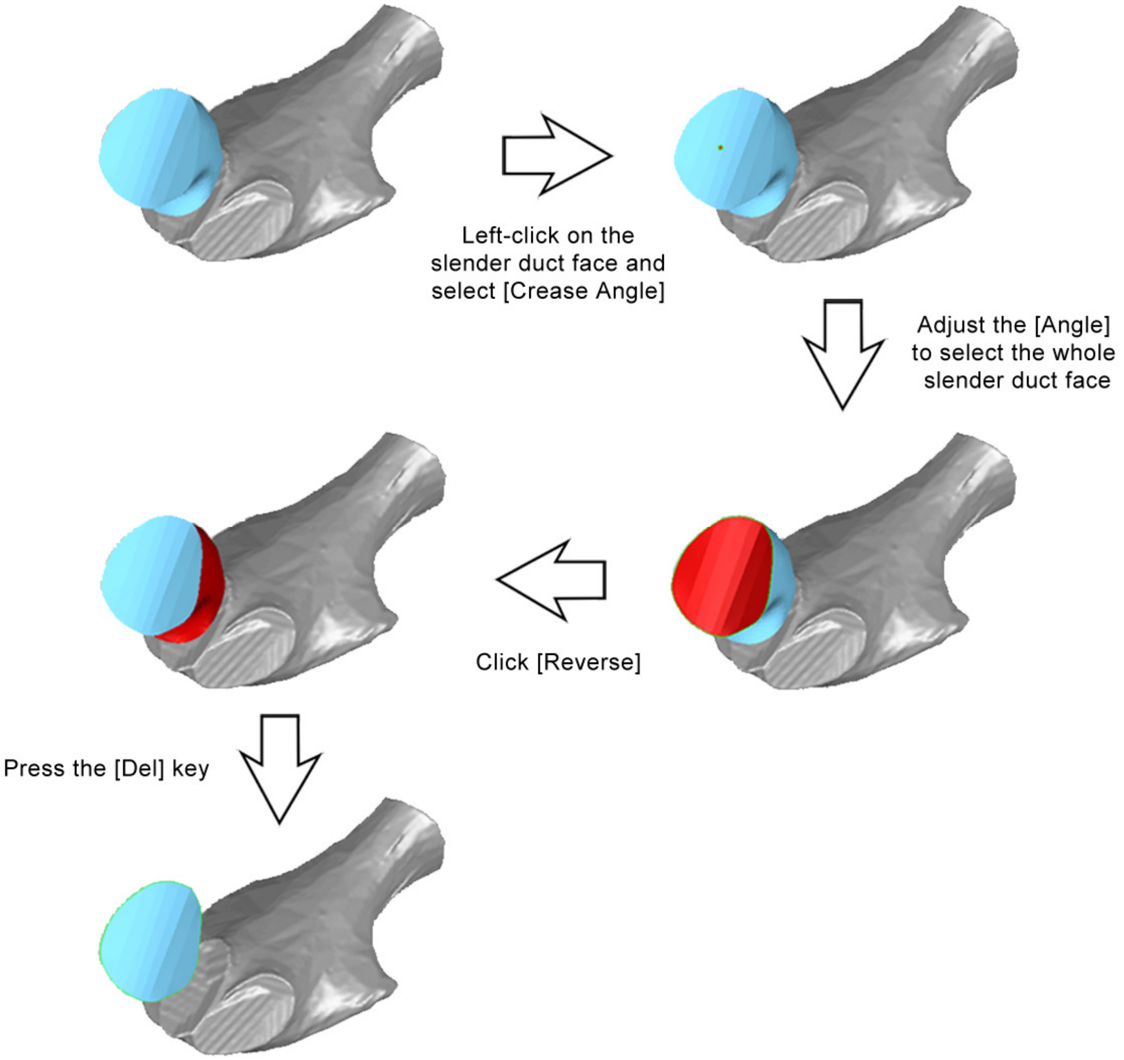

Figure 28. 3D model of the left anterior cupula model and anterior utricle of a human, illustrating the procedure in Geomagic Studio 12 to isolate the slender duct face of the cupula,.

57.

a. In the [Model Manager], select the **rs_SPECIESNAME_Ant_Cup_Ut** model.
b. Click [Select>Select By>Crease Angle].
c. In the [Graphics] window, click on the utricular face of the cupula model.
d. In the [Settings], adjust the [Angle] such that the whole utricular face of the cupula model is selected and click [OK].
e. Click [Select>Data>Reverse] then press the [Del] key.

58.

Save the **rs_SPECIESNAME_Ant_Cup_Ut** model as a STL ascii file in folder **/AriadneToolbox/Data/SPECIESNAME/ScanRef/Surfaces/**.

59.

In the [Model Manager], right-click the **rs_SPECIESNAME_Ant_Cup_Ut** and **rv_SPECIESNAME_Ua** models and hide them.

60.

In the [Model Manager], right-click the **rs_SPECIESNAME_Ant_Cup_Wall** model and rename it as **rs_SPECIESNAME_Ant_Cup_Mid**.

61.

a. In the [Model Manager], select the **rs_SPECIESNAME_Ant_Cup_Mid** model.
b. In the [Graphics] window, rotate the **rs_SPECIESNAME_Ant_Cup_Mid** model in order to overlay the roof of the ampulla with the *crista ampullaris* (Fig.29).
c. Click [Polygons>Repair>Trim>Trim with Sheet].
d. In the [Graphics] window, draw a line dividing the **rs_SPECIESNAME_Ant_Cup_Mid** model in half along its long axis (Fig.29).
e. In [Extrusion], click the [Create] button.
f. In [Operations], click the [Intersect Extrusion] button (Fig.29).
g. Make sure that [Delete Selection], [Fill intersection] and [Create Boundary] are selected and click [OK].

62.

a. In the [Graphics] window, click on the cross-section of the cupula.
b. Click [Select>Data>Select Components>Bounded Components].
c. Click [Select>Data>Reverse] then press the [Del] key (Fig.29).

63.

Save the **rs_SPECIESNAME_Ant_Cup_Mid** model as a STL ascii file in folder **/AriadneToolbox/Data/SPECIESNAME/ScanRef/Surfaces/**.

64.

a. In the [Model Manager], right-click the **rs_SPECIESNAME_Ant_Cup_Mid** model and hide it.
b. Right-click the **rs_SPECIESNAME_Post_Cup_Wall** and **rs_SPECIESNAME_Post_Cup_Wall_Temp** models and show them.

65.

Reiterate the steps 41 to 44 by replacing the **rs_SPECIESNAME_Ant_Cup_Wall** model by the **rs_SPECIESNAME_Post_Cup_Wall** model, the **rs_SPECIESNAME_Ant_Cup_Wall_Temp** model by the **rs_SPECIESNAME_Post_Cup_Wall_Temp** model and the term 'anterior' by the term 'posterior'.

66.

Save the **rs_SPECIESNAME_Post_Cup_Wall** model as a STL ascii file in folder **/AriadneToolbox/Data/SPECIESNAME/ScanRef/Surfaces/**.

67.

Reiterate the steps 46 to 49 by replacing the **rs_SPECIESNAME_Ant_Cup_Wall** model by the **rs_SPECIESNAME_Post_Cup_Wall** model and the **rv_SPECIESNAME_Ant_Cup** model by the **rv_SPECIESNAME_Post_Cup** model.

68.

Save the **rv_SPECIESNAME_Post_Cup.stl** model as a STL ascii file in folder **/AriadneToolbox/Data/SPECIESNAME/ScanRef/Volumes/**.

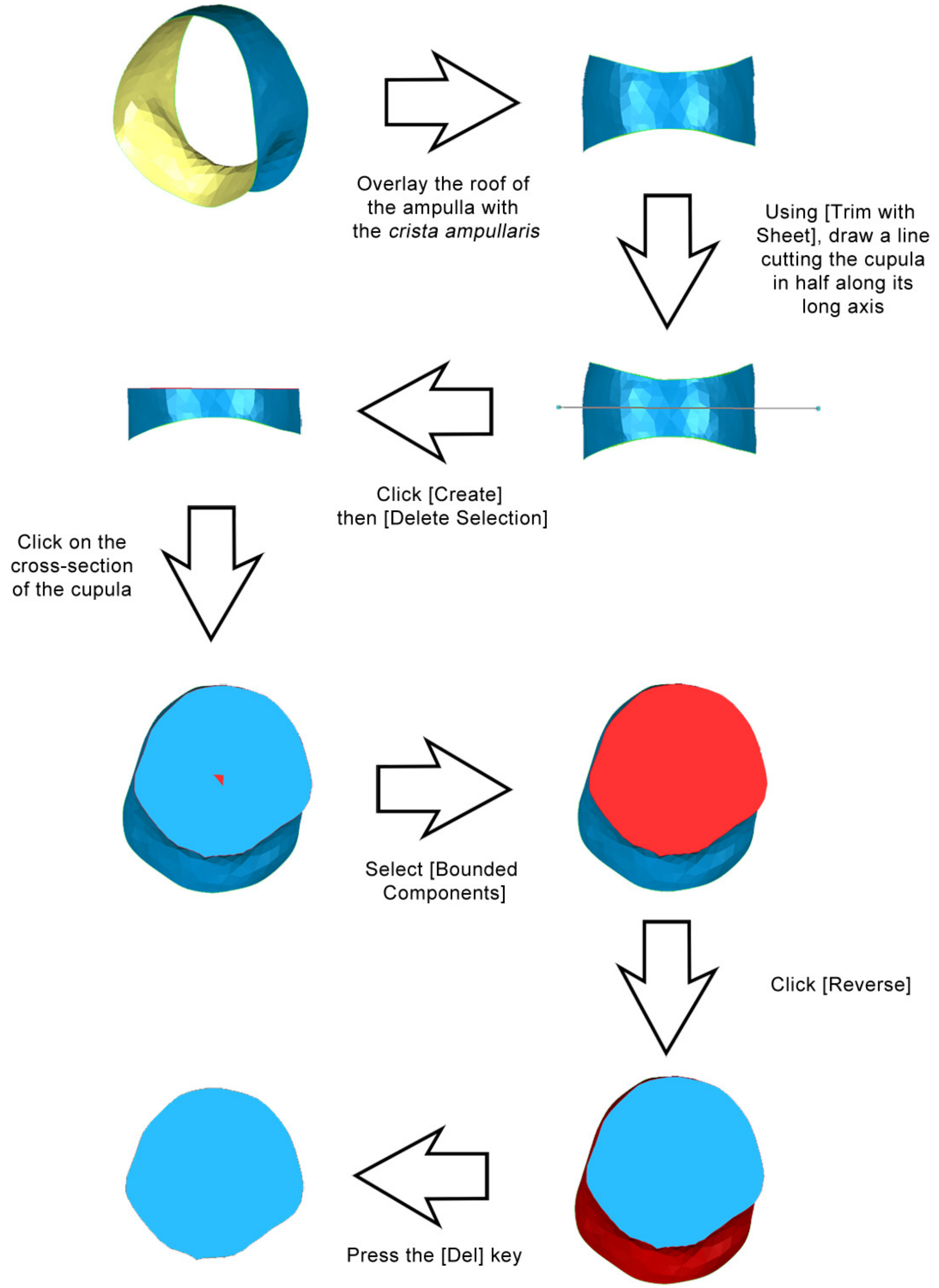

Figure 29. 3D model of the ampullary walls of the left anterior cupula model of a human, illustrating the procedure in Geomagic Studio 12 to isolate the middle cross-section of the cupula.

69.

a. In the [Model Manager], right-click the **rv_SPECIESNAME_Post_Cup** model and select [Duplicate].
b. Right-click the original and copied models and rename them as **rs_SPECIESNAME_Post_Cup_Duct** and **rs_SPECIESNAME_Post_Cup_Ut** respectively.

70.

In the [Model Manager], right-click the **rs_SPECIESNAME_Post_Cup_Ut** to hide it and the **rv_SPECIESNAME_Up** model to show it.

71.

Reiterate the step 54 by replacing the **rs_SPECIESNAME_Ant_Cup_Duct** model by the **rs_SPECIESNAME_Post_Cup_Duct** model and the term 'anterior' by the term 'posterior'.

72.

Save the **rs_SPECIESNAME_Post_Cup_Duct** model as a STL ascii file in folder **/AriadneToolbox/Data/SPECIESNAME/ScanRef/Surfaces/**.

73.

Reiterate the steps 56 to 57 by replacing the **rs_SPECIESNAME_Ant_Cup_Duct** model by the **rs_SPECIESNAME_Post_Cup_Duct** model, the **rs_SPECIESNAME_Ant_Cup_Ut** model by the **rs_SPECIESNAME_Post_Cup_Ut** model and the term 'anterior' by the term 'posterior'.

74.

Save the **rs_SPECIESNAME_Post_Cup_Ut** model as a STL ascii file in folder **/AriadneToolbox/Data/SPECIESNAME/ScanRef/Surfaces/**.

75.

In the [Model Manager], right-click the **rs_SPECIESNAME_Post_Cup_Ut** and **rv_SPECIESNAME_Up** models and hide them.

76.

Reiterate the steps 60 to 62 by replacing the **rs_SPECIESNAME_Ant_Cup_Wall** model by the **rs_SPECIESNAME_Post_Cup_Wall** model and the **rs_SPECIESNAME_Ant_Cup_Mid** model by the **rs_SPECIESNAME_Post_Cup_Mid** model.

77.

Save the **rs_SPECIESNAME_Post_Cup_Mid** model as a STL ascii file in folder **/AriadneToolbox/Data/SPECIESNAME/ScanRef/Surfaces/**.

78.

a. In the [Model Manager], right-click the **rs_SPECIESNAME_Post_Cup_Mid** model and hide it.
b. Right-click the **rs_SPECIESNAME_Lat_Cup_Wall** and **rs_SPECIESNAME_Lat_Cup_Wall_Temp** models and show them.

79.

Reiterate the steps 41 to 44 by replacing the **rs_SPECIESNAME_Ant_Cup_Wall** model by the **rs_SPECIESNAME_Lat_Cup_Wall** model, the **rs_SPECIESNAME_Ant_Cup_Wall_Temp** model by the **rs_SPECIESNAME_Lat_Cup_Wall_Temp** model and the term 'anterior' by the term 'lateral'.

80. Save the **rs_SPECIESNAME_Lat_Cup_Wall** model as a STL ascii file in folder **/AriadneToolbox/Data/SPECIESNAME/ScanRef/Surfaces/**.

81. Reiterate the steps 46 to 49 by replacing the **rs_SPECIESNAME_Ant_Cup_Wall** model by the **rs_SPECIESNAME_Lat_Cup_Wall** model and the **rv_SPECIESNAME_Ant_Cup** model by the **rv_SPECIESNAME_Lat_Cup** model.

82. Save the **rv_SPECIESNAME_Lat_Cup.stl** model as a STL ascii file in folder **/AriadneToolbox/Data/SPECIESNAME/ScanRef/Volumes/**.

83.
    a. In the [Model Manager], right-click the **rv_SPECIESNAME_Lat_Cup** model and select [Duplicate].
    b. Right-click the original and copied models and rename them as **rs_SPECIESNAME_Lat_Cup_Duct** and **rs_SPECIESNAME_Lat_Cup_Ut** respectively.

84. In the [Model Manager], right-click the **rs_SPECIESNAME_Lat_Cup_Ut** to hide it and the **rv_SPECIESNAME_Ua** model to show it.

85. Reiterate the step 54 by replacing the **rs_SPECIESNAME_Ant_Cup_Duct** model by the **rs_SPECIESNAME_Lat_Cup_Duct** model and the term 'anterior' by the term 'lateral'.

86. Save the **rs_SPECIESNAME_Lat_Cup_Duct** model as a STL ascii file in folder **/AriadneToolbox/Data/SPECIESNAME/ScanRef/Surfaces/**.

87. Reiterate the steps 56 to 57 by replacing the **rs_SPECIESNAME_Ant_Cup_Duct** model by the **rs_SPECIESNAME_Lat_Cup_Duct** model and the **rs_SPECIESNAME_Ant_Cup_Ut** model by the **rs_SPECIESNAME_Lat_Cup_Ut** model.

88. Save the **rs_SPECIESNAME_Lat_Cup_Ut** model as a STL ascii file in folder **/AriadneToolbox/Data/SPECIESNAME/ScanRef/Surfaces/**.

89. In the [Model Manager], right-click the **rs_SPECIESNAME_Lat_Cup_Ut** and **rv_SPECIESNAME_Ua** models and hide them.

90. Reiterate the steps 60 to 62 by replacing the **rs_SPECIESNAME_Ant_Cup_Wall** model by the **rs_SPECIESNAME_Lat_Cup_Wall** model and the **rs_SPECIESNAME_Ant_Cup_Mid** model by the **rs_SPECIESNAME_Lat_Cup_Mid** model.

91. Save the **rs_SPECIESNAME_Lat_Cup_Mid** model as a STL ascii file in folder **/AriadneToolbox/Data/SPECIESNAME/ScanRef/Surfaces/**.

In the end, this full procedure provides the user with 3 volume meshes, corresponding to the 3D models of the three cupulae, and with 12 surface meshes. An overview of these 15 meshes is provided (Fig.30).

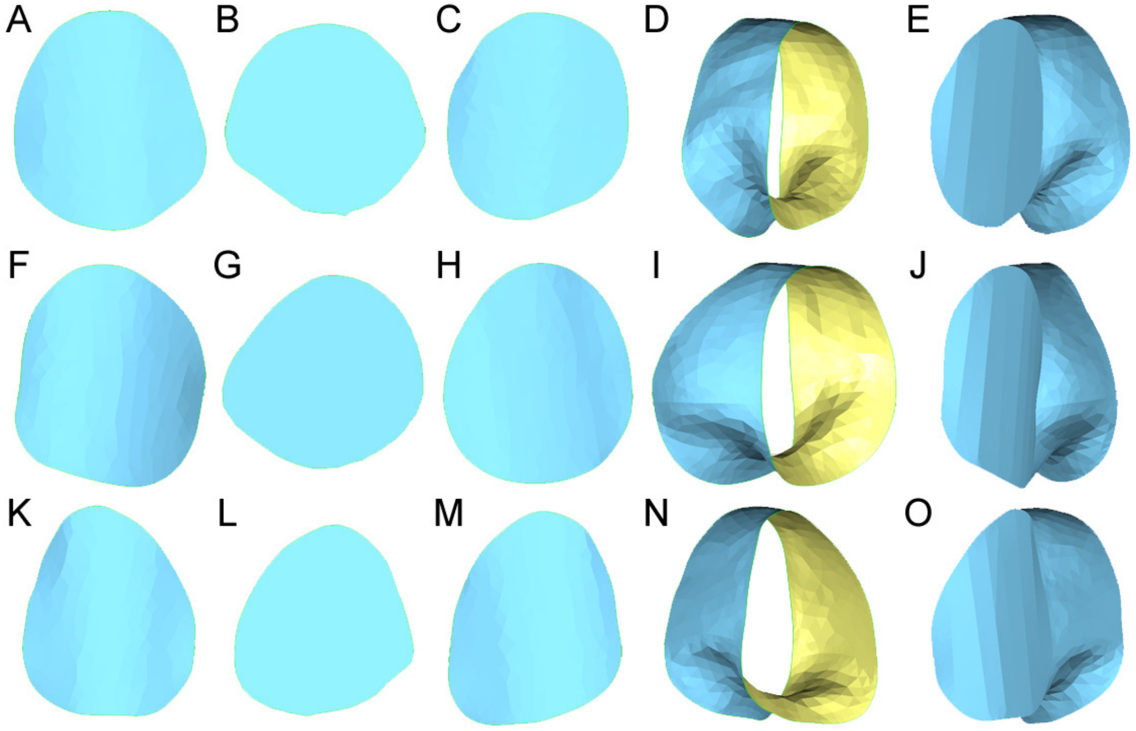

Figure 30. Overview of the 15 volume and surface STL files of the cupula models of the left semicircular duct system of a human. Surface meshes of the slender duct face (A, F, K), of the middle cross-section (B, G, L), of the utricular face (C, H, M) and of the ampullary walls (D, I, N) of the anterior, lateral and posterior models, respectively. Volume meshes (E, J, O) of the anterior (top row), lateral (middle row) and posterior (bottom row) models, respectively.

v. Preparing the finite element analyses of the cupulae

The fifth step of data preparation consists of dividing the middle cross-section of each cupula into four surface STL files, in order to delineate the main part of the cupula from three areas, associated with stereocilia and/or kinocilia of three different lengths. The resulting 12 surface STL files will be used to build three 2D finite element meshes of the cupulae (see section I.ii. of Ariadne Manual – Modules description), which in turn will be analyzed using the finite element method to obtain their deflection pattern (see section I.v. of Ariadne Manual – Modules description). This approach provides more precise information about cupula gross biomechanics than previous approaches[2-4]. It also allows quantifying the deflection of areas of the cupulae where stereocilia and/or kinocilia with various lengths can be found. As "shear strain between adjacent hair bundles […] is the key mechanical quantity leading to opening of transduction channels" (Ref 2, p.178), and thus to the modulation of afferent fibers firing rate, this approach provides the best biomechanical proxy, to date, for estimating semicircular duct sensitivity.

The principal reason the middle cross-section of the cupulae is used to build 2D finite element meshes, instead of adapting the full model of the cupula to build 3D finite element meshes[7] is practical. Most 3D finite element meshes built from CT scans are generated from triangulated surfaces and subsequently filled by tetrahedral elements, which are well-known to be highly sensitive to 'shear locking' and 'volumetric locking'. These two phenomena lead to important computational errors when considering deflection of nearly incompressible materials[8]. To tackle this problem while staying in three dimensions, one can either use higher order elements, which will automatically lead to exponentially larger, and sometimes impossible problems to solve, or use hexahedral elements by manually building CAD models of the cupula, a highly time consuming process that is not suitable for analyzing large and biologically diverse datasets. In light of these problems the most appropriate approach is to solve the shear deformable model of Reissner and Mindlin using the bi-dimensional stabilized MITC-plate elements, which are locking-free[9]. Indeed, it significantly speeds up the analyses, while providing accurate results, similar to well performed 3D analyses[7].

Here we describe how to divide the middle cross-section of each cupula using Geomagic Studio 12:

1.
    a. Click [Geomagic>Import].
    b. Select the file **/AriadneToolbox/Data/SPECIESNAME/ScanRef/Surfaces/rs_SPECIESNAME_Ant_Cup_Mid.stl**.
    c. Specify the units that were used during the segmentation process and click [OK].
    d. Decline using the [Mesh Doctor].
    e. Do the same for the files **rs_SPECIESNAME_Post_Cup_Mid.stl**, **rs_SPECIESNAME_Lat_Cup_Mid.stl**, **rs_SPECIESNAME_Ant_Cup_Wall.stl**, **rs_ SPECIESNAME_Post_Cup_Wall.stl** and **rs_SPECIESNAME_Lat_Cup_Wall.stl**.
2.
    In the [Model Manager], right-click all surface models and hide them except for the **rs_SPECIESNAME_Ant_Cup_Mid** and **rs_SPECIESNAME_Ant_Cup_Wall** models.

3.

a. In the [Model Manager], right-click the **rs_SPECIESNAME_Ant_Cup_Wall** model and select [Duplicate].
b. Do it twice.
c. Right-click the original and copied models and rename them as **Wall_30**, **Wall_60** and **Wall_90** respectively.

4.

a. In the [Model Manager], select the **Wall_30** model.
b. Click [Polygons>Offset>Offset>Offset Entire Model].
c. In the [Settings], enter '-30' µm in the [Distance] textbox, select [Smooth] and click [Apply], then [OK].
d. Select the **Wall_60** model.
e. Click [Polygons>Offset>Offset>Offset Entire Model].
f. In the [Settings], enter '-60' µm in the [Distance] textbox, select [Smooth] and click [Apply], then [OK].
g. Select the **Wall_90** model.
h. Click [Polygons>Offset>Offset>Offset Entire Model].
i. In the [Settings], enter '-90' µm in the [Distance] textbox, select [Smooth] and click [Apply], then [OK] (Fig. 31).

5.

a. In the [Model Manager], select both the **Wall_30** and the **rs_SPECIESNAME_Ant_Cup_Mid** models ([Ctrl] + [Left click]).
b. Click [Polygons>Combine>Boolean].
c. In [Operation], enter **Isoline_30** in the [Name] textbox and select [Intersect].
d. In [Options], select everything and click [Apply], then [OK].
e. Select both the **Wall_60** and the **rs_SPECIESNAME_Ant_Cup_Mid** models ([Ctrl] + [Left click]).
f. Click [Polygons>Combine>Boolean].
g. In [Operation], enter **Isoline_60** in the [Name] textbox and select [Intersect].
h. In [Options], select everything and click [Apply], then [OK].
i. Select both the **Wall_90** and the **rs_SPECIESNAME_Ant_Cup_Mid** models ([Ctrl] + [Left click]).
j. Click [Polygons>Combine>Boolean].
k. In [Operation], enter **Isoline_90** in the [Name] textbox and select [Intersect].
l. In [Options], select everything and click [Apply], then [OK] (Fig. 31).

6.

a. In the [Model Manager], select the **Wall_30**, **Wall_60** and **Wall_90** models ([Ctrl] + [Left click]).
b. Right-click the **Wall_30** model, select [Delete] and click the [Yes] button.

7.

a. In the [Model Manager], select the **rs_SPECIESNAME_Ant_Cup_Mid** model.
b. Click [Polygons>Smooth>Relax].
c. In the [Parameters], set [Smoothness Level], [Strength] and [Curvature Priority] to [Max].
d. Deselect [Fix Boundaries].

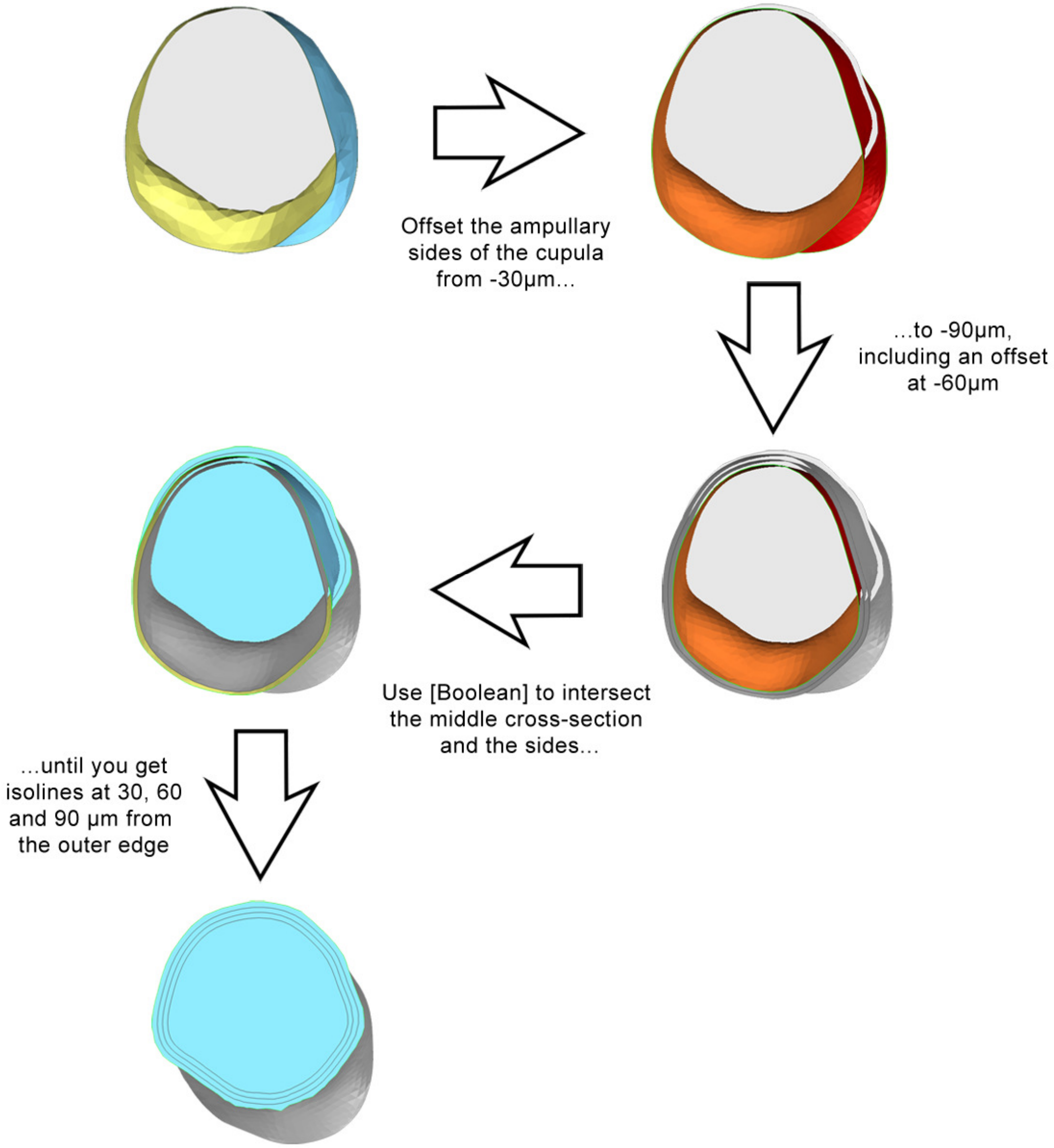

Figure 31. 3D models of the middle cross-section and the ampullary walls of the anterior cupula of a human, illustrating the procedure in Geomagic Studio 12 to define isolines running at 30, 60 and 90 µm from the outer edge of the cupula.

e. In the textbox of [Deviation Tolerance], enter the largest number possible and click [Apply], then [OK] (deviation statistics can be checked by clicking on [Deviations]).

8.

a. Click [Polygons>Repair>Remesh>Refine].
b. Select [4X Subdivision], [Move Vertices], [Fix Boundary] and click [Apply] until the number of triangles comes close to '30000', then on click [OK].

9.

c. Click [Select>Select All].
d. Click [Polygons>Repair>Rewrap].

e. In [Options], select [Create New Vertices].
f. Select [Optimize for Evenly Spaced Data] and click [Apply], then [OK].

10.
a. Click [Polygons>Repair>Decimate].
b. In the [Reduce to Percentage] textbox enter '99'.
c. Deselect [Fix Boundaries].
d. Select [Curvature Priority], using [Max].
e. Select [Mesh Priority], using [Max].
f. In [Maximum Aspect Ratio], select [Edge/Edge] and enter '3.0' in the textbox.
g. Select [Edge/Height], enter '3.5' in the textbox and click [Apply], then [OK].

11.
a. Click [Polygons>Repair>Remesh].
b. In the [Target Edge Length] textbox enter '1' µm.
c. Select [Use Existing Boundaries Only] and click [Apply], then [OK].

12.
a. Click [Polygons>Smooth>Relax].
b. In the [Parameters], set [Smoothness Level], [Strength] and [Curvature Priority] to [Max].
c. Deselect [Fix Boundaries].
d. In the textbox of [Deviation Tolerance], enter the largest number possible and click [Apply], then [OK] (deviation statistics can be checked by clicking on [Deviations]).

13.
a. Click [Polygons>Repair>Decimate].
b. In the [Reduce to Percentage] textbox enter '99'.
c. Deselect [Fix Boundaries].
d. Select [Curvature Priority], using [Max].
e. Select [Mesh Priority], using [Max].
f. In [Maximum Aspect Ratio], select [Edge/Edge] and enter '3.0' in the textbox.
g. Select [Edge/Height], enter '3.5' in the textbox and click [Apply], then [OK].

14.
a. Click [Polygons>Boundaries>Create>Boundary From Polyline].
b. In the [Graphics] window, define the boundary between the 30µm cilia region and the cupula, using the overlaid **Isoline_30** model as a guide. Stop at the level of the lateral margins of the *crista ampullaris*.
c. Click [Next Boundary] and define the boundary between the 60µm cilia region and the cupula, using the overlaid **Isoline_60** model as a guide. Stop at the same level than for the 30µm cilia region.
d. Click [Next Boundary] and define the boundary between the 90µm cilia region and the cupula, using the overlaid **Isoline_90** model as a guide. Stop at the same level than for the 30µm cilia region.
e. Click [Next Boundary] and define the first lateral boundary of the cilia regions by clicking on the *crista ampullaris* at the level of one of its lateral margin and extend this boundary perpendicularly such that it crosses the boundaries of the 30, 60 and 90µm cilia regions.

f. Click [Next Boundary] and do the same for the second lateral boundary of the cilia regions, then click [OK] (Fig.32).

15.

a. Click [Polygons>Boundaries>Remove>Remove Boundary].
b. Click on the intersecting boundary parts in the [Graphics] window to remove them, then click [OK] (Fig.32).

16.

a. In the [Model Manager], select the **Isoline_30**, **Isoline_60** and **Isoline_90** models ([Ctrl] + [Left click]).
b. Right-click the **Isoline_30** model, select [Delete] and click the [Yes] button.

17.

a. In the [Model Manager], select the **rs_SPECIESNAME_Ant_Cup_Mid** model.
b. Click [Polygons>Repair>Decimate].
c. In the [Target Triangle Count] textbox enter '50000'.
d. Select [Fix Boundaries].
e. Select [Curvature Priority], using [Max].
f. Select [Mesh Priority], using [Max].
g. In [Maximum Aspect Ratio], select [Edge/Edge] and enter '3.0' in the textbox.
h. Select [Edge/Height], enter '3.5' in the textbox and click [Apply], then [OK].

18.

a. In the [Model Manager], right-click the **rs_SPECIESNAME_Ant_Cup_Mid** model and select [Duplicate].
b. Do it three times.
c. Right-click the original and the copied models and rename them as **rfea_SPECIESNAME_Ant_Cup**, **rfea_SPECIESNAME_Ant_Cilia_Short**, **rfea_ SPECIESNAME_Ant_Cilia_Medium** and **rfea_SPECIESNAME_Ant_Cilia_Long** successively.

19.

a. In the [Model Manager], select the **rfea_SPECIESNAME_Ant_Cup** model.
b. In the [Graphics] window, click anywhere on the anterior cupula, excluding the cilia regions.
c. Click [Select>Data>Select Components>Bounded Components].
d. Click [Select>Data>Reverse] then press the [Del] key.
e. Do the same for the **rfea_SPECIESNAME_Ant_Cilia_Short**, **rfea_SPECIESNAME_ Ant_Cilia_Medium** and **rfea_SPECIESNAME_Ant_Cilia_Long** models by clicking anywhere on the lower, middle and higher cilia regions respectively (Fig.33).

20.

a. Save the **rfea_SPECIESNAME_Ant_Cup** model ('**lfea_'** instead of **'rfea_'** for a left labyrinth) as a STL ascii file in folder **/AriadneToolbox/Data/SPECIESNAME/FEA/**.
b. Do the same for the **rfea_SPECIESNAME_Ant_Cilia_Short**, **rfea_SPECIESNAME_ Ant_Cilia_Medium** and **rfea_SPECIESNAME_Ant_Cilia_Long** models, by replacing the filename suffix '**_Cup**' by '**_Cilia_Short**', '**_Cilia_Medium**' and '**_Cilia_Long**' respectively.

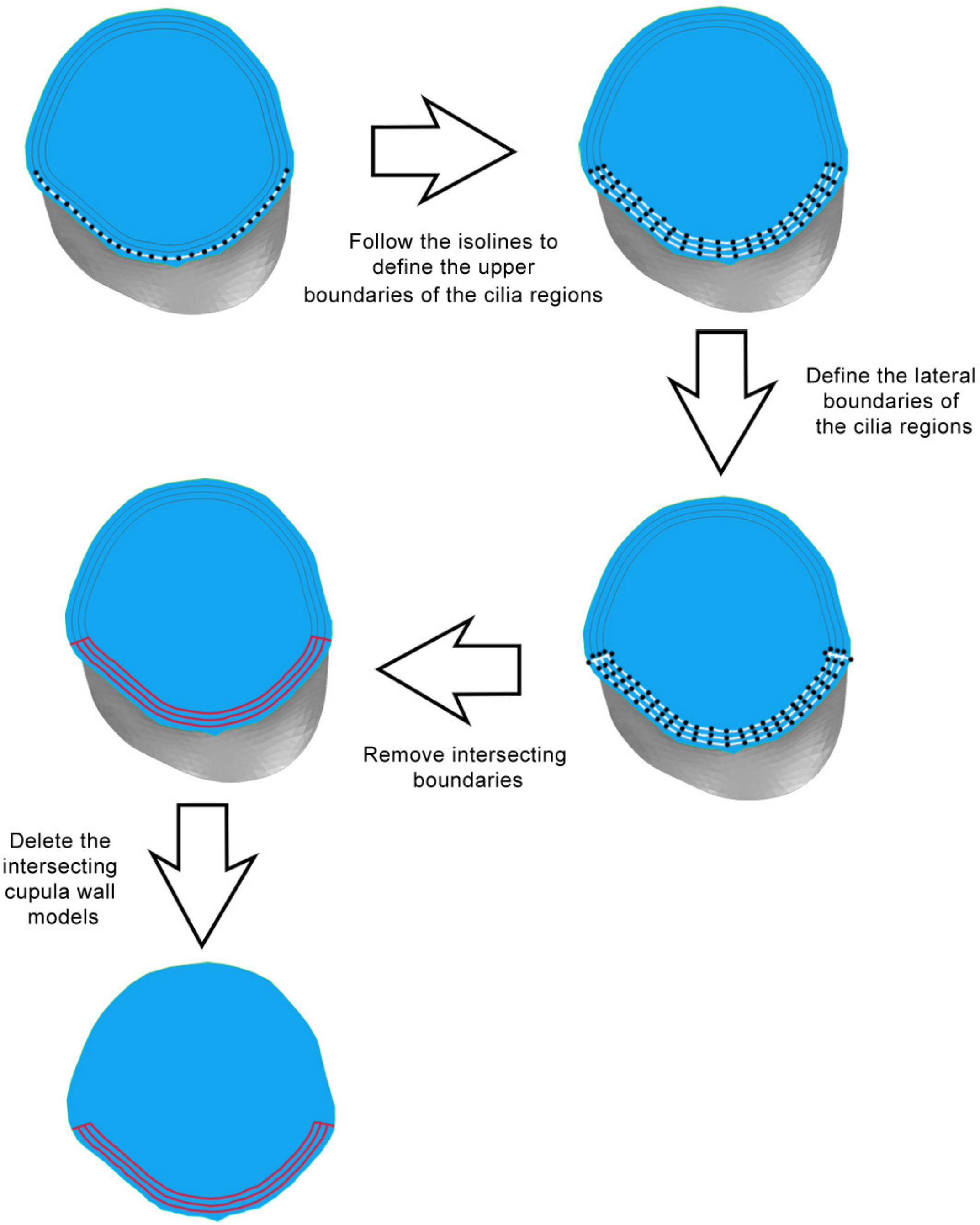

Figure 32. 3D model of the middle cross-section of the anterior cupula of a human, illustrating the procedure in Geomagic Studio 12 to define the boundaries of areas of the cupula where stereocilia and/or kinocilia with various lengths can be found.

21.

a. In the [Model Manager], right-click the **rfea_SPECIESNAME_Ant_Cup**, **rfea_SPECIESNAME_Ant_Cilia_Short**, **rfea_SPECIESNAME_Ant_Cilia_Medium** and **rfea_SPECIESNAME_Ant_Cilia_Long** models and hide them.
b. Right-click the **rs_SPECIESNAME_Post_Cup_Mid** and **rs_SPECIESNAME_Post_Cup_Wall** models and show them.

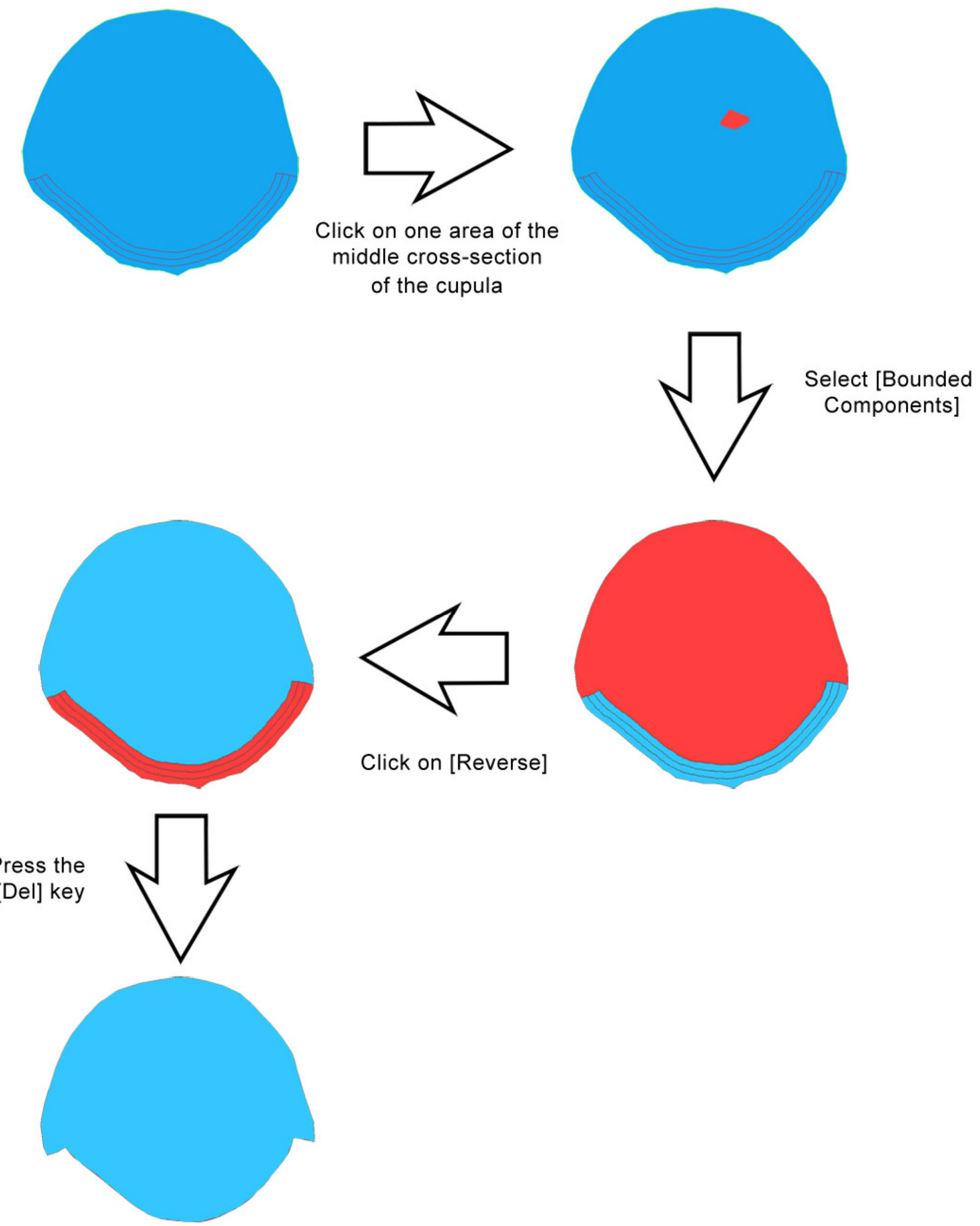

Figure 33. 3D model of the middle cross-section of the anterior cupula of a human, illustrating the procedure in Geomagic Studio 12 to isolate the main part of the cupula from the regions where stereocilia and/or kinocilia with various lengths can be found.

22. Reiterate the steps 3 to 19 by replacing the **rs_SPECIESNAME_Ant_Cup_Wall** model by the **rs_SPECIESNAME_Post_Cup_Wall** model, the **rs_SPECIESNAME_Ant_Cup_Mid** model by the **rs_SPECIESNAME_Post_Cup_Mid** model, the **rfea_SPECIESNAME_Ant_Cup** model by the **rfea_SPECIESNAME_Post_Cup** model, the **rfea_SPECIESNAME_Ant_Cilia_Short** model by the **rfea_SPECIESNAME_Post_Cilia_Short** model, the **rfea_SPECIESNAME_Ant_Cilia_Medium** model by the **rfea_SPECIESNAME_Post_**

Cilia_Medium model, the **rfea_SPECIESNAME_Ant_Cilia_Long** model by the **rfea_SPECIESNAME_Post_Cilia_Long** model and the term 'anterior' by the term 'posterior'.

23.

a. Save the **rfea_SPECIESNAME_Post_Cup** model as a STL ascii file in folder **/AriadneToolbox/Data/SPECIESNAME/FEA/**.
b. Do the same for the **rfea_SPECIESNAME_Post_Cilia_Short**, **rfea_SPECIESNAME_Post_Cilia_Medium** and **rfea_SPECIESNAME_Post_Cilia_Long** models, by replacing the filename suffix '**_Cup**' by '**_Cilia_Short**', '**_Cilia_Medium**' and '**_Cilia_Long**' respectively.

24.

a. In the [Model Manager], right-click the **rfea_SPECIESNAME_Post_Cup**, **rfea_SPECIESNAME_Post_Cilia_Short**, **rfea_SPECIESNAME_Post_Cilia_Medium** and **rfea_SPECIESNAME_Post_Cilia_Long** models and hide them.
b. Right-click the **rs_SPECIESNAME_Lat_Cup_Mid** and **rs_SPECIESNAME_Lat_Cup_Wall** models and show them.

25.

Reiterate the steps 3 to 19 by replacing the **rs_SPECIESNAME_Ant_Cup_Wall** model by the **rs_SPECIESNAME_Lat_Cup_Wall** model, the **rs_SPECIESNAME_Ant_Cup_Mid** model by the **rs_SPECIESNAME_Lat_Cup_Mid** model, the **rfea_SPECIESNAME_Ant_Cup** model by the **rfea_SPECIESNAME_Lat_Cup** model, the **rfea_SPECIESNAME_Ant_Cilia_Short** model by the **rfea_SPECIESNAME_Lat_Cilia_Short** model, the **rfea_SPECIESNAME_Ant_Cilia_Medium** model by the **rfea_SPECIESNAME_Lat_Cilia_Medium** model, the **rfea_SPECIESNAME_Ant_Cilia_Long** model by the **rfea_SPECIESNAME_Lat_Cilia_Long** model and the term 'anterior' by the term 'lateral'.

26.

a. Save the **rfea_SPECIESNAME_Lat_Cup** model as a STL ascii file in folder **/AriadneToolbox/Data/SPECIESNAME/FEA/**.
b. Do the same for the **rfea_SPECIESNAME_Lat_Cilia_Short**, **rfea_SPECIESNAME_Lat_Cilia_Medium** and **rfea_SPECIESNAME_Lat_Cilia_Long** models, by replacing the filename suffix '**_Cup**' by '**_Cilia_Short**', '**_Cilia_Medium**' and '**_Cilia_Long**' respectively.

This procedure provides the user with 3 sets of separated middle cross-section meshes of the cupulae that will be used to build finite element models (see section I.ii. of Ariadne Manual – Modules description). An overview of these 3 sets is provided (Fig.34).

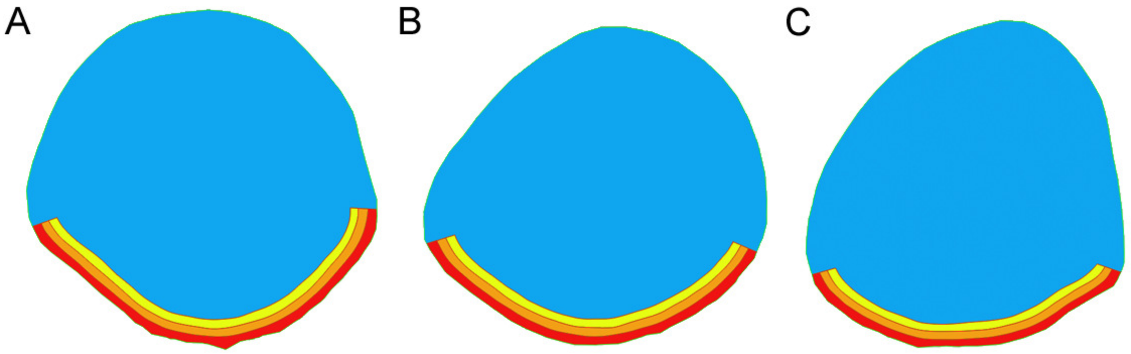

Figure 34. Overview of the 12 meshes that make up the FEA STL sets of the anterior (A), posterior (B) and lateral (C) cupulae of the left semicircular duct system of a human, based on the middle cupular cross-sections. The blue mesh represents the main part of the cupula, and the red, orange and yellow meshes the regions of the cupula where stereocilia/kinocilia can potentially be found with lengths of 0-30, 31-60 and 61-90 µm, respectively.

vi. Registering the mid-sagittal plane of the vestibular frame of reference

The sixth step of data preparation consists of recording the mid-sagittal plane of the vestibular frame of reference. The coordinates of this plane will mainly be used for building a complete vestibular frame of reference[5] (see section I.iii.8. of Ariadne Manual – Modules description), so that STL, lineset and landmark files can be transferred into this frame of reference (see section I.iv.1 of Ariadne Manual – Modules description), but also to perform directional sensitivity analyses (see section I.vi.3 of Ariadne Manual – Modules description). It should be noted that this particular step is not needed if all files described in the previous four steps were prepared for both the left and right membranous labyrinths of the same specimen. Here we describe how to register the mid-sagittal plane of the vestibular frame of reference using Avizo 7.1 but the same result can be achieved using other software:

1. If both bony labyrinths can be segmented in the reference scan that was used to segment the membranous labyrinth:

    a. Segment both bony labyrinths using the same label field.

    b. Generate a 3D mesh containing these two bony labyrinths. It is important that both the membranous and the bony labyrinths are kept in the same reference coordinate for the complete procedure to work (i.e. there should be no rotation or translation between their reconstructed meshes).

    c. Name the mesh containing both bony labyrinths **Both_SPECIESNAME_ BonyMP.ply** and save it in the folder **/AriadneToolbox/Data/SPECIESNAME/ ScanRef/Other/**.

    d. Go to step 2.

    If both bony labyrinths cannot be segmented in the reference scan that was used to segment the membranous labyrinth:

    a.
        i. Segment the bony labyrinth in the reference scan that was used to segment the membranous labyrinth.
        ii. Generate a 3D mesh of the bony labyrinth. It is important that both the membranous and the bony labyrinth are kept in the same reference coordinate for the complete procedure to work (i.e. there should be no rotation or translation between their reconstructed meshes).
        iii. Name the mesh of the bony labyrinth **r_SPECIESNAME_Bony.stl** and save it in the folder **/AriadneToolbox/Data/SPECIESNAME/ScanRef/Other/**.

b.

   i. Find another scan of the same species in which both bony labyrinths can be seen *in situ*.
   ii. On this new scan, segment the bony labyrinth that is from the same side of the head as **r_SPECIESNAME_Bony.stl**.
   iii. Generate a 3D mesh of this bony labyrinth.
   iv. Name the mesh of this bony labyrinth **r_SPECIESNAME_BonyMP.ply** and save it in the folder **/AriadneToolbox/Data/SPECIESNAME/ScanRef/Other/**.
   vi. Using the label field that was used to segment **r_SPECIESNAME_ BonyMP.ply**, also segment the bony labyrinth that is from the other side of the head.
   vii. Generate a 3D mesh of these bony labyrinths (the mesh should contain <u>both</u> the right and left labyrinths from the new scan).
   viii. Name the mesh of both these bony labyrinths **Both_SPECIESNAME_ BonyMP.ply** and save it in the folder **/AriadneToolbox/Data/ SPECIESNAME/ScanRef/Other/**.

c.

   i. In Avizo 7.1, click [Project View>Open Data].
   ii. In the folder **/AriadneToolbox/Data/SPECIESNAME/ScanRef/Other/**, select the files **r_SPECIESNAME_Bony.stl**, **r_SPECIESNAME_BonyMP.ply** and **Both_SPECIESNAME_BonyMP.ply** and click [Open].

d.

   i. In [Project View], right-click the **r_SPECIESNAME_Bony.stl** object and select [Display>Surface View].
   ii. Do the same for the **r_SPECIESNAME_BonyMP.ply** object.
   iii. Click the [Surface View] and [Surface View 2] objects.
   iv. In the [Colors] scroll of the [Properties] panel, select [constant].
   v. In the [Colormap] section of the [Properties] panel, click on the color box, pick two different colors and click [Apply]

e.

   i. In [Project View], right-click the **Both_SPECIESNAME_BonyMP.ply** object and select [Geometry Transforms>Copy Transformation].
   ii. Click the [Copy Transformation] object.
   iii. In [Properties], select the **Both_SPECIESNAME_BonyMP.ply** object in the [Data] scroll and the **r_SPECIESNAME_BonyMP.ply** object in the [Reference] scroll.

f.

   i. In [Project View], right-click the **r_SPECIESNAME_BonyMP.ply** object and select [Geometry Transforms>Align Surfaces].
   ii. Click the [Align Surfaces] object.
   iii. In [Properties], select the **r_SPECIESNAME_BonyMP.ply** object in the [Surface_to_be_transformed] scroll and the **r_SPECIESNAME_Bony.stl** object in the [Reference_surface] scroll.
   iv. In [Transformation], select [rigid + uniform scale].
   v. In [Align], click the [Center] button.

g.

   i. In [Project View], click the [Copy Transformation] object.
   ii. In [Properties], click [Apply].

h.

   i. In [Project View], click the [Align Surfaces] object.
   ii. In [Properties], section [Align], click the [Principal axes] button.

i.

   i. In [Project View], click the [Copy Transformation] object.
   ii. In [Properties], click [Apply].

j.

   i. In [Project View], click the [Align Surfaces] object.
   ii. In [Properties], section [Align], click the [Surfaces] button.

k.

   i. In [Project View], click the [Copy Transformation] object.
   ii. In [Properties], click [Apply] (Fig.35).

l.

   i. In [Project View], click the **Both_SPECIESNAME_BonyMP.ply** object.
   ii. In [Properties], click the [Transform editor] button.
   iii. In [Action] section, click the [Apply transform] button.
   iv. Save again the mesh **Both_SPECIESNAME_BonyMP.ply** in the folder **/AriadneToolbox/Data/SPECIESNAME/ScanRef/Other/**, by overwriting the previous version.

m.

   Go to step 2.

2.

   a. In Avizo 7.1, click [Project View>Open Data].
   b. Select the file **Both_SPECIESNAME_BonyMP.ply** and click [Open].

3.

   In [Project View], right-click the **Both_SPECIESNAME_BonyMP.ply** object and select [Display>Surface View].

4.

   In the [Visualization] space, with the [Hand], orient the view such that the two bony labyrinths best overlap (Fig. 36A).

5.

   In the [Console], type 'viewer rotate 90' and press the [Enter] key

6.

   a. In [Project View], right-click the object **Both_SPECIESNAME_BonyMP.ply** and select [Display> Clipping Plane].
   b. Click the [Clipping Plane] object.
   c. In [Options], select the [set plane] box.
   d. Play with the numbers in the [Plane Normal] section in order to align the [Clipping Plane] to the vertical axis of the screen (Fig.36B). Make sure the plane looks like a line in this configuration.

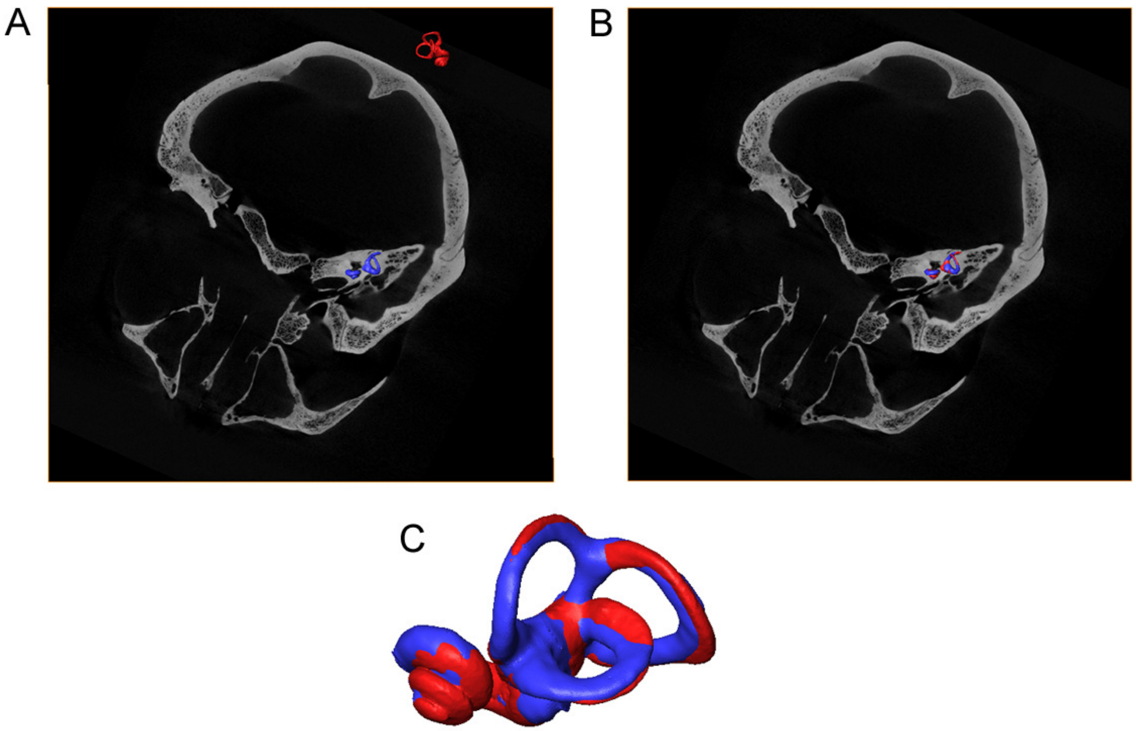

Figure 35. Views of the reference skull that will be used to register the mid-plane of the vestibular frame of reference before (A) and after (B) the spatial transformation. Note that initially neither the reference skull nor its bony labyrinth (in blue) were in the same frame of reference as the bony labyrinth enclosing the studied membranous labyrinth (in red). (C) The match between the two bony labyrinths after the spatial transformation operation.

e. Play with the numbers in the [Plane Point] section in order to place the [Clipping Plane] in-between the two bony labyrinths (Fig.36B). Although this specific sub-step does not affect any latter morphological or functional analysis, the [2D Measurement] tool of Avizo can potentially be used in order to help finding the best location for the [Plane Point].
f. Copy the values appearing in the [Plane Normal] section and the [Plane Point] section into an empty text file by following the format:

   Plane_Normal_x Plane_Normal_y Plane_Normal_z
   Plane_Point_x Plane_point_y Plane_Point_z

   Either Notepad or Notepad++ can be used to do this.
g. Name the text file **SPECIESNAME.midplane** and save it in the folder **/AriadneToolbox/Data/SPECIESNAME/Matrix/**.

This procedure provides the user with the coordinates of the midplane of the vestibular frame of reference.

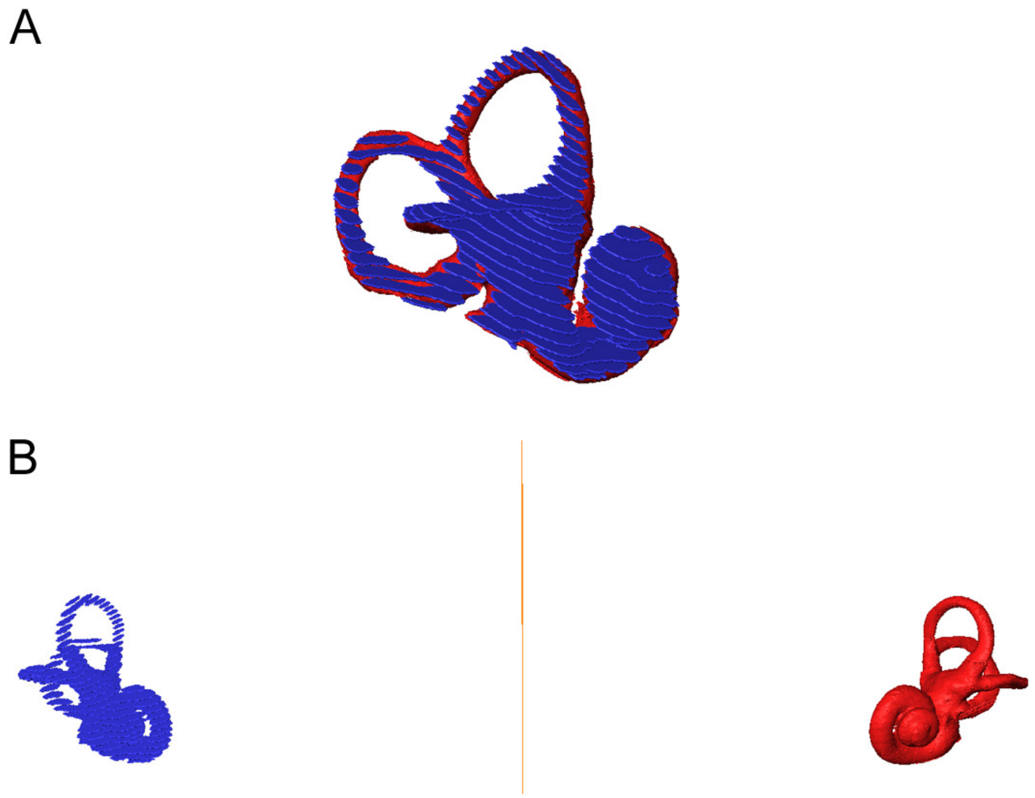

Figure 36. A, The overlap between the two bony labyrinths of Both_SPECIESNAME_BonyMP.ply in lateral view. B, The clipping plane (in orange) placed in-between these two bony labyrinths in an anterior view that is perpendicular to that shown in A. Note that the right bony labyrinth has not been fully segmented here, but that only each tenth slice was segmented to save time in the process.

References

1. David, R., Stoessel A., Berthoz, A., Spoor, F. & Bennequin, D. Assessing morphology and function of the semicircular duct system, introducing new in-situ visualization and software toolbox.
2. Highstein, S.M., Fay, R.R., & Popper, A.N. *The Vestibular System*. (Springer-Verlag, New York, 2004).
3. Oman, C.M., Marcus, E.N., & Curthoys, I.S. The influence of semicircular canal morphology on endolymph flow dynamics. An anatomically descriptive mathematical model. *Acta Otolaryngol*. **103**, 1-13 (1987).
4. Rabbitt, R.D. Directional coding of three-dimensional movements by the vestibular semicircular canals. *Biol Cybern*. **80**, 417-431 (1999).
5. David, R. et al. Motion from the past. A new method to infer vestibular capacities of extinct species. *C. R. Palevol*, **9**, 397-410 (2010).
6. Selva, P., Oman, C. M., & Stone, H. A. Mechanical properties and motion of the cupula of the human semicircular canal. *J Vestib Res*. **19**, 95-110 (2009).
7. Dohlman, G. F. The shape and function of the cupula. *J Laryngol Otol*. **83**, 43-53 (1969).
8. Bower, A. F. *Applied Mechanics of Solids.* (CRC Press, 2009).
9. Lyly, M. & Stenberg, R. Stabilized mitc plate bending elements. In *Advances in Finite Element Techniques* (Papadrakakis, M. & Topping, B. H. V., eds.). Civil Comp Press. 11-16 (1994).

Raw outputs of Ariadne

Homo sapiens (p.1)
Macaca mulatta (p.12)
Saimiri sciureus (p.23)

Homo sapiens

ENDOLYMPH VOLUMES

Anterior slender duct: 1.668632 mm^3
Posterior slender duct: 2.176416 mm^3
Lateral slender duct: 1.373401 mm^3

Common crus: 1.789232 mm^3
Simple crus: 0.473990 mm^3

Anterior utricle: 8.359233 mm^3
Posterior utricle: 1.432657 mm^3

Anterior ampulla: 2.017598 mm^3
Posterior ampulla: 1.582256 mm^3
Lateral ampulla: 2.115520 mm^3

Anterior duct: 13.834696 mm^3
Posterior duct: 6.980562 mm^3
Lateral duct: 12.322145 mm^3

Total SCDS volume: 22.011684 mm^3
Smoothing effect: -0.726177 %
Division error: -2.997551 %

STREAMLINE LENGTHS

Anterior slender duct: 15.489201 mm
Posterior slender duct: 18.843638 mm
Lateral slender duct: 15.273282 mm

Common crus (anterior trajectory): 3.442925 mm
Common crus (posterior trajectory): 3.434206 mm
Common crus (common part): 3.617736 mm
Simple crus: 1.674934 mm

Anterior utricle (anterior trajectory): 5.215839 mm
Anterior utricle (lateral trajectory): 5.210527 mm
Anterior utricle (common part): 4.147362 mm
Posterior utricle: 2.537371 mm

Anterior ampulla: 1.995775 mm
Posterior ampulla: 1.922879 mm
Lateral ampulla: 2.077083 mm

Anterior duct: 26.143740 mm
Posterior duct: 26.738093 mm
Lateral duct: 24.235827 mm

STREAMLINE ENCLOSED AREAS

Anterior duct: 46.363739 mm^2
Posterior duct: 50.639618 mm^2
Lateral duct: 41.050465 mm^2

RADII OF CURVATURE

Anterior duct: 4.001262 mm
Posterior duct: 4.135179 mm
Lateral duct: 3.736025 mm

CROSS-SECTIONAL AREAS

Anterior slender duct: 0.107729 mm^2
Posterior slender duct: 0.115499 mm^2
Lateral slender duct: 0.089922 mm^2

Common crus (anterior trajectory): 0.519684 mm^2
Common crus (posterior trajectory): 0.521004 mm^2
Common crus (common part): 0.521004 mm^2
Simple crus: 0.282990 mm^2

Anterior utricle (anterior trajectory): 1.602663 mm^2
Anterior utricle (lateral trajectory): 1.604297 mm^2
Anterior utricle (common part): 1.604297 mm^2
Posterior utricle: 0.564623 mm^2

Anterior ampulla: 1.010935 mm^2
Posterior ampulla: 0.822858 mm^2
Lateral ampulla: 1.018506 mm^2

Anterior duct: 0.529178 mm^2
Posterior duct: 0.261072 mm^2
Lateral duct: 0.508427 mm^2

SLENDER DUCT CROSS-SECTIONAL RADII

Anterior slender duct: 0.190597 mm
Posterior slender duct: 0.196884 mm
Lateral slender duct: 0.174887 mm

CROSS-SECTIONAL RATIOS

Common crus to anterior slender duct: 2.228080
Common crus to posterior slender duct: 2.149431

Anterior utricle to anterior slender duct: 4.041271
Anterior utricle to lateral slender duct: 4.457516
Posterior utricle to posterior slender duct: 2.360933

Anterior ampulla to anterior slender duct: 3.122356
Posterior ampulla to posterior slender duct: 3.396170

Lateral ampulla to lateral slender duct: 2.761152

WALL SHAPE DRAG FACTORS

Anterior slender duct: 28.160653
Posterior slender duct: 27.901934
Lateral slender duct: 28.636149

Common crus (anterior trajectory): 29.764642
Common crus (posterior trajectory): 29.225292
Common crus (common part): 29.494967
Simple crus: 43.033173

Anterior utricle (anterior trajectory): 33.634918
Anterior utricle (lateral trajectory): 35.101020
Anterior utricle (common part): 34.367969
Posterior utricle: 35.764484

Anterior ampulla: 30.310131
Posterior ampulla: 31.776648
Lateral ampulla: 29.656031

Anterior duct: 29.628124
Posterior duct: 29.096690
Lateral duct: 31.108433

CUPULA MORPHOMETRY

CUPULA CROSS-SECTIONAL AREAS

Anterior cupula (average): 1.266620 mm^2
Posterior cupula (average): 1.114714 mm^2
Lateral cupula (average): 1.248302 mm^2

Anterior cupula (central section): 1.125751 mm^2
Posterior cupula (central section): 1.008347 mm^2
Lateral cupula (central section): 1.147096 mm^2

CUPULA THICKNESS

Anterior cupula (long cilia): 0.596014 mm
Anterior cupula (medium cilia): 0.553594 mm
Anterior cupula (short cilia): 0.511174 mm

Posterior cupula (long cilia): 0.535803 mm
Posterior cupula (medium cilia): 0.493383 mm
Posterior cupula (short cilia): 0.450963 mm

Lateral cupula (long cilia): 0.624879 mm
Lateral cupula (medium cilia): 0.582459 mm
Lateral cupula (short cilia): 0.540039 mm

Anterior cupula (Rabbitt's empirical estimation): 0.419029 mm
Posterior cupula (Rabbitt's empirical estimation): 0.396577 mm
Lateral cupula (Rabbitt's empirical estimation): 0.422983 mm

SEMICIRCULAR DUCT PLANES (UNCOUPLED)

Right Anterior duct: 0.600927x 0.789214y 0.126604z
Right Posterior duct: 0.705954x -0.700855y 0.102140z
Right Lateral duct: -0.000000x -0.092974y -0.995669z
Left Anterior duct: -0.600927x 0.789214y -0.126604z
Left Posterior duct: -0.705954x -0.700855y -0.102140z
Left Lateral duct: -0.000000x -0.092974y 0.995669z

SEMICIRCULAR DUCT CENTER OF ROTATION (UNCOUPLED)

Right Anterior duct: -7990.608645x -77965.510766y -736.486847z
Right Posterior duct: -12863.911487x -78678.114217y -4280.943877z
Right Lateral duct: -9073.785854x -80456.380310y -3730.774890z
Left Anterior duct: -7990.608645x 2464.657752y -736.486847z
Left Posterior duct: -12863.911487x 3177.261203y -4280.943877z
Left Lateral duct: -9073.785854x 4955.527296y -3730.774890z

IPSILATERAL ANGLES (UNCOUPLED)

Anterior and Posterior ducts: 96.666481 Degrees
Anterior and Lateral ducts: 78.502090 Degrees
Lateral and Posterior ducts: 92.100662 Degrees

SYNERGISTIC ANGLES (UNCOUPLED)

Anterior and Posterior ducts: 172.018454 Degrees
Lateral ducts: 169.343149 Degrees

REFERENCE ANGLES (UNCOUPLED)

VESTIBULAR PITCH / MID-SAGITTAL PLANE

Anterior duct: 37.890716 Degrees
Posterior duct: 45.507751 Degrees
Lateral duct: 84.671540 Degrees

VESTIBULAR ROLL / CORONAL PLANE

Anterior duct: 53.067561 Degrees
Posterior duct: 45.096679 Degrees
Lateral duct: 90.000000 Degrees

VESTIBULAR YAW / HORIZONTAL PLANE

Anterior duct: 97.280540 Degrees
Posterior duct: 84.143767 Degrees
Lateral duct: 5.335020 Degrees

DEVIATION FROM IDEAL CONFIGURATION (UNCOUPLED)

Variance from orthogonality: 60.352226 Degrees^2

Average deviation from orthogonality: 6.755018 Degrees

Variance from planarity: 80.326207 Degrees^2

Average deviation from planarity: 8.873314 Degrees

SHRINKAGE CORRECTION

Factor slender duct cross-sectional diameter: 16.000000
Factor slender duct cross-sectional perimeter: 15.000000
Factor ampulla cross-sectional diameter: 14.000000
Factor ampulla cross-sectional perimeter: 18.000000
Factor utricle cross-sectional diameter: 31.000000
Factor utricle cross-sectional perimeter: 9.000000
Factor crus cross-sectional diameter: 24.000000
Factor crus cross-sectional perimeter: 12.000000

Shrinkage Correction Strength: 0.000000

CUPULA FINITE ELEMENT ANALYSIS

DEFORMATION PROFILE FACTORS

Anterior cupula (long cilia): 18.045159
Anterior cupula (medium cilia): 17.363525
Anterior cupula (short cilia): 16.588683

Posterior cupula (long cilia): 17.616539
Posterior cupula (medium cilia): 16.814357
Posterior cupula (short cilia): 15.895895

Lateral cupula (long cilia): 18.532301
Lateral cupula (medium cilia): 17.860051
Lateral cupula (short cilia): 17.100691

DEFLECTION CILIA AREAS

Anterior cupula (long cilia, high thickness): 0.277582 deg/nL
Anterior cupula (medium cilia, high thickness): 0.277300 deg/nL
Anterior cupula (medium cilia, medium thickness): 0.270714 deg/nL
Anterior cupula (short cilia, high thickness): 0.273009 deg/nL
Anterior cupula (short cilia, medium thickness): 0.265671 deg/nL
Anterior cupula (short cilia, low thickness): 0.257036 deg/nL

Posterior cupula (long cilia, high thickness): 0.323870 deg/nL
Posterior cupula (medium cilia, high thickness): 0.308324 deg/nL
Posterior cupula (medium cilia, medium thickness): 0.299589 deg/nL
Posterior cupula (short cilia, high thickness): 0.290009 deg/nL
Posterior cupula (short cilia, medium thickness): 0.280736 deg/nL
Posterior cupula (short cilia, low thickness): 0.269654 deg/nL

Lateral cupula (long cilia, high thickness): 0.270890 deg/nL
Lateral cupula (medium cilia, high thickness): 0.272085 deg/nL
Lateral cupula (medium cilia, medium thickness): 0.266060 deg/nL
Lateral cupula (short cilia, high thickness): 0.269912 deg/nL
Lateral cupula (short cilia, medium thickness): 0.263212 deg/nL
Lateral cupula (short cilia, low thickness): 0.255360 deg/nL

STIFFNESS

Anterior cupula (long cilia): 12.207997 g/s^2.mm^4
Anterior cupula (medium cilia): 10.910798 g/s^2.mm^4
Anterior cupula (short cilia): 9.625159 g/s^2.mm^4

Posterior cupula (long cilia): 14.146287 g/s^2.mm^4
Posterior cupula (medium cilia): 12.433150 g/s^2.mm^4
Posterior cupula (short cilia): 10.743421 g/s^2.mm^4

Lateral cupula (long cilia): 12.660115 g/s^2.mm^4
Lateral cupula (medium cilia): 11.372618 g/s^2.mm^4
Lateral cupula (short cilia): 10.096042 g/s^2.mm^4

STRUCTURAL PARAMETERS

MASS

Common crus (common part): 0.006944 g/mm^4
Anterior utricle (common part): 0.002585 g/mm^4

Anterior duct (long cilia): 0.155514 g/mm^4
Anterior duct (medium cilia): 0.155522 g/mm^4
Anterior duct (short cilia): 0.155531 g/mm^4

Posterior duct (long cilia): 0.176402 g/mm^4
Posterior duct (medium cilia): 0.176415 g/mm^4
Posterior duct (short cilia): 0.176429 g/mm^4

Lateral duct (long cilia): 0.180943 g/mm^4
Lateral duct (medium cilia): 0.180951 g/mm^4
Lateral duct (short cilia): 0.180959 g/mm^4

DAMPING

Common crus (common part): 0.320413 g/s.mm^4
Anterior utricle (common part): 0.045140 g/s.mm^4

Anterior duct (long cilia): 31.088117 g/s.mm^4
Anterior duct (medium cilia): 31.083337 g/s.mm^4
Anterior duct (short cilia): 31.078608 g/s.mm^4

Posterior duct (long cilia): 32.773844 g/s.mm^4
Posterior duct (medium cilia): 32.767969 g/s.mm^4
Posterior duct (short cilia): 32.762196 g/s.mm^4

Lateral duct (long cilia): 44.974105 g/s.mm^4
Lateral duct (medium cilia): 44.968933 g/s.mm^4
Lateral duct (short cilia): 44.963814 g/s.mm^4

INERTIAL FORCING

Anterior duct: 0.092727 g/mm
Posterior duct: 0.101279 g/mm
Lateral duct: 0.082101 g/mm

DYNAMIC PARAMETERS

TIME CONSTANTS

LONG TIME CONSTANTS

First constant (long cilia): 2.544035 s
First constant (medium cilia): 2.847279 s
First constant (short cilia): 3.229174 s

Second constant (long cilia): 2.308867 s

Second constant (medium cilia): 2.626696 s
Second constant (short cilia): 3.038811 s

Third constant (long cilia): 3.548410 s
Third constant (medium cilia): 3.950127 s
Third constant (short cilia): 4.449597 s

SHORT TIME CONSTANTS

First constant (long cilia): 4.952391 ms
First constant (medium cilia): 4.952402 ms
First constant (short cilia): 4.952415 ms

Second constant (long cilia): 5.455615 ms
Second constant (medium cilia): 5.455454 ms
Second constant (short cilia): 5.455297 ms

Third constant (long cilia): 4.023540 ms
Third constant (medium cilia): 4.023711 ms
Third constant (short cilia): 4.023880 ms

BANDWIDTH

LOWER CORNER FREQUENCIES

First frequency (long cilia): 0.062560 Hz
First frequency (medium cilia): 0.055897 Hz
First frequency (short cilia): 0.049287 Hz

Second frequency (long cilia): 0.068932 Hz
Second frequency (medium cilia): 0.060591 Hz
Second frequency (short cilia): 0.052374 Hz

Third frequency (long cilia): 0.044852 Hz
Third frequency (medium cilia): 0.040291 Hz
Third frequency (short cilia): 0.035768 Hz

UPPER CORNER FREQUENCIES

First frequency (long cilia): 32.136993 Hz
First frequency (medium cilia): 32.136917 Hz
First frequency (short cilia): 32.136834 Hz

Second frequency (long cilia): 29.172686 Hz
Second frequency (medium cilia): 29.173548 Hz
Second frequency (short cilia): 29.174389 Hz

Third frequency (long cilia): 39.555946 Hz
Third frequency (medium cilia): 39.554271 Hz
Third frequency (short cilia): 39.552605 Hz

IN-PLANE SENSITIVITY PARAMETERS

MECHANICAL GAIN

Anterior duct (cilia average deflection, long cilia): 2.887655 deg/deg
Anterior duct (cilia average deflection, medium cilia): 2.806180 deg/deg
Anterior duct (cilia average deflection, short cilia): 2.689294 deg/deg

Posterior duct (cilia average deflection, long cilia): 3.102992 deg/deg
Posterior duct (cilia average deflection, medium cilia): 2.928754 deg/deg
Posterior duct (cilia average deflection, short cilia): 2.721540 deg/deg

Lateral duct (cilia average deflection, long cilia): 2.138708 deg/deg
Lateral duct (cilia average deflection, medium cilia): 2.088684 deg/deg
Lateral duct (cilia average deflection, short cilia): 2.015385 deg/deg

VELOCITY GAIN

Anterior duct (cilia average deflection, long cilia): 0.014384 deg/deg.s-1
Anterior duct (cilia average deflection, medium cilia): 0.013981 deg/deg.s-1
Anterior duct (cilia average deflection, short cilia): 0.013401 deg/deg.s-1

Posterior duct (cilia average deflection, long cilia): 0.016600 deg/deg.s-1
Posterior duct (cilia average deflection, medium cilia): 0.015672 deg/deg.s-1
Posterior duct (cilia average deflection, short cilia): 0.014567 deg/deg.s-1

Lateral duct (cilia average deflection, long cilia): 0.008630 deg/deg.s-1
Lateral duct (cilia average deflection, medium cilia): 0.008430 deg/deg.s-1
Lateral duct (cilia average deflection, short cilia): 0.008135 deg/deg.s-1

ACCELERATION GAIN

Anterior duct (cilia average deflection, long cilia): 0.036584 deg/deg.s-2
Anterior duct (cilia average deflection, medium cilia): 0.039781 deg/deg.s-2
Anterior duct (cilia average deflection, short cilia): 0.043219 deg/deg.s-2

Posterior duct (cilia average deflection, long cilia): 0.038411 deg/deg.s-2
Posterior duct (cilia average deflection, medium cilia): 0.041253 deg/deg.s-2
Posterior duct (cilia average deflection, short cilia): 0.044367 deg/deg.s-2

Lateral duct (cilia average deflection, long cilia): 0.030669 deg/deg.s-2
Lateral duct (cilia average deflection, medium cilia): 0.033344 deg/deg.s-2
Lateral duct (cilia average deflection, short cilia): 0.036243 deg/deg.s-2

FUNCTIONAL PLANES

MAXIMAL RESPONSE PLANES

Right Anterior duct: 0.592846x 0.795726y 0.123909z
Right Posterior duct: 0.699584x -0.707497y 0.100155z
Right Lateral duct: 0.000000x -0.091697y -0.995787z
Left Anterior duct: -0.592846x 0.795726y -0.123909z
Left Posterior duct: -0.699584x -0.707497y -0.100155z
Left Lateral duct: -0.000000x -0.091697y 0.995787z

PRIME DIRECTIONS

Right Anterior duct: 0.713700x 0.696637y -0.064150z
Right Posterior duct: 0.781012x -0.590348y 0.054362z
Right Lateral duct: 0.167361x 0.027309y -0.976114z
Left Anterior duct: -0.713700x 0.696637y 0.064150z
Left Posterior duct: -0.781012x -0.590348y -0.054362z
Left Lateral duct: -0.167361x 0.027309y 0.976114z

IPSILATERAL ANGLES

Anterior and Posterior ducts: 97.813141 Degrees

Anterior and Lateral ducts: 78.682027 Degrees
Lateral and Posterior ducts: 92.004363 Degrees

SYNERGISTIC ANGLES

Anterior and Posterior ducts: 171.955655 Degrees
Lateral ducts: 169.490012 Degrees

REFERENCE ANGLES

VESTIBULAR PITCH / MID-SAGITTAL PLANE

Anterior duct: 37.278833 Degrees
Posterior duct: 44.971718 Degrees
Lateral duct: 84.745006 Degrees

VESTIBULAR ROLL / CORONAL PLANE

Anterior duct: 53.644738 Degrees
Posterior duct: 45.609730 Degrees
Lateral duct: 90.000000 Degrees

VESTIBULAR YAW / HORIZONTAL PLANE

Anterior duct: 97.124929 Degrees
Posterior duct: 84.258133 Degrees
Lateral duct: 5.261624 Degrees

DEVIATION FROM IDEAL CONFIGURATION

Variance from orthogonality: 64.386391 Degrees^2

Average deviation from orthogonality: 7.045159 Degrees

Variance from planarity: 79.960946 Degrees^2

Average deviation from planarity: 8.866226 Degrees

SEMICIRCULAR DUCT CENTER OF ROTATION

Right Anterior duct: -6015.370061x -75313.354916y -315.683451z
Right Posterior duct: -10536.378138x -81036.200358y -3934.548169z
Right Lateral duct: -9077.429490x -80762.006088y -7040.858657z
Left Anterior duct: -9967.279872x 5116.813602y -1141.663012z
Left Posterior duct: -15199.804400x 819.175062y -4602.178677z
Left Lateral duct: -9077.429490x 4649.901518y -402.942754z

MATERIAL PROPERTIES

Body temperature: 37.000000 C

Endolymph density: 1000.000000 kg/m^3
Endolymph viscosity: 0.000815 Pa.s
Endolymph viscosity factor: 2.850000 e-5 Pa.s

Cupula density: 1000.000000 kg/m^3
Cupula viscosity: 0.008151 Pa.s
Cupula Poisson ratio: 0.480000
Cupula Young modulus: 4.258000 Pa

Cupula Shear modulus: 1.438514 Pa

SATURATING MECHANICAL GAIN OF THE SEMICIRCULAR DUCT SYSTEM TO ROTATIONS IN SPACE

- MAXIMUM VALUE AMONG THE ABSOLUTE MECHANICAL GAINS OF THE SIX SEMICIRCULAR DUCTS, WEIGHTED ACCORDING TO INHIBITION/ACTIVATION
-- TO ANGULAR VELOCITY
--- MEASURED IN UNITS OF CILIA AVERAGE DEFLECTION

Nose-down Pitch (long cilia): 0.011744 deg/deg.s-1
Nose-down Pitch (medium cilia): 0.011088 deg/deg.s-1
Nose-down Pitch (short cilia): 0.010306 deg/deg.s-1

Nose-up Pitch (long cilia): 0.011446 deg/deg.s-1
Nose-up Pitch (medium cilia): 0.011125 deg/deg.s-1
Nose-up Pitch (short cilia): 0.010664 deg/deg.s-1

Roll (long cilia): 0.011613 deg/deg.s-1
Roll (medium cilia): 0.010964 deg/deg.s-1
Roll (short cilia): 0.010191 deg/deg.s-1

Yaw (long cilia): 0.008594 deg/deg.s-1
Yaw (medium cilia): 0.008394 deg/deg.s-1
Yaw (short cilia): 0.008101 deg/deg.s-1

Global mean (long cilia): 0.010466 deg/deg.s-1
Global mean (medium cilia): 0.010065 deg/deg.s-1
Global mean (short cilia): 0.009549 deg/deg.s-1

Global standard deviation (long cilia): 0.002627 deg/deg.s-1
Global standard deviation (medium cilia): 0.002439 deg/deg.s-1
Global standard deviation (short cilia): 0.002229 deg/deg.s-1

Global maximum (long cilia): 0.016600 deg/deg.s-1
Global maximum (medium cilia): 0.015672 deg/deg.s-1
Global maximum (short cilia): 0.014567 deg/deg.s-1

Global minimum (long cilia): 0.005982 deg/deg.s-1
Global minimum (medium cilia): 0.005766 deg/deg.s-1
Global minimum (short cilia): 0.005562 deg/deg.s-1

SATURATING MECHANICAL GAIN OF THE SEMICIRCULAR DUCT SYSTEM TO ROTATIONS IN SPACE

- MAXIMUM VALUE AMONG THE ABSOLUTE MECHANICAL GAINS OF THE SIX SEMICIRCULAR DUCTS, WEIGHTED ACCORDING TO INHIBITION/ACTIVATION
-- TO ANGULAR ACCELERATION
--- MEASURED IN UNITS OF CILIA AVERAGE DEFLECTION

Nose-down Pitch (long cilia): 0.027176 deg/deg.s-1
Nose-down Pitch (medium cilia): 0.029186 deg/deg.s-1
Nose-down Pitch (short cilia): 0.031390 deg/deg.s-1

Nose-up Pitch (long cilia): 0.029111 deg/deg.s-1
Nose-up Pitch (medium cilia): 0.031655 deg/deg.s-1
Nose-up Pitch (short cilia): 0.034390 deg/deg.s-1

Roll (long cilia): 0.026872 deg/deg.s-1

Roll (medium cilia): 0.028860 deg/deg.s-1
Roll (short cilia): 0.031038 deg/deg.s-1

Yaw (long cilia): 0.030540 deg/deg.s-1
Yaw (medium cilia): 0.033204 deg/deg.s-1
Yaw (short cilia): 0.036090 deg/deg.s-1

Global mean (long cilia): 0.028969 deg/deg.s-1
Global mean (medium cilia): 0.031386 deg/deg.s-1
Global mean (short cilia): 0.034007 deg/deg.s-1

Global standard deviation (long cilia): 0.003914 deg/deg.s-1
Global standard deviation (medium cilia): 0.004192 deg/deg.s-1
Global standard deviation (short cilia): 0.004502 deg/deg.s-1

Global maximum (long cilia): 0.038410 deg/deg.s-1
Global maximum (medium cilia): 0.041252 deg/deg.s-1
Global maximum (short cilia): 0.044366 deg/deg.s-1

Global minimum (long cilia): 0.017562 deg/deg.s-1
Global minimum (medium cilia): 0.019096 deg/deg.s-1
Global minimum (short cilia): 0.020539 deg/deg.s-1

STANDARDIZED MECHANICAL GAIN OF THE SEMICIRCULAR DUCT SYSTEM TO ROTATIONS IN SPACE

- OF THE THREE SYNERGISTIC SEMICIRCULAR DUCT PAIRS
-- TO ANGULAR VELOCITY
--- MEASURED IN UNITS OF CILIA AVERAGE DEFLECTION

Pitch (long cilia): 0.010932 deg/deg.s-1
Pitch (medium cilia): 0.010471 deg/deg.s-1
Pitch (short cilia): 0.009885 deg/deg.s-1

Roll (long cilia): 0.009494 deg/deg.s-1
Roll (medium cilia): 0.009076 deg/deg.s-1
Roll (short cilia): 0.008549 deg/deg.s-1

Yaw (long cilia): 0.005955 deg/deg.s-1
Yaw (medium cilia): 0.005809 deg/deg.s-1
Yaw (short cilia): 0.005597 deg/deg.s-1

Global mean (long cilia): 0.008230 deg/deg.s-1
Global mean (medium cilia): 0.007923 deg/deg.s-1
Global mean (short cilia): 0.007524 deg/deg.s-1

Global standard deviation (long cilia): 0.001639 deg/deg.s-1
Global standard deviation (medium cilia): 0.001536 deg/deg.s-1
Global standard deviation (short cilia): 0.001415 deg/deg.s-1

Global maximum (long cilia): 0.010932 deg/deg.s-1
Global maximum (medium cilia): 0.010471 deg/deg.s-1
Global maximum (short cilia): 0.009885 deg/deg.s-1

Global minimum (long cilia): 0.005605 deg/deg.s-1
Global minimum (medium cilia): 0.005471 deg/deg.s-1
Global minimum (short cilia): 0.005276 deg/deg.s-1

STANDARDIZED MECHANICAL GAIN OF THE SEMICIRCULAR DUCT SYSTEM TO ROTATIONS IN SPACE

- OF THE THREE SYNERGISTIC SEMICIRCULAR DUCT PAIRS
-- TO ANGULAR ACCELERATION
--- MEASURED IN UNITS OF CILIA AVERAGE DEFLECTION

Pitch (long cilia): 0.026534 deg/deg.s-2
Pitch (medium cilia): 0.028681 deg/deg.s-2
Pitch (short cilia): 0.031009 deg/deg.s-2

Roll (long cilia): 0.022892 deg/deg.s-2
Roll (medium cilia): 0.024722 deg/deg.s-2
Roll (short cilia): 0.026710 deg/deg.s-2

Yaw (long cilia): 0.020740 deg/deg.s-2
Yaw (medium cilia): 0.022544 deg/deg.s-2
Yaw (short cilia): 0.024500 deg/deg.s-2

Global mean (long cilia): 0.022780 deg/deg.s-2
Global mean (medium cilia): 0.024678 deg/deg.s-2
Global mean (short cilia): 0.026736 deg/deg.s-2

Global standard deviation (long cilia): 0.001955 deg/deg.s-2
Global standard deviation (medium cilia): 0.002079 deg/deg.s-2
Global standard deviation (short cilia): 0.002214 deg/deg.s-2

Global maximum (long cilia): 0.026534 deg/deg.s-2
Global maximum (medium cilia): 0.028681 deg/deg.s-2
Global maximum (short cilia): 0.031009 deg/deg.s-2

Global minimum (long cilia): 0.019370 deg/deg.s-2
Global minimum (medium cilia): 0.021029 deg/deg.s-2
Global minimum (short cilia): 0.022828 deg/deg.s-2

Macaca mulatta

ENDOLYMPH VOLUMES

Anterior slender duct: 0.573168 mm^3
Posterior slender duct: 0.616183 mm^3
Lateral slender duct: 0.593940 mm^3

Common crus: 0.583482 mm^3
Simple crus: 0.293619 mm^3

Anterior utricle: 3.540135 mm^3
Posterior utricle: 1.196645 mm^3

Anterior ampulla: 1.154027 mm^3
Posterior ampulla: 1.070914 mm^3
Lateral ampulla: 1.026399 mm^3

Anterior duct: 5.850812 mm^3
Posterior duct: 3.467224 mm^3
Lateral duct: 5.454094 mm^3

Total SCDS volume: 9.839311 mm^3
Smoothing effect: -0.608460 %
Division error: 1.975531 %

STREAMLINE LENGTHS

Anterior slender duct: 11.200181 mm
Posterior slender duct: 12.650838 mm
Lateral slender duct: 12.231592 mm

Common crus (anterior trajectory): 2.697941 mm
Common crus (posterior trajectory): 2.776912 mm
Common crus (common part): 2.918817 mm
Simple crus: 0.780659 mm

Anterior utricle (anterior trajectory): 3.629982 mm
Anterior utricle (lateral trajectory): 3.684781 mm
Anterior utricle (common part): 2.895387 mm
Posterior utricle: 1.816206 mm

Anterior ampulla: 1.678271 mm
Posterior ampulla: 1.598474 mm
Lateral ampulla: 1.610875 mm

Anterior duct: 19.206375 mm
Posterior duct: 18.842430 mm
Lateral duct: 18.307907 mm

STREAMLINE ENCLOSED AREAS

Anterior duct: 24.297770 mm^2
Posterior duct: 24.958677 mm^2
Lateral duct: 23.150373 mm^2

RADII OF CURVATURE

Anterior duct: 2.918918 mm
Posterior duct: 2.908741 mm
Lateral duct: 2.814191 mm

CROSS-SECTIONAL AREAS

Anterior slender duct: 0.051175 mm^2
Posterior slender duct: 0.048707 mm^2
Lateral slender duct: 0.048558 mm^2

Common crus (anterior trajectory): 0.216269 mm^2
Common crus (posterior trajectory): 0.210120 mm^2
Common crus (common part): 0.216269 mm^2
Simple crus: 0.376117 mm^2

Anterior utricle (anterior trajectory): 0.975249 mm^2
Anterior utricle (lateral trajectory): 0.960746 mm^2
Anterior utricle (common part): 0.975249 mm^2
Posterior utricle: 0.658870 mm^2

Anterior ampulla: 0.687628 mm^2
Posterior ampulla: 0.669960 mm^2
Lateral ampulla: 0.637169 mm^2

Anterior duct: 0.304629 mm^2
Posterior duct: 0.184012 mm^2
Lateral duct: 0.297909 mm^2

SLENDER DUCT CROSS-SECTIONAL RADII

Anterior slender duct: 0.132946 mm
Posterior slender duct: 0.128519 mm
Lateral slender duct: 0.127674 mm

CROSS-SECTIONAL RATIOS

Common crus to anterior slender duct: 2.066313
Common crus to posterior slender duct: 2.095747

Anterior utricle to anterior slender duct: 4.580746
Anterior utricle to lateral slender duct: 4.702522
Posterior utricle to posterior slender duct: 3.864728

Anterior ampulla to anterior slender duct: 3.687035
Posterior ampulla to posterior slender duct: 3.707601
Lateral ampulla to lateral slender duct: 3.814217

WALL SHAPE DRAG FACTORS

Anterior slender duct: 29.494623
Posterior slender duct: 28.469766
Lateral slender duct: 27.914956

Common crus (anterior trajectory): 30.080688
Common crus (posterior trajectory): 29.474932
Common crus (common part): 29.777810
Simple crus: 29.587668

Anterior utricle (anterior trajectory): 35.354617
Anterior utricle (lateral trajectory): 34.485259
Anterior utricle (common part): 34.919938
Posterior utricle: 34.354671

Anterior ampulla: 30.160825
Posterior ampulla: 31.696439
Lateral ampulla: 30.532517

Anterior duct: 30.742693
Posterior duct: 29.458875
Lateral duct: 29.538981

CUPULA MORPHOMETRY

CUPULA CROSS-SECTIONAL AREAS

Anterior cupula (average): 0.862511 mm^2
Posterior cupula (average): 0.838372 mm^2
Lateral cupula (average): 0.784392 mm^2

Anterior cupula (central section): 0.751719 mm^2
Posterior cupula (central section): 0.728021 mm^2
Lateral cupula (central section): 0.675604 mm^2

CUPULA THICKNESS

Anterior cupula (long cilia): 0.421012 mm
Anterior cupula (medium cilia): 0.378592 mm
Anterior cupula (short cilia): 0.336172 mm

Posterior cupula (long cilia): 0.421512 mm
Posterior cupula (medium cilia): 0.379092 mm
Posterior cupula (short cilia): 0.336672 mm

Lateral cupula (long cilia): 0.441360 mm
Lateral cupula (medium cilia): 0.398940 mm
Lateral cupula (short cilia): 0.356520 mm

Anterior cupula (Rabbitt's empirical estimation): 0.342414 mm
Posterior cupula (Rabbitt's empirical estimation): 0.336973 mm
Lateral cupula (Rabbitt's empirical estimation): 0.324616 mm

SEMICIRCULAR DUCT PLANES (UNCOUPLED)

Right Anterior duct: 0.648091x 0.754418y 0.104079z
Right Posterior duct: 0.744888x -0.666971y 0.017110z
Right Lateral duct: 0.000000x 0.090375y -0.995908z
Left Anterior duct: -0.648091x 0.754418y -0.104079z
Left Posterior duct: -0.744888x -0.666971y -0.017110z
Left Lateral duct: -0.000000x 0.090375y 0.995908z

SEMICIRCULAR DUCT CENTER OF ROTATION (UNCOUPLED)

Right Anterior duct: 8357.595331x -36183.144372y -12157.289962z
Right Posterior duct: 4610.862095x -36306.836812y -14300.062233z
Right Lateral duct: 6808.375944x -37618.866657y -14594.391184z

Left Anterior duct: 8357.595331x -6036.370096y -12157.289962z
Left Posterior duct: 4610.862095x -5912.677657y -14300.062233z
Left Lateral duct: 6808.375944x -4600.647811y -14594.391184z

IPSILATERAL ANGLES (UNCOUPLED)

Anterior and Posterior ducts: 91.074674 Degrees
Anterior and Lateral ducts: 87.973581 Degrees
Lateral and Posterior ducts: 94.441256 Degrees

SYNERGISTIC ANGLES (UNCOUPLED)

Anterior and Posterior ducts: 171.020463 Degrees
Lateral ducts: 169.642334 Degrees

REFERENCE ANGLES (UNCOUPLED)

VESTIBULAR PITCH / MID-SAGITTAL PLANE

Anterior duct: 41.028509 Degrees
Posterior duct: 48.169868 Degrees
Lateral duct: 95.192189 Degrees

VESTIBULAR ROLL / CORONAL PLANE

Anterior duct: 49.605864 Degrees
Posterior duct: 41.853628 Degrees
Lateral duct: 90.000000 Degrees

VESTIBULAR YAW / HORIZONTAL PLANE

Anterior duct: 95.981199 Degrees
Posterior duct: 89.026195 Degrees
Lateral duct: 5.185365 Degrees

DEVIATION FROM IDEAL CONFIGURATION (UNCOUPLED)

Variance from orthogonality: 8.328684 Degrees^2

Average deviation from orthogonality: 2.514116 Degrees

Variance from planarity: 89.515138 Degrees^2

Average deviation from planarity: 9.438913 Degrees

SHRINKAGE CORRECTION

Factor slender duct cross-sectional diameter: 16.000000
Factor slender duct cross-sectional perimeter: 15.000000
Factor ampulla cross-sectional diameter: 14.000000
Factor ampulla cross-sectional perimeter: 18.000000
Factor utricle cross-sectional diameter: 31.000000
Factor utricle cross-sectional perimeter: 9.000000
Factor crus cross-sectional diameter: 24.000000
Factor crus cross-sectional perimeter: 12.000000

Shrinkage Correction Strength: 0.000000

CUPULA FINITE ELEMENT ANALYSIS

DEFORMATION PROFILE FACTORS

Anterior cupula (long cilia): 16.832474
Anterior cupula (medium cilia): 15.744043
Anterior cupula (short cilia): 14.458987

Posterior cupula (long cilia): 17.034148
Posterior cupula (medium cilia): 15.968040
Posterior cupula (short cilia): 14.701544

Lateral cupula (long cilia): 17.823371
Lateral cupula (medium cilia): 16.849414
Lateral cupula (short cilia): 15.690934

DEFLECTION CILIA AREAS

Anterior cupula (long cilia, high thickness): 0.476548 deg/nL
Anterior cupula (medium cilia, high thickness): 0.473361 deg/nL
Anterior cupula (medium cilia, medium thickness): 0.453902 deg/nL
Anterior cupula (short cilia, high thickness): 0.461266 deg/nL
Anterior cupula (short cilia, medium thickness): 0.439246 deg/nL
Anterior cupula (short cilia, low thickness): 0.412137 deg/nL

Posterior cupula (long cilia, high thickness): 0.498549 deg/nL
Posterior cupula (medium cilia, high thickness): 0.493105 deg/nL
Posterior cupula (medium cilia, medium thickness): 0.473081 deg/nL
Posterior cupula (short cilia, high thickness): 0.486910 deg/nL
Posterior cupula (short cilia, medium thickness): 0.463837 deg/nL
Posterior cupula (short cilia, low thickness): 0.435400 deg/nL

Lateral cupula (long cilia, high thickness): 0.570069 deg/nL
Lateral cupula (medium cilia, high thickness): 0.565223 deg/nL
Lateral cupula (medium cilia, medium thickness): 0.545758 deg/nL
Lateral cupula (short cilia, high thickness): 0.557331 deg/nL
Lateral cupula (short cilia, medium thickness): 0.534940 deg/nL
Lateral cupula (short cilia, low thickness): 0.507198 deg/nL

STIFFNESS

Anterior cupula (long cilia): 18.175341 g/s^2.mm^4
Anterior cupula (medium cilia): 15.287196 g/s^2.mm^4
Anterior cupula (short cilia): 12.466356 g/s^2.mm^4

Posterior cupula (long cilia): 19.642811 g/s^2.mm^4
Posterior cupula (medium cilia): 16.560351 g/s^2.mm^4
Posterior cupula (short cilia): 13.540766 g/s^2.mm^4

Lateral cupula (long cilia): 25.256297 g/s^2.mm^4
Lateral cupula (medium cilia): 21.581383 g/s^2.mm^4
Lateral cupula (short cilia): 17.960548 g/s^2.mm^4

STRUCTURAL PARAMETERS

MASS

Common crus (common part): 0.013496 g/mm^4
Anterior utricle (common part): 0.002969 g/mm^4

Anterior duct (long cilia): 0.237374 g/mm^4
Anterior duct (medium cilia): 0.237386 g/mm^4
Anterior duct (short cilia): 0.237399 g/mm^4

Posterior duct (long cilia): 0.277965 g/mm^4
Posterior duct (medium cilia): 0.277978 g/mm^4
Posterior duct (short cilia): 0.277991 g/mm^4

Lateral duct (long cilia): 0.260206 g/mm^4
Lateral duct (medium cilia): 0.260218 g/mm^4
Lateral duct (short cilia): 0.260231 g/mm^4

DAMPING

Common crus (common part): 1.505756 g/s.mm^4
Anterior utricle (common part): 0.086138 g/s.mm^4

Anterior duct (long cilia): 103.868048 g/s.mm^4
Anterior duct (medium cilia): 103.857975 g/s.mm^4
Anterior duct (short cilia): 103.848187 g/s.mm^4

Posterior duct (long cilia): 124.785469 g/s.mm^4
Posterior duct (medium cilia): 124.774907 g/s.mm^4
Posterior duct (short cilia): 124.764610 g/s.mm^4

Lateral duct (long cilia): 117.757614 g/s.mm^4
Lateral duct (medium cilia): 117.745125 g/s.mm^4
Lateral duct (short cilia): 117.732858 g/s.mm^4

INERTIAL FORCING

Anterior duct: 0.048596 g/mm
Posterior duct: 0.049917 g/mm
Lateral duct: 0.046301 g/mm

DYNAMIC PARAMETERS

TIME CONSTANTS

LONG TIME CONSTANTS

First constant (long cilia): 5.702727 s
First constant (medium cilia): 6.779643 s
First constant (short cilia): 8.313086 s

Second constant (long cilia): 6.360284 s
Second constant (medium cilia): 7.544206 s
Second constant (short cilia): 9.226695 s

Third constant (long cilia): 4.660279 s
Third constant (medium cilia): 5.453638 s
Third constant (short cilia): 6.552852 s

SHORT TIME CONSTANTS

First constant (long cilia): 2.212219 ms
First constant (medium cilia): 2.212405 ms
First constant (short cilia): 2.212590 ms

Second constant (long cilia): 2.352393 ms
Second constant (medium cilia): 2.352575 ms
Second constant (short cilia): 2.352755 ms

Third constant (long cilia): 2.158305 ms
Third constant (medium cilia): 2.158489 ms
Third constant (short cilia): 2.158671 ms

BANDWIDTH

LOWER CORNER FREQUENCIES

First frequency (long cilia): 0.027909 Hz
First frequency (medium cilia): 0.023475 Hz
First frequency (short cilia): 0.019145 Hz

Second frequency (long cilia): 0.025023 Hz
Second frequency (medium cilia): 0.021096 Hz
Second frequency (short cilia): 0.017249 Hz

Third frequency (long cilia): 0.034151 Hz
Third frequency (medium cilia): 0.029183 Hz
Third frequency (short cilia): 0.024288 Hz

UPPER CORNER FREQUENCIES

First frequency (long cilia): 71.943578 Hz
First frequency (medium cilia): 71.937518 Hz
First frequency (short cilia): 71.931524 Hz

Second frequency (long cilia): 67.656620 Hz
Second frequency (medium cilia): 67.651373 Hz
Second frequency (short cilia): 67.646202 Hz

Third frequency (long cilia): 73.740697 Hz
Third frequency (medium cilia): 73.734407 Hz
Third frequency (short cilia): 73.728203 Hz

IN-PLANE SENSITIVITY PARAMETERS

MECHANICAL GAIN

Anterior duct (cilia average deflection, long cilia): 1.688814 deg/deg
Anterior duct (cilia average deflection, medium cilia): 1.603219 deg/deg
Anterior duct (cilia average deflection, short cilia): 1.479509 deg/deg

Posterior duct (cilia average deflection, long cilia): 1.553060 deg/deg
Posterior duct (cilia average deflection, medium cilia): 1.476119 deg/deg
Posterior duct (cilia average deflection, short cilia): 1.371885 deg/deg

Lateral duct (cilia average deflection, long cilia): 1.751890 deg/deg
Lateral duct (cilia average deflection, medium cilia): 1.677729 deg/deg
Lateral duct (cilia average deflection, short cilia): 1.574723 deg/deg

VELOCITY GAIN

Anterior duct (cilia average deflection, long cilia): 0.003843 deg/deg.s-1
Anterior duct (cilia average deflection, medium cilia): 0.003649 deg/deg.s-1
Anterior duct (cilia average deflection, short cilia): 0.003368 deg/deg.s-1

Posterior duct (cilia average deflection, long cilia): 0.003443 deg/deg.s-1
Posterior duct (cilia average deflection, medium cilia): 0.003273 deg/deg.s-1
Posterior duct (cilia average deflection, short cilia): 0.003042 deg/deg.s-1

Lateral duct (cilia average deflection, long cilia): 0.003872 deg/deg.s-1
Lateral duct (cilia average deflection, medium cilia): 0.003708 deg/deg.s-1
Lateral duct (cilia average deflection, short cilia): 0.003481 deg/deg.s-1

ACCELERATION GAIN

Anterior duct (cilia average deflection, long cilia): 0.021951 deg/deg.s-2
Anterior duct (cilia average deflection, medium cilia): 0.024776 deg/deg.s-2
Anterior duct (cilia average deflection, short cilia): 0.028040 deg/deg.s-2

Posterior duct (cilia average deflection, long cilia): 0.021860 deg/deg.s-2
Posterior duct (cilia average deflection, medium cilia): 0.024645 deg/deg.s-2
Posterior duct (cilia average deflection, short cilia): 0.028014 deg/deg.s-2

Lateral duct (cilia average deflection, long cilia): 0.018052 deg/deg.s-2
Lateral duct (cilia average deflection, medium cilia): 0.020233 deg/deg.s-2
Lateral duct (cilia average deflection, short cilia): 0.022820 deg/deg.s-2

FUNCTIONAL PLANES

MAXIMAL RESPONSE PLANES

Right Anterior duct: 0.638733x 0.762532y 0.102787z
Right Posterior duct: 0.735482x -0.677373y 0.015234z
Right Lateral duct: 0.000000x 0.091042y -0.995847z
Left Anterior duct: -0.638733x 0.762532y -0.102787z
Left Posterior duct: -0.735482x -0.677373y -0.015234z
Left Lateral duct: -0.000000x 0.091042y 0.995847z

PRIME DIRECTIONS

Right Anterior duct: 0.673173x 0.732427y 0.066959z
Right Posterior duct: 0.768723x -0.636081y -0.058151z
Right Lateral duct: 0.081242x 0.065867y -0.993489z
Left Anterior duct: -0.673173x 0.732427y -0.066959z
Left Posterior duct: -0.768723x -0.636081y 0.058151z
Left Lateral duct: -0.081242x 0.065867y 0.993489z

IPSILATERAL ANGLES

Anterior and Posterior ducts: 92.596123 Degrees
Anterior and Lateral ducts: 88.118964 Degrees
Lateral and Posterior ducts: 94.413921 Degrees

SYNERGISTIC ANGLES

Anterior and Posterior ducts: 171.076130 Degrees
Lateral ducts: 169.565412 Degrees

REFERENCE ANGLES

VESTIBULAR PITCH / MID-SAGITTAL PLANE

Anterior duct: 40.315057 Degrees

Posterior duct: 47.364755 Degrees
Lateral duct: 95.230553 Degrees

VESTIBULAR ROLL / CORONAL PLANE

Anterior duct: 50.306285 Degrees
Posterior duct: 42.655218 Degrees
Lateral duct: 90.000000 Degrees

VESTIBULAR YAW / HORIZONTAL PLANE

Anterior duct: 95.906725 Degrees
Posterior duct: 89.133670 Degrees
Lateral duct: 5.223923 Degrees

DEVIATION FROM IDEAL CONFIGURATION

Variance from orthogonality: 9.920283 Degrees^2

Average deviation from orthogonality: 2.963693 Degrees

Variance from planarity: 89.383839 Degrees^2

Average deviation from planarity: 9.427442 Degrees

SEMICIRCULAR DUCT CENTER OF ROTATION

Right Anterior duct: 10479.701240x -33641.625673y -11819.368278z
Right Posterior duct: 7054.234156x -38564.522492y -14251.859477z
Right Lateral duct: 6800.223199x -37315.424650y -17917.350038z
Left Anterior duct: 6221.905930x -3494.851397y -12504.544106z
Left Posterior duct: 2151.514024x -8170.363337y -14353.411313z
Left Lateral duct: 6800.223199x -4297.205804y -11279.033400z

MATERIAL PROPERTIES

Body temperature: 37.300000 C

Endolymph density: 1000.000000 kg/m^3
Endolymph viscosity: 0.000810 Pa.s
Endolymph viscosity factor: 2.850000 e-5 Pa.s

Cupula density: 1000.000000 kg/m^3
Cupula viscosity: 0.008103 Pa.s
Cupula Poisson ratio: 0.480000
Cupula Young modulus: 4.258000 Pa
Cupula Shear modulus: 1.438514 Pa

SATURATING MECHANICAL GAIN OF THE SEMICIRCULAR DUCT SYSTEM TO ROTATIONS IN SPACE

- MAXIMUM VALUE AMONG THE ABSOLUTE MECHANICAL GAINS OF THE SIX SEMICIRCULAR DUCTS, WEIGHTED ACCORDING TO INHIBITION/ACTIVATION
-- TO ANGULAR VELOCITY
--- MEASURED IN UNITS OF CILIA AVERAGE DEFLECTION

Nose-down Pitch (long cilia): 0.002332 deg/deg.s-1
Nose-down Pitch (medium cilia): 0.002217 deg/deg.s-1

Nose-down Pitch (short cilia): 0.002061 deg/deg.s-1

Nose-up Pitch (long cilia): 0.002930 deg/deg.s-1
Nose-up Pitch (medium cilia): 0.002782 deg/deg.s-1
Nose-up Pitch (short cilia): 0.002568 deg/deg.s-1

Roll (long cilia): 0.002532 deg/deg.s-1
Roll (medium cilia): 0.002407 deg/deg.s-1
Roll (short cilia): 0.002237 deg/deg.s-1

Yaw (long cilia): 0.003856 deg/deg.s-1
Yaw (medium cilia): 0.003693 deg/deg.s-1
Yaw (short cilia): 0.003467 deg/deg.s-1

Global mean (long cilia): 0.003219 deg/deg.s-1
Global mean (medium cilia): 0.003072 deg/deg.s-1
Global mean (short cilia): 0.002868 deg/deg.s-1

Global standard deviation (long cilia): 0.000478 deg/deg.s-1
Global standard deviation (medium cilia): 0.000462 deg/deg.s-1
Global standard deviation (short cilia): 0.000440 deg/deg.s-1

Global maximum (long cilia): 0.003872 deg/deg.s-1
Global maximum (medium cilia): 0.003708 deg/deg.s-1
Global maximum (short cilia): 0.003481 deg/deg.s-1

Global minimum (long cilia): 0.001923 deg/deg.s-1
Global minimum (medium cilia): 0.001841 deg/deg.s-1
Global minimum (short cilia): 0.001714 deg/deg.s-1

SATURATING MECHANICAL GAIN OF THE SEMICIRCULAR DUCT SYSTEM TO ROTATIONS IN SPACE

- MAXIMUM VALUE AMONG THE ABSOLUTE MECHANICAL GAINS OF THE SIX SEMICIRCULAR DUCTS, WEIGHTED ACCORDING TO INHIBITION/ACTIVATION
-- TO ANGULAR ACCELERATION
--- MEASURED IN UNITS OF CILIA AVERAGE DEFLECTION

Nose-down Pitch (long cilia): 0.014807 deg/deg.s-1
Nose-down Pitch (medium cilia): 0.016694 deg/deg.s-1
Nose-down Pitch (short cilia): 0.018976 deg/deg.s-1

Nose-up Pitch (long cilia): 0.016738 deg/deg.s-1
Nose-up Pitch (medium cilia): 0.018892 deg/deg.s-1
Nose-up Pitch (short cilia): 0.021381 deg/deg.s-1

Roll (long cilia): 0.016078 deg/deg.s-1
Roll (medium cilia): 0.018126 deg/deg.s-1
Roll (short cilia): 0.020604 deg/deg.s-1

Yaw (long cilia): 0.017977 deg/deg.s-1
Yaw (medium cilia): 0.020149 deg/deg.s-1
Yaw (short cilia): 0.022725 deg/deg.s-1

Global mean (long cilia): 0.016995 deg/deg.s-1
Global mean (medium cilia): 0.019116 deg/deg.s-1
Global mean (short cilia): 0.021628 deg/deg.s-1

Global standard deviation (long cilia): 0.002225 deg/deg.s-1
Global standard deviation (medium cilia): 0.002522 deg/deg.s-1

Global standard deviation (short cilia): 0.002872 deg/deg.s-1

Global maximum (long cilia): 0.021951 deg/deg.s-1
Global maximum (medium cilia): 0.024776 deg/deg.s-1
Global maximum (short cilia): 0.028040 deg/deg.s-1

Global minimum (long cilia): 0.011017 deg/deg.s-1
Global minimum (medium cilia): 0.012395 deg/deg.s-1
Global minimum (short cilia): 0.013979 deg/deg.s-1

STANDARDIZED MECHANICAL GAIN OF THE SEMICIRCULAR DUCT SYSTEM TO ROTATIONS IN SPACE

- OF THE THREE SYNERGISTIC SEMICIRCULAR DUCT PAIRS
-- TO ANGULAR VELOCITY
--- MEASURED IN UNITS OF CILIA AVERAGE DEFLECTION

Pitch (long cilia): 0.002481 deg/deg.s-1
Pitch (medium cilia): 0.002357 deg/deg.s-1
Pitch (short cilia): 0.002182 deg/deg.s-1

Roll (long cilia): 0.002351 deg/deg.s-1
Roll (medium cilia): 0.002234 deg/deg.s-1
Roll (short cilia): 0.002069 deg/deg.s-1

Yaw (long cilia): 0.002579 deg/deg.s-1
Yaw (medium cilia): 0.002470 deg/deg.s-1
Yaw (short cilia): 0.002318 deg/deg.s-1

Global mean (long cilia): 0.002498 deg/deg.s-1
Global mean (medium cilia): 0.002383 deg/deg.s-1
Global mean (short cilia): 0.002222 deg/deg.s-1

Global standard deviation (long cilia): 0.000097 deg/deg.s-1
Global standard deviation (medium cilia): 0.000097 deg/deg.s-1
Global standard deviation (short cilia): 0.000099 deg/deg.s-1

Global maximum (long cilia): 0.002615 deg/deg.s-1
Global maximum (medium cilia): 0.002502 deg/deg.s-1
Global maximum (short cilia): 0.002344 deg/deg.s-1

Global minimum (long cilia): 0.002311 deg/deg.s-1
Global minimum (medium cilia): 0.002198 deg/deg.s-1
Global minimum (short cilia): 0.002039 deg/deg.s-1

STANDARDIZED MECHANICAL GAIN OF THE SEMICIRCULAR DUCT SYSTEM TO ROTATIONS IN SPACE

- OF THE THREE SYNERGISTIC SEMICIRCULAR DUCT PAIRS
-- TO ANGULAR ACCELERATION
--- MEASURED IN UNITS OF CILIA AVERAGE DEFLECTION

Pitch (long cilia): 0.014871 deg/deg.s-2
Pitch (medium cilia): 0.016776 deg/deg.s-2
Pitch (short cilia): 0.019025 deg/deg.s-2

Roll (long cilia): 0.014189 deg/deg.s-2
Roll (medium cilia): 0.016005 deg/deg.s-2
Roll (short cilia): 0.018156 deg/deg.s-2

Yaw (long cilia): 0.012047 deg/deg.s-2
Yaw (medium cilia): 0.013503 deg/deg.s-2
Yaw (short cilia): 0.015230 deg/deg.s-2

Global mean (long cilia): 0.013322 deg/deg.s-2
Global mean (medium cilia): 0.014987 deg/deg.s-2
Global mean (short cilia): 0.016959 deg/deg.s-2

Global standard deviation (long cilia): 0.000975 deg/deg.s-2
Global standard deviation (medium cilia): 0.001127 deg/deg.s-2
Global standard deviation (short cilia): 0.001306 deg/deg.s-2

Global maximum (long cilia): 0.014871 deg/deg.s-2
Global maximum (medium cilia): 0.016776 deg/deg.s-2
Global maximum (short cilia): 0.019025 deg/deg.s-2

Global minimum (long cilia): 0.011841 deg/deg.s-2
Global minimum (medium cilia): 0.013276 deg/deg.s-2
Global minimum (short cilia): 0.014978 deg/deg.s-2

Saimiri sciureus

ENDOLYMPH VOLUMES

Anterior slender duct: 0.298077 mm^3
Posterior slender duct: 0.331530 mm^3
Lateral slender duct: 0.324779 mm^3

Common crus: 0.279173 mm^3
Simple crus: 0.106680 mm^3

Anterior utricle: 1.231248 mm^3
Posterior utricle: 0.341831 mm^3

Anterior ampulla: 0.783920 mm^3
Posterior ampulla: 0.763061 mm^3
Lateral ampulla: 0.707747 mm^3

Anterior duct: 2.592419 mm^3
Posterior duct: 1.715594 mm^3
Lateral duct: 2.370455 mm^3

Total SCDS volume: 4.930984 mm^3
Smoothing effect: -0.635023 %
Division error: 3.529205 %

STREAMLINE LENGTHS

Anterior slender duct: 8.891746 mm
Posterior slender duct: 8.677226 mm
Lateral slender duct: 9.587321 mm

Common crus (anterior trajectory): 2.463365 mm
Common crus (posterior trajectory): 2.489668 mm
Common crus (common part): 2.606881 mm
Simple crus: 0.738762 mm

Anterior utricle (anterior trajectory): 2.571090 mm
Anterior utricle (lateral trajectory): 2.582754 mm
Anterior utricle (common part): 1.733996 mm
Posterior utricle: 1.224518 mm

Anterior ampulla: 1.606355 mm
Posterior ampulla: 1.646668 mm
Lateral ampulla: 1.556655 mm

Anterior duct: 15.532556 mm
Posterior duct: 14.038079 mm
Lateral duct: 14.465493 mm

STREAMLINE ENCLOSED AREAS

Anterior duct: 16.074441 mm^2
Posterior duct: 12.791368 mm^2
Lateral duct: 13.680950 mm^2

RADII OF CURVATURE

Anterior duct: 2.367043 mm
Posterior duct: 2.126027 mm
Lateral duct: 2.194533 mm

CROSS-SECTIONAL AREAS

Anterior slender duct: 0.033523 mm^2
Posterior slender duct: 0.038207 mm^2
Lateral slender duct: 0.033876 mm^2

Common crus (anterior trajectory): 0.113330 mm^2
Common crus (posterior trajectory): 0.112133 mm^2
Common crus (common part): 0.113330 mm^2
Simple crus: 0.144404 mm^2

Anterior utricle (anterior trajectory): 0.478882 mm^2
Anterior utricle (lateral trajectory): 0.476720 mm^2
Anterior utricle (common part): 0.478882 mm^2
Posterior utricle: 0.279155 mm^2

Anterior ampulla: 0.488012 mm^2
Posterior ampulla: 0.463397 mm^2
Lateral ampulla: 0.454659 mm^2

Anterior duct: 0.166902 mm^2
Posterior duct: 0.122210 mm^2
Lateral duct: 0.163870 mm^2

SLENDER DUCT CROSS-SECTIONAL RADII

Anterior slender duct: 0.105711 mm
Posterior slender duct: 0.112705 mm
Lateral slender duct: 0.106414 mm

CROSS-SECTIONAL RATIOS

Common crus to anterior slender duct: 1.869877
Common crus to posterior slender duct: 1.732924

Anterior utricle to anterior slender duct: 4.175842
Anterior utricle to lateral slender duct: 4.094533
Posterior utricle to posterior slender duct: 2.871029

Anterior ampulla to anterior slender duct: 3.963033
Posterior ampulla to posterior slender duct: 3.822687
Lateral ampulla to lateral slender duct: 3.643033

WALL SHAPE DRAG FACTORS

Anterior slender duct: 27.535097
Posterior slender duct: 27.392538
Lateral slender duct: 27.684813

Common crus (anterior trajectory): 29.393829
Common crus (posterior trajectory): 28.644630
Common crus (common part): 29.019230
Simple crus: 32.104237

Anterior utricle (anterior trajectory): 39.982259
Anterior utricle (lateral trajectory): 38.460454
Anterior utricle (common part): 39.221357
Posterior utricle: 34.464340

Anterior ampulla: 31.858272
Posterior ampulla: 32.556036
Lateral ampulla: 32.582131

Anterior duct: 30.337344
Posterior duct: 28.837138
Lateral duct: 30.361471

CUPULA MORPHOMETRY

CUPULA CROSS-SECTIONAL AREAS

Anterior cupula (average): 0.619417 mm^2
Posterior cupula (average): 0.578723 mm^2
Lateral cupula (average): 0.561797 mm^2

Anterior cupula (central section): 0.543270 mm^2
Posterior cupula (central section): 0.516870 mm^2
Lateral cupula (central section): 0.496768 mm^2

CUPULA THICKNESS

Anterior cupula (long cilia): 0.352550 mm
Anterior cupula (medium cilia): 0.310130 mm
Anterior cupula (short cilia): 0.267710 mm

Posterior cupula (long cilia): 0.422726 mm
Posterior cupula (medium cilia): 0.380306 mm
Posterior cupula (short cilia): 0.337886 mm

Lateral cupula (long cilia): 0.383971 mm
Lateral cupula (medium cilia): 0.341551 mm
Lateral cupula (short cilia): 0.299131 mm

Anterior cupula (Rabbitt's empirical estimation): 0.291092 mm
Posterior cupula (Rabbitt's empirical estimation): 0.283932 mm
Lateral cupula (Rabbitt's empirical estimation): 0.278356 mm

SEMICIRCULAR DUCT PLANES (UNCOUPLED)

Right Anterior duct: 0.650990x 0.759038y 0.008613z
Right Posterior duct: 0.642541x -0.756619y 0.121117z
Right Lateral duct: -0.000000x -0.142152y -0.989845z
Left Anterior duct: -0.650990x 0.759038y -0.008613z
Left Posterior duct: -0.642541x -0.756619y -0.121117z
Left Lateral duct: 0.000000x -0.142152y 0.989845z

SEMICIRCULAR DUCT CENTER OF ROTATION (UNCOUPLED)

Right Anterior duct: 1773.106402x -5251.507198y 12254.709170z
Right Posterior duct: -674.537412x -5085.816371y 10276.724143z
Right Lateral duct: 620.767562x -6534.684971y 10295.227754z

Left Anterior duct: 1773.106402x 14581.127227y 12254.709170z
Left Posterior duct: -674.537412x 14415.436400y 10276.724143z
Left Lateral duct: 620.767562x 15864.305000y 10295.227754z

IPSILATERAL ANGLES (UNCOUPLED)

Anterior and Posterior ducts: 98.922438 Degrees
Anterior and Lateral ducts: 83.320334 Degrees
Lateral and Posterior ducts: 90.713247 Degrees

SYNERGISTIC ANGLES (UNCOUPLED)

Anterior and Posterior ducts: 173.544066 Degrees
Lateral ducts: 163.667236 Degrees

REFERENCE ANGLES (UNCOUPLED)

VESTIBULAR PITCH / MID-SAGITTAL PLANE

Anterior duct: 40.623566 Degrees
Posterior duct: 40.835943 Degrees
Lateral duct: 81.833623 Degrees

VESTIBULAR ROLL / CORONAL PLANE

Anterior duct: 49.387364 Degrees
Posterior duct: 50.022110 Degrees
Lateral duct: 90.000000 Degrees

VESTIBULAR YAW / HORIZONTAL PLANE

Anterior duct: 90.500177 Degrees
Posterior duct: 83.049563 Degrees
Lateral duct: 8.173016 Degrees

DEVIATION FROM IDEAL CONFIGURATION (UNCOUPLED)

Variance from orthogonality: 41.578857 Degrees^2

Average deviation from orthogonality: 5.438450 Degrees

Variance from planarity: 116.705777 Degrees^2

Average deviation from planarity: 9.748210 Degrees

SHRINKAGE CORRECTION

Factor slender duct cross-sectional diameter: 16.000000
Factor slender duct cross-sectional perimeter: 15.000000
Factor ampulla cross-sectional diameter: 14.000000
Factor ampulla cross-sectional perimeter: 18.000000
Factor utricle cross-sectional diameter: 31.000000
Factor utricle cross-sectional perimeter: 9.000000
Factor crus cross-sectional diameter: 24.000000
Factor crus cross-sectional perimeter: 12.000000

Shrinkage Correction Strength: 0.000000

CUPULA FINITE ELEMENT ANALYSIS

DEFORMATION PROFILE FACTORS

Anterior cupula (long cilia): 16.484052
Anterior cupula (medium cilia): 15.146731
Anterior cupula (short cilia): 13.525886

Posterior cupula (long cilia): 18.427385
Posterior cupula (medium cilia): 17.468551
Posterior cupula (short cilia): 16.311102

Lateral cupula (long cilia): 17.907911
Lateral cupula (medium cilia): 16.797054
Lateral cupula (short cilia): 15.440627

DEFLECTION CILIA AREAS

Anterior cupula (long cilia, high thickness): 0.767557 deg/nL
Anterior cupula (medium cilia, high thickness): 0.763079 deg/nL
Anterior cupula (medium cilia, medium thickness): 0.725762 deg/nL
Anterior cupula (short cilia, high thickness): 0.759028 deg/nL
Anterior cupula (short cilia, medium thickness): 0.714506 deg/nL
Anterior cupula (short cilia, low thickness): 0.657699 deg/nL

Posterior cupula (long cilia, high thickness): 0.885006 deg/nL
Posterior cupula (medium cilia, high thickness): 0.891705 deg/nL
Posterior cupula (medium cilia, medium thickness): 0.865364 deg/nL
Posterior cupula (short cilia, high thickness): 0.891121 deg/nL
Posterior cupula (short cilia, medium thickness): 0.859715 deg/nL
Posterior cupula (short cilia, low thickness): 0.819808 deg/nL

Lateral cupula (long cilia, high thickness): 0.940703 deg/nL
Lateral cupula (medium cilia, high thickness): 0.947028 deg/nL
Lateral cupula (medium cilia, medium thickness): 0.913242 deg/nL
Lateral cupula (short cilia, high thickness): 0.942585 deg/nL
Lateral cupula (short cilia, medium thickness): 0.902038 deg/nL
Lateral cupula (short cilia, low thickness): 0.849874 deg/nL

STIFFNESS

Anterior cupula (long cilia): 28.324722 g/s^2.mm^4
Anterior cupula (medium cilia): 22.895159 g/s^2.mm^4
Anterior cupula (short cilia): 17.648642 g/s^2.mm^4

Posterior cupula (long cilia): 41.944177 g/s^2.mm^4
Posterior cupula (medium cilia): 35.771657 g/s^2.mm^4
Posterior cupula (short cilia): 29.675806 g/s^2.mm^4

Lateral cupula (long cilia): 40.081857 g/s^2.mm^4
Lateral cupula (medium cilia): 33.442070 g/s^2.mm^4
Lateral cupula (short cilia): 26.923458 g/s^2.mm^4

STRUCTURAL PARAMETERS

MASS

Common crus (common part): 0.023003 g/mm^4
Anterior utricle (common part): 0.003621 g/mm^4

Anterior duct (long cilia): 0.295487 g/mm^4
Anterior duct (medium cilia): 0.295505 g/mm^4
Anterior duct (short cilia): 0.295524 g/mm^4

Posterior duct (long cilia): 0.257072 g/mm^4
Posterior duct (medium cilia): 0.257090 g/mm^4
Posterior duct (short cilia): 0.257108 g/mm^4

Lateral duct (long cilia): 0.296809 g/mm^4
Lateral duct (medium cilia): 0.296826 g/mm^4
Lateral duct (short cilia): 0.296844 g/mm^4

DAMPING

Common crus (common part): 4.707767 g/s.mm^4
Anterior utricle (common part): 0.237034 g/s.mm^4

Anterior duct (long cilia): 179.254201 g/s.mm^4
Anterior duct (medium cilia): 179.235530 g/s.mm^4
Anterior duct (short cilia): 179.217641 g/s.mm^4

Posterior duct (long cilia): 135.444611 g/s.mm^4
Posterior duct (medium cilia): 135.422394 g/s.mm^4
Posterior duct (short cilia): 135.400517 g/s.mm^4

Lateral duct (long cilia): 186.444267 g/s.mm^4
Lateral duct (medium cilia): 186.420765 g/s.mm^4
Lateral duct (short cilia): 186.397789 g/s.mm^4

INERTIAL FORCING

Anterior duct: 0.032149 g/mm
Posterior duct: 0.025583 g/mm
Lateral duct: 0.027362 g/mm

DYNAMIC PARAMETERS

TIME CONSTANTS

LONG TIME CONSTANTS

First constant (long cilia): 6.332924 s
First constant (medium cilia): 7.833594 s
First constant (short cilia): 10.160695 s

Second constant (long cilia): 3.221269 s
Second constant (medium cilia): 3.777175 s
Second constant (short cilia): 4.553211 s

Third constant (long cilia): 4.649966 s
Third constant (medium cilia): 5.572814 s
Third constant (short cilia): 6.921619 s

SHORT TIME CONSTANTS

First constant (long cilia): 1.623608 ms
First constant (medium cilia): 1.623814 ms
First constant (short cilia): 1.624016 ms

Second constant (long cilia): 1.927875 ms
Second constant (medium cilia): 1.928143 ms
Second constant (short cilia): 1.928409 ms

Third constant (long cilia): 1.583222 ms
Third constant (medium cilia): 1.583427 ms
Third constant (short cilia): 1.583629 ms

BANDWIDTH

LOWER CORNER FREQUENCIES

First frequency (long cilia): 0.025131 Hz
First frequency (medium cilia): 0.020317 Hz
First frequency (short cilia): 0.015664 Hz

Second frequency (long cilia): 0.049408 Hz
Second frequency (medium cilia): 0.042136 Hz
Second frequency (short cilia): 0.034954 Hz

Third frequency (long cilia): 0.034227 Hz
Third frequency (medium cilia): 0.028559 Hz
Third frequency (short cilia): 0.022994 Hz

UPPER CORNER FREQUENCIES

First frequency (long cilia): 98.025459 Hz
First frequency (medium cilia): 98.013017 Hz
First frequency (short cilia): 98.000817 Hz

Second frequency (long cilia): 82.554612 Hz
Second frequency (medium cilia): 82.543111 Hz
Second frequency (short cilia): 82.531736 Hz

Third frequency (long cilia): 100.526011 Hz
Third frequency (medium cilia): 100.512961 Hz
Third frequency (short cilia): 100.500115 Hz

IN-PLANE SENSITIVITY PARAMETERS

MECHANICAL GAIN

Anterior duct (cilia average deflection, long cilia): 1.477702 deg/deg
Anterior duct (cilia average deflection, medium cilia): 1.394188 deg/deg
Anterior duct (cilia average deflection, short cilia): 1.273231 deg/deg

Posterior duct (cilia average deflection, long cilia): 1.586132 deg/deg
Posterior duct (cilia average deflection, medium cilia): 1.538329 deg/deg
Posterior duct (cilia average deflection, short cilia): 1.462010 deg/deg

Lateral duct (cilia average deflection, long cilia): 1.515809 deg/deg
Lateral duct (cilia average deflection, medium cilia): 1.458204 deg/deg
Lateral duct (cilia average deflection, short cilia): 1.365316 deg/deg

VELOCITY GAIN

Anterior duct (cilia average deflection, long cilia): 0.002402 deg/deg.s-1
Anterior duct (cilia average deflection, medium cilia): 0.002267 deg/deg.s-1
Anterior duct (cilia average deflection, short cilia): 0.002070 deg/deg.s-1

Posterior duct (cilia average deflection, long cilia): 0.002951 deg/deg.s-1
Posterior duct (cilia average deflection, medium cilia): 0.002863 deg/deg.s-1
Posterior duct (cilia average deflection, short cilia): 0.002721 deg/deg.s-1

Lateral duct (cilia average deflection, long cilia): 0.002416 deg/deg.s-1
Lateral duct (cilia average deflection, medium cilia): 0.002325 deg/deg.s-1
Lateral duct (cilia average deflection, short cilia): 0.002177 deg/deg.s-1

ACCELERATION GAIN

Anterior duct (cilia average deflection, long cilia): 0.015119 deg/deg.s-2
Anterior duct (cilia average deflection, medium cilia): 0.017649 deg/deg.s-2
Anterior duct (cilia average deflection, short cilia): 0.020910 deg/deg.s-2

Posterior duct (cilia average deflection, long cilia): 0.009467 deg/deg.s-2
Posterior duct (cilia average deflection, medium cilia): 0.010766 deg/deg.s-2
Posterior duct (cilia average deflection, short cilia): 0.012335 deg/deg.s-2

Lateral duct (cilia average deflection, long cilia): 0.011241 deg/deg.s-2
Lateral duct (cilia average deflection, medium cilia): 0.012961 deg/deg.s-2
Lateral duct (cilia average deflection, short cilia): 0.015075 deg/deg.s-2

FUNCTIONAL PLANES

MAXIMAL RESPONSE PLANES

Right Anterior duct: 0.630360x 0.776294y 0.003545z
Right Posterior duct: 0.617687x -0.777280y 0.119575z
Right Lateral duct: -0.000000x -0.140964y -0.990015z
Left Anterior duct: -0.630360x 0.776294y -0.003545z
Left Posterior duct: -0.617687x -0.777280y -0.119575z
Left Lateral duct: 0.000000x -0.140964y 0.990015z

PRIME DIRECTIONS

Right Anterior duct: 0.786375x 0.611519y -0.087072z
Right Posterior duct: 0.768043x -0.624066y 0.088858z
Right Lateral duct: 0.095581x -0.073186y -0.969474z
Left Anterior duct: -0.786375x 0.611519y 0.087072z
Left Posterior duct: -0.768043x -0.624066y -0.088858z
Left Lateral duct: -0.095581x -0.073186y 0.969474z

IPSILATERAL ANGLES

Anterior and Posterior ducts: 102.341478 Degrees
Anterior and Lateral ducts: 83.521356 Degrees
Lateral and Posterior ducts: 90.511590 Degrees

SYNERGISTIC ANGLES

Anterior and Posterior ducts: 173.321161 Degrees
Lateral ducts: 163.804777 Degrees

REFERENCE ANGLES

VESTIBULAR PITCH / MID-SAGITTAL PLANE

Anterior duct: 39.080335 Degrees

Posterior duct: 38.990639 Degrees
Lateral duct: 81.902388 Degrees

VESTIBULAR ROLL / CORONAL PLANE

Anterior duct: 50.927031 Degrees
Posterior duct: 51.856403 Degrees
Lateral duct: 90.000000 Degrees

VESTIBULAR YAW / HORIZONTAL PLANE

Anterior duct: 90.209771 Degrees
Posterior duct: 83.138540 Degrees
Lateral duct: 8.104241 Degrees

DEVIATION FROM IDEAL CONFIGURATION

Variance from orthogonality: 64.848876 Degrees^2

Average deviation from orthogonality: 6.443904 Degrees

Variance from planarity: 117.166347 Degrees^2

Average deviation from planarity: 9.850967 Degrees

SEMICIRCULAR DUCT CENTER OF ROTATION

Right Anterior duct: 3886.289412x -2664.117642y 12264.755230z
Right Posterior duct: 1394.437964x -7676.491624y 10675.934140z
Right Lateral duct: 631.010250x -7004.518656y 6994.886066z
Left Anterior duct: -315.693292x 17168.516783y 12241.122758z
Left Posterior duct: -2723.062948x 11824.761147y 9878.846204z
Left Lateral duct: 631.010250x 15394.471315y 13594.324014z

MATERIAL PROPERTIES

Body temperature: 38.000000 C

Endolymph density: 1000.000000 kg/m^3
Endolymph viscosity: 0.000799 Pa.s
Endolymph viscosity factor: 2.850000 e-5 Pa.s

Cupula density: 1000.000000 kg/m^3
Cupula viscosity: 0.007993 Pa.s
Cupula Poisson ratio: 0.480000
Cupula Young modulus: 4.258000 Pa
Cupula Shear modulus: 1.438514 Pa

SATURATING MECHANICAL GAIN OF THE SEMICIRCULAR DUCT SYSTEM TO ROTATIONS IN SPACE

- MAXIMUM VALUE AMONG THE ABSOLUTE MECHANICAL GAINS OF THE SIX SEMICIRCULAR DUCTS, WEIGHTED ACCORDING TO INHIBITION/ACTIVATION
-- TO ANGULAR VELOCITY
--- MEASURED IN UNITS OF CILIA AVERAGE DEFLECTION

Nose-down Pitch (long cilia): 0.002294 deg/deg.s-1
Nose-down Pitch (medium cilia): 0.002225 deg/deg.s-1

Nose-down Pitch (short cilia): 0.002115 deg/deg.s-1

Nose-up Pitch (long cilia): 0.001865 deg/deg.s-1
Nose-up Pitch (medium cilia): 0.001760 deg/deg.s-1
Nose-up Pitch (short cilia): 0.001607 deg/deg.s-1

Roll (long cilia): 0.001823 deg/deg.s-1
Roll (medium cilia): 0.001768 deg/deg.s-1
Roll (short cilia): 0.001681 deg/deg.s-1

Yaw (long cilia): 0.002392 deg/deg.s-1
Yaw (medium cilia): 0.002302 deg/deg.s-1
Yaw (short cilia): 0.002155 deg/deg.s-1

Global mean (long cilia): 0.002159 deg/deg.s-1
Global mean (medium cilia): 0.002074 deg/deg.s-1
Global mean (short cilia): 0.001940 deg/deg.s-1

Global standard deviation (long cilia): 0.000329 deg/deg.s-1
Global standard deviation (medium cilia): 0.000326 deg/deg.s-1
Global standard deviation (short cilia): 0.000321 deg/deg.s-1

Global maximum (long cilia): 0.002951 deg/deg.s-1
Global maximum (medium cilia): 0.002863 deg/deg.s-1
Global maximum (short cilia): 0.002721 deg/deg.s-1

Global minimum (long cilia): 0.001303 deg/deg.s-1
Global minimum (medium cilia): 0.001245 deg/deg.s-1
Global minimum (short cilia): 0.001160 deg/deg.s-1

SATURATING MECHANICAL GAIN OF THE SEMICIRCULAR DUCT SYSTEM TO ROTATIONS IN SPACE

- MAXIMUM VALUE AMONG THE ABSOLUTE MECHANICAL GAINS OF THE SIX SEMICIRCULAR DUCTS, WEIGHTED ACCORDING TO INHIBITION/ACTIVATION
-- TO ANGULAR ACCELERATION
--- MEASURED IN UNITS OF CILIA AVERAGE DEFLECTION

Nose-down Pitch (long cilia): 0.007359 deg/deg.s-1
Nose-down Pitch (medium cilia): 0.008368 deg/deg.s-1
Nose-down Pitch (short cilia): 0.009588 deg/deg.s-1

Nose-up Pitch (long cilia): 0.011737 deg/deg.s-1
Nose-up Pitch (medium cilia): 0.013701 deg/deg.s-1
Nose-up Pitch (short cilia): 0.016232 deg/deg.s-1

Roll (long cilia): 0.009530 deg/deg.s-1
Roll (medium cilia): 0.011125 deg/deg.s-1
Roll (short cilia): 0.013181 deg/deg.s-1

Yaw (long cilia): 0.011129 deg/deg.s-1
Yaw (medium cilia): 0.012832 deg/deg.s-1
Yaw (short cilia): 0.014924 deg/deg.s-1

Global mean (long cilia): 0.010140 deg/deg.s-1
Global mean (medium cilia): 0.011722 deg/deg.s-1
Global mean (short cilia): 0.013700 deg/deg.s-1

Global standard deviation (long cilia): 0.001877 deg/deg.s-1
Global standard deviation (medium cilia): 0.002234 deg/deg.s-1

Global standard deviation (short cilia): 0.002715 deg/deg.s-1

Global maximum (long cilia): 0.015119 deg/deg.s-1
Global maximum (medium cilia): 0.017649 deg/deg.s-1
Global maximum (short cilia): 0.020910 deg/deg.s-1

Global minimum (long cilia): 0.005873 deg/deg.s-1
Global minimum (medium cilia): 0.006755 deg/deg.s-1
Global minimum (short cilia): 0.007780 deg/deg.s-1

STANDARDIZED MECHANICAL GAIN OF THE SEMICIRCULAR DUCT SYSTEM TO ROTATIONS IN SPACE

- OF THE THREE SYNERGISTIC SEMICIRCULAR DUCT PAIRS
-- TO ANGULAR VELOCITY
--- MEASURED IN UNITS OF CILIA AVERAGE DEFLECTION

Pitch (long cilia): 0.001960 deg/deg.s-1
Pitch (medium cilia): 0.001879 deg/deg.s-1
Pitch (short cilia): 0.001755 deg/deg.s-1

Roll (long cilia): 0.001573 deg/deg.s-1
Roll (medium cilia): 0.001507 deg/deg.s-1
Roll (short cilia): 0.001407 deg/deg.s-1

Yaw (long cilia): 0.001604 deg/deg.s-1
Yaw (medium cilia): 0.001543 deg/deg.s-1
Yaw (short cilia): 0.001445 deg/deg.s-1

Global mean (long cilia): 0.001690 deg/deg.s-1
Global mean (medium cilia): 0.001622 deg/deg.s-1
Global mean (short cilia): 0.001517 deg/deg.s-1

Global standard deviation (long cilia): 0.000132 deg/deg.s-1
Global standard deviation (medium cilia): 0.000127 deg/deg.s-1
Global standard deviation (short cilia): 0.000121 deg/deg.s-1

Global maximum (long cilia): 0.001960 deg/deg.s-1
Global maximum (medium cilia): 0.001879 deg/deg.s-1
Global maximum (short cilia): 0.001755 deg/deg.s-1

Global minimum (long cilia): 0.001500 deg/deg.s-1
Global minimum (medium cilia): 0.001439 deg/deg.s-1
Global minimum (short cilia): 0.001345 deg/deg.s-1

STANDARDIZED MECHANICAL GAIN OF THE SEMICIRCULAR DUCT SYSTEM TO ROTATIONS IN SPACE

- OF THE THREE SYNERGISTIC SEMICIRCULAR DUCT PAIRS
-- TO ANGULAR ACCELERATION
--- MEASURED IN UNITS OF CILIA AVERAGE DEFLECTION

Pitch (long cilia): 0.009002 deg/deg.s-2
Pitch (medium cilia): 0.010403 deg/deg.s-2
Pitch (short cilia): 0.012172 deg/deg.s-2

Roll (long cilia): 0.007249 deg/deg.s-2
Roll (medium cilia): 0.008379 deg/deg.s-2
Roll (short cilia): 0.009805 deg/deg.s-2

Yaw (long cilia): 0.007440 deg/deg.s-2
Yaw (medium cilia): 0.008578 deg/deg.s-2
Yaw (short cilia): 0.009976 deg/deg.s-2

Global mean (long cilia): 0.007803 deg/deg.s-2
Global mean (medium cilia): 0.009008 deg/deg.s-2
Global mean (short cilia): 0.010510 deg/deg.s-2

Global standard deviation (long cilia): 0.000502 deg/deg.s-2
Global standard deviation (medium cilia): 0.000582 deg/deg.s-2
Global standard deviation (short cilia): 0.000690 deg/deg.s-2

Global maximum (long cilia): 0.009002 deg/deg.s-2
Global maximum (medium cilia): 0.010403 deg/deg.s-2
Global maximum (short cilia): 0.012172 deg/deg.s-2

Global minimum (long cilia): 0.007048 deg/deg.s-2
Global minimum (medium cilia): 0.008143 deg/deg.s-2
Global minimum (short cilia): 0.009512 deg/deg.s-2
